# A phase II, multicenter, nonblinded, randomized controlled trial for evaluating protective effects of ABPC/SBT plus, azithromycin versus erythromycin, in pregnant women with pPROM occurring at <28 weeks of gestation on the development of BPD in neonates: Study protocol

**DOI:** 10.1371/journal.pone.0304705

**Published:** 2024-07-09

**Authors:** Akihide Ohkuchi, Kaoru Okazaki, Shintaro Iwamoto, Mayumi Sako, Tohru Kobayashi, Itaru Yanagihara, Makoto Nomiyama, on behalf of the group of the PPROM-AZM study

**Affiliations:** 1 Maternal and Fetal Neonatal Intensive Care Unit, Jichi Medical University Hospital, Tochigi, Japan; 2 Department of Neonatology, Tokyo Metropolitan Children’s Medical Center, Fuchu-shi, Tokyo, Japan; 3 Division of Biostatistics, Department of Data Science, Clinical Research Center, National Center for Child Health and Development, Setagaya-ku, Tokyo, Japan; 4 Department of Clinical Research Promotion, Clinical Research Center, National Center for Child Health and Development, Setagaya-ku, Tokyo, Japan; 5 Department of Data Science, Clinical Research Center, National Center for Child Health and Development, Setagaya-ku, Tokyo, Japan; 6 Department of Developmental Medicine, Research Institute, Osaka Women’s and Children’s Hospital, Izumi-shi, Osaka, Japan; 7 Department of Obstetrics and Gynecology, National Hospital Organization Saga Hospital, Saga-shi, Saga, Japan; Kobe University Graduate School of Medicine School of Medicine, JAPAN

## Abstract

This is a protocol for PPROM-AZM Study, phase II, nonblinded, randomized controlled trial. Bronchopulmonary dysplasia (BPD) at a postmenstrual age of 36 weeks (BPD_36_) is often observed in infants with preterm premature rupture of the membranes (pPROM). A regimen of ampicillin (ABPC) intravenous infusion for 2 days and subsequent amoxicillin (AMPC) oral administration for 5 days plus erythromycin (EM) intravenous infusion for 2 days followed by EM oral administration for 5 days is standard treatment for pPROM. However, the effect on the prevention of moderate/severe BPD_36_ using the standard treatment has not been confirmed. Recently, it is reported that ampicillin/sulbactam (ABPC/SBT) plus azithromycin (AZM) was effective for the prevention of moderate/severe BPD_36_ in pPROM patients with amniotic infection of *Ureaplasma* species. Therefore, our aim is to evaluate the occurrence rate of the composite outcome of “incidence rate of either moderate/severe BPD_36_ or intrauterine fetal death, and infantile death at or less than 36 weeks 0 days” comparing subjects to receive ABPC/SBT for 14 days plus AZM for 14 days (intervention group) and those to receive ABPC/SBT for 14 days plus EM for 14 days (control group), in a total of 100 subjects (women with pPROM occurring at 22–27 weeks of gestation) in Japan. The recruit of subjects was started on April 2022, and collection in on-going. We also investigate the association between the detection of *Ureaplasma* species and occurrence of BPD_36_. In addition, information on any adverse events for the mother and fetus and serious adverse events for infants are collected during the observation period. We allocate patients at a rate of 1:1 considering two stratification factors: onset of pPROM (22–23 or 24–27 weeks) and presence/absence of a hospital policy for early neonatal administration of caffeine.

**Trial registration:** The trial number in the Japan Registry of Clinical Trials is jRCTs031210631.

## Introduction

### Association between pPROM and BPD

Preterm premature rupture of the membranes (pPROM) is defined as PROM occurring at <37 weeks of gestation. In Japan, preterm birth incidence has been approximately 6% for the past 10 years. The rate of preterm birth at less than 28 weeks of gestation (extremely preterm birth) is about 0.3%. As there were almost 940,000 births in 2017, about 2,800 extremely preterm infants were delivered annually. The proportion of pPROM in extremely premature cases in Japan was 30.0% at 22–23 weeks, 31.7% at 24–25 weeks, and 25.8% at 26–27 weeks in 2013 [1]. Therefore, it is estimated that about 700 children were extremely preterm birth infants born to women with pPROM occurring at <28 weeks of gestation in 2017.

Since pPROM increases the risk of intrauterine infection due to ascending infection, antibiotics are administered for prevention. pPROM is associated with high perinatal mortality [2, 3]. In addition, there are many kinds of neonatal complications in infants born to women with pPROM: pulmonary hypoplasia due to oligohydramnios, bronchopulmonary dysplasia (BPD), dry lung syndrome, amniotic band syndrome, neonatal asphyxia, persistent pulmonary hypertension in the neonate, intraventricular hemorrhage (IVH), acute inflammatory disease due to amniotic infection such as sepsis, pneumonia, and meningitis, fetal inflammatory response syndrome such as chronic lung disease, necrotizing enterocolitis (NEC), periventricular leukomalacia (PVL), and long-term neurodevelopment impairments.

BPD is a chronic lung disease occurring in preterm newborn infants. It can interfere with normal lung development and require long-term mechanical ventilation and/or oxygen administration. Diagnosis of BPD at a postmenstrual age (PMA) of 36 weeks (BPD_36_) is based on oxygen demand, i.e., required ventilatory assistance over a long period (≥28 days), or oxygen administration and ventilatory assistance at 36 weeks PMA, although the definition of BPD_36_ is slightly different for infants delivered at <32 and ≥32 weeks of gestation [4]. The severity of BPD_36_ is classified as mild, moderate, and severe according to the delivered gestational age (either at <32 or ≥32 weeks of gestation), treatment with oxygen >21% for at least 28 days, breathing room air at 36 weeks PMA, oxygen concentration, positive airway pressure, and intubation [4]. A report summarizing the association between the presence/absence of recent *Ureaplasma* infection and BPD_36_ suggests prevalence in about 30–50% of newborn babies at less than 28 weeks gestation or less than 1,500 g [5, 6]. BPD_36_ morbidity is significantly linked to earlier weeks at delivery, decreasing at later weeks at delivery. Recent analyses using the Neonatal Research Network database Japan (http://plaza.umin.ac.jp/nrndata/) reported that the incidence of BPD_36_ in surviving discharged children delivered at 22, 23, 24, 25, 26, 27, 28, 29, and 30 weeks of gestation was 76, 72, 67, 58, 50, 41, 32, 24, and 17%, respectively. Because these data included infants not complicated with pPROM, we estimate that the incidence of BPD_36_ in infants with pPROM may be higher, because pPROM is an independent risk factor for BPD_36_ occurrence after adjusting for the gestational age in weeks, birth weight, antenatal steroids, inborn delivery, multiple birth, center, sex, and small-for-gestational-age infant [7]. We analyzed 55 pregnant women with singleton pregnancy who had pPROM at <28 weeks of gestation, and delivered between 22 and 31 weeks of gestation, and we investigated the association between pPROM and the subsequent occurrence of short- and long-term prognoses; perinatal death occurred in 13% (intrauterine death: 6%, early neonatal death: 7%), pulmonary hypoplasia in 14%, respiratory distress syndrome (RDS) in 56%, transient tachypnea of newborn (TTN) in 42%, NEC in 6%, IVH in 10%, PVL in 10%, sepsis in 4%, BPD at 28 days of life (BPD_28_) in 72%, and BPD_36_ in 60% [8]. Long-term neurodevelopmental impairments, which were defined as either cerebral palsy or developmental delay, evaluated at 1.5 and/or 3 years old, occurred in 36% [8]. Some infants with moderate/severe BPD_36_ show continued respiratory failure beyond the neonatal period, and they need long-term oxygen and/or respiratory support. On entering school, children with moderate/severe BPD_36_ showed increased risks of cognitive, language, and executive dysfunction, academic achievement limitations, social skill deficits, and low scores on assessments of the health-related quality of life [9].

Since supportive care such as oxygen therapy is the only treatment for BPD, the prevention of BPD is strongly desired. Because pPROM is a risk factor for the occurrence of BPD_36_ [7], and because chorioamnionitis is associated with the occurrence of moderate/severe BPD_36_ [10], it is very important for women with pPROM occurring at <28 weeks of gestation to receive appropriate antibiotics, and to prolong the pregnancy period.

### Standard treatment for women with pPROM

Standard treatment was not recommended in the guidelines for obstetrical practice in the Japan 2020 edition [11]. However, in the guidelines published in the United States [12], the regimen of ampicillin (ABPC) 2 g intravenous infusion 4x/day for 2 days and subsequent amoxicillin (AMPC) 250 mg oral administration 3x/day for 5 days plus erythromycin (EM) 250 mg intravenous infusion 4x/day for 2 days followed by EM 333 mg oral administration 3x/day for 5 days was recommended; in the guidelines published in England [13], the regimen of EM 250 mg oral administration 4x/day for 10 days or until the onset of labor was recommended; and in the guidelines published in Canada [14], the regimens of either ABPC 2 g intravenous infusion 4x/day for 2 days and subsequent AMPC 250 mg oral administration 3x/day for 5 days plus EM 250 mg intravenous infusion 4x/day for 2 days followed by EM 333 mg oral administration 3x/day for 5 days or EM 250 mg oral administration 4x/day for 10 days were recommended. As for ampicillin/sulbactam (ABPC/SBT), only one randomized controlled trial (RCT) has compared the three groups of ABPC/SBT followed by AMPC-clavulanate, ABPC followed by AMPC, and placebo in women with singleton pregnancies at 23–35 weeks of gestation with pPROM; the incidence rates of neonatal mortality, sepsis, and RDS were significantly lower in the ABPC/SBT group than in the placebo group; these effects in the ABPC/SBT group were comparable to those in the ABPC group [15].

Meta-analysis of antibiotic effects on mothers and infants with pPROM was reported in 2013 [16]: chorioamnionitis, parturition within 48 hours after pPROM, delivery within 7 days, newborn infection, surfactant use, oxygen therapy, and an abnormal cerebral echogram prior to discharge were significantly decreased in the group receiving antibiotics compared with a control group [16]. However, meta-analysis of antibiotic RCT for women with pPROM reported in 2008 showed no significant difference for moderate/severe BPD_36_ [17]. Thus, although preventive treatment for pPROM mainly with ABPC has a certain effect on prevention of infantile infectious disease development, the treatment may have no effect on the prevention of BPD. It was recently discovered that pulmonary colonization by *Ureaplasma* species (spp.) was significantly involved in BPD onset in preterm infants [5]. Azithromycin (AZM) was effective for *Ureaplasma* spp. [18], and, moreover, AZM was effectively transferred from a pregnant woman to the placenta and amniotic cavity [19]. However, to the best of our knowledge, there has been no RCT on whether BPD can be prevented by administering AZM to pregnant women who developed pPROM at <28 weeks of gestation.

We recently compared two different regimens for pregnant women with pPROM occurring at <28 weeks of gestation chronologically. The incidence of moderate/severe BPD_36_ in the standard treatment period predominantly with piperacillin + EM was 71% (10/14), and in interventional treatment mainly with ABPC/SBT + AZM, the incidence significantly decreased to 14% (2/14) [20]. These results suggest that the administration of AZM to pPROM patients with intra-amniotic *Ureaplasma* infection may markedly reduce the subsequent occurrence of BPD. Although the usage of AZM for pPROM is not recommended as the standard treatment for pPROM in any guidelines for the management of pPROM [12–14], it is stated that AZM could be used in a situation in which EM is not available or not tolerated in the guidelines published in the United States [12].

### Aims of the phase II, multicenter, nonblinded, randomized controlled trial

The aims of this trial are: (1) to investigate the effectiveness and safety of AZM and EM under the co-administration of ABPC/SBT to prevent BPD in infants who are delivered by pregnant women with pPROM occurring at <28 weeks of gestation, and to calculate the sample size requirements for a full-scale clinical trial powered at 80% to determine a clinically important difference in the occurrence rate of BPD between AZM and EM treatments, (2) to investigate the association between the detection of *Ureaplasma* spp. in the vagina, on the surface of the placenta, in the neonatal throat, and in the neonatal ear cavity and the occurrence of BPD in infants who are delivered by pregnant women with pPROM occurring at <28 weeks of gestation. Because it is very difficult to set the same administration intervals for AZM and EM, and because we could not calculate an appropriate sample size for this trial mainly due to lack of appropriate previous research, we decided to perform this trial as a phase II, multicenter, nonblinded, randomized controlled trial.

## Materials and methods

### Ethics approval

The original protocol (Version 2.2) had reviewed and approved by Jichi Medical University Central Clinical Research Ethics Committee (ID 21JMU001M; approval date: January 25, 2022) and the Japanese Ministry of Health, Labour and Welfare (approval date: February 22, 2022). This treatment protocol has been performed in accordance with Clinical Trial Act enacted in 2019 in Japan, which is in accordance with the Declaration of Helsinki. This treatment protocol was not designed to satisfy market approval requirements. Therefore, the national drug regulatory agency (Pharmaceuticals and Medical Devices Agency) did not supervise this treatment protocol. All methods have been carried out in accordance with relevant guidelines and regulations. The current protocol version was 2.9 created on Novenber 29, 2023, and was approved by the Ethics Committee on December 19, 2023 (**S1 Appendix**: A copy of the protocol, written by Japanese; **S2 Appendix**: A translation to English of the protocol). We published the protocol and research explanatory document on the home page of the Department of Obstetrics and Gynecology, Jichi Medical University, and anyone can access and read the documents. The trial number in the Japan Registry of Clinical Trials is jRCTs031210631.

### Study design

This is a phase II, multicenter, nonblinded, randomized controlled trial. Our trial is based on a prospectively registered protocol, and we report our findings in accordance with the Standard Protocol Items: Recommendations for Interventional Trials (SPIRIT) (**S1 Checklist**).

### Setting of the study

Tertiary maternal and fetal neonatal units and neonatal intensive care units (NICU) in Japan has been participated in this study.

### Definition of investigator in this trial

#### Principal investigator (PI)

When conducting the multicenter trial, he/she is a researcher representing the chief investigators of multiple medical institutions.

#### Chief investigator (CI)

When conducting the multicenter trial, he/she is a doctor or dentist who conducts the clinical trial as stipulated by law, and supervises the work related to the clinical trial at the implementing medical institution.

#### Sub-investigators (SIs)

When conducting the multicenter trial, they are doctors or dentists who share the work related to the clinical trial under the guidance of CI at the implementing medical institution.

#### Clinical research coordinators

They are pharmacists, nurses, and other medical persons who cooperate in clinical trial work under PI (or CIs) and SIs at the implementing medical institution.

### Eligibility criteria

#### Eligibility assessment

We collect patients’ background information during the screening period before entry: age, ethnicity, blood type (ABO, Rh), smoking habit, day of consent, gestational weeks and days at entry into this trial, gravidity, parity, therapy for sterility, height, body weight (pre-pregnancy, on giving consent), basic complications, any past obstetrical histories, presence/absence of allergy (food, drugs), onset date (gestational weeks and days) of pPROM, findings of pPROM (presence/absence of leak alone, pooling alone, or both leak and pooling). We also examine patients, and check maternal and fetal conditions, vital signs (blood pressure, pulse, and axillary temperature). We take blood samples to measure the white blood cell count, red blood cell count, hemoglobin concentration, hematocrit value, platelet count, leukocyte fraction, total protein, albumin, total bilirubin, aspartate aminotransferase, alanine aminotransferase, lactate dehydrogenase, blood urea nitrogen, creatinine, uric acid, natrium, potassium, chloride, calcium, magnesium, amylase, creatine kinase, and C-reactive protein during the screening period. We also collect single voided urine to determine the presence/absence of either urine protein or urine sugar. We perform an electrocardiogram to rule out long QT syndrome, with which the administration of EM may lead to ventricular tachycardia. We measure the estimated fetal weight and amniotic fluid index. We check for any drugs which patients have been using until the screening period. The information is input in an electric data capture (EDC) before judging the appropriateness of the candidates for this trial. The completion of all data input was the necessitate for starting RCT allocation.

#### Inclusion criteria

1. Pregnant woman who gave consent to participate in the current trial.
2. Pregnant woman whose age at the time of receiving informed consent is equal to or over 18 years old.
3. Pregnant woman with a singleton pregnancy who developed pPROM at 22 weeks 0 days—27 weeks 6 days.

#### Exclusion criteria

1. Pregnant woman with placenta previa.
2. Pregnant woman with trachelectomy; however, pregnant woman with cervical incision should not be excluded, because they are at high risk for the occurrence of pPROM.
3. Pregnant woman with hypertensive disorders of pregnancy; however, pregnant woman with chronic hypertension should not be excluded.
4. Pregnant woman with a fetus who shows either absent end-diastolic velocity or reverse end-diastolic velocity before the onset of pPROM.
5. Pregnant woman with any malignant tumors for whom the attending physicians consider the termination of pregnancy at or soon after the onset of pPROM.
6. Pregnant woman with pPROM for whom the attending physicians consider the imminent termination of pregnancy at or soon after the onset of pPROM.
7. Pregnant woman with pPROM who has had diseases or symptoms causing attending physicians to consider the imminent termination of pregnancy at or soon after the onset of pPROM.
8. Pregnant woman with the administration of AZM due to *Chlamydia* infection within 2 weeks before the onset of pPROM.
9. Pregnant woman receiving any kinds of antibiotics other than either penicillin or cephem antibiotics for two weeks before the onset of pPROM.
10. Pregnant woman who has been administered antibiotics rather than either ABPC or ABPC/SBT, from the onset of pPROM to the time obtaining consent; However, the following pregnant woman should not be excluded: when the administration of antibiotics rather than either ABPC or ABPC/SBT (excluding any kinds of macrolide antibiotics) were performed one day before the time of obtaining consent or earlier and the administration period of them were within 3 days.
11. Pregnant woman with the administration of any kinds of macrolide antibiotics, from the onset of pPROM to the time obtaining consent.
12. Pregnant woman with allergy for either penicillin or macrolide antibiotics.
13. Pregnant woman complicated with infectious mononucleosis.
14. Pregnant woman with a fetus with either chromosomal abnormalities or suspected severe malformation syndrome.
15. Pregnant woman with a creatinine level of ≥1.1 mg/dL at the screening test just after obtaining consent.
16. Pregnant woman complicated with QT syndrome.
17. Pregnant woman with any types of psychiatric disorders for whom the attending physicians think that the patient should not participate in the current trial.
18. Pregnant woman for whom the attending physicians think that the patient should not participate in the current trial, because there are some factors that may hamper the achievement of the trial.

### Interventions

Intervention group (AZM administration group)

1. ABPC/SBT (Sulbacillin^®^) 1.5 g intravenous infusion (div.) 4x/day 14 days (from Days 1 to 14).
2. AZM (Zithromax^®^) 500 mg div. 1x/day 4 days (on Days 1, 2, 8, and 9).
3. AZM (Zithromax^®^) 250 mg tablets orally 1x/day 10 days (from Days 3 to 7, and from Days 10 to 14).

Control group (standard antibiotic administration group, EM administration group)

1. ABPC/SBT (Sulbacillin^®^) 1.5 g div. 4x/day 14 days (from Days 1 to 14).
2. EM (Erythrocin^®^) 500 mg div. 2x/day 4 days (on Days 1, 2, 8, and 9).
3. EM (Erythrocin^®^) 200 mg tablets orally 4x/day 10 days (from Days 3 to 7, and from Days 10 to 14).

### Outcomes

The primary outcome is the composite outcome of “incidence rate of either moderate/severe BPD_36_ or intrauterine fetal death (IUFD), and infantile death at or less than 36 weeks 0 days PMA”.

Secondary outcomes are as follows (A-E):

A. Efficacy evaluation items for mother:

days from the onset of pPROM to delivery, gestational weeks at delivery, reasons for pregnancy termination, findings at delivery (mode of delivery, presence/absence of stillbirth, placental weight), incidence rate of IUFD, incidence rate of histological chorioamnionitis, rate of cesarean delivery, incidence rate of placental abruption.

B. Efficacy evaluation items for infant:

incidence rate of moderate/severe BPD_36_, incidence rate of BPD_28_, perinatal death rate (total of IUFD + early neonatal death / total of neonatal live infants), rate of infantile death until just before discharge to home of the infants, birth weight, birth height, birth head circumference, day of hospitalization (from the birth date to discharge to home), day of invasive ventilation, day of non-invasive ventilation, day of oxygen administration, incidence rate of infants with surfactant administration, incidence rate of infants with RDS, incidence rate of infants with pulmonary hypertension in the neonate, incidence rate of infants with IVH, incidence rate of infants with PVL, incidence rate of infants with sepsis, incidence rate of infants with NEC, incidence rate of infants with symptomatic patent ductus arteriosus.

C. Safety evaluation items for mother and fetus:

incidence rates of adverse events or side effects, incidence rates of serious adverse events or serious side effects, incidence rates of critical adverse events (IUFD, sepsis of mother, admission to intensive care unit of mother, multiple organ disorders of mother, artificial ventilation for mother, hysterectomy for mother).

D. Safety evaluation items for infants:

incidence rates of serious adverse events.

E. Other evaluation items in the current trial:

detection rate of *Ureaplasma* spp. (in vagina, on surface of the placenta, in infantile pharynx, and in infantile ear cavity).

### Schedule of enrollment intervention and assessments

**Fig 1:**
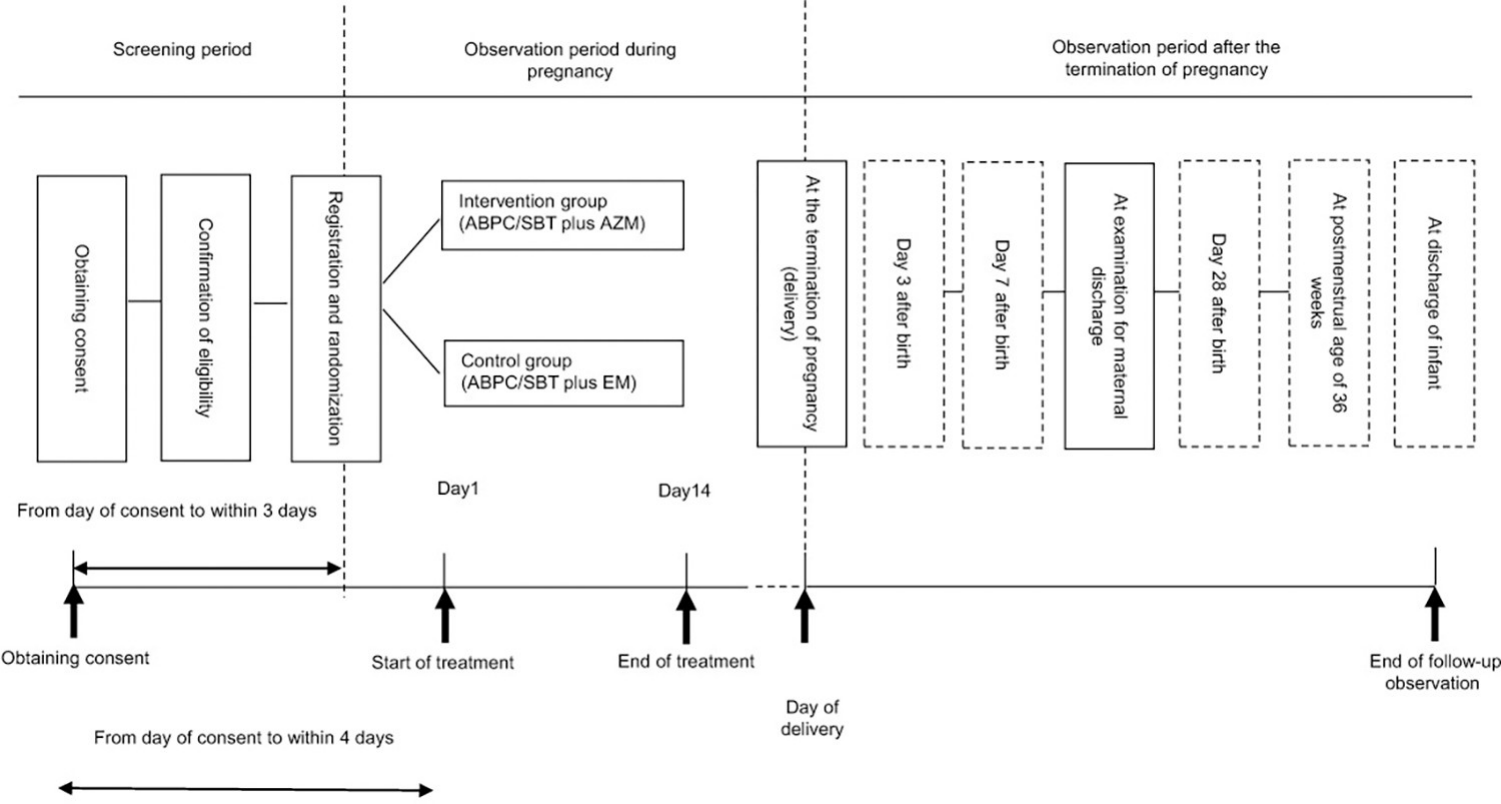
Schedule of enrollment intervention and assessments. CI or SIs must obtain written consent by the screening test. The maximum screening period (from consent acquisition to registration / randomization) is 3 days. CI or SIs must start protocol treatment within 4 days of obtaining consent. Predetermined observations, investigations, and tests must be performed prior to administration of the study drug. If the decision to terminate the pregnancy is made before the protocol treatment is completed, the protocol treatment must be terminated at that point, and the patient shifts to the post-pregnancy observation period after delivery.

The schedule of enrollment intervention and assessments is shown in **Fig 1**.

### Sample size

We set the target sample size as 100 cases (intervention group: 50 cases, control group: 50 cases) of women with singleton pregnancy complicated with pPROM occurring at 22–27 weeks of gestation, in view of the feasibility during the two-year recruitment period. If the samples will be collected too small during the two-year recruitment period, we will plan extension of the recruitment period for collecting more.

### Allocation and blinding

After quality assurance, the randomization process must be started.

Randomization is performed in order of entry, using Research Electronic Data Capture (REDCap^®^), which is an EDC System built in Vanderbilt University, USA. After confirming all the inclusion criteria during the screening period, REDCap^®^ allocates patients at a rate of 1:1, to either the intervention group or control group, using two stratification factors: onset of pPROM (22–23 or 24–27 weeks) and presence/absence of a hospital policy for the early neonatal administration of caffeine, because these two are confounding factors for the occurrence of BPD [21, 22]. We investigate whether the director of the NICU in the research participation facility has a priori policy to perform early neonatal administration of caffeine to prevent the occurrence of BPD. After starting the entry, we do not change the fixed allocation for early neonatal administration of caffeine for the research participation facilities regardless of the true usage of caffeine during the recruitment period.

We do not blind trial subjects, care providers, or data analysts, because it is very difficult to mask the selection of either AZM or EM for participants. However, due to the open-label allocation of study drugs in this study, the diagnosis of BPD may be biased by the PI (or CI) and SIs. Therefore, in order to reduce the diagnostic bias as much as possible and guarantee the reliability of the BPD diagnosis in this study, we decided to blind the assignment of study drugs to third parties who make the final diagnosis of primary outcome. This study design is called as Prospective Randomized Open Blinded End-point study design (PROBE). We set up “BPD Diagnosis and Evaluation Committee”. Diagnosis of BPD and evaluation of severity of BPD is judged by 3 medical experts working in tertiary NICU who are not participating in this research, who do not know to which group the subject is assigned. The diagnosis by the Committee is the final diagnosis in this study. In addition, the BPD diagnoses by the Committee is masked for CI and SIs until the completion of the trial.

### Clinical data collection and patient management

In accordance with the schedule (**Tables 1 and 2**), necessary observations and inspections, etc., are performed on each prescribed date. Observation, investigation, and examination of the protocol treatment start date (Day 1) are performed from waking up to within 2 hours after administration of test drugs in the morning. CI or SI should check the mother for the occurrence of adverse events, during the treatment observation period, observation period after the termination of pregnancy, at the time of cancelation of participation in this trial, and during the follow-up period after the cancelation of participation in this trial. In addition, CI or SI should check the infant for the occurrence of serious adverse events, during the postnatal observation period until discharge to home, and at the time of cancelation of participation in this trial.

**Table 1:** Schedule of data collection and monitoring for participant (mother and fetus).

Period	Screening period^a^		Observation period during pregnancy^b^								Observation period after the termination of pregnancy		At the cancelation of the participation in the trial^d^	Follow-up observation after the cancelation of participation in the trial^e^
Working item	Screening period Days -3 to 0	Registration Day 0	Week 1 Day 1	Week 2 Day 8 ± 2 day2	Week 3 Day 15 ± 2 days	Week 4 Day 22 ± 2 days	Week 5 Day 29 ± 2 days	Week 6 Day 36 ± 2 days	Week 7 Day 43 ± 2 days	Week 8~^c^ Day 50 ± 2 days	At the termination of pregnancy (delivery) ± 1 day	At examination for maternal discharge ± 2 days		
Obtaining consent^f^	X													
Participant: background information · body weight	X°													
Registration · randomization		X												
Care in hospital^**g**^			X	X	X	X	X	X	X	X	X	X		
Protocol treatment^**h**^			X	X										
Physical findings^**i**^	X		X	X	X	X	X	X	X	X	X	X	X	
Vital signs	X		X	X	X	X	X	X	X	X	X	X	X	
(blood pressure · pulse · body temperature)^**i**^														
Hematological examination^**i**^	X^p^			X	X	X	X	X	X	X	X	X	X	
Blood biochemical test^**i**^	X^p^			X	X	X	X	X	X	X	X	X	X	
Urinalysis	X^p^			X	X	X	X	X	X	X	X	X	X	
(Qualitative: protein · sugar)^**i**^														
ECG	X^p^													
Culture of vaginal microorganisms (each institute)^**i**^			X^**r**^	X^**s**^	X	X	X	X	X	X	X			
Culture of vaginal *Ureaplasma* spp.^**j**^			X^**r**^	X^**s**^										
PCR for vaginal *Ureaplasma* spp.^**j**^			X^**r**^	X^**s**^										
Culture of *Ureaplasma* spp. on the surface of the placenta^**j**^											X			
PCR for *Ureaplasma* spp. on the surface of the placenta^**j**^											X			
Histological examination for placenta											X			
Estimated fetal weight^**i**^	X^q^			X	X	X	X	X	X	X				
Fetal ultrasonography (Doppler · amniotic fluid volume estimation)^**i**^	X^q^			X	X	X	X	X	X	X				
Confirmation of drug used concomitantly^**k**^	X		X	X	X	X	X	X	X	X	X	X	X	X
Confirmation of adverse events^**l**^			X	X	X	X	X	X	X	X	X	X	X	X
Survey for the reasons of the termination of pregnancy^**m**^											X			
Findings on delivery^**n**^											X			

a. The maximum length of screening period is 3 days.

b. The planned interview, physical examination, ultrasonography, and sampling of biological specimens should be performed before the administration of test drugs. If the chief investigator (CI) or sub-investigator (SI) decides to terminate the pregnancy before the completion of protocol treatment, he/she should finish protocol treatment at the time of the decision, and will transfer to the observation period after the termination of pregnancy.

c. After week 8, CI or SI should perform the planned interview, physical examination, ultrasonography, and sampling of biological specimens once a week until the day of the termination of pregnancy.

d. CI or SI should perform the physical examination, sampling of biological specimens, and confirmation of drugs used concomitantly and adverse events within 3 days after the cancelation of participation in the trial.

e. CI or SI should perform follow-up observation after the cancelation of participation in the trial at the one-month postpartum medical examination.

f. CI or SI must obtain consent before starting examination during the screening period.

g. CI or SI should care for patients in hospital at least for the period from the start of protocol treatment to 3 days after delivery.

h. CI or SI must start protocol treatment within 4 days after obtaining consent.

i. CI or SI should examine the estimated fetal weight once a week at or after Week 3, until the decision to terminate the pregnancy. However, the hematological examination and blood biochemical test should be performed only when needed at or after Week 4, until the decision to terminate the pregnancy.

j. CI or SI should not take biological specimens from patients if the patients become complicated with COVID-19 infection. In addition, all preserved specimens should be abandoned.

k. If CI or SI decides to cancel participation in the trial due to the adverse events, CI or SI should continue the investigation of adverse events due to the test drugs or drugs used concomitantly which are the reasons for the cancelation of participation in the trial, from the cancelation to the one-month postpartum medical examination.

k. If CI or SI decides to cancel participation in the trial due to the adverse events, CI or SI should continue the investigation of adverse events due to the test drugs or drugs used concomitantly which are the reasons for the cancelation of participation in the trial, from the cancelation to the one-month postpartum medical examination.

l. CI or SI should investigate the occurrence of adverse events in the subject (mother and fetus). As for the mother, CI or SI should investigate from the starting day of protocol treatment to the observation period after the termination of pregnancy (or day of cancelation of participation in the trial). As for the fetus, CI or SI should investigate from the starting day of protocol treatment to the termination of pregnancy (or day of cancelation of participation in the trial). However, if CI or SI decides to cancel participation in the trial due to the appearance of adverse events, CI or SI should continue the investigation of adverse events due to the test drugs or drugs used concomitantly which are the reasons for the cancelation of participation in the trial, from the cancelation to the one-month postpartum medical examination

m. CI or SI should investigate the decision day of the termination of pregnancy, the time (day, hour, minute) of delivery, the reasons for termination of the pregnancy (both maternal and fetal causes).

n. CI or SI should investigate the delivery mode, presence/absence of stillbirth and the day of stillbirth, placental weight, and presence/absence of placental infarction.

o. CI or SI can use the data for body weight which is measured and recorded appropriately within 14 days before obtaining consent instead of body weight during screening period.

p. CI or SI can use the data for hematological examination, blood biochemical test, urinalysis, and ECG, which are measured and recorded appropriately within 3 days before obtaining consent instead of those during screening period.

q. CI or SI can use the data for estimated fetal weight, and fetal ultrasound (amniotic fluid volume estimation), which are measured and recorded appropriately within 7 days before obtaining consent instead of those during screening period.

r. The sampling of vaginal flora should be performed before the starting of protocol treatment. It is possible to perform it during the screening period.

s. CI or SI should perform the sampling of vaginal flora for the culture of *Ureaplasma* spp. or PCR of *Ureaplasma* spp. on Day 8 ± 2 days. However, if CI or SI finishs the protocol treatment before Day 8, CI or SI should not perform these samplings.

**Table 2:** Schedule of data collection and monitoring for participant (infant).

Working item	At the termination of pregnancy (delivery)	Day 3 after birth (± 1 day)	Day 7 after birth (± 3 days)	Day 28 after birth^†^ (± 2 days)	postmenstrual age of 36 weeks^#^ (± 2 days)	At discharge of infant (± 7 days)	At the cancelation of participation in the trial^a^
Umbilical blood gas analysis	X						
Neonatal findings^**b**^	X						
Apgar scores^**c**^	X						
Blood gas analysis^**d**^	X	X ^**k**^					
Hematological examination^**d**^	X ^**i**^	X ^**k**^					
Blood biochemical test^**d**^	X^**i**^^**、**^^**j**^	X^**k**^					
Culture of *Ureaplasma* spp. in throat^**e**^	X						
PCR for *Ureaplasma* spp. in throat^**e**^	X						
Culture of *Ureaplasma* spp. in ear cavity^**e**^	X						
PCR for *Ureaplasma* spp. in ear cavity^**e**^	X						
Chest X-ray	X		X^**l**^	X	X^**l**^		
Total admission days	X	X	X	X	X	X	
Presence/absence, kinds, and duration of respiratory management	X	X	X	X	X	X	
Presence/absence of BPD_36_					X^**m**^		
Presence/absence of BPD_28_				X			
Neonatal diseases^**f**^	X	X	X	X	X	X	
Treatment for infant^**g**^	X	X	X	X	X	X	
Serious adverse events^**h**^	X	X	X	X	X	X	X

† Chief investigator (CI) or sub-investigator (SI) should perform data collection and monitoring at the one-month medical checkup, when the infant discharges within 28 days after birth.

# As for infants with birth at gestational ages <32 weeks PMA, the assessment of BPD_36_ should be performed at 36 weeks PMA or discharge to home, whichever comes first. As for infants with birth at gestational ages ≥32 weeks PMA, the assessment of BPD_36_ should be performed at ≥28 and <56 days postnatal age or discharge to home, whichever comes first.

a. CI or SI should investigate serious adverse events of the infant within 3 days after the cancelation of participation in this trial.

b. CI or SI should investigate the neonatal sex, birth weight (g), birth height (cm), head circumference at birth (cm), and presence/absence of small for dates, light for dates, large for dates, and heavy for dates.

c. The Apgar score should be recorded at 1 and 5 minutes after delivery.

d. The sampling of blood for the blood gas test is acceptable from either artery, vein, or capillary vessels.

e. CI or SI should not take biological specimens from the infant if the mother becomes complicated with COVID-19 infection.

f. CI or SI should investigate the presence/absence of intracranial hemorrhage (ICH), periventricular leukomalacia (PVL), respiratory distress syndrome (RDS), meconium aspiration syndrome (MAS), transient tachypnea in newborns (TTN), prolonged pulmonary hypertension (PPH), sepsis, necrotizing enterocolitis (NEC), local intestinal perforation, symptomatic patent ductus arteriosus (PDA), subglottic stenosis, laryngomalacia,.and other upper airway diseases. CI or SI should also investigate the presence/absence of surgery for the infant. If the infant has any surgeries, CI or SI should investigate the surgical procedure, day of surgery, aim of surgery, and execution of intubation for the surgery.

g. CI or SI should investigate oxygen administration, intravenous/intratracheal administration of steroid, nitrogen monoxide inhalation, surfactant administration, caffeine administration, and azithromycin (AZM) treatment.

h. CI or SI should investigate serious adverse events of infants from the termination of pregnancy to discharge of the infant (or day of cancelation of participation in the trial for the mother).

i. The hematological examination and blood biochemical test at the termination of pregnancy for the infant are possible using cord blood.

j. The examination of IgM is performed only at delivery.

k. The blood gas test, hematological examination, and blood biochemical test should not be performed if the infant’s birth weight is under 1,000 g.

l. Chest X-ray at 7 and 28 days after birth should be performed when needed.

m. As for an infant delivered at or after 32 weeks of gestation, the evaluation of BPD_36_ should be performed at 56 days after delivery or at discharge to home, whichever comes first.

CI or SI performs a screening test after obtaining consent to evaluate eligibility. CI or SI enrolls the subject in REDCap^®^ prior to the start of protocol treatment. After enrollment, a subject who is determined to be eligible is assigned to the intervention or control group by stratification. The maximum screening period from consent acquisition to randomization is 3 days.

CI or SI starts protocol treatment within 4 days after the date of consent. The starting date of the study drug AZM 500 mg for intravenous infusion or EM intravenous infusion 500 mg is set as Day 1. CI or SI must start this trial under hospitalization management.

The protocol treatment period is 14 days from Days 1 to 14. However, if the decision to terminate the pregnancy is made before the 14-day administration is completed, the protocol treatment is terminated at that point.

From the completion or discontinuation of protocol treatment to the termination of pregnancy, CI or SI performs prescribed observations, surveys, and tests under hospitalization control.

CI or SI performs the examinations at the termination of pregnancy, at the time of discharge examination of the mother, at 28 days of life, at 36 weeks and 0 days PMA, and at the time of discharge of the child, and then he/she declares the completion of follow-up observation. Even after the termination of pregnancy, the safety of the subjects (mother, infant) should be ensured by in- or out-patient management.

If “cancelation of participation in the trial” occurs, an examination at the time of cancelation is conducted within 3 days after the cancelation, and a follow-up survey is conducted after cancelation of participation in the trial at or around the day of the one-month postpartum medical examination.

### Data management

In this trial, the National Center for Child Health and Development Clinical Research Center has managed and collected data using the electronic case report (eCRF). Outliers and abnormal values confirmed by data cleaning in the data input to eCRF are requested to be confirmed and corrected on eCRF as queries. After fixing the data in the data center in National Center for Child Health and Development Clinical Research Center, the fixed data are provided to the person in charge of analysis. The details are stipulated in the data management plan for this trial.

### Planned statistical analysis

The analysis target population is as follows:

1. Full Analysis Set (FAS)

Subject population (mother, fetus/infant) excluding those who fall under the following from all randomized subjects

- Subjects who have never received protocol treatment
- Subjects who received protocol treatment at least once, but data were not obtained after the treatment
- Subjects who do not meet the eligibility criteria
  2. Per Protocol Set (PPS)

A group of subjects (mother, fetus/infant) excluding subjects showing deviations associated with protocol treatment and related to prohibited drugs from FAS.

3. Safety analysis target population

All subject populations (mother, fetus/infant) who received at least one protocol treatment.

In analyses using FAS, all subjects with missing primary outcome will be treated as having "moderate or greater BPD_36_ or occurrence of death in child up to 36 weeks PMA" for any reason. In analyses using the PPS, subjects with missing primary endpoints will be excluded from the analysis.

The detailed methods are shown in **Table 3**. Briefly, we will analyze data (background information, primary outcome, secondary outcome) using mainly FAS, followed by PPS. The analysis target group of the primary outcome is FAS. We will compare intervention and control groups with the following statistics if appropriate: *t*-test, Mann-Whitney test, Fisher’s exact test, χ^2^ test, Mantel-Haenszel method, Cox proportional hazards model, Kaplan-Meier curve, and stratified Cox proportional hazard model. Regarding safety evaluation items for the mother, fetus, and infant, we will perform coding of events using Medical Dictionary for Regulatory Activities (MedDRA), and will count the numbers according to System Organ Class (SOC) and Preferred Term (PT).

**Table 3:** Planned statistical analysis.

Background information/primary outcome/secondary outcomes		FAS/PPS	Statistics
Background information			
	Age, ethnicity, blood type (ABO, Rh), smoking habit, day of consent, gestational weeks and days at entry into this trial, gravidity, parity, therapy for sterility, body weight (pre-pregnancy, on giving consent), basic complications, any past obstetrical histories, presence/absence of allergy (food, drugs), onset date (gestational weeks and days) of pPROM, findings of pPROM (presence/absence of leak alone, pooling alone, or both leak and pooling)	Mainly FAS, followed by PPS.	Summary statistics (mean, standard deviation, minimum value, median, maximum value)* for continuous variables, and the numbers, frequencies, and 95% CI for discrete variables.
Primary outcome			Intervention group vs. control group
	Incidence rate of either moderate/severe BPD_36_ or IUFD at or less than 36 weeks 0 days	Mainly FAS, followed by PPS.	(1) The difference in crude rate, 95% CI. (2) adjusted risk difference and 95% CI by the Mantel-Haenszel method, adjusted for gestational weeks at onset of pPROM (22 weeks 0 days to 23 weeks 6 days, 24 weeks 0 days to 27 weeks 6 days) and institute (institute where early caffeine will be used, institute where early caffeine will not be used)**.
Secondary outcomes			Intervention group vs. control group
A. Efficacy evaluation items for mother			
	Days from the onset of pPROM to delivery	Mainly FAS, followed by PPS.	(1) Cox proportional hazards model: hazard ratio and 95% CI. (2) Kaplan-Meier curve, (3) by stratified Cox proportional hazards model stratified by stratified factors**.
	Gestational weeks at delivery		(1) Summary statistics**, (2) box-plots, (3) (1) and (2) according to stratified factors**.
	Reasons of pregnancy termination		(1) list 0f reasons for termination of pregnancy, (2) the numbers and frequencies of reasons for pregnancy according to therapy groups, (3) (2) according to stratified factors*.
	Findings at delivery (mode of delivery, presence/absence of stillbirth, placental weight)		A: mode of delivery (1) list of mode of delivery (spontaneous vaginal delivery, induced vaginal delivery, cesarean section, vacuum delivery, breech delivery), (2) the numbers and frequencies of the list according to therapy groups, (3) (2) according to stratified factors**. B: presence/absence of stillbirth (1) the numbers, frequencies, and 95% CI, (2) the difference in frequency and 95% CI, (3) adjusted risk difference and 95% CI by the Mantel-Haenszel method, adjusted for stratified factors**. C: placental weight (1) Summary statistics*, (2) Box-plots, (3) (1) and (2) adjusted for stratified factors**.
	Incidence rate of IUFD		(1) the numbers, frequencies, and 95% CI, (2) the difference in frequency and 95% CI, (3) adjusted risk difference and 95% CI by the Mantel-Haenszel method, adjusted for stratified factors**.
	Incidence rate of histological chorioamnionitis		(1) the numbers, frequencies, and 95% CI, (2) the difference in frequency and 95% CI, (3) adjusted risk difference and 95% CI by the Mantel-Haenszel method, adjusted for stratified factors**.
	Rate of cesarean delivery		(1) the numbers, frequencies, and 95% CI, (2) the difference in frequency and 95% CI, (3) adjusted risk difference and 95% CI by the Mantel-Haenszel method, adjusted for stratified factors**.
	Incidence rate of placental abruption		(1) the numbers, frequencies, and 95% CI, (2) the difference in frequency and 95% CI, (3) adjusted risk difference and 95% CI by the Mantel-Haenszel method, adjusted for stratified factors**.
B. Efficacy evaluation items for infant			
	Incidence rate of moderate or greater BPD_36_	Mainly FAS, followed by PPS.	(1) the numbers, frequencies, and 95% CI, (2) the difference in frequency and 95% CI, (3) adjusted risk difference and 95% CI by the Mantel-Haenszel method, adjusted for stratified factors**.
	Incidence rate of BPD_28_		(1) the numbers, frequencies, and 95% CI, (2) the difference in frequency and 95% CI, (3) adjusted risk difference and 95% CI by the Mantel-Haenszel method, adjusted for stratified factors**.
	Perinatal death rate (total of IUFD + early neonatal death / total of neonatal live infants)		(1) the numbers, frequencies, and 95% CI, (2) the difference in frequency and 95% CI, (3) adjusted risk difference and 95% CI by the Mantel-Haenszel method, adjusted for stratified factors**.
	Rate of infantile deaths until just before discharge of the infants		(1) the numbers, frequencies, and 95% CI, (2) the difference in frequency and 95% CI, (3) adjusted risk difference and 95% CI by the Mantel-Haenszel method, adjusted for stratified factors**.
	Birth weight		(1) Summary statistics*, (2) box-plots, and (3) (1) and (2) according to stratified factors**.
	Birth height		(1) Summary statistics)*, (2) box-plots, and (3) (1) and (2) according to stratified factors**.
	Birth head circumference		(1) Summary statistics*, (2) box-plots, and (3) (1) and (2) according to stratified factors**.
	Day of hospitalization (from the birth date to discharge)		(1) Summary statistics*, (2) box-plots, and (3) (1) and (2) according to stratified factors**.
	Day of invasive ventilation		(1) Summary statistics*, (2) box-plots, and (3) (1) and (2) according to stratified factors**.
	Day of non-invasive ventilation		(1) Summary statistics*, (2) box-plots, and (3) (1) and (2) according to stratified factors**.
	Day of oxygen administration		(1) Summary statistics*, (2) box-plots, and (3) (1) and (2) according to stratified factors**.
	Incidence rate of infants with surfactant administration		(1) the numbers, frequencies, and 95% CI, (2) the difference in frequency and 95% CI, (3) adjusted risk difference and 95% CI by the Mantel-Haenszel method, adjusted for stratified factors**.
	Incidence rate of infants with RDS		(1) the numbers, frequencies, and 95% CI, (2) the difference in frequency and 95% CI, (3) adjusted risk difference and 95% CI by the Mantel-Haenszel method, adjusted for stratified factors**.
	Incidence rate of infants with PPHN		(1) the numbers, frequencies, and 95% CI, (2) the difference in frequency and 95% CI, (3) adjusted risk difference and 95% CI by the Mantel-Haenszel method, adjusted for stratified factors**.
	Incidence rate of infants with IVH		(1) the numbers, frequencies, and 95% CI, (2) the difference in frequency and 95% CI, (3) adjusted risk difference and 95% CI by the Mantel-Haenszel method, adjusted for stratified factors**.
	Incidence rate of infants with PVL		(1) the numbers, frequencies, and 95% CI, (2) the difference in frequency and 95% CI, (3) adjusted risk difference and 95% CI by the Mantel-Haenszel method, adjusted for stratified factors**.
	Incidence rate of infants with sepsis		(1) the numbers, frequencies, and 95% CI, (2) the difference in frequency and 95% CI, (3) adjusted risk difference and 95% CI by the Mantel-Haenszel method, adjusted for stratified factors**.
	Incidence rate of infants with NEC		(1) the numbers, frequencies, and 95% CI, (2) the difference in frequency and 95% CI, (3) adjusted risk difference and 95% CI by the Mantel-Haenszel method, adjusted for stratified factors**.
	Incidence rate of infants with symptomatic PDA		(1) the numbers, frequencies, and 95% CI, (2) the difference in frequency and 95% CI, (3) adjusted risk difference and 95% CI by the Mantel-Haenszel method, adjusted for stratified factors**.
C. Safety evaluation items for mother and fetus			
	Incidence rates of adverse events or side effects	Safety analysis target population	(1) We will perform coding of events using Medical Dictionary for Regulatory Activities (MedDRA), and count the numbers according to System Organ Class (SOC) and Preferred Term (PT). (2) The numbers, frequencies, and 95% CI.
	Incidence rates of serious adverse events or serious side effects		(1) We will perform coding of events using MedDRA, and count the numbers according to SOC and PT. (2) The numbers, frequencies, and 95% CI.
	Incidence rate of critical adverse events (IUFD, sepsis of mother, admission to ICU of mother, multiple organ disorders of mother, artificial ventilation for mother, hysterectomy for mother		(1) We will perform coding of events using MedDRA, and count the numbers according to SOC and PT. (2) The numbers, frequencies, and 95% CI.
D. Safety evaluation items for infants			
	Incidence rates of serious adverse events	Safety analysis target population	(1) We will perform coding of events using MedDRA, and count the numbers according to SOC and PT. (2) The numbers, frequencies, and 95% CI.
E. Other evaluation items in the current trial			
	Vital signs (body temperature, pulse, blood pressure)	Mainly FAS, followed by PPS.	(1) Summary statistics*, at each observation point, (2) box-plots
	Hematological examination, blood biochemical tests		(1) Summary statistics*, at each observation point, (2) box-plots
	Urine protein or urine sugar		(1) Summary statistics*, at each observation point, (2) box-plots
	Detection rate of *Ureaplasma* spp. (in vagina, on surface of the placenta, in infantile pharynx, and in infantile ear cavity		(1) the numbers, frequencies, and 95% CI, for *Ureaplasma* spp. in the vagina, on the surface of the placenta, in the infantile pharynx, and in the infantile ear cavity, respectively, (2) the difference in frequency and 95% CI, (3) adjusted risk difference and 95% CI by the Mantel-Haenszel method, adjusted for stratified factors**.

*, Summary statistics: mean, standard deviation, minimum value, median, maximum value.

**, Stratified factors: gestational weeks of onset of pPROM (22 weeks 0 days to 23 weeks 6 days, 24 weeks 0 days to 27 weeks 6 days) and institute (institute where early caffeine will be used, institute where early caffeine will not be used).

Abbreviations: FAS, full analysis set; PPS, per protocol set; BPD_36_, bronchopulmonary dysplasia (BPD) at week 36 as modified, CI, confidence interval; pPROM, preterm premature rupture of the membranes; IUFD, intrauterine fetal death; BPD_28_, BPD at 28 days of life; RDS, respiratory distress syndrome; PPHN, persistent pulmonary hypertension in the neonate; IVH, intraventricular hemorrhage; PVL, periventricular leukomalacia; NEC, necrotizing enterocolitis; and PDA, patent ductus arteriosus.

If necessary, we will perform exploratory data analysis such as subgroup analysis.

### Confidentiality

In this study, we do not collect personal information. We use anonymization numbers to link the collected data to original source materials. CI in each institute strictly preserves a corresponding table in which anonymization numbers and some personal information for patients recruited into this trial in each institute are written simultaneously. We collect all data using an EDC system built by REDCap^®^, which can be accessed solely by persons with a license for this PPROM-AZM study.

### Access to data

After the data are fixed in this trial, only two persons (Shintaro Iwamoto who performs statistical analysis, and Mieko Makino who is responsible for statistical analysis) can access the fixed data.

### Ancillary and post-trial care

In this trial, we are covered by insurance for clinical research. Therefore, if participants develop some serious adverse events, irrespective of known or unknown associations, costs for treatments for almost all events with clear relationships with antibiotics used in this trial are covered by the insurance.

### Biological specimens

In this study, we obtain swabs from the vagina, surface of the placenta, infant’s throat, and ear cavity. These specimens are stored at -70-80°C, and are used for other research if participants consent to such re-use.

### Timescale

Recruitment started on April 1, 2022, and will be finished on March 31, 2024.

### Trial status

The first patient was included on September 9, 2022.

### Supplementary information for the protocol

We stated the following information in the **S3 Appendix:**

- **Recruitment**
- **Consent to participate**
- **Presence/absence of BPD**_**36**_; **The procedure of supplement oxygen reduction test (SORT) with S1–S3 Figs.**

- **Definition of “discontinuation of protocol treatment”, “cancelation of participation in the trial”, and “cancelation of the entire trial”**
- **Declaration of completion of the trial**; **Data monitoring**
- **Serious adverse events reporting and monitoring**
- **Auditing**

## Discussion

### Characteristics of antibiotic regimen for pPROM occurring at 28 weeks of gestation

This phase II, multicenter, nonblinded, randomized controlled trial is focused on collecting data to estimate the sample size for future phase III randomized controlled trial evaluating the effect of treatment with ABPC/SBT plus AZM on the occurrence of moderate/severe BPD or IUFD or neonatal death at or less than 36 weeks 0 days PMA compared with treatment with ABPC/SBT plus EM in pregnant women with pPROM occurring at 22–27 weeks of gestation. In addition, we also focus on collecting information on adverse events by treatments with either ABPC/SBT plus AZM and ABPC/SBT plus EM, because we use these regimens for a maximum of 14 days; such long-term treatment with AZM has not been recommended on the package insert of AZM; such long-term treatment with EM has also not been recommended in guidelines for the standard regimen using ABPC/AMPC plus EM [12–14].

### Rationale for ABPC/SBT plus AZM and ABPC/SBT plus EM for maximum of 14 days

*Ureaplasma* spp. are often cultured in amniotic fluid in women with pPROM at <28 weeks of gestation [20]. The detection rate of microorganisms was 91%, and that of *Ureaplasma* spp. was 55%, in 22 women with pPROM occurring at <28 weeks of gestation [20]. Macrolide antibiotics are effective for *Ureaplasma* infection; however, EM may be ineffective for the prevention of intra-amniotic inflammation, amniotic fluid culture for microorganisms, and microbial invasion of the amniotic cavity in women with pPROM occurring at 18–32 weeks of gestation [23]. Since AZM is effectively transferred to placental tissue and amniotic fluid [19], it may be more effective for the control of intra-amniotic inflammation, amniotic fluid culture for microorganisms, and microbial invasion of the amniotic cavity, especially in women with pPROM with intrauterine *Ureaplasma* infection.

In Saga Hospital, one of the authors, MN, has performed amniocentesis for women with pPROM occurring at <28 weeks. He has cultured microorganisms including *Ureaplasma* spp. as well as detected *Ureaplasma* spp. using PCR. In addition, as a pilot study, he compared the intra-amniotic levels of interleukin (IL)-6 before and after the administration of AZM in women with pPROM occurring at <28 weeks (under submission). Intra-amniotic levels of IL-6 two days after the administration of AZM were almost one-tenth of those before the administration of AZM. However, the disappearance rate of *Ureaplasma* spp. 7 days after the administration of AZM was under 50%. Interestingly, MN encountered two cases of pPROM in whom *Ureaplasma* spp. were detected in the amniotic fluid, and in whom AZM was repeatedly administered twice at 1–3 days and 8–10 days. In one case, *Ureaplasma* spp. disappeared 15 days after the administration of AZM. In the other case, although *Ureaplasma* spp. did not disappear, the intra-amniotic levels of IL-6 became within normal ranges. Moreover, these 2 patients did not develop BPD. Based on these data and experience of long-term administration of AZM, we hypothesized that a maximum of 14 days administration of EM or AZM may be necessary to inhibit intrauterine inflammation due to *Ureaplasma* spp. as well as prevent the occurrence of BPD.

Accordingly, we planned to use both broad-spectrum penicillin antibiotics plus a macrolide antibiotic, AZM. We also planned to use ABPC/SBT for 14 days instead of the standard regimen of ABPC/AMPC for 7 days, because we expect that the long-term administration of broad-spectrum penicillin antibiotics can increase the effect of antibiotics on the elimination of intrauterine infection, finally leading to a decreased occurrence of BPD. Thus, we planned new antibiotic combination therapy (ABPC/SBT + AZM for 14 days), while the combination of ABPC/SBT plus EM for 14 days is set as the control group. We hypothesized that the combination of ABPC/SBT plus AZM for 14 days may be more effective for the prevention of BPD than ABPC/SBT plus EM for 14 days.

### Rationale for the target sample size in this phase II trial

We set the target sample size as 100 women with singleton pregnancy complicated with pPROM occurring at 22–27 weeks of gestation. Because we expect almost 20 tertiary centers to be transferred 3–7 of such women every year, and that almost half of the candidates will be accepted in this trial, we set the target sample size as 100 in view of the feasibility during the two-year recruitment period.

We created Scoping review sheets to investigate previous RCT and observational studies on pPROM using any antibiotics described in PubMed published until December 2020. Finally, 21 RCTs and 30 cohort studies were selected. However, there were no studies in which antibiotics were administrated only to women with pPROM which occurred at <28 weeks of gestation. In addition, there were no studies in which AZM was administered in women with pPROM. Although we had a case series of women with pPROM which occurred at <28 weeks of gestation [8, 20], we finally concluded that there were no reliable articles by which we could estimate the sample size for a phase III RCT, in which we will examine the effectiveness of AZM and EM under the co-administration of ABPC/SBT for the prevention of BPD in infants delivered by pregnant women with pPROM which occurs at < 28 weeks of gestation. Then, we planned this phase II trial to finally calculate the sample size requirements for a full-scale clinical trial in the future.

### Perspective

The PPROM-AZM trial will generate pilot data on the effectiveness and safety of AZM and EM under the co-administration of ABPC/SBT to prevent moderate/severe BPD_36_ in infants who are delivered by pregnant women with pPROM occurring at <28 weeks of gestation.

## Supporting information

S1 Checklist SPIRIT checklist. (DOCX)

S1 Appendix A PDF of the latest version of the protocol, written by Japanese. (PDF)

S2 Appendix A PDF of the translation to Englich of the latest version of the protocol. (PDF)

S3 Appendix Supporting information for the protocol. (DOCX)

S1 Fig SORT: Baseline evaluation period. (TIF)

S2 Fig SORT: Oxygen/pressure reduction period. (TIF)

S3 Fig SORT: Stability observation period. (TIF)
