# Supplementary material for: A phase II, multicenter, nonblinded, randomized controlled trial for evaluating protective effects of ABPC/SBT plus, azithromycin versus erythromycin, in pregnant women with pPROM occurring at <28 weeks of gestation on the development of BPD in neonates: Study protocol
Source: PLoS One. 2024 Jul 9;19(7):e0304705. doi: 10.1371/journal.pone.0304705 (PMC11232965; doi:10.1371/journal.pone.0304705)
Supplement: S1 Appendix — (PDF) [file pone.0304705.s002.pdf]

妊娠 28 週未満発症の早産期前期破水妊婦から出生した児の
気管支肺異形成発症予防薬として、アジスロマイシンと
エリスロマイシンの有効性と安全性を検討する
第Ⅱ相多施設共同非盲検ランダム化並行群間比較試験

〇代表機関
医療機関名：自治医科大学附属病院
研究代表医師：総合周産期母子医療センター母体・胎児集中治療管理部　大口昭英

版数：2.9 版

作成日：2023 年 11 月 29 日

目次

1　研究概要 5
1.1　概要 5
1.2　概略図 8
1.3　研究スケジュール 9
2　背景 12
2.1　背景 12
2.1.1　妊娠 28 週未満発症の早産前期破水と気管支肺異形成 12
2.1.2　国内外の pPROM に対する抗菌薬治療の標準治療とその問題点 13
2.1.3　ABPC/SBT＋AZM に期待される効果 14
2.2　研究の意義 15
3　目的及び評価項目 16
4　研究デザイン 18
4.1　研究デザイン 18
4.2　ランダム化 19
4.3　科学的合理性の根拠 19
5　対象集団 20
5.1　適格性基準 20
5.1.1　選択基準 20
5.1.2　除外基準 20
5.1.3　設定根拠 21
5.2　目標症例数 21
5.2.1　目標症例数 21
5.2.2　症例数の設定根拠 22
6　介入 23
6.1　使用する医薬品 23
6.1.1　医薬品の概要 23
6.1.2　予測される副作用 26
6.2　プロトコル治療 26
6.2.1　用法・用量 26
6.2.2　プロトコル治療の設定根拠 27
6.2.3　用量変更 28
6.2.4　中断または休薬 28
6.2.5　プロトコル治療期間 28
6.2.6　プロトコル治療の中止基準 28
6.2.7　包装・表示 29
6.2.8　事前準備・事後処理 29
6.3　管理方法 29
6.3.1　保管・交付 29
6.3.2　廃棄・返却 30

6.4 併用薬 30
6.4.1 併用薬 30
6.4.2 併用制限薬・併用制限治療 30
6.4.3 プロトコル治療終了後の感染増悪時の抗菌薬治療 31
6.4.4 併用禁止治療 31
7 研究方法及び手順 31
7.1 被験者リクルート 31
7.2 同意取得 32
7.3 スクリーニング 32
7.3.1 実施項目 32
7.3.2 スクリーニング脱落 32
7.4 被験者登録 32
7.5 観察項目及び収集する情報・手順 32
7.5.1 被験者（母体、胎児）の観察・調査項目及び実施時期 36
7.5.2 被験者（母体、胎児）における検査項目及び実施時期 37
7.5.3 児の観察・調査項目及び実施時期 40
7.5.4 児における検査項目及び実施時期 44
7.5.5 中央測定検体の取扱い 45
7.6 実施期間及び登録期間 45
7.7 登録期間及び観察期間の終了 45
8 同意取得方法 46
8.1 インフォームド・コンセント 46
8.2 18 歳以上の成人した妊婦インフォームド・アセント 46
8.3 同意撤回 46
9 有害事象及び疾病等 47
9.1 定義 47
9.1.1 有害事象の定義 47
9.1.2 重大な有害事象の定義 47
9.1.3 重篤な有害事象の定義 47
9.1.4 疾病等の定義 47
9.2 研究との因果関係 48
9.3 重症度 48
9.4 予測性 48
9.5 収集期間及び追跡期間 48
9.6 有害事象の報告 49
9.6.1 重篤な有害事象の報告 49
9.6.2 疾病等の報告 49
10 中止と終了 50
10.1 被験者の参加中止 50
10.2 研究の中止 51
10.3 研究終了 51

11 予測される利益・不利益及びリスクを最小化する方法 51
11.1 予測される利益 51
11.2 予測される不利益 52
11.3 リスクを最小化する方法 52
12 倫理的事項及び要配慮事項 52
12.1 法令・指針の遵守 52
12.2 認定臨床研究審査委員会 52
12.3 個人情報等の取り扱い 53
12.3.1 匿名化について 53
12.3.2 個人情報に関する被験者等の権利 53
12.4 健康被害に対する補償 53
12.5 研究に参加しない場合の治療方法 53
12.6 研究終了後の医療の提供 54
12.7 遺伝的特徴等に関する研究結果の取り扱い 54
12.8 被験者の経済的負担又は負担軽減費 54
13 研究資金・利益相反及び情報の公開 54
13.1 研究の資金源 54
13.2 利益相反の状況 54
13.3 情報公開の方法 55
13.4 結果の公表 55
14 統計学的事項 56
14.1 解析対象集団 56
14.2 統計解析 56
14.2.1 被験者背景 56
14.2.2 主要評価項目の解析 56
14.2.3 副次評価項目の解析 57
14.2.4 安全性評価項目の解析 59
14.2.5 部分集団解析 60
14.2.6 中間解析計画 60
14.3 統計解析計画の変更 60
15 試料・情報の保管及び廃棄 60
15.1 保管方法・保管期間 60
15.1.1 試料の保存方法・保存期間 60
15.1.2 情報の保管方法・保管期間 60
15.2 廃棄方法 61
15.2.1 試料の廃棄方法 61
15.2.2 情報の廃棄方法 61
15.3 試料・情報の新たな研究での利用 61
16 品質管理及び品質保証 61
16.1 原資料 61
16.2 データマネジメント 62

16.3　モニタリング 62
16.4　監査 62
16.5　効果安全性評価委員会 62
16.6　BPD 診断評価委員会 62
17　法令に基づく報告及び情報共有等 63
17.1　報告先及び情報共有の範囲 63
17.2　実施計画の作成・変更等 63
17.3　定期報告 64
17.4　不適合報告 64
17.5　疾病等報告 64
17.6　研究の中止に関する報告 65
17.7　研究終了及び報告書の作成に関する報告 65
17.8　その他 65
18　研究体制 66
18.1　研究組織 66
18.2　相談窓口 67
18.3　業務委託 67
19　その他 67
19.1　略号一覧 67
19.2　改訂履歴 68
20　引用文献 70

1　研究概要

1.1　概要

**研究課題名**	妊娠 28 週未満発症の早産期前期破水妊婦から出生した児の気管支肺異形成発症予防薬として、アジスロマイシン（AZM）とエリスロマイシン（EM）の有効性と安全性を検討する第Ⅱ相多施設共同非盲検ランダム化並行群間試験
**研究の主旨**	妊娠 28 週未満発症の早産期前期破水（pPROM）妊婦から出生した児の気管支肺異形成（BPD）発症予防薬として、アンピシリンナトリウム/スルバクタムナトリウム（ABPC/SBT）併用下で、AZM と EM の有効性と安全性を探索的に比較検討すること<br>腟・胎盤・新生児咽頭・耳腔でのウレアプラズマ属有無と BPD_{36} 発症との関連を調査すること
**目的**	主目的：妊娠 28 週未満発症の早産期前期破水（pPROM）妊婦から出生した児の気管支肺異形成（BPD）発症予防薬として、アンピシリンナトリウム/スルバクタムナトリウム（ABPC/SBT）併用下で、AZM と EM の有効性と安全性を探索的に比較検討すること
	副次目的：腟・胎盤・新生児咽頭・耳腔でのウレアプラズマ属有無と BPD_{36} 発症との関連を調査すること
**評価項目**	主要評価項目：<br>中等症以上の BPD_{36} または修正 36 週時までの児死亡の発生割合
	副次評価項目（母体の有効性評価項目）：<br>pPROM 発症 から分娩までの日数、分娩時の妊娠週数、妊娠終結理由、分娩所見（分娩様式、死産の有無、胎盤重量）、子宮内胎児死亡割合、組織学的絨毛膜羊膜炎割合、帝王切開割合、常位胎盤早期剝離割合<br><br>副次評価項目（児の有効性評価項目）：<br>BPD_{28} 発生割合、周産期死亡（子宮内胎児死亡＋早期新生児死亡）割合、退院前死亡割合、出生時体重、出生時身長、出生時頭囲、入院日数（出生日～退院日）、侵襲的人工換気日数、非侵襲的人工換気日数、酸素投与された日数、サーファクタント投与を要した児の割合、新生児呼吸窮迫症候群（RDS）発生割合、遷延性肺高血圧症発生割合、頭蓋内出血発生割合、脳室周囲白質軟化症（PVL）発生割合、敗血症発生割合、壊死性腸炎発生割合、症候性動脈管開存症発生割合<br><br>副次評価項目（母体・胎児の安全性評価項目）：<br>有害事象及び副作用の発現状況、重篤な有害事象及び副作用の発現状況、重大な有害事象（胎児死亡、敗血症、intensive care unit (ICU)入室、多臓器不全、人工換気、子宮全摘術） の発現状況

	副次評価項目（児の安全性評価項目）<br>重篤な有害事象及び副作用の発現状況<br><br>副次評価項目（その他の評価項目）:<br>ウレアプラズマ属感染割合（膣、胎盤　児咽頭、児耳腔）
**研究デザイン**	妊娠 28 週未満発症の pPROM 妊婦から出生した児の BPD 発症予防薬として、ABPC/SBT 併用下で、AZM と EM の有効性と安全性を探索的に比較検討するために、第 II 相多施設共同非盲検ランダム化並行群間比較試験を行う。なお、BPD 診断は、試験参加施設ではない施設から当該研究に直接関与しない医学専門家 3 名を選出し、BPD 診断評価委員会を設置し評価者盲検（PROBE 法）で実施する。
**対象**	選択基準:<br>（1）研究参加に関して、本人から文書による同意が得られた妊婦<br>（2）同意取得時の年齢が 18 歳以上の成人した妊婦<br>（3）妊娠 22 週 0 日から妊娠 27 週 6 日で早産期前期破水（pPROM）を発症した単胎妊娠の妊婦<br>除外基準:<br>（1）前置胎盤を合併する妊婦<br>（2）腟式広汎子宮全摘術に伴う子宮頸部切除術既往のある妊婦<br>ただし、高頻度に pPROM が多い子宮頸部円錐切除術既往のある妊婦は除く。<br>（3）pPROM 発症前に妊娠高血圧症候群（高血圧合併妊娠を除く）を合併する妊婦<br>（4）pPROM 発症前に absent endodiastolic velocity (AEDV)あるいは reverse endodiastolic velocity (REDV)を認める子宮内胎児発育不全を合併する妊婦<br>（5）妊娠末期早期での早期娩出を必要とする担癌妊婦<br>（6）pPROM 発症時に早急な分娩が必要と判断された妊婦<br>（7）pPROM 以外で早急な分娩が必要となる疾患及び症状を有する妊婦<br>（8）pPROM 発症前 2 週間以内に、クラミジア感染症と診断され、AZM が投与された妊婦<br>（9）pPROM 前 2 週間以内にペニシリン系あるいはセフェム系以外の抗菌薬を投与されていた妊婦<br>（10）　pPROM 発症後同意取得までに ABPC あるいは ABPC/SBT 以外の抗菌薬を投与されていた妊婦<br>ただし、ABPC あるいは ABPC/SBT 以外の抗菌薬(マクロライド系抗菌薬を除く)投与が同意取得前 1 日前までで、かつ投与期間が 3 日以内である場合は除く。<br>（11）　pPROM 発症後同意取得までにマクロライド系抗菌薬を投与されていた妊婦<br>（12）　ペニシリン系抗菌薬、マクロライド系抗菌薬に対するアレルギーを有する妊婦

	（13）　伝染性単核症を合併する妊婦<br>（14）　染色体異常を有する胎児又は重篤な奇形症候群の疑いがある胎児を妊娠している妊婦<br>（15）　スクリーニング検査で Cr 1.1 mg/dL 以上を示した妊婦<br>（16）　QT 延長症候群を合併する妊婦<br>（17）　精神・中枢神経系疾患を合併し、本研究への参加が困難と研究責任医師が判断する妊婦<br>（18）　その他、研究責任医師が研究への組み入れを不適切と判断した妊婦
**介入**	＜被験群＞<br>（1）スルバシリン静注用 1.5g<br>スルバシリン静注用 1.5gを 1 回 1.5g、1 日 4 回　計 14 日間（Day1～Day14）点滴静注投与する。<br>（2）ジスロマック点滴静注用 500 mg<br>ジスロマック点滴静注用 500 mg を1回 500 mg、1 日 1 回　計 4 日間（Day1～Day2、Day8～Day9）点滴静注投与する。<br>（3）ジスロマック錠 250 mg<br>ジスロマック錠 250 mg を 1 回 1 錠、1 日 1 回（朝食後）　計 10 日間（Day3～Day7、Day10～Day14）服用する。<br>＜対照群＞<br>（1）スルバシリン静注用 1.5g<br>スルバシリン静注用 1.5gを 1 回 1.5g、1 日 4 回　計 14 日間（Day1～Day14）点滴静注投与する。<br>（2）エリスロシン点滴静注用<br>エリスロシン点滴静注用 500mg を1回 500 mg、1 日 2 回　計 4 日間（Day1～Day2、Day8～Day9）点滴静注投与する。<br>（3）エリスロシン錠 200mg<br>エリスロシン錠 200mg を 1 回 1 錠、1 日 4 回（朝食後、昼食後、夕食後、就寝前）　計 10 日間（Day3～Day7、Day10～Day14）服用する。
**目標症例数**	合計 100 例（被験群：50 例、対照群：50 例）
**研究期間**	予定実施期間：　jRCT 登録日～2025 年 3 月 31 日<br>予定登録期間：　2022 年 4 月 1 日～2024 年 3 月 31 日<br>予定観察期間：　2022 年 4 月 1 日～2024 年 8 月 31 日 jRCT 登録日：未登録
**参加期間**	同意取得から最終来院まで最大 5 か月間
**併用禁止**	＜被験群＞<br>（1）アジスロマイシン以外のマクロライド製剤<br>＜対照群＞<br>（1）エリスロマイシン以外のマクロライド製剤<br>（2）エルゴタミン（エルゴタミン酒石酸塩、ジヒドロエルゴタミンメシル酸塩）含有製剤　［クリアミン®、ジヒデルゴット®等］<br>（3）ピモジド［オーラップ®］<br>（4）アスナプレビル［スンベプラ®］
**研究組織**	○研究代表医師・代表機関

	自治医科大学附属病院　総合周産期母子医療センター母体・胎児集中治療管理部　教授　大口昭英<br>〇研究事務局<br>自治医科大学附属病院 産科婦人科学講座　非常勤講師　高橋佳代

1.2　概略図

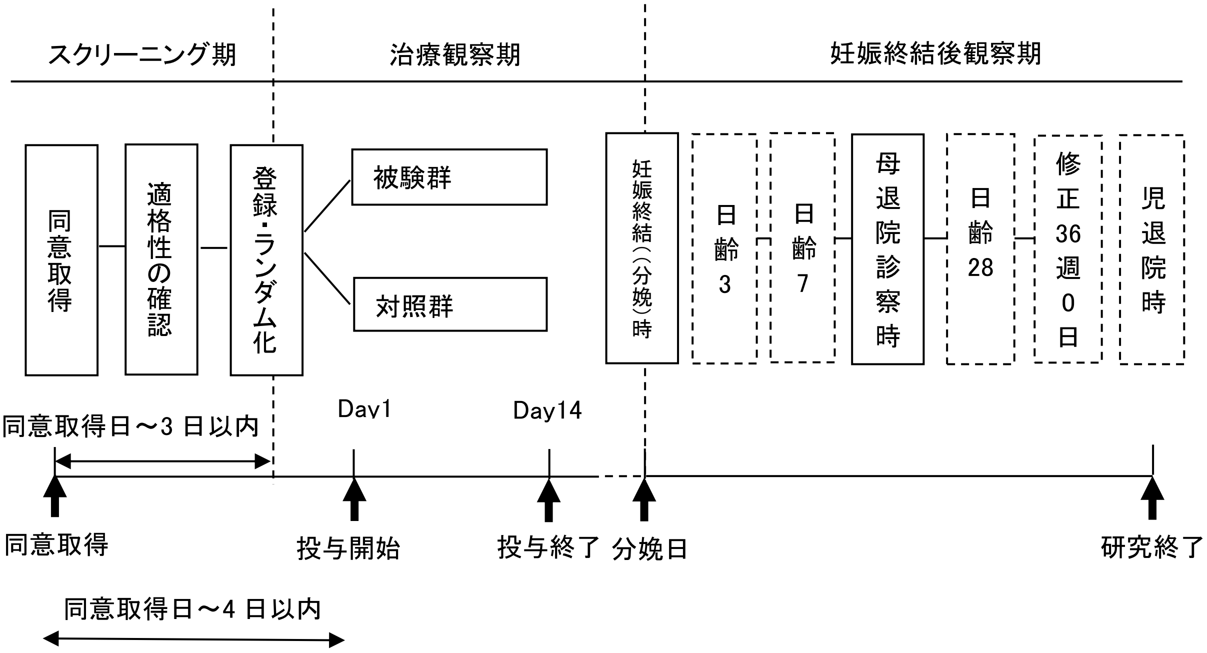

スクリーニング検査までに文書による同意を取得する。
スクリーニング期（同意取得から登録・ランダム化まで）は最大 3 日とする。
同意取得から 4 日以内にプロトコル治療を開始する。
既定の観察、調査、検査は、試験薬の投与前に行う。プロトコル治療を完遂する前に妊娠終結の判断をした場合は、その時点でプロトコル治療を中止して、分娩後に妊娠終結後観察期に移行する。

1.3 研究スケジュール

以下の表に従って、研究を実施する。

＜表 1.3-1 被験者（母体、胎児）のスケジュール＞

期間	SC 期[a]		治療観察期[b]								妊娠終結後観察期			
実施項目	スクリーニング期 Day -3-0	登録 Day0	Week 1 Day 1	Week 2 Day 8 ±2day	Week 3 Day 15 ± 2 day	Week 4 Day 22 ± 2 day	Week 5 Day 29 ± 2 day	Week 6 Day 36 ± 2 day	Week 7 Day 43 ± 2day	Week 8～[c] Day 50 ± 2 day	妊娠終結時 ± 1 day	退院診察時 ± 2 day	研究参加中止時[d]	研究参加中止後フォローアップ[e]
同意取得[f]	X													
被験者背景情報・体重	X[o]													
登録・ランダム化		X												
入院[g]			X	X	X	X	X	X	X	X	X	X		
プロトコル治療[h]			X	X										
身体所見[i]	X		X	X	X	X	X	X	X	X	X	X	X	
バイタル（血圧・脈拍・体温）[i]	X		X	X	X	X	X	X	X	X	X	X	X	
血液学的検査[i]	X[p]			X	X	X	X	X	X	X	X	X	X	
血液生化学検査[i]	X[p]			X	X	X	X	X	X	X	X	X	X	
尿検査（定性：蛋白・尿糖）[i]	X[p]			X	X	X	X	X	X	X	X	X	X	
心電図	X[p]													
膣内細菌培養（施設）[i]			X[r]	X[s]	X	X	X	X	X	X	X			
膣内ウレアプラズマ培養[j]			X[r]	X[s]										
膣内ウレアプラズマ PCR[j]			X[r]	X[s]										
胎盤ウレアプラズマ培養[j]											X			
胎盤ウレアプラズマ PCR[j]											X			
胎盤病理組織検査											X			
胎児推定体重[i]	X[q]			X	X	X	X	X	X	X				
胎児超音波検査（ドップラー・羊水量）[i]	X[q]			X	X	X	X	X	X	X				
併用薬の確認[k]	X		X	X	X	X	X	X	X	X	X	X	X	X
有害事象の確認[l]			X	X	X	X	X	X	X	X	X	X	X	X
妊娠終結に関する調査[m]											X			
分娩所見[n]											X			

a. スクリーニング期（同意取得から登録・ランダム化まで）は最大 3 日間とする。

b. 特に記載のない限り、規定の観察、調査、検査は試験薬の投与前に行う。プロトコル治療を完遂する前に妊娠終結の判断をした場合には、その時点でプロトコル治療を中止し、妊娠終結後に妊娠終結後観察期へ移行する。

c. Week 8 以降は、Week 8 で規定された検査項目を妊娠終結の判断をした日まで、週 1 回行う。

d. 研究参加中止後 3 日以内に行う。

e. 母体の 1 か月健診時を目安に行う。
f. スクリーニング検査開始までに文書による同意取得を行う。
g. 少なくともプロトコル治療開始日から妊娠終結 3 日後までの期間は入院管理を行う。
h. 同意取得後 4 日以内にプロトコル治療を開始する。
i. Week3 以降は、妊娠終結の判断がされる時点まで、週 1 回実施する。ただし、血液学的検査及び血液生化学検査については、Week 4 以降は妊娠終結の判断がされる時点まで必要時とする。
j. 可能な場合に実施する。被験者（母体）に新型コロナウイルス感染症が判明した場合は、検体を採取せず、採取ずみの検体は廃棄する。
k. 有害事象の発現により研究参加を中止した被験者に関しては、研究参加中止の原因となった有害事象に対して使用した併用薬及び併用療法の調査を中止後も継続し、研究参加中止後フォローアップまで調査する。
l. 被験者（母体・胎児）の有害事象を調査する。母体については、プロトコル治療開始日から妊娠終結後観察期（又は研究参加中止時）まで調査する。胎児については、プロトコル治療開始日から妊娠終結日（又は研究参加中止日）まで調査する。ただし、有害事象の発現により研究参加を中止した被験者被験者（母体・胎児）に関しては、研究参加中止の原因となった有害事象については調査を継続し、研究参加中止後フォローアップまで調査する。
m. 妊娠終結判断日、胎児を娩出した時刻、妊娠終結判断理由（母体要件、胎児要件）、妊娠終結日を調査する。
n. 分娩様式、死産の有無及び死亡を確認した日、胎盤重量、胎盤梗塞の有無を調査する。
o. スクリーニング期体重(Kg)は、同意取得前 14 日以内に測定され適切に記録されている場合は、スクリーニング期のデータとして使用してよい。
p. 血液学的検査、血液生化学検査、尿検査（定性：蛋白・尿糖）、心電図は、同意取得前 3 日以内に行われ適切に記録されている場合は、スクリーニング期のデータとして使用してよい。
q. 胎児推定体重、胎児超音波検査（ドップラー・羊水量）は、同意取得前 7 日以内に行われ適切に記録されている場合は、スクリーニング期のデータとして使用してよい。
r. 原則、プロトコル治療開始前に行うが、同意取得後スクリーニング期に行ってもよい。
s. Day8 の腟内ウレアプラズマ培養及び腟内ウレアプラズマ PCR のための検体採取は Day6～Day10 に行う。ただし、Day8 までにプロトコル治療を中止した場合は、これらの検査は行わない。

＜表 1.3-2 児のスケジュール＞

実施項目	妊娠終結時（出生時）	日齢 3（±1 day）	日齢 7（±3 day）	日齢 28[†]（±2 day）	修正 36 週 0 日[#]（±2 day）	児の退院時（±7 day）	母体研究参加中止時 [a]
臍帯動脈血ガス検査	X						
新生児所見 [b]	X						
アプガースコア [c]	X						
血液ガス検査 [d]	X	X [k]					
血液学的検査 [d]	X [i]	X [k]					
血液生化学的検査 [d]	X [i、j]	X [k]					
咽頭ウレアプラズマ培養 [e]	X						
咽頭ウレアプラズマ PCR [e]	X						
耳腔ウレアプラズマ培養 [e]	X						
耳腔ウレアプラズマ PCR [e]	X						
胸部 X 線	X		X [l]	X	X[l]		
入院期間	X	X	X	X	X	X	
呼吸器管理の有無、種類及び期間	X	X	X	X	X	X	
BPD_{36}の有無					X[m]		
BPD_{28}の有無				X			
新生児の合併症 [f]	X	X	X	X	X	X	
治療内容 [g]	X	X	X	X	X	X	
重篤な有害事象 [h]	X	X	X	X	X	X	X

† 日齢 28 までに退院している児の場合は、1 か月健診時に行う。

\# 出生時在胎週数 32 週未満で出生した児の場合は、修正 36 週 0 日にまたは児の退院時のいずれか早いほうの時点で評価する。出生時在胎週数 32 週以上で出生した児の場合は、日齢 56 または退院時のいずれか早いほうの時点で評価する。

a. 研究参加中止後 3 日以内に行う。
b. 新生児の性別、出生時体重（g）、出生時身長（cm）、出生時頭囲（cm）、新生児の発育度を調査する。
c. 出生 1 分後、5 分後に評価する。
d. 動脈血、静脈血、毛細血管血のいずれでも測定可能とする。
e. 被験者（母体）に新型コロナウイルス感染症が判明した場合は、検体を採取しない。
f. 頭蓋内出血、脳室周囲白質軟化症、新生児呼吸窮迫症候群、胎便吸引症候群、新生児一過性多呼吸、遷延性肺高血圧、敗血症、壊死性腸炎、限局性腸穿孔、症候性動脈管開存症、声門下狭窄、喉頭軟化症、上気道疾患（その他）の有無を調査する。
g. 酸素投与、ステロイドの静注・吸入、吸入一酸化窒素、サーファクタント、カフェイン、アジスロマイシン投与について調査する。児の手術について、術式名、手術実施日、手術目的、及び手術目的の挿管の有無を調査する。
h. 児については妊娠終結日から児の退院時（又は母体研究参加中止時）までの情報を調査する。
i. 臍帯血で測定することも可とする。
j. IgM は出生時のみ測定する。

k. 出生体重 1000g 未満の場合、測定しなくてもよい。
l. 必要と判断した場合に行う。
m. 退院時に評価する場合は、退院時（－2 day）に評価する。

2 背景

2.1 背景

2.1.1 妊娠 28 週未満発症の早産前期破水と気管支肺異形成

早産期前期破水 (pPROM)とは、前期破水のなかで妊娠 37 週未満に発症したものである。

本邦では、妊娠 28 週未満の超早産、32 週未満の極早産に占める pPROM の割合は、各々 28.7%、28.1%であったことから、pPROM に伴い超早産となる児は毎年 700 名程度、極早産児となる児は毎年 1,700 名程度と推計される。

pPROM 合併妊婦から出生した児の合併症には、羊水過少による肺低形成、気管支肺異形成、dry lung syndrome、羊膜索症候群、新生児仮死、新生児遷延性肺高血圧、頭蓋内出血、羊水感染による急性炎症性疾患（敗血症、肺炎、髄膜炎）、胎児炎症反応症候群（慢性呼吸障害、壊死性腸炎、脳室周囲白質軟化症(PVL)、神経学的後障害）があり、長期的には脳性麻痺の発生率が高くなる。

気管支肺異形成（BPD）とは、新生児期の呼吸障害が軽快した後、あるいはそれに引き続いて、酸素吸入を必要とするような呼吸窮迫症状が日齢 28 を超えて続くものと定義される [1)]。BPD の重症度は、NICHD により、修正妊娠 36 週での児の酸素使用状況、投与酸素濃度、陽圧換気の有無により、軽症、中等症、重症度に分類される [1)]。BPD は、極低出生体重児の約 50%にみられる発生頻度の高い合併症である。この BPD 合併率は在胎週数が早いほど高率である。最新の NRN データベースによれば、妊娠 22、23、24、25、26、27、28、29、及び 30 週で出生した生存退院児における BPD 発生率は各々76、72、67、58、50、41、32、24、17%であった(周産期母子医療センターネットワークデータベース解析報告：http://plaza.umin.ac.jp/nrndata/)。これらのデータは pPROM 合併妊婦以外の妊婦を含む成績であることから、pPROM 合併妊婦から出生した新生児に限定すれば、BPD 発生率は更に高率と予想される。

BPD を合併すると、乳児期には呼吸不全症状がみられ、長期間、酸素投与や呼吸補助を必要とする。就学期以降は、脳容量低下、認知や言語発達の IQ 低下など、中枢神経系の影響もみられる [2)]。これまでに多くの研究や治療がなされているが、その発生率は 50%前後と現在も高く、極低出生体重児の予後に大きな影響を与えている。

pPROM は BPD の独立危険因子であることが報告されている [3)]。我々が経験した妊娠 22～27 週で pPROM を発症した単胎妊娠妊婦から出生した児 55 名について短期・長期予後を検討したところ、周産期死亡率 13%（子宮内胎児死亡：6%、早期新生児死亡：7%）、肺低形成 14%、新生児呼吸窮迫症候群（RDS）56%、新生児一過性多呼吸（TTN）42%、壊死性腸炎（NEC）6%、脳室内出血（IVH）10%、脳室周囲白質軟化症（PVL）10%、敗血症 4%、生後 28 日での気管支肺異形成症（BPD_{28}）72%、修正妊娠 36 週での BPD （BPD_{36}）60%であった [4)]。

BPD に対する治療は酸素投与等の対症療法しかなく、BPD 合併を予防することが重要である。そのためには、pPROM に対する適切な抗菌薬治療を行い、妊娠継続日数を延長させることが肝要である。

2.1.2　国内外の pPROM に対する抗菌薬治療の標準治療とその問題点

日本産科婦人科学会の産婦人科診療ガイドライン産科編 2020 では、「妊娠 26 週未満であれば、臨床的絨毛膜羊膜炎の有無・推定体重・妊娠週数、施設の低出生体重児対応能力を考慮して小児科医と相談して方針を決める(推奨レベル B)」、「妊娠 26 週以降 34 週未満では、抗菌薬投与下での待機を原則とするが、施設の対応能力によっては、早期の分娩が考慮される(推奨レベル C)」とされているが、推奨される抗菌薬の詳細は示されていない。また、国内で、pPROM の効能・効果で承認されている抗菌薬は存在しない。

アメリカ、イギリス、カナダの学会ガイドラインにおいて、妊娠期間の延長を目的として抗菌薬投与下に待機する基本管理指針は概ね共通しており、抗菌薬としては、アメリカではアンピシリン(ABPC)・アモキシシリン(AMPC)とエリスロマイシン (EM)の併用、イギリスでは EM、カナダではその両者が推奨されている。妊娠 37 週未満の pPROM に対する抗菌薬母体投与についてのランダム化比較試験に対するメタ解析では [5]、抗菌薬投与が、絨毛膜羊膜炎、pPROM 後 48 時間以内及び 7 日以内に出生する新生児数を有意に減少させた。新生児の死亡率に差はみられなかったが、新生児感染症、サーファクタント治療、酸素投与の必要を減少させた。

なお、アジスロマイシン(AZM)については、現時点では標準治療とはなっていないが、アメリカのガイドライン [7]では、EM が使用出来ない場合の代替薬として AZM が第一選択薬として候補にあげられている。

(表 2-1)国内外の pPROM に対する標準治療指針(ガイドラインで推奨されている治療)

国	学会ガイドライン	基本管理指針	推奨される抗菌薬
日本	日本産科婦人科学会産婦人科診療ガイドライン産科編(2020) [6]	・妊娠 26 週未満であれば、小児科医と相談して方針を決める(B)<br>・妊娠 26 週以降 34 週未満では、抗菌薬投与下での待機を原則とする(C)	記載なし
アメリカ	ACOG Practice Bulletins No.217 (2020) [7]	・妊娠 24 週未満であれば、抗菌薬投与が考慮される<br>・妊娠 24 週以降 34 週未満では、抗菌薬投与下での待機が推奨される	ABPC 2g 点滴＋EM 250mg 点滴を 6 時間毎×2 日間<br>→AMPC 500mg 内服＋EM 333mg 内服を 8 時間毎×5 日間
イギリス	RCOG Green-top Guideline No.73 (2019) [8]	・妊娠 24 週以降では、10 日間もしくは陣痛発来までの抗菌薬投が推奨される(Grade A)	EM 250mg を 6 時間毎×10 日間もしくは陣痛発来まで
カナダ	SOGC Reaffirmed Guidelines (2017) [9]	・妊娠 32 週未満では、陣痛発来してなければ抗菌薬投与が考慮される (I-A)	・ABPC 2g 点滴＋EM 250mg 点滴を 6 時間毎×2 日間

		・妊娠 32 週以降では、児の肺成熟が得られてなければ抗菌薬投が推奨される(I-A)	→AMPC 500mg 内服＋EM 333mg 内服を 8 時間毎×5 日間<br>・EM 250mg 内服を 6 時間毎×10 日間

pPROM に対する抗菌薬母体投与が児にサーファクタント治療、酸素投与の必要を減少させることは示されているが、BPD 発症自体を減少させるかは明らかではない。

34 週未満の pPROM に対する抗菌薬母体投与についてのランダム化比較試験に対するメタ解析では、抗菌薬【アンピシリン（ABPC）、アモキシシリン(AMPC)、エリスロマイシン (EM)】により妊娠期間は有意に延長したものの、BPD の発症に差はみられなかった（治療群 vs 対照群：RR 0.90, 95% CI 0.71-1.14）[10]。従って、国外で推奨されている標準的治療法（ABPC/AMPC ＋ EM）では、BPD の発症予防効果は期待できないと考えられる。

2.1.3 ABPC/SBT＋AZM に期待される効果

妊娠 28 週未満発症の pPROM では、高率に子宮内にウレアプラズマ属を認める。妊娠 28 週未満の pPROM 妊婦 22 例における羊水培養陽性は 91%、ウレアプラズマ陽性は 55%と報告されている [11]。ウレアプラズマ属感染に対してはマクロライド系抗菌薬が有効であるが、EM は子宮内への移行率が低いため無効と報告されている [12]。

このように、特に妊娠 28 週未満の pPROM は、既に子宮内感染が発生している確率が高率であると予想されることから、妊娠 28 週以降の pPROM とは異なる抗菌薬管理（一般細菌に効果のあるブロードスペクトラムのペニシリン系抗菌薬あるいはセフェム系抗菌薬とウレアプラズマ属に効果のあるマクロライド系抗菌薬）が検討される。

このため、本試験では、国外で推奨されている標準的治療法（ABPC/AMPC ＋ EM）ではなく、ABPC/AMPC に変えてよりブロードスペクトラムの ABPC/SBT とし、投与期間も 7 日間ではなく 14 日間まで投与可能とした。EM の投与期間も、7 日間ではなく、14 日間とした。これによって、従来法では期待できなかった BPD に対する効果を期待出来る可能性があると考えている。

AZM は、胎盤への移行率が非常に高いことが報告されている [13]。

国内の妊娠 28 週未満発症の pPROM 妊婦から出生した児における BPD 中等症／重度(NICHD が 2001 年に発表した重症度分類)の発症率は、羊水感染・炎症を認めた時点で抗緑膿菌ペニシリン（PIPC） or セフメタゾール（CMZ）+クリンダマイシン（CLDM）投与群（10/11 例）と比較して、アンピシリンナトリウム/スルバクタムナトリウム（ABPC/SBT）＋アジスロマイシン (AZM)群（2/11 例）で有意に低かった [11]。羊水ウレアプラズマ属感染を有する妊婦への AZM 投与を検討したところ、投与前と投与 2 日後の羊水インターロイキン(IL)-6（12 例）の中央値と四分位は各々28.2 ng/mL(10.8-73.7)、2.4 ng/mL(0.5-23.1)、投与前と投与 2 日後のウレアプラズマ属細菌の核酸量（8 例）では各々2.21×10^{8}/mL(3.12×10^{7}-4.96×10^{8})、4.29×10^{6}/mL(3.47×10^{5}-1.48×10^{7})であり、羊水中 IL-6、ウレアプラズマ属細菌量ともに有意な低下を認めている（野見山ら、未発表データ）。とはいえ、7 日以内にウレアプラズマ属が陰性化したのはわずか 40%であった。しかし、投与開始日から 7 日目を超えて 2 回 AZM が使用された 2 例において、1 例は day15 にウレアプラズマが陰性化し、day9 以降に子宮内炎症が軽症化した。これは AZM を反復投与した効果であると思われる。もう 1 例ではウレアプラズマは陰性化しなかったが、day12 に子宮内炎症が正常化した。これはウレアプラズマの根絶にまではいたらないものの AZM によってウレアプラズマが減少し炎症が正常化したためと思われる。このように、症例によっては AZM 投与を投与

開始後 7 日目以降に投与することによって、ウレアプラズマ属の子宮内での増殖を抑制し、結果的に子宮内炎症を正常化あるいは軽症化することで妊娠期間を延長出来ている症例がみられた。また、この AZM 長期投与された 2 例は BPD_{36} を発症しなかった。このような事実から、我々は、妊娠 28 週未満発症の pPROM に対しては、EM あるいは AZM を最大 2 週間まで投与することは、ウレアプラズマによる子宮内炎症を抑制し、ひいては、BPD_{36} 発症予防において意味があると考えている。しかし、この研究 [11)]は患者背景を揃えた無作為化比較試験ではなく、前後比較試験による成績であり、本当にアジスロマイシンが気管支肺異形成の発生を予防可能であるかどうかは不明である。

このように、ABPC/SBT＋EM、ABPC/SBT＋AZM のいずれも従来法よりは BPD に対する効果を期待できるものの、これらの方法を用いた BPD のための試験がこれまで行われてこなかったために、どの程度の治療成績が見込めるかというデータが全く存在していない。そのため、我々は、ABPC/SBT＋AZM を用いた単群試験ではなく、ABPC/SBT＋EM と ABPC/SBT＋AZM との 2 種類の抗菌薬治療を比較する並行群間比較試験を計画し、併せて ABPC/SBT、EM、及び、AZM の安全性を調べるために第 II 相試験を計画した。本研究で得られた両群における BPD 発生率のデータは、今後予定される第 III 相試験のサンプルサイズを計算する上でも必須の情報となる。

安全性については、EM に関しては、自治医科大学での pPROM 症例については、本試験と同様のプロトコルで行っているが、血管痛以外、重篤（あるいは重大）な有害事象は発生していない。また、pPROM に対しては、通常 2 週間までの抗菌薬投与はしばしば行われている。また、学会等では EM の母児に対する重篤(あるいは重大)な有害事象の発生報告は我々の知る範囲ではない。一方、AZM については、これまでの臨床試験での AZM の使用よりも用量、投与日数ともに多くなることから、有害事象の発生率及び使用上の安全性については詳細な調査が必要と考えている。因果関係が見られると判断した場合は、臨床研究保険でしっかりと補償が行われる体制も整備した。なお、試験途中で重大な有害事象が多発するようであれば、本研究全体の中止を検討する。

以上より、ABPC/SBT + AZM 投与は、国外で推奨されている ABPC/AMPC ＋ EM 投与よりも、子宮内感染の予防・治療効果が期待され、ウレアプラズマ属細菌を主体とした羊水炎症・感染を介した胎児肺損傷および新生児肺感染・炎症への移行を防ぐことによる BPD の発症予防が期待される。

2.2 研究の意義

本邦では、アンピシリンナトリウム/スルバクタムナトリウム（ABPC/SBT）、アジスロマイシン(AZM)、エリスロマイシン (EM)すべて、pPROM に対しては適応外使用である。ABPC/SBT 併用下での EM 投与は、海外で推奨されている pPROM に対する抗菌薬治療であるが、本邦ではほとんど行われていない。また、ABPC/SBT 併用下 AZM 投与の先行研究も少ない。

そこで、本研究では、妊娠 28 週未満発症の早産期前期破水（pPROM）妊婦から出生した児の気管支肺異形成（BPD）発症予防薬として、ABPC/SBT 併用下で、AZM と EM の有効性と安全性を探索的に比較検討する。

本研究により、ABPC/SBT 併用下で、AZM 投与が EM 投与よりも、BPD 発症予防効果が高いことが期待されれば、Ⅲ相検証試験を行う。

pPROM 合併妊婦から出生した多くの新生児が中等症／重度の BPD 発症を免れることができれば、短期的には、児の酸素投与日数・人工換気日数の短縮が見込まれ、在院日数の低下やステロイドなどの治療薬減少により医療経済的な貢献も期待される。長期的には、BPD による就学期 IQ 低下を予防でき極低出生体重児の知能予後を改善させることが期待される。また、早期退院は、母子分離期間を短縮させ、母子愛着形成促進効果も期待できる。さらに、妊娠 28 週未満出生の超早産児では、胎盤におけるウレアプラズマの菌量が多いほど、生後 8 年での呼吸機能（%FEF50）が低くなる事が示されており（柳原ら、未発表データ）、AZM が子宮内のウレアプラズマ属の菌量減少に寄与するのであれば、長期的な児の呼吸機能の改善も期待できる。

3　目的及び評価項目

本試験の目的は、以下である。

1） 妊娠 28 週未満発症の早産期前期破水（pPROM）妊婦から出生した児の気管支肺異形成（BPD）発症予防薬として、アンピシリンナトリウム/スルバクタムナトリウム（ABPC/SBT）併用下で、アジスロマイシン(AZM)とエリスロマイシン(EM)の有効性と安全性を探索的に比較検討すること
2） 腟・胎盤・新生児咽頭・耳腔でのウレアプラズマ属有無と BPD_{36} 発症との関連を調査すること

従って、第Ⅱ相多施設共同非盲検ランダム化並行群間比較試験を行う。主要評価項目は、中等症以上の BPD_{36} と修正 36 週時までの児死亡をイベントとし、イベントの発生割合とする。なお、BPD 診断は、試験参加施設ではない施設から当該研究に直接関与しない医学専門家 3 名を選出し、BPD 診断評価委員会を設置し評価者盲検（PROBE 法）で実施する。

ABPC/SBT 併用下での、AZM と EM の有効性と安全性を探索的に評価するため、副次評価項目として、以下を設定する。

副次評価項目（母体の有効性評価項目）

pPROM 発症 から分娩までの日数
分娩時の妊娠週数
妊娠終結理由
分娩所見（分娩様式、死産の有無、胎盤重量）
子宮内胎児死亡割合
組織学的絨毛膜羊膜炎割合
帝王切開割合
常位胎盤早期剥離割合

副次評価項目（児の有効性評価項目）

BPD_{28} 発生割合
周産期死亡（子宮内胎児死亡＋早期新生児死亡）割合
退院前死亡割合

出生時体重、出生時身長、出生時頭囲
入院日数（出生日～退院日）
侵襲的人工換気日数
非侵襲的人工換気日数
酸素投与された日数
サーファクタント投与を要した児の割合
新生児呼吸窮迫症候群（RDS）発生割合
遷延性肺高血圧症発生割合
頭蓋内出血発生割合
脳室周囲白質軟化症（PVL）発生割合
敗血症発生割合
壊死性腸炎（NEC）発生割合
症候性動脈管開存症発生割合

副次評価項目（母体・胎児の安全性評価項目）
有害事象及び副作用の発現状況
重篤な有害事象及び副作用の発現状況
重大な有害事象（胎児死亡、敗血症、intensive care unit (ICU)入室、多臓器不全、人工換気、子宮全摘術）の発現状況

副次評価項目（児の安全性評価項目）
重篤な有害事象及び副作用の発現状況

副次評価項目（その他の評価項目）
ウレアプラズマ属感染割合（膣、胎盤　児咽頭、児耳腔）

4 研究デザイン

4.1 研究デザイン

妊娠 28 週未満発症の早産期前期破水（pPROM）妊婦から出生した児の気管支肺異形成（BPD）発症予防薬として、アンピシリンナトリウム/スルバクタムナトリウム（ABPC/SBT）併用下で、アジスロマイシン(AZM)とエリスロマイシン(EM)の有効性と安全性を比較検討するため、第Ⅱ相多施設共同非盲検ランダム化並行群間比較試験を行う。中等症以上の BPD_{36} と修正 36 週時までの児死亡をイベントとし、イベントの発生割合を比較し、AZM と EM の有効性を評価する。母体と児における有害事象及び副作用の発現状況により安全性を評価する。

本研究は、pPROM 発症後に入院した妊婦を対象に入院期間中に実施される臨床試験であり、スクリーニング期、プロトコル治療開始日から妊娠終結までの治療観察期、妊娠終結後から児の退院日までの妊娠終結後観察期で構成される。

研究責任医師又は研究分担医師は、同意取得後にスクリーニング検査を行い、適格性を評価する。研究責任医師又は研究分担医師は、被験者をプロトコル治療始前までに REDCap で登録する。登録後、適格と判定された被験者は、層別割付けによって、被験群又は対照群に割り付けられる。同意取得からランダム化までのスクリーニング期は最大 3 日間とする。

研究責任医師又は研究分担医師は、同意取得日から 4 日以内にプロトコル治療を開始する。試験薬ジスロマック点滴静注用 500 mg 又はエリスロシン点滴静注用 500mg の投与開始日を Day1 とし、入院管理下で投与を開始する。

プロトコル治療期間は、Day1 から Day14 の 14 日間とするが、14 日間の投与を完遂する前に妊娠終結の判断[*1]をした場合には、その時点でプロトコル治療を中止する。

プロトコル治療が終了してから妊娠終結[*2]までは、入院管理下で、規定された観察、調査、検査を行う。

妊娠終結後は、妊娠終結日に妊娠終結時検査、母の退院診察時に母退院診察時検査、妊娠終結日から 28 日後に妊娠終結日から 28 日後検査、修正 36 週 0 日時点で修正 36 週 0 日時検査、児の退院時に児退院時検査を行い、研究を終了する。なお、妊娠終結後も入院管理または外来管理で被験者（母体、胎児）の安全性を確保する。

研究参加を中止した場合には可能な限り中止後 3 日以内に中止時検査、母の 1 か月健診時を目安に研究参加中止後フォローアップ調査を行う。

[*1] 妊娠終結を判断した日を妊娠終結判断日とする。

[*2] 胎児の娩出が完了した日を妊娠終結日とする。

研究デザインの概略図を図 4.1-1 に示す。

図 4.1-1 研究デザインの概略図

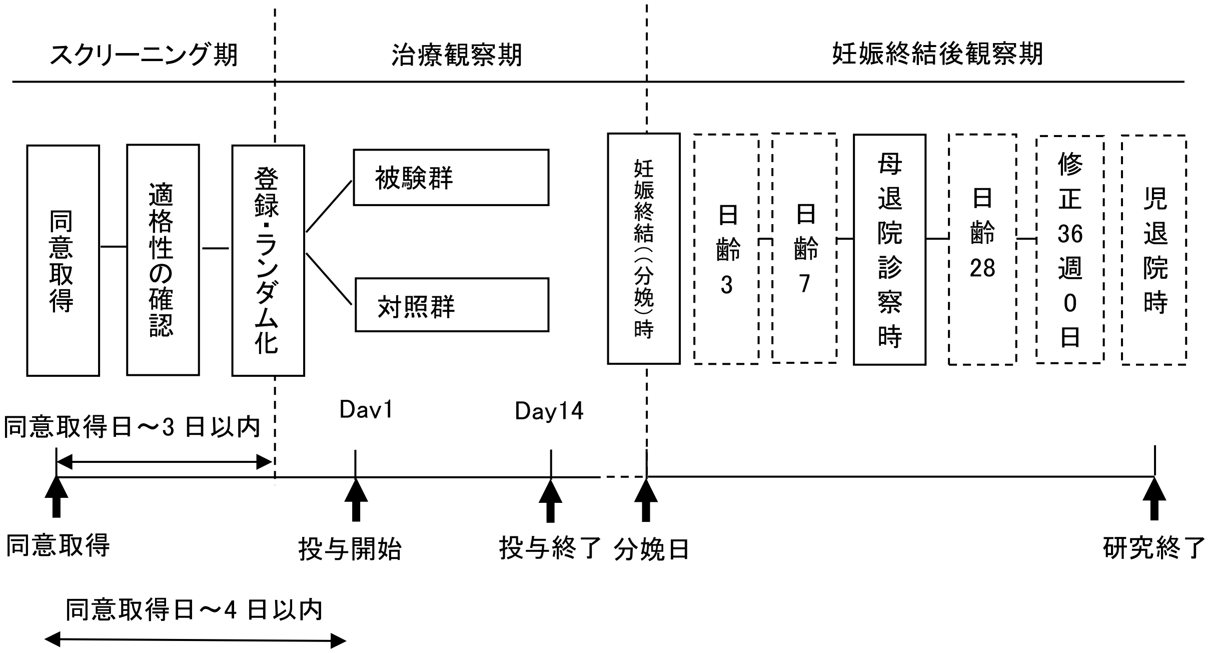

スクリーニング検査までに文書による同意を取得する。
スクリーニング期（同意取得から登録・ランダム化まで）は最大 3 日とする。
同意取得から 4 日以内に試験薬の投与を開始する。既定の観察、調査、検査は、試験薬の投与前に行う。プロトコル治療を完遂する前に妊娠終結の判断をした場合は、その時点でプロトコル治療を中止して、分娩後に妊娠終結後観察期に移行する。

4.2　　ランダム化

割付けは、登録順に REDCap によって行われる。REDCap はすべての組み入れ基準をみたした被験者（母体、胎児）を層別割付けにより、被験群又は対照群に1：1に割り付ける。pPROM 発症時の妊娠週数及び早期カフェイン使用により、BPD 発生割合が異なるため、ランダム化の際には、pPROM 発症時の妊娠週数（22 週 0 日〜23 週 6 日、24 週 0 日〜27 週 6 日）と施設（早期カフェイン使用施設、早期カフェイン未使用施設）を層別因子とする。早期カフェインの使用施設かどうかは、参加前に実施施設に調査する。

4.3　　科学的合理性の根拠

本邦では、アジスロマイシン(AZM)とエリスロマイシン(EM)は、早産期前期破水（pPROM）に対する適応は承認されておらず、臨床試験も行われていない。そこで、本研究では、妊娠 28 週未

満発症の pPROM 妊婦から出生した児の気管支肺異形成（BPD）発症予防薬として、AZM と EM の有効性と安全性を探索的に比較検討するために、第Ⅱ相多施設共同非盲検ランダム化並行群間比較試験を行うこととした。

本研究では、先行研究を参考に、対象集団として、妊娠 22 週 0 日から妊娠 27 週 6 日で pPROM を発症した単胎妊娠の妊婦を設定した。この BPD 発症割合は pPROM 発症妊娠週数が早いほど高率である。よって、pPROM 発症時の妊娠週数をランダム化の層別因子に設定した。

本研究の主要評価項目は、中等症以上の BPD_{36} または修正 36 週時までの児死亡の発生割合とした。BPD は、未熟性、長期間の人工呼吸器管理、高濃度酸素、感染、などを原因として発症する肺組織障害である。主に早産児に発症し、長期にわたり酸素投与や呼吸サポート（気管挿管による人工呼吸器や NCPAP、HFNC など）を必要とする。

近年提唱されている BPD 定義のほとんどが、酸素投与と呼吸サポートの有無を使用している[1,14–17)]。また、現在、修正 36 週での酸素と呼吸サポート使用を用いた重症度分類は、2001 年に NICHD が報告した分類「NICHD,2001」[1)]が、内外を問わず広く使用されている。以上より、本研究では、BPD_{36} 発症の有無は、「修正 36 週時点での酸素または呼吸サポート使用の有無（出生時在胎週数 32 週以上で出生した児の場合は、日齢 28 から 56 または自宅退院のいずれか早いほうの時点での酸素または呼吸サポート使用の有無）」と定義し、BPD 重症度分類は、「NICHD,2001」を使用する [1)]。評価時点で、22%以上 30%未満の酸素を使用している場合は、酸素減量テスト [18–19)]を用いて、BPD の診断を行う。

5　対象集団

5.1　適格性基準

5.1.1　選択基準

本研究に参加するためには、次の基準を全て満たさなければならない。

（1）研究参加に関して、本人から文書による同意が得られた妊婦

（2）同意取得時の年齢が 18 歳以上の成人した妊婦

（3）妊娠 22 週 0 日から妊娠 27 週 6 日で早産期前期破水（pPROM）を発症した単胎妊娠の妊婦

pPROM の診断は、クスコ診で持続的に leak、pooling のいずれか、あるいは両方がみられる場合とする。

破水診断試薬で陽性となっただけの場合は、pPROM とはしない。

5.1.2　除外基準

次の基準に一つでも該当する場合は、本研究に参加することができない。

（1）前置胎盤を合併する妊婦

（2）腟式広汎子宮全摘術に伴う子宮頸部切除術既往のある妊婦、ただし、高頻度に pPROM が多い子宮頸部円錐切除術既往のある妊婦は除く。

（3）pPROM 発症前に妊娠高血圧症候群（高血圧合併妊娠を除く）を合併する妊婦

(4) pPROM 発症前に absent endodiastolic velocity (AEDV)あるいは reverse endodiastolic velocity (REDV)を認める子宮内胎児発育不全を合併する妊婦
(5) 妊娠末期早期での早期娩出を必要とする担癌妊婦
(6) pPROM 発症時に早急な分娩が必要と判断された妊婦
(7) pPROM 以外で早急な分娩が必要となる疾患及び症状を有する妊婦
(8) pPROM 発症前 2 週間以内に、クラミジア感染症と診断され、AZM が投与された妊婦
(9) pPROM 発症前 2 週間以内にペニシリン系あるいはセフェム系以外の抗菌薬を投与されていた妊婦
(10) pPROM 発症後同意取得までに ABPC あるいは ABPC/SBT 以外の抗菌薬を投与されていた妊婦
ただし、ABPC あるいは ABPC/SBT 以外の抗菌薬(マクロライド系抗菌薬を除く)投与が同意取得前 1 日前までで、かつ投与期間が 3 日以内である場合は除く。
(11) pPROM 発症後同意取得までにマクロライド系抗菌薬を投与されていた妊婦
(12) ペニシリン系抗菌薬、マクロライド系抗菌薬に対するアレルギーを有する妊婦
(13) 伝染性単核症を合併する妊婦
(14) 染色体異常を有する胎児又は重篤な奇形症候群の疑いがある胎児を妊娠している妊婦
(15) スクリーニング検査で Cr 1.1 mg/dL 以上を示した妊婦
(16) QT 延長症候群を合併する妊婦
(17) 精神・中枢神経系疾患を合併し、本研究への参加が困難と研究責任医師が判断する妊婦
(18) その他、研究責任医師が研究への組み入れを不適切と判断した妊婦

5.1.3 設定根拠

選択基準
(1) 倫理的な観点から設定した。
(2) 試験薬の評価に適切な年齢を選択するため。
(3) 対象疾患と診断された患者を選択するため。

除外基準
(1)～(11) 試験薬の有効性の観点から設定した。
(12)～(17)被験者(母体、胎児)の安全性の観点から設定した。
(18)本研究の対象として適格でない患者を除外するため設定した。

5.2 目標症例数

5.2.1 目標症例数

目標症例数: 被験群 50 例、対照群 50 例(合計 100 例)

5.2.2　症例数の設定根拠

本研究では、胎児死亡例、新生児死亡例は気管支肺異形成（BPD）発症リスクが高いと考え、修正 36 週時までの児死亡もイベントとしているため、修正 36 週時までの児死亡(5%)も加味し、両群の効果量を設定した。

海外先行研究では、妊娠 28 週未満に pPROM を発症した妊婦 110 例にアジスロマイシン（AZM）を投与した際の BPD_{36} 発症割合は 26.4%（29/110、児は全員生存）であった[20)]。国内先行研究では、妊娠 28 週未満に pPROM を発症した妊婦 11 例にスルバクタムナトリウム（SBT）／アンピシリンナトリウム（ABPC）＋AZM を投与した際の BPD_{36} 発症割合は 18. 2%（2/11、児は全員生存）であった[11)]。また、佐賀病院において、2013 年 6 月～2020 年 5 月までに妊娠 28 週未満に pPROM を発症した妊婦 10 例に SBT/ABPC＋AZM を投与した際の BPD_{36} 発症割合は 40.0%（4/10、児は全員生存）であった（野見山ら、未公表データ）。以上より、被験群（AZM 群）の中等症以上の BPD_{36} または修正 36 週時までの児死亡の発生割合は、18.0～40.0%仮定した。

一方、文献検索からは、妊娠 28 週未満に pPROM を発症した妊婦にエリスロマイシン（EM）投与した報告は検索できなかった。そこで、国外で推奨されている対照群（EM 群）の中等症以上の BPD_{36} または修正 36 週時までの児死亡の発生割合は、一般的な BPD_{36} 発生割合から 60.0%と仮定した。

pPROM 合併妊婦の妊娠継続週数中央値は 2 週間である。年間分娩数、週数別出生数、pPROM の割合から、pPROM 合併妊婦から 30 週未満で出生する新生児は年間 1200 例と推計できる。また本研究に参加予定の地域・総合周産期母子医療センター20 施設では、2 年間で約 200 例と推計される。本研究の適格性基準に合致し、同意を得られる割合を 50%と仮定すると 2 年間で 100 名は登録可能と考え、実施可能性の観点から本研究の目標症例数を 100 例とした。両側検定、有意水準 5%のもとで、被験群において想定される BPD_{36} 発症割合を 18.0%～40.0%としたときの検出力は 0.516～0.995 であり、少なくとも 0.5 以上の検出力が担保されると考えられる。

参考までに被験群の BPD_{36} 発症割合を 18.0%～60.0%としたときの検出力曲線を以下に示す。あわせて、海外・国内先行研究、佐賀病院の研究における発症割合を赤線で示す。

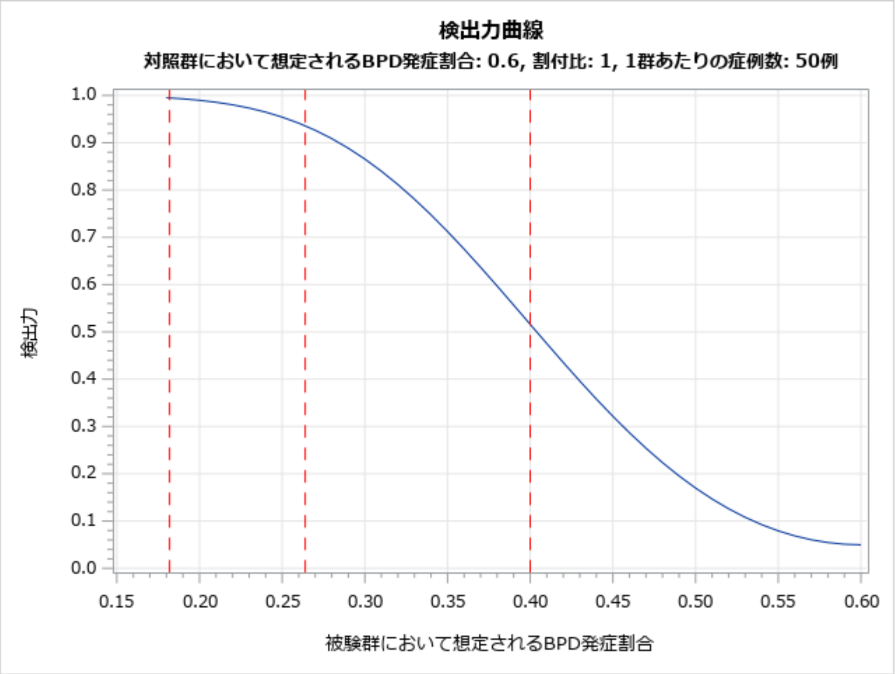

6 介入

6.1 使用する医薬品

6.1.1 医薬品の概要

本研究では、以下の被験薬・対照薬（試験薬）を用いる。

被験薬

一般名	注射用アンピシリンナトリウム・スルバクタムナトリウム
商品名／製造販売業者	スルバシリン静注用 1.5g（Meiji Seika ファルマ株式会社）
剤形・性状・含量	1バイアル中に日局スルバクタムナトリウム 0.5g（力価）、日局アンピシリンナトリウム 1g（力価）を含有する、用時溶解して用いる注射液である。<br>白色～帯黄白色の粉末で、pH：8.0～10.0［1.5g（力価）/10mL、水溶液］。
薬効分類	β-ラクタマーゼ阻害剤配合抗生物質製剤
効能・効果 適応菌種	本剤に感性のブドウ球菌属、肺炎球菌、モラクセラ（ブランハメラ）・カタラーリス、大腸菌、プロテウス属、インフルエンザ菌
効能・効果 適応症	肺炎、肺膿瘍、膀胱炎、腹膜炎
用法・用量	[肺炎、肺膿瘍、腹膜炎]通常成人にはスルバクタムナトリウム・アンピシリンナトリウムとして、1 日 6g（力価）を 2 回に分けて静脈内注

	射又は点滴静注する。なお、重症感染症の場合は必要に応じて適宜増量することができるが、1 回 3g（力価）1 日 4 回（1 日量として 12g（力価））を上限とする。<br>[膀胱炎の場合]通常成人にはスルバクタムナトリウム・アンピシリンナトリウムとして、1 日 3g（力価）を 2 回に分けて静脈内注射又は点滴静注する。
保管条件	室温保存

一般名	点滴静注用アジスロマイシン水和物
商品名／製造販売業者	ジスロマック点滴静注用 500 mg（ファイザー株式会社）
剤形・性状・含量	1 バイアル中に日局アジスロマイシン水和物 524.1 mg （アジスロマイシンとして 500.0 mg（力価））を含有する。<br>白色の塊又は粉末（凍結乾燥品）、pH 6.2～6.8（注射用水 4.8mL にて溶解した濃度 100mg/mL の溶液時）。
薬効分類	15 員環マクロライド系抗生物質製剤
効能・効果 適応菌種	アジスロマイシンに感性のブドウ球菌属、レンサ球菌属、肺炎球菌、 淋菌、モラクセラ（ブランハメラ）・カタラーリス、インフルエンザ菌、 レジオネラ・ニューモフィラ、ペプトストレプトコッカス属、プレ ボテラ属、クラミジア属、マイコプラズマ属
効能・効果 適応症	肺炎、肺膿瘍、膀胱炎、腹膜炎
用法・用量	成人にはアジスロマイシンとして 500 mg（力価）を 1 日 1 回、2 時間かけて点滴静注する。
保管条件	室温保存

一般名	アジスロマイシン水和物錠
商品名／製造販売業者	ジスロマック錠 250 mg（ファイザー株式会社）
剤形・性状・含量	1 錠中　日局 アジスロマイシン水和物 262.0 mg （アジスロマイシンとして 250 mg（力価））を含有する白色 フィルムコート錠
薬効分類	15 員環マクロライド系抗生物質製剤
効能・効果 適応菌種	アジスロマイシンに感性のブドウ球菌属、レンサ球菌属、肺炎球菌、 淋菌、モラクセラ（ブランハメラ）・カタラーリス、インフルエン ザ菌、レジオネラ・ニューモフィラ、ペプトストレプトコッカス 属、プレボテラ属、クラミジア属、マイコプラズマ属
効能・効果 適応症	深在性皮膚感染症、リンパ管・リンパ節炎、咽頭・喉頭炎、扁桃 炎（扁桃周囲炎、扁桃周囲膿瘍を含む）、急性気管支炎、肺炎、肺 膿瘍、慢性呼吸器病変の二次感染、尿道炎、子宮頸管炎、骨盤内 炎症性疾患、副鼻腔炎、歯周組織炎、歯冠周囲炎、顎炎
用法・用量	＜深在性皮膚感染症、リンパ管・リンパ節炎、咽頭・喉頭炎、扁 桃炎（扁桃周囲炎、扁桃周囲膿瘍を含む）、急性気管支炎、肺炎、肺膿瘍、慢性呼吸器病変の二次感染、副鼻腔炎、歯周組織炎、歯 冠周囲炎、顎炎＞

	成人にはアジスロマイシンとして、500 mg（力価）を 1 日 1 回、3 日間合計 1.5 g（力価）を経口投与する。<br>＜尿道炎、子宮頸管炎＞ 成人にはアジスロマイシンとして、1000 mg（力価）を 1 回経口 投与する。<br>＜骨盤内炎症性疾患＞ 成人にはアジスロマイシン注射剤による治療を行った後、アジス ロマイシンとして 250 mg（力価）を 1 日 1 回経口投与する。
保管条件	常温保存

対照薬

一般名	エリスロマイシンラクトビオン酸塩
商品名／製造販売業者	エリスロシン点滴静注用 500mg （マイラン EPD 合同会社）
剤形・性状・含量	1 バイアル中　日局　エリスロマイシンラクトビオン酸塩　500mg（力価）を含有する、白色の粉末（凍結乾燥注射剤）。<br>pH5.0～7.5［50mg（力価）/mL 溶液］
薬効分類	マクロライド系抗生物質製剤
効能・効果 適応菌種	外傷・熱傷及び手術創等の二次感染、肺炎、ジフテリア
効能・効果 適応症	エリスロマイシンに感性のブドウ球菌属、レンサ球菌属、肺炎球菌、ジフテリア菌
用法・用量	通常、成人にはエリスロマイシンとして 1 日 600～1500mg（力価）を 2～3 回に分けて 1 回 2 時間以上かけて点滴静注する。なお、年齢、症状により適宜増減する。
保管条件	室温保存

一般名	エリスロマイシンステアリン酸塩錠
商品名／製造販売業者	エリスロシン錠 200mg（マイラン EPD 合同会社）
剤形・性状・含量	1 錠中に日局エリスロマイシンス テアリン酸塩 200 ㎎（力価）を含有する腸溶性フィルム コーティング錠。
薬効分類	マクロライド系抗生物質製剤
効能・効果 適応菌種	本剤に感性のブドウ球菌属、レンサ球菌属、肺炎球菌、淋菌、髄膜炎菌、ジフテリア菌、赤痢菌、軟性下疳菌、百日咳菌、破傷風菌、ガス壊疽菌群、梅毒トレポネーマ、トラコーマクラミジア （クラミジア・トラコマティス）、マイコプラズマ属、赤痢アメー バ
効能・効果 適応症	表在性皮膚感染症、深在性皮膚感染症、リンパ管・リンパ節炎、慢性膿皮症、外傷・熱傷及び手術創等の二次感染、乳腺炎、骨髄炎、咽頭・喉頭炎、扁桃炎（扁桃周囲炎を含む）、急性気管支炎、肺炎、肺膿瘍、膿胸、慢性呼吸器病変の二次感染、膀胱炎、腎盂腎炎、尿道炎、淋菌感染症、軟性下疳、梅毒、性病性（鼠径）リンパ肉芽腫、感染性腸炎、子宮内感染、子宮付属器炎、涙嚢炎、麦

	粒腫、外耳炎、中耳炎、副鼻腔炎、歯冠周囲炎、猩紅熱、ジフテリア、百日咳、破傷風、ガス壊疽、アメーバ赤痢
用法・用量	通常、成人にはエリスロマイシンとして 1 日 800～1,200 mg（力価）を 4～6 回に分割経口投与する。
保管条件	室温保存

6.1.2　予測される副作用

被験薬及び対照薬(試験薬)として使用される医薬品はいずれも適応外使用である。予測される副作用は、それぞれの添付文書・インタビューフォームを参照する。

6.2　プロトコル治療

6.2.1　用法・用量

試験薬ジスロマック点滴静注用 500 mg 又はエリスロシン点滴静注用 500mg の投与開始日をプロトコル治療開始日（Day1）とし、入院管理下でプロトコル治療を開始する。Day1 に、ジスロマック点滴静注用 500 mg とエリスロシン点滴静注用 500mg は、プロトコル治療中止基準に該当しない限り、1 日投与量を全量投与する。

同意取得後からプロトコル治療開始前日まで、ABPC 点滴投与あるいは ABPC/SBT 点滴投与を行ってよい。

＜被験群＞

（1） スルバシリン静注用 1.5g

スルバシリン静注用 1.5gを 1 回 1.5g、1 日 4 回　計 14 日間（Day1～Day14）　点滴静注投与する。
6 時間±1 時間毎に、1 時間かけて点滴静注投与する。

（2） ジスロマック点滴静注用 500 mg

ジスロマック点滴静注用 500 mg を1回 500 mg、1 日 1 回　計 4 日間（Day1～Day2、Day8～Day9）点滴静注投与する。
24 時間±2 時間毎に、2 時間かけて点滴静注投与する。

（3） ジスロマック錠 250 mg

ジスロマック錠 250 mg を 1 回 1 錠、1 日 1 回（朝食後）　計 10 日間（Day3～Day7、Day10～Day14）服用する。
服用忘れの場合、服用時間の 4 時間以内に気付いた場合は、すぐに服用する。それ以上経過していた場合は、その回は服用せず、次から通常通り服用を再開する。

＜対照群＞

（1） スルバシリン静注用 1.5g

スルバシリン静注用 1.5gを 1 回 1.5g、1 日 4 回　計 14 日間（Day1～Day14）　点滴静注投与する。

6 時間±1 時間毎に、1 時間かけて点滴静注投与する。

（2） エリスロシン点滴静注用 500mg
エリスロシン点滴静注用 500mg を1回 500 mg、1 日 2 回　計 4 日間（Day1～Day2、Day8～Day9）点滴静注投与する。
12 時間±2 時間毎に、1 回 2 時間以上かけて点滴静注投与する。

（3） エリスロシン錠 200mg
エリスロシン錠 200mg を 1 回 1 錠、1 日 4 回（投与間隔は、各回 4 時間以上 6 時間未満の間隔をあけることを推奨する）　計 10 日間（Day3～Day7、Day10～Day14）服用する。
服用忘れの場合、服用時間の 2 時間以内に気付いた場合は、すぐに服用する。それ以上経過していた場合は、その回は服用せず、次から通常通り服用を再開する。

6.2.2　　プロトコル治療の設定根拠

＜ABPC/SBT について＞
特に妊娠 28 週未満の pPROM は、既に子宮内感染が発生している確率が高率であると予想されることから、妊娠 28 週以降の pPROM とは異なる抗菌薬管理（一般細菌に効果のあるブロードスペクトラムのペニシリン系抗菌薬あるいはセフェム系抗菌薬とウレアプラズマ属に効果のあるマクロライド系抗菌薬）が検討される。
このため、本試験では、国外で推奨されている標準的治療法（ABPC/AMPC ＋ EM）ではなく、ABPC/AMPC に変えてよりブロードスペクトラムの ABPC/SBT とし、投与期間も 7 日間ではなく 14 日間まで投与可能とした。EM の投与期間も、7 日間ではなく、14 日間とした。これによって、従来法では期待できなかった BPD に対する効果を期待出来る可能性があると考えている。

＜EM と AZM について＞
本研究では、EM と AZM について 2 日間点滴静注＋5 日間経口内服を 2 回繰り返す投与方法（スイッチ療法）を設定した。
対照群はプラセボではなく、ABPC/SBT + EM 群とした。現在の標準的治療は、米国では、ABPC (点滴静注)2 日間⇨AMPC (経口内服)5 日間 + EM (点滴静注)2 日間⇨EM (経口内服)5 日間、イギリスでは、EM (経口内服)10 日間、カナダでは、上記のいずれかとなっており、いずれも EM が入っている。今回は、従来の RCT における pPROM の対象よりも妊娠週数が早く、子宮内で一般細菌による混合感染が既に発症しているケースが多いことから、ABPC⇨AMPC は ABPC/SBT (点滴静注)に変更し、更に、投与期間を7日から 14 日へ増やすのが妥当と考えた。対照群で使用する EM の使用方法については、欧米のガイドラインに準じて、EM は点滴と内服を併用する方法（スイッチ療法という）を選択した。更に、従来の RCT における pPROM の対象よりも妊娠週数が早く、ウレアプラズマの子宮内感染が既に発症しているケースが多いことから、投与期間を7日（1 クール）から 14 日（2 クール）へ増やすのが妥当と考えた。
一方、被験群は、EM に対する AZM の有意性を検証することも 1 つの目的であることから、ABPC/SBT の投与方法は変えず、EM の部分を AZM 投与とした。AZM 投与については、骨盤内炎症性疾患に対する国内での第 3 相試験での AZM 投与方法を参考に、AZM (点滴静注)2 日間⇨AZM (経口内服)5 日間を 1 クールとした。そして、最大 2 クールまでこの治療を継続できることにした。この理由は、先行研究において、子宮内でウレアプラズマ感染がある場合、AZM (点滴

静注）3 日間では、1 週間後の羊水中ウレアプラズマ属の消失率が約 40%にとどまっていたこと、3 日間投与を 2 クール実施した 2 症例では、ウレアプラズマは消失しなかったが、子宮内炎症が制御でき、また、BPD の発生は生じなかったことを根拠とした。

6.2.3　用量変更

本研究では、プロトコル治療の投与量の変更は認めない。

6.2.4　中断または休薬

（1）点滴静注薬の場合

被験薬又は対照薬の点滴静注投与開始後、有害事象等が発現し、点滴静注投与の中断が必要と判断された場合に限り、被験薬又は対照薬の点滴静注投与を中断することを可とする。点滴静注投与を中断した場合は、症状が回復し、研究責任医師又は研究分担医師が被験薬又は対照薬の点滴静注投与再開が可能と判断した後に、点滴静注投与を再開する。被験薬又は対照薬の点滴静注投与を中断した後、当該投与日のうちに投与が再開できず、全量を投与できなかった場合は休薬として取り扱う。翌日、再開できない場合にはプロトコル治療を中止する。

被験薬又は対照薬の初回点滴静注投与以降、点滴静注投与前に有害事象等が発現し、休薬が必要と判断された場合、休薬を可とする。休薬は 1 日までとし、2 日以上の休薬が必要な場合は、プロトコル治療を中止する。

（2）錠剤の場合

被験薬又は対照薬の服用開始後、有害事象等が発現し、休薬が必要と判断された場合に限り、休薬を可とする。休薬した場合は、症状が回復し、研究責任医師又は研究分担医師が被験薬又は対照薬の服用再開が可能と判断した後に服用を再開する。

6.2.5　プロトコル治療期間

プロトコル治療期間は、各群ともに 14 日間である。14 日間のプロトコル治療を完遂する前に妊娠終結の判断をした場合は、その時点でプロトコル治療を中止する。

6.2.6　プロトコル治療の中止基準

以下のいずれかに該当する場合、当該被験者（母体、胎児）に対するプロトコル治療を中止し、被験者（母体、胎児）の安全を確保する。プロトコル治療を中止した場合は、被験者（母体、胎児）毎に適切な抗菌薬治療を検討し、切り替える。

プロトコル治療中止後も「10.1 被験者の参加中止」に該当しない限り当該被験者（母体、胎児）の研究参加は継続するものとし、研究責任医師又は研究分担医師はスケジュール（7.5）に従い、観察、調査、検査を継続する。

（1）Lencki の診断基準*またはその他の理由で子宮内感染症が強く疑われる場合

Lencki による臨床的絨毛膜羊膜炎の診断基準*

1）母体に 38 度以上の発熱が認められ、かつ以下の 4 項目（A～D）中、1 項目以上認める場合：A. 母体頻脈≧100/分、B. 子宮の圧痛、C. 腟分泌物/羊水の悪臭、D. 母体の白血球数≧15,000/μL

2)母体体温が 38 度未満であっても、上記の 4 項目すべてを認める場合
ただし、肺炎、腎盂腎炎、虫垂炎、髄膜炎、インフルエンザなどにより、1)に合致する可能性があることから、母体発熱時にはこれらも可能な限り鑑別診断する。
（2） 腟培養で緑膿菌又は多剤耐性菌が検出された場合
（3） 有害事象等が発現し、点滴静注薬の試験薬を 2 日以上休薬することが必要な場合
（4） 妊娠終結に至った場合
（5） 被験者がプロトコル治療の中止を申し出た場合
（6） その他、研究責任医師又は研究分担医師がプロトコル治療の継続を困難と判断した場合

6.2.7 包装・表示

試験薬は、市販のスルバシリン静注用 1.5g、ジスロマック点滴静注用 500 mg、ジスロマック錠 250 mg、エリスロシン点滴静注用 500mg、エリスロシン錠 200mg を研究用に購入して使用する。また、薬剤調整、投与に必要な、注射用水、生理食塩水、及び、5%ブドウ糖液も研究用に購入して使用する。試験薬は、通常診療で処方されている薬剤とは区別して管理及び使用する。詳細は、研究用の試験薬管理手順書に別途規定する。

6.2.8 事前準備・事後処理

スルバシリン静注用 1.5g を 100 mL の補液に溶解し、1 時間かけて点滴静注する。溶解後は速やかに使用する。
ジスロマック点滴静注用 500 mg、を注射用水 4.8 mL に溶解した液（濃度 100 mg/mL）を、5 %ブドウ糖注射液等の配合変化がないことが確認されて いる輸液を用いて注射溶液濃度 1.0 mg/mL に希釈する。100 mg/mL 溶液を調製の際には、注射用水以外での調製データ はないことから、注射用水以外の溶液を使用しないこと。溶解後速やかに使用する。
エリスロシン点滴静注用 500mg の注射液は注射用水で 5%溶液をつくり、これをブドウ糖注射液、 生理食塩液等で希釈して点滴静注溶液とする。5%溶液を調製する には、本剤 1 バイアルに注射用水 10mL を加える。5%溶液調製の際には、生理食塩液あるいは無機塩類を含有する溶液を使用しない。5%溶液をさらに希釈する際には、注射用水を使用しない。5%溶液は冷蔵庫内で 2 週間安定である。
ジスロマック錠 250 mg とエリスロシン錠 200mg は錠剤の内用薬であり、服用直前に PTP シートから取り出す。事前準備は特にない。

6.3 管理方法

6.3.1 保管・交付

試験薬は研究用に購入し、各実施医療機関の薬剤部または薬剤科等で適切に保管する。交付する際は研究用として処方し、研究責任医師または研究分担者の薬剤師または医師から直接、被験者に提供する。服用忘れによる未使用薬及び期限切れ薬剤は、院内の薬剤廃棄手順に従って通常通り廃棄する。詳細は、研究用の試験薬管理手順書に別途規定する。

6.3.2　廃棄・返却

研究期間中に研究用医薬品の破損や汚染、その他の不備等により研究用医薬品を使用しない場合、試験薬管理表に廃棄するバイアル数、製造番号、使用期限、廃棄の理由、廃棄後の在庫数と共に記録する。廃棄の際は、各実施医療機関の規定に従って、医療用廃棄物として廃棄する。

試験薬投与期間の終了後、未使用の試験用医薬品の返却等は行わず、廃棄の場合と同様に医療用廃棄物として廃棄する。

6.4　併用薬

6.4.1　併用薬

同意取得日から研究参加終了（又は研究参加中止時）までに、以下の薬剤を被験者（母体、胎児）（母体）に対して使用した際には、以下の項目を eCRF に入力する。

・塩酸リトドリン
・硫酸マグネシウム
・ベタメタゾン
・プロトコル治療期間終了後の感染増悪時に使用された抗菌薬

また、プロトコル治療開始日から妊娠終結時（又は研究参加中止時）までに、胎児に有害事象が発現し、その有害事象に対して試験薬以外の薬剤又は療法を使用した際にも、以下の項目を eCRF に入力する。

薬剤名／療法名
投与経路
処置の開始日及び終了日
使用（処置）理由

なお、治療を目的としない生理食塩水等補液又は溶解液は入力不要とする。

6.4.2　併用制限薬・併用制限治療

研究参加期間中の副腎皮質ホルモン剤の使用は、以下の目的でのみ、同意取得から妊娠終結までの使用は可とする。

（1） 早産が予期される場合における母体投与による胎児肺成熟を介した RDS の発症抑制目的での使用
（2） 皮膚、気管支、耳鼻咽喉、眼、口腔内、肛門、腟等に対する局所投与
（3） 研究参加前から合併症の治療目的でのプレドニゾロンの使用

同意取得後からプロトコル治療開始までの抗菌薬の使用は、研究責任医師又は研究分担医師が必要と判断した場合に、ABPC 点滴投与あるいは ABPC/SBT 点滴投与のみ可とする。

6.4.3　プロトコル治療終了後の感染増悪時の抗菌薬治療

プロトコル治療終了後妊娠終結までに感染増悪を認めた場合は、研究責任医師又は研究分担医師が適切と判断した、マクロライド系以外の抗菌薬治療を行うことができる。

本研究では感染増悪とは以下のいずれかと定義する。

（1）プロトコル治療期間中よりも WBC 数又は CRP 値が増悪し、研究責任医師又は研究分担医師が抗菌薬治療を必要と判断した場合
（2）膣培養で緑膿菌又は多剤耐性菌を認めた場合

6.4.4　併用禁止治療

対照群では、同意取得からプロトコル治療期間終了まで、次の薬剤は併用できない。

（1）エリスロマイシン以外のマクロライド製剤
（2）エルゴタミン（エルゴタミン酒石酸塩、ジヒドロエルゴタミンメシル酸塩）含有製剤 ［クリアミン®、ジヒデルゴット®等］
（3）ピモジド［オーラップ®］
（4）アスナプレビル［スンベプラ®］

設定根拠
（1）：対照薬の同種同効薬のため設定した。
（2）～（4）：対照薬は CYP3A と結合し、複合体を形成するため、これらの薬剤代謝を抑制することがあるため設定した。

被験群では、同意取得から治療観察期終了まで、次の薬剤は併用できない。
（1）　アジスロマイシン以外のマクロライド製剤

設定根拠
（1）：被験薬対照薬の同種同効薬のため設定した。

7　研究方法及び手順

7.1　被験者リクルート

実施医療機関の産婦人科受診中の妊婦のうち、事前のカルテ調査により適格性基準を満たす可能性がある患者に対して、外来受診時に担当医が研究に関して案内する。

各実施医療機関の関連産婦人科医師に、本研究について紹介し、妊娠 22 週 0 日～27 週 6 日で pPROM を発症した妊婦に対しては、可能な限り抗菌薬投与を行う前に紹介すること、抗菌薬投与を ABPC 点滴あるいは ABPC/SBT 点滴投与を行っていただくことを協力依頼する。

7.2 同意取得

同意取得方法に従って説明した後、説明した旨及び同意を得た場合はその旨を診療記録に記載する。説明日には同意せず、後日、同意の意思を確認する場合は、同意書に説明日と医師の署名をし、説明書と共に研究対象者に提供する。説明日と同意日が同日となる場合は、研究参加の検討について、十分な時間を与えてから同意をした旨を記録する。

同意取得後、同日にスクリーニングを実施することができる。同日にスクリーニングを実施しない場合は、同意日より 3 日以内にスクリーニングを行う。

7.3 スクリーニング

7.3.1 実施項目

スクリーニングでは同意取得から登録までに以下の検査及び観察・評価を行う。
スクリーニングの結果、5.1 適格性基準に合致したら当該被験者（母体、胎児）を研究に登録し、Day1（投与開始日）の実施項目を経て、試験薬の服用を開始する。

7.3.2 スクリーニング脱落

スクリーニング検査の結果、適格性基準を満たさなかった被験者は、スクリーニング脱落例となり、研究に参加できない。スクリーニング脱落例の再スクリーニングは行わない。

7.4 被験者登録

研究責任医師又は研究分担医師は以下の手順で被験者を登録する。

（1） 同意を取得した全ての被験者に対し、実施医療機関で以下の規則に基づき被験者識別コードを付与する。同意取得から研究終了まで同一の被験者識別コードを使用する。
被験者識別コードの付与方法
被験者識別コード：PROM-AZM-YYY-ZZZ
YYY：実施医療機関番号
ZZZ：当該実施医療機関で同意を取得した被験者の通し番号

（2） 同意取得後、スクリーニング検査を行い、選択基準を全て満たし、除外基準のいずれにも該当しないことを確認する。

（3） REDCap にログインし、適格性について入力する。

（4） REDCap に入力された情報に基づいて適格と判定された被験者は、REDCap により被験群又は対照群のいずれかに割り付けられる。REDCap 上で割り付け群が提示される。

7.5 観察項目及び収集する情報・手順

スケジュール（表 7.5-1 及び表 7.5-2）に従い、各規定日に必要な観察及び検査等を行う。特に記載がない限り、プロトコル治療開始日（Day 1）の観察、調査、検査は、起床から試験薬の投与前に行う。規定日の観察及び検査等で実施及び収集する内容は以下のとおりとする。

＜表 7.5-1 被験者(母体、胎児)のスケジュール＞

期間	SC 期[a]		治療観察期[b]								妊娠終結後観察期		研究参加中止時[d]	研究参加中止後フォローアップ[e]
実施項目	スクリーニング期 Day -3-0	登録 Day 0	Week 1 Day 1	Week 2 Day 8 ± 2day	Week 3 Day 15 ± 2 day	Week 4 Day 22 ± 2 day	Week 5 Day 29 ± 2 day	Week 6 Day 36 ± 2 day	Week 7 Day 43 ± 2day	Week 8～[c] Day 50 ± 2 day	妊娠終結時 ± 1 day	退院診察時 ± 2 day		
同意取得[f]	X													
被験者背景情報・体重	X[o]													
登録・ランダム化		X												
入院[g]			X	X	X	X	X	X	X	X	X	X		
プロトコル治療[h]			X	X										
身体所見[i]	X		X	X	X	X	X	X	X	X	X	X	X	
バイタル (血圧・脈拍・体温)[i]	X		X	X	X	X	X	X	X	X	X	X	X	
血液学的検査[i]	X[p]			X	X	X	X	X	X	X	X	X	X	
血液生化学検査[i]	X[p]			X	X	X	X	X	X	X	X	X	X	
尿検査 (定性：蛋白・尿糖)[i]	X[p]			X	X	X	X	X	X	X	X	X	X	
心電図	X[p]													
腟内細菌培養(施設)[i]			X[r]	X[s]	X	X	X	X	X	X	X			
腟内ウレアプラズマ培養[j]			X[r]	X[s]										
腟内ウレアプラズマ PCR[j]			X[r]	X[s]										
胎盤ウレアプラズマ培養[j]											X			
胎盤ウレアプラズマ PCR[j]											X			
胎盤病理組織検査											X			
胎児推定体重[i]	X[q]			X	X	X	X	X	X	X				
胎児超音波検査(ドップラー・羊水量)[i]	X[q]			X	X	X	X	X	X	X				
併用薬の確認[k]	X		X	X	X	X	X	X	X	X	X	X	X	X
有害事象の確認[l]			X	X	X	X	X	X	X	X	X	X	X	X
妊娠終結に関する調査[m]											X			
分娩所見[n]											X			

a. スクリーニング期(同意取得から登録・ランダム化まで)は最大 3 日間とする。

b. 特に記載のない限り、規定の観察、調査、検査は試験薬の投与前に行う。プロトコル治療を完遂する前に妊娠終結の判断をした場合には、その時点でプロトコル治療を中止し、妊娠終結後に妊娠終結後観察期へ移行する。

c. Week 8 以降は、Week 8 で規定された検査項目を妊娠終結の判断をした日まで、週 1 回行う。

d. 研究参加中止後 3 日以内に行う。

e. 母体の 1 か月健診時を目安に行う。

f. スクリーニング検査開始までに文書による同意取得を行う。

g. 少なくともプロトコル治療開始日から妊娠終結 3 日後までの期間は入院管理を行う。
h. 同意取得後 4 日以内にプロトコル治療を開始する。
i. Week3 以降は、妊娠終結の判断がされる時点まで、週 1 回実施する。ただし、血液学的検査及び血液生化学検査については、Week 4 以降は妊娠終結の判断がされる時点まで必要時とする。
j. 可能な場合に実施する。被験者（母体）に新型コロナウイルス感染症が判明した場合は、検体を採取せず、採取ずみの検体は廃棄する。
k. 有害事象の発現により研究参加を中止した被験者に関しては、研究参加中止の原因となった有害事象に対して使用した併用薬及び併用療法の調査を中止後も継続し、研究参加中止後フォローアップまで調査する。
l. 被験者（母体・胎児）の有害事象を調査する。母体については、プロトコル治療開始日から妊娠終結後観察期（又は研究参加中止時）まで調査する。胎児については、プロトコル治療開始日から妊娠終結日（又は研究参加中止日）まで調査する。ただし、有害事象の発現により研究参加を中止した被験者被験者（母体・胎児）に関しては、研究参加中止の原因となった有害事象については調査を継続し、研究参加中止後フォローアップまで調査する。
m. 妊娠終結判断日、胎児を娩出した時刻、妊娠終結判断理由（母体要件、胎児要件）、妊娠終結日を調査する。
n. 分娩様式、死産の有無及び死亡を確認した日、胎盤重量、胎盤梗塞の有無を調査する。
o. スクリーニング期体重(Kg)は、同意取得前 14 日以内に測定され適切に記録されている場合は、スクリーニング期のデータとして使用してよい。
p. 血液学的検査、血液生化学検査、尿検査（定性：蛋白・尿糖）、心電図は、同意取得前 3 日以内に行われ適切に記録されている場合は、スクリーニング期のデータとして使用してよい。
q. 胎児推定体重、胎児超音波検査（ドップラー・羊水量）は、同意取得前 7 日以内に行われ適切に記録されている場合は、スクリーニング期のデータとして使用してよい。
r. 原則、プロトコル治療開始前に行うが、同意取得後スクリーニング期に行ってもよい。
s. Day8 の腟内ウレアプラズマ培養及び腟内ウレアプラズマ PCR のための検体採取は Day6～Day10 に行う。ただし、Day8 までにプロトコル治療を中止した場合は、これらの検査は行わない。

＜表 7.5-2 児のスケジュール＞

実施項目	妊娠終結時（出生時）	日齢 3（±1 day）	日齢 7（±3 day）	日齢 28[†]（±2 day）	修正 36 週 0 日[#]（±2 day）	児の退院時（±7 day）	母体研究参加中止時[a]
臍帯動脈血ガス検査	X						
新生児所見[b]	X						
アプガースコア[c]	X						
血液ガス検査[d]	X	X[k]					
血液学的検査[d]	X[i]	X[k]					
血液生化学的検査[d]	X[i、j]	X[k]					
咽頭ウレアプラズマ培養[e]	X						
咽頭ウレアプラズマ PCR[e]	X						
耳腔ウレアプラズマ培養[e]	X						
耳腔ウレアプラズマ PCR[e]	X						
胸部 X 線	X		X[l]	X	X[l]		
入院期間	X	X	X	X	X	X	
呼吸器管理の有無、種類及び期間	X	X	X	X	X	X	
BPD_{36}の有無					X[m]		
BPD_{28}の有無				X			
新生児の合併症[f]	X	X	X	X	X	X	
治療内容[g]	X	X	X	X	X	X	
重篤な有害事象[h]	X	X	X	X	X	X	X

† 日齢 28 までに退院している児の場合は、1 か月健診時に行う。

\# 出生時在胎週数 32 週未満で出生した児の場合は、修正 36 週 0 日にまたは児の退院時のいずれか早いほうの時点で評価する。出生時在胎週数 32 週以上で出生した児の場合は、日齢 56 または退院時のいずれか早いほうの時点で評価する。

a. 研究参加中止後 3 日以内に行う。

b. 新生児の性別、出生時体重（g）、出生時身長（cm）、出生時頭囲（cm）、新生児の発育度を調査する。

c. 出生 1 分後、5 分後に評価する。

d. 動脈血、静脈血、毛細血管血のいずれでも測定可能とする。

e. 被験者（母体）に新型コロナウイルス感染症が判明した場合は、検体を採取しない。

f. 頭蓋内出血、脳室周囲白質軟化症、新生児呼吸窮迫症候群、胎便吸引症候群、新生児一過性多呼吸、遷延性肺高血圧、敗血症、壊死性腸炎、限局性腸穿孔、症候性動脈管開存症、声門下狭窄、喉頭軟化症、上気道疾患（その他）の有無を調査する。

g. 酸素投与、ステロイドの静注・吸入、吸入一酸化窒素、サーファクタント、カフェイン、アジスロマイシン投与について調査する。児の手術について、術式名、手術実施日、手術目的、及び手術目的の挿管の有無を調査する。

h. 児については妊娠終結日から児の退院時（又は母体研究参加中止時）までの情報を調査する。

i. 臍帯血で測定することも可とする。

j. IgM は出生時のみ測定する。

k.　出生体重 1000g 未満の場合、測定しなくてもよい。
l.　必要と判断した場合に行う。
m.　退院時に評価する場合は、退院時（－2 day）に評価する。

7.5.1　被験者（母体、胎児）の観察・調査項目及び実施時期

（1）被験者背景情報
- 年齢（歳）
- 人種
- 血液型（ABO 型、Rh 型）
- 喫煙
- 文書同意取得日
- 妊娠歴（今回の妊娠を含まない）
- 出産歴（今回の妊娠を含まない）
- 不妊治療歴

  ART (assisted reproductive technique、生殖補助医療)：（複数選択可）1. in vitro fertilization and embryo transfer (IVF-ET)、2. gamete intrafallopian transfer (GIFT). 3. 顕微授精、4. IVF-ET + 顕微授精、5. 融解胚移植、6. 胚盤胞移植、7. その他（自由記載）
- 登録日妊娠週数
- 身長(cm)
- 妊娠前体重(Kg)
- スクリーニング期体重(Kg)

  ただし、同意取得前 14 日以内に体重が測定され適切に記録されている場合は、スクリーニング期のデータとして使用してよい。
- 合併症（同意取得日時点で有する疾患・症状等）

  高血圧、白衣高血圧、I 型糖尿病、II 型糖尿病、妊娠糖尿病、精神疾患、てんかん、消化器疾患、甲状腺疾患、膠原病、悪性腫瘍、神経疾患、呼吸器系疾患、その他
- 既往歴（子宮頸部円錐切除術、後期流産歴、早産歴）
- アレルギー
- pPROM 発生日、pPROM 発生妊娠週日
- pPROM 診断根拠（複数選択可）：1. Leak、2. Pooling

実施時期：スクリーニング期

（2）身体所見

研究期間中、被験者（母体、胎児）（母体）の症状・所見の変化、新たな症状・所見の有無等について、評価する。

実施時期： スクリーニング期、Day1、Day8、Week3～*1、妊娠終結時、退院診察時、研究参加中止時

*1：Week3 以降は、妊娠終結の判断がされる時点まで、週 1 回実施する。

（3）試験薬の投与状況

以下の項目を電子症例報告書(eCRF)に入力する。
- 試験薬の投与日、投与回数

- 試験薬の 1 日投与量
- 中断の有無、中断した場合はその理由
- 休薬の有無、休薬した場合はその理由

実施時期:治療観察期

(4) 妊娠終結に関する調査

妊娠終結判断日、妊娠終結判断理由(母体要件、胎児要件)、妊娠終結日、胎児が娩出した時刻を調査し、その結果を eCRF に入力する。

実施時期:妊娠終結時

(5) 分娩所見

分娩様式(自然分娩、分娩誘発、帝王切開、吸引分娩、骨盤位牽出術)、帝王切開の場合は、母体適応の有無とその内容、胎児適応の有無とその内容、死産の有無及び死亡を確認した日、胎盤重量、胎盤梗塞の有無を調査し、その結果を eCRF に入力する。

実施時期:妊娠終結時

(6) 併用薬の確認

併用薬を確認し、その結果を eCRF に入力する。子宮収縮抑制剤(種類も聴取)、降圧剤、インスリン、向精神薬、抗てんかん薬、サラゾピリン、甲状腺薬、チアマゾール/プロピルチオウラシル、抗腫瘍薬、吸入ステロイド薬、気管支拡張薬、低用量アスピリン、ヘパリン自己注射については、スクリーニング期のみ調査し、その結果を eCRF に入力する。

実施時期:スクリーニング期、治療観察期、妊娠終結後観察期、研究参加中止時、研究参加中止後フォローアップ

(7) 有害事象の確認

有害事象を確認し、その結果を eCRF に入力する。

実施時期: 治療観察期、妊娠終結後観察期、研究参加中止時、研究参加中止後フォローアップ

7.5.2 被験者(母体、胎児)における検査項目及び実施時期

(1) バイタルサイン

バイタルサインを 1 日 1 回測定し、測定時刻及びその結果を eCRF に入力する。

検査項目:体温、脈拍数、収縮期血圧及び拡張期血圧

実施時期:スクリーニング期、Day1*1、Day8*1、Week3〜*2、妊娠終結時、退院診察時、研究参加中止時

*1:試験薬投与日は、原則投与前に, 投与後であれば投与開始後 2 時間以内に測定する。

*2:Week3 以降は、妊娠終結の判断がされる時点まで、週 1 回行う。

(2) 血液学的検査

血液学的検査を行い、その結果を eCRF に入力する。

検査項目:WBC、RBC、Hb 濃度、Ht 値、PLT、白血球分画

実施時期：スクリーニング期[*1]、Day8[*2]、Week3～[*2*3]、妊娠終結時、退院診察時、研究参加中止時

＊1：同意取得前 3 日以内に行われ適切に記録されている場合は、スクリーニング期のデータとして使用してよい。

＊2：試験薬投与日は、原則投与前に，投与後であれば点滴を行っていない上腕を用いて，投与開始後 2 時間以内に測定する。

＊3：Week3 は週 1 回、Week 4 以降は妊娠終結の判断がされる時点まで必要時とする。

（3） 血液生化学検査

血液学的検査を行い、その結果を eCRF に入力する。

検査項目：TP、ALB、T-Bil、AST、ALT、LDH、BUN、Cr、UA、Na、K、Cl、Ca、Mg、Amy、CK、CRP

実施時期：スクリーニング期[*1]、Day8[*2]、Week3～[*2*3]、妊娠終結時、退院診察時、研究参加中止時

＊1：同意取得前 3 日以内に行われ適切に記録されている場合は、スクリーニング期のデータとして使用してよい。

＊2：試験薬投与日は、原則投与前に，投与後であれば点滴を行っていない上腕を用いて，投与開始後 2 時間以内に測定する。

＊3：Week3 は週 1 回、Week 4 以降は妊娠終結の判断がされる時点まで必要時とする。

（4） 尿定性検査

尿定性検査を行い、その結果を eCRF に入力する。

検査項目：蛋白、尿糖

実施時期：スクリーニング期[*1]、Day8[*2]、Week3～[*2*3]、妊娠終結時、退院診察時、研究参加中止時

＊1：同意取得前 3 日以内に行われ適切に記録されている場合は、スクリーニング期のデータとして使用してよい。

＊2：試験薬投与日は、原則投与前に，投与後であれば投与開始後 2 時間以内に測定する。

＊3：Week3 以降は、妊娠終結の判断がされる時点まで、週 1 回行う。

（5） 心電図

心電図検査を行い、その結果を eCRF に入力する。

実施時期：スクリーニング期[*1]

＊1：同意取得前 3 日以内に行われ適切に記録されている場合は、スクリーニング期のデータとして使用してよい。

（6） 腟内細菌培養

腟内細菌培養検査を行い、その結果を eCRF に入力する。

実施時期：Day1（スクリーニング期でも可）、Day8、Week3～[*1]、妊娠終結時

＊1：Week3 以降は、妊娠終結の判断がされる時点まで、週 1 回行う。嫌気培養は不要。

(7) 膣内ウレアプラズマ培養（中央測定）
被験者（母体）に新型コロナウイルス感染症が判明した場合は、検体を採取せず、採取ずみの検体は廃棄する。
実施時期：Day1（スクリーニング期でも可）、Day 8（±2 日）

(8) 膣内ウレアプラズマ PCR（中央測定）
被験者（母体）に新型コロナウイルス感染症が判明した場合は、検体を採取せず、採取ずみの検体は廃棄する。
実施時期：Day1（スクリーニング期でも可）、Day 8（±2 日）

(9) 胎盤ウレアプラズマ培養（中央測定）
可能な場合に実施する。被験者（母体）に新型コロナウイルス感染症が判明した場合は、検体を採取せず、採取ずみの検体は廃棄する。
実施時期：妊娠終結時（分娩時）

(10) 胎盤ウレアプラズマ PCR（中央測定）
可能な場合に実施する。被験者（母体）に新型コロナウイルス感染症が判明した場合は、検体を採取せず、採取ずみの検体は廃棄する。
実施時期：妊娠終結時（分娩時）

(11) 羊水ウレアプラズマ培養及び羊水ウレアプラズマ PCR（中央測定）
オプションとして、羊水穿刺を行っている施設では、3 回に限り、羊水のウレアプラズマ培養、PCR を提出できる。被験者（母体）に新型コロナウイルス感染症が判明した場合は、検体を採取せず、採取ずみの検体は廃棄する。
実施時期：施設の判断で 3 回まで

(12) 胎盤病理組織検査
組織学的絨毛膜羊膜炎の有無【Blanc 分類（絨毛膜羊膜炎、臍帯炎）】
実施時期：妊娠終結時（分娩時）から退院診察時までに行う。

(13) 胎児の推定体重
胎児の体重を超音波検査により推定し、その結果を eCRF に入力する。
実施時期：スクリーニング期、Day8（±2 日）、Week3～[*1]
*1：同意取得前 7 日以内に行われ適切に記録されている場合は、スクリーニング期のデータとして使用してよい。
*2：Week3 以降は、妊娠終結の判断がされる時点まで、週 1 回行う。

(14) 胎児超音波検査（羊水量・ドップラー）
胎児超音波検査を行い、その結果を eCRF に入力する。
羊水量：羊水ポケット、羊水インデックスを超音波検査により測定する。
ドップラー：臍帯下垂の有無を確認する。
実施時期：スクリーニング期、Day8（±2 日）、Week3～[*1]

＊1：Week3 以降は、妊娠終結の判断がされる時点まで、週 1 回行う。

7.5.3 児の観察・調査項目及び実施時期

（1） 新生児所見

アプガースコア（出生 1 分後、5 分後）、新生児の性別、出生時体重（g）、出生時身長（㎝）、出生時頭囲（㎝）、新生児発育度を eCRF に入力する。

新生児発育度は、light-for-dates、small-for-dates、appropriate-for-dates、large-for-dates、heavy-for-dates のいずれかで評価する。

実施時期：妊娠終結時（出生時）

（2） 児の入院期間

児の入院期間（出生日から退院日もしくは転院日までの合計日数）を調査し、その結果を eCRF に入力する。修正 36 週 0 日時点（又は中止後フォローアップ時点）で児の入院が継続していた場合は、可能な限り退院まで調査する。

実施時期：妊娠終結時（出生時）、修正 36 週 0 日[*1]、児の退院時

＊1：出生時在胎週数 32 週以上で出生した児の場合は、日齢 56 または退院時のいずれか早いほうの時点で評価する。

（3） 児の呼吸管理

児の呼吸管理の有無と、呼吸管理を実施していた場合には、呼吸管理方法別（侵襲的[*1]、非侵襲的[*2]、その他）に期間、使用目的を調査し、eCRF に入力する。修正 36 週 0 日時点（又は母体研究参加中止時点）で児の入院が継続していた場合は、可能な限り退院まで調査する。

実施時期：妊娠終結時（出生時）、日齢 3、日齢 7、日齢 28[*3]、修正 36 週 0 日[*4]、児の退院時

＊1：気管挿管による人工呼吸管理

＊2：（同期式）経鼻的間欠的陽圧換気（(s)NIPPV）、経鼻的持続陽圧呼吸法（NCPAP）、呼気吸気変換方式経鼻的持続陽圧呼吸法（NDPAP）、非侵襲的神経調節補助換気（NIV-NAVA）、高流量鼻カニューレ（HFNC）、深呼吸気道陽圧（SiPAP）、体外式気道陽圧（BiPAP）

＊3：日齢 28 までに退院している児の場合は、1 か月健診時に評価する。

＊4：出生時在胎週数 32 週以上で出生した児の場合は、日齢 56 または退院時のいずれか早いほうの時点で評価する。

（4） 児への酸素投与

児への酸素投与の有無と酸素投与を行っていた場合は、酸素投与方法別に期間、酸素濃度、酸素流量、吸入酸素濃度（FiO_2、酸素マスク、口元酸素吹き流し、フードの場合）を調査し、eCRF に入力する。修正 36 週 0 日時点（又は母体研究参加中止時点）で新生児の入院が継続していた場合は、可能な限り退院まで調査する。

実施時期：妊娠終結時（出生時）、日齢 3、日齢 7、日齢 28[*1]、修正 36 週 0 日[*2]、児の退院時

＊1：日齢 28 までに退院している児の場合は、1 か月健診時に評価する。

＊2：出生時在胎週数 32 週以上で出生した児の場合は、日齢 56 または退院時のいずれか早いほうの時点で評価する。

（5）BPD_{28}の有無

BPD_{28}発症の有無は、「日齢 28 時点での酸素または呼吸サポート使用の有無」と定義する [1)]。

実施時期：日齢 28*1（±2day）

*1：日齢 28 までに退院している児の場合は、1 か月健診時に評価する。

（6）BPD_{36} の有無

BPD_{36}発症の有無は、「修正 36 週時点での酸素または呼吸サポート使用の有無（出生時在胎週数 32 週以上で出生した児の場合は、日齢 56 または退院のいずれか早いほうの時点での酸素または呼吸サポート使用の有無）」と定義し、その重症度の評価は、表 7.5-3　BPD 重症度分類を使用する [1)]。

＜表 7.5-3　BPD 重症度分類 [1)]＞

出生時在胎週数	32 週未満	32 週以上
評価時期	修正 36 週または退院のいずれか早いほう	日齢 56 または退院のいずれか早いほう
	少なくとも 28 日間以上、22%以上の酸素を使用している	
Mild BPD	修正 36 週 0 日または退院のいずれか早い時点で酸素使用なし	日齢 56 までまたは退院のいずれか早い時点で酸素使用なし
Moderate BPD	修正 36 週 0 日または退院のいずれか早い時点で、30%未満の酸素使用	日齢 56 または退院のいずれか早い時点で、30%未満の酸素使用
Severe BPD	修正 36 週 0 日または退院のいずれか早い時点で、30%以上の酸素または陽圧換気（人工換気または NCPAP）が必要	日齢 56 または退院のいずれか早い時点で、30%以上の酸素または陽圧換気（人工換気または NCPAP）が必要

評価時点で、呼吸状態について評価し、以下の①～⑭のうち⑧～⑪に該当する児には、酸素減量テスト*1 を行って、BPD 重症度の判定を行う。上気道疾患は除く。

① 気管挿管による人工換気 NIPPV・NIV-NAVA による非挿管陽圧換気を行っている
② マスクで酸素 30%以上投与されている
③ 非挿管下での陽圧換気 (*)使用中で酸素 30%以上投与されている
④ 鼻カニュラで 100%酸素 251mL/min 以上使用している
⑤ 酸素投与のみ（FiO2 0.30 以上）で退院した
⑥ 酸素投与のみ（FiO2 0.30 未満）で退院した
⑦ 人工呼吸器ありで退院した
⑧ 酸素 29%以下かつ非挿管下での陽圧換気あり（high flow、biPAP、CPAP を含む）を行っている
⑨ フードまたは鼻カニュラで FiO2 0.30 以下の酸素使用している
⑩ 鼻カニュラで 100%酸素を 250mL/min 以下で使用している
⑪ 修正 36 週 0 日前の連続した 14 日間に酸素や呼吸補助を使用しても、修正 36 週 0 日時点で室内気で SpO2 91%以上を維持できない
⑫ 酸素投与期間が 28 日未満である

⑬ 何らかの呼吸補助や酸素投与なしで、少なくとも 14 日間 SpO2 91%以上を維持している
⑭ 呼吸補助や酸素投与なしで自宅退院した

実施時期：修正 36 週 0 日（±2day）、ただし出生時在胎週数 32 週以上で出生した児の場合は、日齢 56 または退院時のいずれか早い時点で行う。
手術等により、酸素減量テストを行えない場合は、手術等の影響がなくなったと判断した時点で酸素減量テストを行う。

＊1 酸素減量テストの方法 [18,19]
酸素減量テスト実施中は、呼吸心拍モニターと SpO_2 モニターで継続的に監視しながら児を直接観察する。可能な限り日中の栄養摂取の 1 時間後に開始する。酸素減量テストは次の 3 つの評価期間で構成される。酸素減量テストの結果、心肺イベント、大きな心肺イベント発生状況を eCRF に入力する。

1） ベースライン評価期間（15 分間）
児が安定していること（SpO_2 90%以上）を確認して開始する。平均酸素飽和度（SpO_2）と心肺イベント（以下に定義）を 15 分間記録する。児が不安定だった場合（SpO_2 90%未満）は中止とし、評価開始から 12～24 時間以内に再評価する。再評価時に児が不安定だった場合は酸素減量テスト失敗と判断する。

2） 酸素/圧減量期間
ベースライン評価期間の 15 分間安定していれば（SpO_2 90%以上を維持していれば）、次の要領で段階的に酸素や圧・フローを減量する。

●CPAP/DPAP/biPAP の圧
圧を 5 分ごとに"1 mmHg（約 1cmH2O）"ずつ徐々に 5 mmHg（約 4cmH2O）になるまで減圧する。その後、酸素濃度を室内気まで下げて、呼吸サポートを中止する。

●酸素
児が室内気を呼吸するまで、経鼻酸素投与の場合は 5 分ごとに最大 100 mL / min ずつ減少して中止する。フードまたはマスクで酸素投与の場合は、FiO2 を最大 0.10 ずつ減少して中止する。

●高流量鼻カニューレ（HFNC）
流量を 5 分ごとに 1 L / min ずつ 3L / min まで減量、その後中止する。

3) 安定性観察期間
酸素および呼吸サポートの離脱に成功した児を、SpO_2 モニターと呼吸心拍モニターを使用して 30 分間観察する。

3 つの評価期間の間に、心肺イベント、大きな心肺イベントの発生を記録する。

●心肺イベント
i）SpO_2 90%未満の継続（5 分未満）。
ii）徐脈（心拍数 80 回/分未満が 10 秒間以上）
iii）無呼吸（15 秒以上の呼吸停止）

●大きな心肺イベント
i）5 分間または酸素減量テスト期間中に、3 回以上連続した無呼吸または徐脈
ii）SpO_2 80%未満が 15 秒以上
iii）回復するために刺激を必要とする $SpO_2$70%未満への低下

酸素減量テスト
●成功
大きな心肺イベントがなく、安定観察期間に室内空気で SpO_2 90%以上を維持できた場合

●失敗
i）酸素/圧減量期間、安定性観察期間中に大きな心肺イベントが発生した場合
ii）酸素/圧減量期間、安定性観察期間中にいずれかの心肺イベントが発生し、担当医師が継続できないと判断した場合

（7） 児の合併症
頭蓋内出血、脳室周囲白質軟化症、新生児呼吸窮迫症候群、胎便吸引症候群、新生児一過性多呼吸、遷延性肺高血圧、敗血症、壊死性腸炎、限局性腸穿孔、症候性動脈管開存症、声門下狭窄、喉頭軟化症、上気道疾患（その他）の有無を調査する。
頭蓋内出血については重症度（3 度、4 度）、診断方法も調査する。
脳室周囲白質軟化症については部位（片側、両側）、診断方法も調査する。
各合併症の診断基準は、別途定める。
実施時期：妊娠終結時（出生時、日齢 3、日齢 7、日齢 28*1、修正 36 週 0 日*2、児の退院時
*1：日齢 28 までに退院している児の場合は、1 か月健診時に評価する。
*2：出生時在胎週数 32 週以上で出生した児の場合は、日齢 56 または退院時のいずれか早いほうの時点で評価する。

（8） 児への治療内容
新生児へのステロイドの静注（投与期間、1 日投与量）・吸入（使用の有無）、吸入一酸化窒素（使用の有無）、サーファクタント（使用の有無）、カフェイン（投与期間、1 日投与量）、アジスロマイシン投与（使用の有無）について調査し、eCRF に入力する。また、児の手術について、術式名、手術実施日、手術目的、及び手術目的の挿管の有無を調査し、eCRF に入力する。
実施時期：妊娠終結時（出生時、日齢 3、日齢 7、日齢 28*1、修正 36 週 0 日*2、児の退院時
*1：日齢 28 までに退院している児の場合は、1 か月健診時に評価する。
*2：出生時在胎週数 32 週以上で出生した児の場合は、日齢 56 または退院時のいずれか早いほうの時点で評価する。

7.5.4　児における検査項目及び実施時期

（1）臍帯動脈血ガス
　臍帯動脈血ガス（pH）を測定する。
　実施時期：妊娠終結時（出生時）

（2）血液ガス検査
　血液ガス（pH）を測定する。動脈血、静脈血、毛細血管血のいずれでも測定可能とする。
　実施時期：妊娠終結時（出生時）、日齢 3*1
　　*1：出生体重 1000g 未満の場合、測定しなくてもよい。

（3）血液学的検査
　血液学的検査は、動脈血、静脈血、毛細血管血のいずれでも測定可能とする。
　検査項目：WBC、RBC、Hb 濃度、Ht 値、PLT
　実施時期：妊娠終結時（出生時）*1、日齢 3*2
　　*1：妊娠終結時（出生時）は、臍帯血で測定することも可とする。
　　*2：出生体重 1000g 未満の場合、測定しなくてもよい。

（4）血液生化学的検査
　血液生化学的検査は、動脈血、静脈血、毛細血管血のいずれでも測定可能とする。
　検査項目：血糖、T-Bil 、AST、ALT　LDH、CK、Na K、Cl、CRP、IgM*1
　実施時期：妊娠終結時（出生時）*2、日齢 3*3
　　*1：IgM は妊娠終結時（出生時）のみ測定する。
　　*2：妊娠終結時は、臍帯血で測定することも可とする。
　　*3：出生体重 1000g 未満の場合、測定しなくてもよい。

（5）咽頭及び耳腔ウレアプラズマ培養（中央測定）
　被験者（母体）に新型コロナウイルス感染症が判明した場合は、検体を採取しない。
　実施時期：妊娠終結時（出生時）

（6）咽頭及び耳腔ウレアプラズマ PCR（中央測定）
　被験者（母体）に新型コロナウイルス感染症が判明した場合は、検体を採取しない。
　実施時期：妊娠終結時（出生時）

（7）胸部 X 線検査
　胸部 X 線について、びまん性の不透亮像、びまん性の泡沫状陰影、不規則索状気腫状陰影の有無を評価し、eCRF に報告する。
　実施時期：妊娠終結時（出生時）、日齢 7 *1、日齢 28*2、修正 36 週 0 日*1、*3
　　*1：必要と判断した場合に行う。
　　*2：日齢 28 までに退院している児の場合は、1 か月健診時に評価する。

*3：出生時在胎週数 32 週以上で出生した児の場合は、日齢 56 または退院時のいずれか早いほうの時点で評価する。

7.5.5 中央測定検体の取扱い

中央測定の PCR 用検体（腟内ウレアプラズマ PCR、胎盤ウレアプラズマ PCR、児の咽頭及び耳腔ウレアプラズマ PCR、オプションとして実施した羊水ウレアプラズマ PCR）は冷蔵庫で保存し、培養用（腟内ウレアプラズマ培養、胎盤ウレアプラズマ培養、児の咽頭及び耳腔ウレアプラズマ培養、オプションとして実施した羊水ウレアプラズマ培養）は、①可能であれば 37℃、②培養装置が無ければ室温（15～25℃）、あるいは③冷蔵庫で一晩静置し、翌日マイナス 70～80℃に保存する。尚、冷蔵庫であれば数日間の保存は可能。マイナス 70～80℃の凍結保存は数か月可とし、送付当日まで冷凍保存する。

中央測定検体は、児の検体採取後 2 か月以内にまとめて常温で、「地方独立行政法人大阪府立病院機構大阪母子医療センター研究所免疫部門」宛にカテゴリーB にて宅急便で送付する。オプションとして、羊水穿刺を行っている施設では、3 回に限り、羊水のウレアプラズマ培養及び PCR 用の検体を提出できる。ただし、新型コロナウイルス感染症が判明した被験者（母体）とその児の検体は廃棄し、「地方独立行政法人大阪府立病院機構大阪母子医療センター研究所免疫部門」に送付しない。詳細は、中央測定検体の取扱い手順書を参照する。

「地方独立行政法人大阪府立病院機構大阪母子医療センター研究所免疫部門」では、ウレアプラズマ属とマイコプラズマ属の培養を行い、菌の培養と PCR を同時に行い、*Ureaplasma urealyticum*、*Ureaplasma parvum*、及び、*Mycoplasma hominis* の有無を検出する。

7.6 実施期間及び登録期間

予定実施期間： jRCT 登録日～2025 年 3 月 31 日
予定登録期間： 2022 年 4 月 1 日～2024 年 3 月 31 日
予定観察期間： 2022 年 4 月 1 日～2024 年 8 月 31 日
jRCT 登録日： 2022 年 2 月 22 日

7.7 登録期間及び観察期間の終了

本研究に登録された症例数が目標症例数に達したら、登録期間を終了とする。ただし、目標症例数に達した時点ですでに研究の候補患者として同意を取得している被験者に関しては、目標症例数に達した後でも登録できるものとする。目標症例数に達した時点で同意を取得していない被験者については原則登録できない。

最終登録被験者の児の観察が完了した時点で観察期間終了とする。

8 同意取得方法

8.1 インフォームド・コンセント

研究参加前に、被験者からインフォームド・コンセントを取得する。本研究の対象者は 18 歳以上の成人した妊婦であるため、代諾者を必要としない。

研究責任医師または分担医師は、臨床研究法に従って作成し、認定臨床研究審査委員会で承認された同意説明文書を使用して、被験者が理解しやすい言葉で研究内容について説明する。被験者の質問に回答し、研究参加について検討する時間を十分に与える。また、同意は被験者の自由意思によるものであり、同意しない場合でも不利な扱いを受けることはないこと、同意後も意思が変わった場合は解析対象集団のデータの固定直前までであればいつでも同意撤回でき、その場合でも不利な扱いを受けることはないことについて強調する。研究参加の同意を得る場合、説明を行った医師及び被験者の署名、説明日、同意日を同意書に記載する。説明文書と同意書の写しを交付し、同意書の原本は実施医療機関で保管する。施設の規則等により、更に同意書の写しを必要とする場合は、説明を行った医師が原本（医師用）を複写し、必要な部署へ提出する。研究に関連する手順は、いかなる手順であっても被験者の同意取得後に実施する。

研究中に、同意取得時には想定されなかった、研究対象者の意思に影響を与える可能性のある新たな情報が得られた場合や、研究対象者の同意に影響を及ぼすような研究内容等の変更が生じた場合は、速やかに同意説明文書を改訂し、研究対象者に説明した上で再度研究参加の意思を確認し、同意を得る。

8.2 18 歳以上の成人した妊婦インフォームド・アセント

18 歳以上の成人した妊婦を対象とした研究であるため該当しない。

8.3 同意撤回

研究参加に同意した後でも、被験者が希望する場合は、解析対象集団のデータの固定直前まででであればいつでも同意を撤回することができる。同意撤回を行う場合、研究責任医師または分担医師は被験者と相談し、同意撤回に係る理由を確認するとともに、同意撤回後に必要な後観察などについて説明する。その後、被験者の同意撤回を可能な限り同意撤回文書により取得する。同意撤回後、研究参加は中止する。研究参加中止後の被験者の安全を確保するため、可能な範囲で被験者の体調変化を確認するなど、後観察を行うよう努める。

9 有害事象及び疾病等

詳細は、「疾病等が発生した場合の対応に関する手順書」に記載する。

9.1 定義

9.1.1 有害事象の定義

本研究の有害事象は、被験者（母体、胎児）ではプロトコル治療開始日から妊娠終結時（又は研究参加中止時）までに生じたあらゆる好ましくない症状、徴候、疾患、臨床検査値の異常のうち、臨床的に問題があるものと定義する。プロトコル治療との因果関係の有無は問わない。児では分娩後から観察終了時までに生じたあらゆる好ましくない症状、徴候、疾患、臨床検査値の異常のうち、臨床的に問題があるものと定義する。ただし、本研究で新生児合併症と定義した、頭蓋内出血、脳室周囲白質軟化症、新生児呼吸窮迫症候群、胎便吸引症候群、新生児一過性多呼吸、遷延性肺高血圧、敗血症、壊死性腸炎、限局性腸穿孔、症候性動脈管科依存症は児の有害事象として取り扱わないが、重篤な有害事象の定義に該当する場合は、重篤な有害事象として報告する。

9.1.2 重大な有害事象の定義

本研究の重大な有害事象は、被験者（母体、胎児）に生じた、胎児死亡、敗血症、intensive care unit (ICU)入室、多臓器不全、人工換気、子宮全摘術とする。

9.1.3 重篤な有害事象の定義

研究責任医師または分担医師が有害事象を以下の基準に該当すると判断した場合、重篤な有害事象とする。

(1) 死亡または死亡につながるおそれ
(2) 治療のために医療機関への入院または入院期間の延長が必要となるもの
(3) 障害または障害につながるおそれ
(4) 1～3に準じて重篤
(5) 後世代における先天性の疾病または異常

研究参加前から予定されていた入院、検査目的の入院や入院期間の延長に関しては重篤な有害事象とはしない。

胎児死亡、児の死亡が発生した場合は、(1)として取り扱う。

9.1.4 疾病等の定義

有害事象のうち、疾病、障害若しくは死亡又は感染症に加え、臨床検査値の異常や諸症状で、特定臨床研究の実施に起因するものと疑われるものを「疾病等」とする。なお、臨床研究の実施に起因するものとは、研究において使用する医薬品等との因果関係があるもの又は研究の手順との因果関係があるものとする。因果関係は、9.2 研究との因果関係に基づいて、研究責任医師、分担医師、または代表医師が判断する。

9.2　研究との因果関係

被験者（母体、胎児）に生じた全ての有害事象及び児に生じた重篤な有害事象と研究との因果関係を、研究責任医師又は分担医師が判断する。判断には介入治療開始との時間的関係だけでなく、合併症、併用薬、研究手順、事故及びその他の外的因子などに起因することも考慮して判断する。因果関係は以下の基準に従って判断し、記録する。

- **因果関係有り又は否定できない** – 当該臨床研究又は介入治療によって発現することが既知・未知に関わらず、以下に従って判断する。
  - ➢ 研究又は介入治療に起因することが合理的である、または合理的な可能性がある
  - ➢ 研究との間に時間的関係がある
  - ➢ 他の原因が示せず、研究との因果関係が否定できない
- **因果関係無し** – 次の基準に従って判断する。
  - ➢ 研究又は介入治療に起因することが合理的でない
  - ➢ 時間的関係が示せない
  - ➢ その他の原因が示せる

9.3　重症度

研究責任医師又は分担医師は、被験者（母体、胎児）に生じた有害事象の重症度を以下の 3 段階で判定する。

軽度	一過性で日常生活を損なわず、治療を要しない程度
中等度	日常生活に多少の支障をきたし、十分な不快感を与え治療を要する程度
高度	日常生活の遂行に大きな支障があり、入院などの治療を要する程度

研究責任医師又は分担医師は、児に生じた有害事象の重症度は、新生児用 INC 版 v1.0 日本語訳（別添資料 1）で判定する。

9.4　予測性

有害事象の予測性は、添付文書及びインタビューフォームに基づいて判断する。有害事象の性質、重症度または頻度が一致しない場合、未知の有害事象とする。

9.5　収集期間及び追跡期間

プロトコル治療開始日を基点として、有害事象を収集・記録する。同意取得日に存在する臨床所見は合併症とし、有害事象とはしない。ただし、合併症の増悪は有害事象として記録する。原則として、研究との因果関係有りと判断された疾病等及び重篤な有害事象を、可能な限り回復または軽快まで追跡する。ただし、死亡・障害・後遺症など、それ以上の回復が困難または追跡不

能であると研究責任医師が判断した時点で追跡を終了とする。研究との因果関係の無い非重篤な有害事象は、転帰に関わらず、研究においては特に追跡を行わない。

有害事象の重篤性に関わらず、発現した有害事象は、被験者の研究参加終了まで収集・記録する。

9.6　有害事象の報告

被験者（母体、胎児）に生じた全ての有害事象について、事象名（診断名）発現日、重症度、因果関係、予測性、転帰、転帰の判断日を調査し、電子症例報告書（eCRF）に入力する。児については、重篤な有害事象について、事象名（診断名）、発現日、重症度、重篤性、因果関係、予測性、転帰（回復、軽快、未回復、後遺症あり、死亡、不明）、転帰の判断日を調査し、eCRF に入力する。被験者（母体、胎児）、児に生じた全ての有害事象のうち疾病等は、**9.6.2 疾病等の報告**に基づいて報告を行い、重篤な有害事象は、**9.6.1 重篤な有害事象の報告**に基づいて報告を行う。

9.6.1　重篤な有害事象の報告

被験者（母体、胎児）、児に重篤な有害事象が発現した場合、情報を知り得た研究分担医師は速やかに研究責任医師に報告する。研究責任医師は、重篤な有害事象について及び対応状況について速やかに把握し、必要な対応を検討する。因果関係の有無に関わらず、重篤な有害事象に関して、「08_統一書式準拠　医薬品の疾病等報告書」を作成し、研究代表医師及び研究事務局にメールにて報告する。研究代表医師及び研究事務局は、他の医療機関の研究責任医師に情報提供する。因果関係が否定できない場合、本項の手順に加えて、9.6.2 疾病等の報告に基づいて報告を行う。さらに、実施医療機関の規定・手順書に従って、実施医療機関の管理者等に報告するなど、必要な対応を行う。

研究代表医師　e−mail：okuchi@jichi.ac.jp
研究事務局　　e−mail：pprom.azm.jimu@gmail.com

9.6.2　疾病等の報告

重篤な疾病等（重篤な有害事象のうち因果関係が否定できないもの）が発生した場合、知り得た研究分担医師は、所属する機関の研究責任医師に報告する。報告を受けた研究責任医師は、当該機関の医療機関の管理者に報告するとともに、研究代表医師及び研究事務局に通知する。その後、疾病等の予測性に応じて、研究代表医師が知り得てから以下の報告期限までに、認定臨床研究審査委員会及び厚生労働大臣に報告する。

●未承認/適応外使用の提出期限

<table>
<tr><th></th><th>有害事象の種類</th><th>因果関係</th><th>予測性</th><th>委員会
報告期限</th><th>厚労大臣
報告期限</th></tr>
<tr><td rowspan="3">①</td><td rowspan="3">1 死亡または
死亡につながるおそれ</td><td rowspan="2">あり</td><td>できない</td><td>7日</td><td>7日</td></tr>
<tr><td>できる</td><td>15日</td><td rowspan="2">—</td></tr>
<tr><td>なし</td><td>—</td><td>—</td></tr>
<tr><td rowspan="3">②</td><td rowspan="3">2 治療のための入院又は入院期間の延長
3 障害・障害につながるおそれ
4 死亡又は 2～4 に準じて重篤
5 後世代における先天性の疾病等又は異常</td><td rowspan="2">あり</td><td>できない</td><td>15日</td><td>15日</td></tr>
<tr><td>できる</td><td rowspan="2">—</td><td rowspan="2">—</td></tr>
<tr><td>なし</td><td>—</td></tr>
</table>

認定臨床研究審査委員会への報告に当たっては、統一書式 8 を使用し、研究代表医師が他施設の医師等の協力を得て作成する。また、研究代表医師は、統一書式 8 の作成毎に、全ての実施医療機関の研究責任医師に当該報告書を送付して情報共有を行うとともに、必要に応じて注意喚起等の必要な対応を行う。さらに、研究代表医師を含む各研究責任医師は、情報共有された疾病等の発生について、それぞれの実施医療機関の管理者に報告するとともに、必要に応じて実施医療機関の管理者の協力を得ながら、疾病等の発生に係る予防策や対策を講じる。

厚生労働大臣への報告にあたっては、原則として疾病等報告システム（https://mh.cr-adr.com/adr/v1/）を使用して別紙様式２－1を作成し、医薬品医療機器総合機構（[redacted]）宛てに送付する。

臨床研究に関して発生した全ての疾病等について、年に1回実施する定期報告において認定臨床研究審査委員会及び実施医療機関の管理者に対し、疾病等の発生状況を報告する。

10　中止と終了

10.1　被験者の参加中止

以下の(1)～(3)に該当する理由により、被験者がプロトコル治療の中止のみならず、有効性及び安全性評価を含む全ての試験スケジュールを遵守できなくなった場合は「研究中止」となる。

プロトコル治療を開始する前に中止した場合は、観察を行わず、通常診療で被験者に不利益が生じないよう適切な治療を行う。

(1)　被験者が研究参加に対する同意を撤回した場合

(2)　死亡または死亡につながる恐れのある疾病等が発現した場合

(3)　その他に研究参加によるリスクが利益を上回ると研究責任医師が判断した場合

設定根拠

(1) 倫理的観点から設定した。

(2)～(3) 被験者の安全性の観点から設定した。

10.2 研究の中止

以下のような状況が発生し、研究責任医師、認定臨床研究審査委員会、実施医療機関の管理者が中止すべきと判断した場合、本研究全体を中止する場合がある。

（1） 予測できない重篤な疾病等が発生し、被験者（母体、胎児）全体への不利益が懸念される場合
（2） 法及び関連法令または研究計画書に対する重大な違反／不遵守が判明した場合
（3） 倫理的妥当性もしくは科学的合理性を損なう、又は損なう恐れのある事実を得た場合
（4） 被験者に対する重大なリスクが特定された場合
（5） 認定臨床研究審査委員会に意見を述べられた場合
（6） 厚生労働大臣に中止要請や勧告を受けた場合

中止の場合、研究責任医師は全ての実施医療機関の研究責任医師及び認定臨床研究審査委員会、実施医療機関の管理者に報告する。さらに、被験者と連絡をとり、研究スケジュールの変更について伝える。中止の場合、被験者は速やかにプロトコル治療を中止し、6.2.5 プロトコル治療の中止基準に従い、被験者（母体、胎児）の安全性を確保する。

10.3 研究終了

以下の事項が全て完了し、かつ、実施計画において研究終了を公表した日を研究終了とする。

・研究への被験者の登録終了と観察期間の終了
・総括報告書及びその概要書作成
・CRB の意見を聴く
・実施医療機関の管理者に提出
・厚生労働大臣に提出し、総括報告書の概要を公表
・実施医療機関の管理者へ報告

11 予測される利益・不利益及びリスクを最小化する方法

11.1 予測される利益

この研究に参加することにより、アンピシリンナトリウム（ABPC）/スルバクタムナトリウム（SBT）併用下で、エリスロマイシン(EM)またはアジスロマイシン（AZM）による治療を受けることができ、児の気管支肺異形成発症が予防できる可能性がある。この治療は、ABPC に変えて ABPC/SBT を用いており、より広範な細菌への治療が期待できること、投与期間が 7 日から 14 日になったことで、7 日間では根治できなかった細菌感染を治癒出来る可能性があることから、対照群、被験群のいずれの群においても、従来の方法では期待できなかった BPD に対する効

果をどちらも期待出来ると考えている。本邦での早産期前期破水に対する抗菌薬治療の確立につながる可能性がある。

11.2　予測される不利益

この研究では被験群または対照群に無作為に割り付けられるため、被験者が希望する治療を選択することはできない。どちらの群に割り付けられても、投与される抗菌薬の副作用（下痢など）が生じる可能性がある。エリスロシン点滴薬やジスロマック点滴薬を投与している間に血管痛が起こる可能性がある。

11.3　リスクを最小化する方法

本研究ではどちらの群に割り付けられた場合でも、それらの薬剤に起因する疾病等が起きた場合は、休薬または研究参加中止も含めて対応を個別に検討する。静脈採血、心電図測定、放射線画像撮影は、通常診療と同様の手技で実施するため、研究参加によるリスクが増大することはない。また、本研究における被験者の採血量は 10mL/回であり、医療上問題のない量である。なお、本研究における児の採血量も 1.0～1.5m L/回で医療上問題のない量であり、出生時体重 1000g 未満の場合は、血液検査を行わなくてもよいこととする。

12　倫理的事項及び要配慮事項

12.1　法令・指針の遵守

本研究は、試験薬が適応外使用に該当するため、臨床研究法（平成 29 年法律第 16 号）における特定臨床研究に該当する。そのため、本研究は臨床研究法を遵守し、また、ヘルシンキ宣言に基づく倫理的原則に則って実施する。

研究計画書と実施計画を含む、法で定められた資料は、認定臨床研究審査委員会での審議と承認を受ける。その後、各実施医療機関の研究責任医師がそれぞれの実施医療機関の管理者の許可を得て、jRCT への登録を完了してから研究を開始する。これらの資料等に変更がある場合も、同様の手順を踏んでから変更事項を施行する。

本研究の各研究責任医師は、研究開始前に、CREDITS(https://www.uhcta.com/uth/member/)に登録し、e ラーニングから、「倫理・行動規範コース」および「臨床研究実施コース」の受講を義務付けることとする。ただし、各施設の倫理委員会が指定する、「研究責任医師として必要な講習」を 1 年以内にすでに終了している場合は、その受講記録を提出することで、本試験遂行能力があると判断する。

12.2　認定臨床研究審査委員会

本研究は、以下の認定臨床研究審査委員会において審査意見業務を受ける。やむを得ない場合を除き、原則として研究期間全体を通して、審査意見業務を受ける委員会は変更しない。

名称： 自治医科大学中央臨床研究審査委員会
認定番号： CRB320006
所在地： 〒329-0498 栃木県下野市薬師寺 3311-1
連絡先： 0285-58-7637
電子メールアドレス：[redacted]

12.3　個人情報等の取り扱い

12.3.1　匿名化について

本研究で収集する被験者（母体、胎児）及び児の個人情報を含むデータは、電子症例報告書に個人情報を含めない情報を転記する。被験者識別コードはカルテ ID との規則性を有さない英数字の組み合わせで、研究共通のコードと施設コードに、症例番号3桁の英数字とする。また、被験者個人を識別するための匿名化対応表を作成し、保管する。

匿名化対応表は被験者の同意を得た機関のみが保有する。

本研究では、認定臨床研究審査委員会やこの研究のモニタリング担当者が患者の診療記録（カルテなど）を直接閲覧して確認することがあるが、情報漏洩に注意して実施する。

12.3.2　個人情報に関する被験者等の権利

各研究責任医師は、研究において保有する個人情報及び個人識別符号に関して、被験者等の求めに応じて個人情報の開示、利用目的の通知、訂正・追加・削除及び利用停止を行う。ただし、実施医療機関の権利、臨床研究の適正な実施、被験者本人の権利などを害するおそれがある場合などの正当な理由により求めに応じない場合は、被験者等に対して理由を説明する。

12.4　健康被害に対する補償

被験者（母体）に健康被害が生じた場合、保険診療の範囲内で適切な治療を行う。医療費の自己負担分は被験者の負担とする。本研究では既承認薬の保険適応外使用であるため、健康被害に対する補償を行うため、臨床研究保険に加入する。本研究との因果関係が否定できない健康被害が生じた場合、健康被害の程度と臨床研究保険の契約内容に応じて補償を行う。ただし、被験者に過失がある場合は対象とはならない。また、医師に過失がある場合は、医師賠償責任保険により賠償を行う。臨床研究保険は、初回の承認が得られた後に速やかに契約締結を行う。

12.5　研究に参加しない場合の治療方法

研究に参加しない場合、各実施医療機関で行われている抗菌薬治療（ペニシリンまたはセフェム系抗生剤の投与またはエリスロマイシンの併用投与）が行われる。研究に参加しない場合でも被験者に合った最善の治療を選択する。

12.6　研究終了後の医療の提供

妊娠終結後又はプロトコル治療終了後も子宮内感染に対しては、保険診療の範囲で、被験者の希望に沿った最善の治療を行う。

12.7　遺伝的特徴等に関する研究結果の取り扱い

本研究では被験者の遺伝学的特徴が得られるような検査・解析は行わないため該当しない。

12.8　被験者の経済的負担又は負担軽減費

本研究で使用する試験薬は、本研究の研究費で負担するため、被験者の経済的負担は生じない。それ以外は被験者の加入する健康保険及び被験者の自己負担により支払われる。また、食事代、差額ベッド代などの保険外の高額になると思われる費用に関しては、試験に参加しないpPROM 発生妊婦と扱い上違いはない。

本研究に参加することで、通院回数及び検査回数が増えることが予想されるため、通常診療に比べて被験者の経済的負担も大きくなる可能性がある。被験者の負担を軽減するため、負担軽減費 10,000 円を研究期間中に 1 回のみ、被験者に支払う。負担軽減費については同意説明文書であらかじめ説明し、被験者が指定する口座に、研究参加後に振り込む。可能な限り、退院前までに、口座番号等を記載する用紙に情報を記入していただき、返信用封筒に入れ、調整事務局へ送付していただく。振込手数料は自治医科大学経理課が負担する。なお、口座番号等は負担軽減費の支払い目的以外に利用されることはない。

13　研究資金・利益相反及び情報の公開

13.1　研究の資金源

本研究は、国立研究開発法人日本医療研究開発機構 研究開発費（成育疾患克服等総合研究事業—BIRTHDAY）「妊娠 28 週未満発症の早産期前期破水妊婦に対するアジスロマイシン投与による気管支肺異形成の予防法の開発」（研究代表医師：大口　昭英）及び自治医科大学から研究代表医師に配当される研究費により実施される。

13.2　利益相反の状況

研究全体に関する利益相反及び研究代表医師・研究責任医師・研究分担医師の個人の利益相反は、研究開始前に利益相反状況を申告し、各々の所属機関において事実確認を行う。研究全体に関する利益相反及び個人の利益相反は、研究開始前に利益相反状況を申告し、各々の所属機関において事実確認を行う。実施医療機関に利益相反状況の確認・助言を行う委員会等が設置されている場合は、当該委員会で事実確認を行う。委員会が設置されていない機関にお

いては、研究機関の長または実施医療機関の管理者に対して利益相反状況について申告したうえで、当該者が事実確認を行う。

利益相反状況の事実確認の結果をもって実施医療機関ごとに利益相反管理計画書を策定し、認定臨床研究審査委員会の承認を得たうえで利益相反の管理・公表を行う。研究成果の公表時も、同様に利益相反状況について公表する。研究対象者に対しても、利益相反状況について開示する。

研究開始後も適切に再申告と承認を得る。たとえば利益相反状態に変更が生じた場合、研究責任医師・研究分担医師を追加または変更する場合、定期報告を行う場合、その他適切なタイミングで利益相反状況・利益相反管理計画等について見直しを行い、再申告した上で再度認定臨床研究審査委員会の承認を得る。

13.3　情報公開の方法

本研究の研究概要及び研究結果は、厚生労働省が整備する臨床研究実施計画・研究概要公開システム（jRCT：https://jrct.niph.go.jp/）に登録し、厚生労働大臣に実施計画を提出することでjRCT において公表する。内容に変更が生じる場合は、jRCT を修正し、認定臨床研究審査委員会の承認を得て、変更を厚生労働大臣に届け出る。また、法に基づき、少なくとも 1 年ごとに情報の更新を行う。

13.4　結果の公表

研究の結果は、論文投稿及び学会発表などにより公表する。また、総括報告書の概要及び主要評価項目に関する結果は、厚生労働大臣に届け出るとともに、jRCT で公表する。総括報告書の概要は全ての収集期間が終了する観察期間終了後1年以内の 2025 年 3 月頃に厚生労働大臣に届け出る。ただし、jRCT での公表に関しては論文が掲載された後、2025 年内を目標として公表する。学術雑誌への投稿時の著者に関しては、筆頭著者は研究登録終了時点で最も症例登録数の多かった施設の研究責任医師あるいは研究分担医師とする。第 2 著者は、次に登録症例数が多かった施設の研究責任医師あるいは研究分担医師とする。各実施施設の産科医師 2 名、新生児科医師 2 名まで著者とする。第 3 著者以降の著者の順番は、登録者数の多い施設の順番とする。本研究でプロトコル作成に寄与した佐古まゆみ・小林徹・三好剛一は著者とする。研究代表医師の大口昭英、及び野見山亮は共同で責任著者とする。なお、登録期間中に 1 症例も登録がなかった施設については、著者ではなく、Acknowledgement に氏名を掲載する。

ウレアプラズマ属及びマイコプラズマ属の検出に関する主要論文については、研究代表医師の大口昭英、及び、研究責任医師の柳原格を責任著者とする。各施設の研究責任医師を著者とする（順番は名字のアルファベット順とする）。大口昭英は最終著者とする。また、大阪府立母子保健総合医療センター研究所において本研究に関与した研究者は著者とする事ができる。筆頭著者は柳原格が決定する。

本研究に参加した研究責任医師は、得られたデータを使い二次解析を行う事が出来る。ただし、二次解析を行うためには、研究代表医師の承認が必要である。論文には本研究に参加した研究代表医師、研究責任医師（少なくとも一人以上）を入れることにする。著者以外の各施設の研究責任医師、プロトコル作成に寄与した佐古まゆみ・小林徹・三好剛一については、謝辞に記

載する。二次解析に関与した研究者は、本研究に関与していなくても著者とすることができる。二次解析を申請した研究者は責任著者になり、筆頭著者を決定する。

14 統計学的事項

14.1 解析対象集団

解析対象集団は以下のとおりとする。尚、主要評価項目の解析対象集団は、"Full Analysis Set"とする。

1)Full Analysis Set (FAS)
無作為化が行った全ての被験者から、以下に該当する者を除外した被験者集団(被験者、胎児/児)
・プロトコル治療を一度も実施していない被験者
・プロトコル治療を 1 回以上実施したが、実施後のデータが得られていない被験者
・適格性基準を満たさない被験者

2)Per Protocol Set (PPS)
FAS のうち、プロトコル治療に関する逸脱及び併用禁止薬に関する逸脱が生じた被験者を除外した被験者集団(被験者、胎児/出生児)

3)安全性解析対象集団
プロトコル治療を 1 回以上実施した全ての被験者集団(被験者、胎児/児)

14.2 統計解析

14.2.1 被験者背景

被験者の背景情報は、FAS、PPS、安全性解析対象集団において要約する。離散型データに関しては、各群で頻度及び割合を算出し、連続型データに関しては、各群で要約統計量(平均値、標準偏差、最小値、中央値、最大値)を算出する。

14.2.2 主要評価項目の解析

主として FAS で解析を行い、副次的に PPS でも解析を行う。FAS を用いた解析において、主要評価項目が欠測している被験者は理由を問わずすべて「中等症以上の BPD_{36} または修正 36 週時までの児死亡発生あり」として扱う。PPS を用いた解析において、主要評価項目が欠測している被験者は解析から除外する。

「中等症以上の BPD_{36} または修正 36 週時までの児死亡発生の割合が群間で等しい」ことを帰無仮説、「中等症以上の BPD_{36} または修正 36 週時までの児死亡発生の割合が群間で異なる」ことを対立仮説とした両側検定とし、割合の差の検定で p 値を算出する。有意水準は 0.05 とする。

被験群と対照群のそれぞれで主要評価項目の割合とその 95%信頼区間を算出する。あわせて群間の割合の差とその 95%信頼区間を算出する。ここまでを主たる解析とする。また、妊娠週数（22 週 0 日～23 週 6 日、24 週 0 日～27 週 6 日）、施設（早期カフェイン使用施設、早期カフェイン未使用施設）を層別因子として調整した Mantel-Haenszel 法による調整済みリスク差とその 95%信頼区間を算出する。

14.2.3 副次評価項目の解析

主として FAS で解析を行い、副次的に PPS でも解析を行う。

1） 母体の有効性評価項目

(1) pPROM 発症 から分娩までの日数

目的変数を pPROM 発症から分娩までの日数、説明変数を群（被験群・対照群）とした Cox 比例ハザードモデルを用いて、対照群に対する被験群のハザード比とその 95%信頼区間を算出する。分娩以外のイベントはいずれも打ち切りとして扱う。あわせて、各群において Kaplan-Meier 曲線を作図する。また、妊娠週数（22 週 0 日～23 週 6 日、24 週 0 日～27 週 6 日）、施設（早期カフェイン使用施設、早期カフェイン未使用施設）を層別因子とした層別 Cox 比例ハザードモデルを用いて、対照群に対する被験群のハザード比とその 95%信頼区間を算出する。

(2) 分娩時の妊娠週数

分娩時の妊娠週数に関して、各群で要約統計量（平均値、標準偏差、最小値、中央値、最大値）を算出する。あわせて、各群において箱ひげ図を作図する。また、妊娠週数（22 週 0 日～23 週 6 日、24 週 0 日～27 週 6 日）、施設（早期カフェイン使用施設、早期カフェイン未使用施設）の層別因子ごとに各群で要約統計量、箱ひげ図を作成する。

(3) 妊娠終結理由

妊娠終結理由に関する一覧表を作成する。各群で妊娠終結理由別に頻度及び割合を算出する。また、妊娠週数（22 週 0 日～23 週 6 日、24 週 0 日～27 週 6 日）、施設（早期カフェイン使用施設、早期カフェイン未使用施設）の層別因子ごとに各群で妊娠終結理由別に頻度及び割合を算出する。

(4) 分娩所見（分娩様式、死産の有無、胎盤重量）

分娩様式（自然分娩、分娩誘発、帝王切開、吸引分娩、鉗子分娩、骨盤位牽出術）に関して、各群で部分娩様式別の頻度及び割合を算出する。また、妊娠週数（22 週 0 日～23 週 6 日、24 週 0 日～27 週 6 日）、施設（早期カフェイン使用施設、早期カフェイン未使用施設）の層別因子ごとに各群で分娩様式別に頻度及び割合を算出する。

死産の有無に関して、各群で頻度及び割合とその 95%信頼区間を算出する。あわせて、割合の差とその 95%信頼区間を算出する。また、妊娠週数（22 週 0 日～23 週 6 日、24 週 0 日～27 週 6 日）、施設（早期カフェイン使用施設、早期カフェイン未使用施設）を層別因子として調整した Mantel-Haenszel 法による調整済みリスク差とその 95%信頼区間を算出する。

胎盤重量に関して、各群で要約統計量（平均値、標準偏差、最小値、中央値、最大値）を算出する。あわせて、各群において箱ひげ図を作図する。また、妊娠週数（22 週 0 日～23 週 6 日、24 週 0 日～27 週 6 日）、施設（早期カフェイン使用施設、早期カフェイン未使用施設）の層別因子ごとに各群で要約統計量、箱ひげ図を作成する。

(5) 子宮内胎児死亡割合、組織学的絨毛膜羊膜炎割合、帝王切開割合、常位胎盤早期剥離割合

それぞれに関して、各群で頻度及び割合とその 95%信頼区間を算出する。あわせて、割合の差とその 95%信頼区間を算出する。また、妊娠週数（22 週 0 日～23 週 6 日、24 週 0 日～27 週 6 日）、施設（早期カフェイン使用施設、早期カフェイン未使用施設）を層別因子として調整した Mantel-Haenszel 法による調整済みリスク差とその 95%信頼区間を算出する。

2） 児の有効性評価項目

(1) BPD_{28}発生割合、周産期死亡（子宮内胎児死亡＋早期新生児死亡）割合、退院前死亡割合

それぞれに関して、各群で頻度及び割合とその 95%信頼区間を算出する。あわせて、割合の差とその 95%信頼区間を算出する。また、妊娠週数（22 週 0 日～23 週 6 日、24 週 0 日～27 週 6 日）、施設（早期カフェイン使用施設、早期カフェイン未使用施設）を層別因子として調整した Mantel-Haenszel 法による調整済みリスク差とその 95%信頼区間を算出する。

(2) 出生時体重、出生時身長、出生時頭囲

それぞれに関して、各群で要約統計量（平均値、標準偏差、最小値、中央値、最大値）を算出する。あわせて、各群において箱ひげ図を作図する。また、妊娠週数（22 週 0 日～23 週 6 日、24 週 0 日～27 週 6 日）、施設（早期カフェイン使用施設、早期カフェイン未使用施設）の層別因子ごとに各群で要約統計量、箱ひげ図を作成する。

(3) 入院日数（出生日～退院日）、侵襲的人工換気日数、非侵襲的人工換気日数、酸素投与された日数

入院日数（出生日～退院日）、侵襲的人工換気日数、非侵襲的人工換気日数、酸素投与された日数に関して、各群で要約統計量（平均値、標準偏差、最小値、中央値、最大値）を算出する。あわせて、各群において箱ひげ図を作図する。また、妊娠週数（22 週 0 日～23 週 6 日、24 週 0 日～27 週 6 日）、施設（早期カフェイン使用施設、早期カフェイン未使用施設）の層別因子ごとに各群で要約統計量、箱ひげ図を作成する。

(4) サーファクタント投与を要した児の割合、新生児呼吸窮迫症候群（RDS）発生割合、遷延性遷延性肺高血圧症発生割合、頭蓋内出血発生割合、脳室周囲白質軟化症（PVL）発生割合、敗血症発生割合、壊死性腸炎発生割合、症候性動脈管開存症発生割合

それぞれに関して、各群で頻度及び割合とその 95%信頼区間を算出する。あわせて、割合の差とその 95%信頼区間を算出する。また、妊娠週数（22 週 0 日～23 週 6 日、24 週 0 日～27 週 6 日）、施設（早期カフェイン使用施設、早期カフェイン未使用施設）を層別因子として調整した Mantel-Haenszel 法による調整済みリスク差とその 95%信頼区間を算出する。

3） その他の評価項目　ウレアプラズマ属感染割合（胎盤　児咽頭、児耳腔）

胎盤、児咽頭、児耳腔におけるウレアプラズマ属感染の有無に関して、各群で頻度及び割合とその 95%信頼区間を算出する。あわせて、割合の差とその 95%信頼区間を算出する。また、妊娠週数（22 週 0 日～23 週 6 日、24 週 0 日～27 週 6 日）、施設（早期カフェイン使用施設、早期カフェイン未使用施設）を層別因子として調整した Mantel-Haenszel 法による調整済みリスク差とその 95%信頼区間を算出する。

14.2.4　安全性評価項目の解析

安全性解析対象集団で解析を行う。有害事象及び副作用は MedDRA を用いてコーディングを行い、器官別大分類 (System Organ Class：SOC) 及び基本語 (Preferred Term：PT) 別に集計を行う。

1） 母体の安全性評価項目

(1) 有害事象及び副作用の発現状況

各群で有害事象及び副作用の発現件数・発現例数・発現割合を算出する。あわせて、発現割合の 95%信頼区間を算出する。

(2) 重篤な有害事象及び副作用の発現状況

各群で重篤な有害事象及び副作用の発現件数・発現例数・発現割合を算出する。あわせて、発現割合の 95%信頼区間を算出する。

(3) 重大な有害事象（胎児死亡、敗血症、intensive care unit (ICU)入室、多臓器不全、人工換気、子宮全摘術） の発現状況

各群で重大な有害事象及び副作用の発現件数・発現例数・発現割合を算出する。あわせて、発現割合の 95%信頼区間を算出する。

(4) その他の安全性評価項目

- バイタルサイン（体温、脈拍数、血圧）

各群で各観察時点の要約統計量（平均値、標準偏差、最小値、中央値、最大値）を算出する。あわせて、各群で平均値と標準偏差の推移に関するプロットを作図する。

- 血液学的検査、血液生化学検査

各群で各観察時点の要約統計量（平均値、標準偏差、最小値、中央値、最大値）を算出する。あわせて、各群で平均値と標準偏差の推移に関するプロットを作図する。

- 尿検査（蛋白、尿糖）

各群で各観察時点のカテゴリごとの頻度及び割合を算出する。

2） 胎児、出生児の安全性評価項目

(1) 重篤な有害事象及び副作用の発現状況

各群で重篤な有害事象及び副作用の発現件数・発現例数・発現割合を算出する。あわせて、発現割合の 95%信頼区間を算出する。

14.2.5　部分集団解析

必要に応じて、部分集団解析等の探索解析を行う。

14.2.6　中間解析計画

中間解析は行わない。

14.3　統計解析計画の変更

統計解析計画を変更する場合、その変更理由、変更が研究結果や全体に及ぼす影響、その影響の倫理的及び科学的妥当性について、研究代表医師、研究責任医師において慎重に検討を行う。検討の結果、変更が妥当であると判断された場合、主要評価項目及びその解析に関するあらゆる主要な変更及び変更の概要について、研究計画書に明記し、改訂を行う。当該変更の妥当性に関する検討の方法・内容・結果に関しては別途記録に残す。

改訂後の研究計画書は、認定臨床研究審査委員会の審査を受け、意見を聴いてから変更に至る。変更に関する詳細は、総括報告書においても説明する。

15　試料・情報の保管及び廃棄

15.1　保管方法・保管期間

15.1.1　試料の保存方法・保存期間

本研究で採取した中央測定のための検体は、被験者識別コード（研究用 ID）を付して中央測定施設に提出される。当該検体は、中央測定施設にて、施錠可能な研究所免疫部門研究室内の病原体保管用冷凍庫にて凍結保管する。保管期間は、研究期間終了までとする。被験者が同意撤回した場合はその時点で試料を廃棄する。

15.1.2　情報の保管方法・保管期間

研究期間中、診療記録以外の紙媒体の情報は、各施設の施錠可能なロッカーにて保管責任者が保管する。電子媒体の情報は、ファイルにパスワードをかけて、自治医科大学附属病院総合周産期母子医療センター母体・胎児集中治療管理部研究室の施錠可能なキャビネットにて保管する。

研究終了後、上記の紙媒体・電子媒体の資料と以下の資料を、自治医科大学附属病院総合周産期母子医療センター母体・胎児集中治療管理部研究室にて保管する。保管期間は研究終了後 10 年間とする。なお、自治医科大学以外の研究参加施設においては、保管期間は研究終了後 5 年間とする。

- 研究計画書、実施計画、試験薬概要書、原資料等
- 研究対象者に対する説明文書及び同意文書
- 主要評価項目報告書、総括報告書及びその概要
- 認定臨床研究審査委員会から受け取った審査結果の通知書等

・実施計画以外の厚生労働大臣への報告に関する写し
・モニタリングに関する文書
・特定臨床研究の実施に係る契約書
・医薬品等の管理に関する記録
・その他研究に関する重要な文書で、研究代表医師が指定したもの

15.2　廃棄方法

15.2.1　試料の廃棄方法

被験者より採取した中央測定以外の検体は、各施設にて必要な検査が完了した後にすぐ廃棄する。本研究で採取した中央測定のための検体は、被験者識別コード（研究用 ID）を付して中央測定施設にて、施錠可能な研究所免疫部門研究室内の病原体保管用冷凍庫にて凍結保管され、研究期間終了後に廃棄される。

15.2.2　情報の廃棄方法

廃棄の際には、特定の個人を識別することができないように匿名化等の適切な措置を行う。

15.3　試料・情報の新たな研究での利用

研究終了後、本研究で収集したデータは自治医科大学にて 10 年間保管する。保管される既存データを新たな研究に利用する場合は、新たな研究の研究計画書等を倫理審査委員会に付議し、承認されてから利用する。また、その際はオプトアウトの手続きにより情報公開文書を作成し、被験者が研究参加を拒否する機会を保障する。他施設の研究者に既存データを提供する場合は、インフォームド・コンセントの範囲で提供を行い、匿名化対応表は提供せず個人の識別ができないよう措置を行う。

16　品質管理及び品質保証

16.1　原資料

本研究における原資料は以下の通りとする。
母体および新生児の診療録、同意書、対応表、病理組織検査結果、eCRF 記録、大阪府立母子保健総合医療センター研究所における、腟・胎盤・新生児咽頭・耳腔で採取した試料に関する培養・PCR 結果、中央事務局や研究代表医師との相談記録等、重篤な有害事象報告書、試験薬管理表
全ての実施医療機関及び各研究責任医師は、臨床研究に関連するモニタリング及びに認定臨床研究審査委員会及び規制当局の調査の際に、原資料等の全ての臨床研究関連記録を直接閲覧に供する。

16.2　データマネジメント

本研究では、国立成育医療研究センター臨床研究センターがデータマネジメントを行い、電子症例報告書（eCRF）を用いデータ収集を行う。eCRF に入力されたデータで、データクリーニングによって確認された外れ値や異常値は、クエリとして eCRF 上で確認及び修正の依頼を行う。データセンターのデータ固定後に、解析責任者に対して固定データが提供される。詳細に関しては、データマネジメント計画書に規定する。

16.3　モニタリング

本研究では、国立成育医療研究センター臨床研究センターがモニタリングを行い、研究代表医師が指名したモニタリング担当者が実施する。詳細に関しては、モニタリング手順書に規定する。モニタリング担当者の氏名は、別途指名書において指名する。

16.4　監査

本研究では監査を行わない。

16.5　効果安全性評価委員会

本研究では、効果安全性評価委員会は設置しない。

16.6　BPD 診断評価委員会

本研究では、BPD 診断は、試験参加施設ではない施設から当該研究に直接関与しない医学専門家 3 名を選出し、BPD 診断評価委員会を設置し評価者盲検（PROBE 法）で実施する。BPD 診断評価委員会は、およそ 6 か月に 1 回程度開催する。詳細は、BPD 診断評価委員会に関する手順書に別途規定する。

17 法令に基づく報告及び情報共有等

17.1 報告先及び情報共有の範囲

各種報告、通知、情報共有等については、以下の通り実施する。

	委員会への審査依頼・報告・通知	厚生労働大臣への報告・提出	研究責任医師への情報提供	実施医療機関の管理者*1への報告・提出
実施計画（新規）	○	jRCT 登録<br>○	○*3	許可申請
厚生労働大臣への実施計画提出完了（公表完了）	○		○	○
実施計画（変更）	○	jRCT 修正<br>○*2	○*3	許可申請
9.6.2 疾病等報告	○	△*5	○	○
不適合	○*6		○	○
定期報告	○	○	○*3	○
主要評価項目報告書*7	○	○	○	○
主要評価項目報告書の公表完了			○	○
総括報告書	○		○	○
総括報告書の概要	○	○	○	○
研究計画書、説明同意文書（研究終了時）		○	○	○
研究中止	○	○	○	
委員会からの意見の内容			○	○
研究実施に関する苦情や告発*4			○	○

*1 実施医療機関の管理者への報告・提出は、研究代表医師及び各研究責任医師が、各自の機関で実施。
*2 変更後の実施計画と届出書（実施計画事項変更届出書または実施計画事項軽微変更届出書）を提出。
*3 委員会での審査結果を情報共有。
*4 苦情・告発により重大な不適合が確認された場合、重大な不適合に関する報告を実施。
*5「9.5.3 疾病等報告」に規定されている範囲の疾病等のみを厚生労働大臣へ報告。
*6 特に重大な不適合が発生した場合、認定臨床研究審査委員会に速やかに報告し意見を聴く。
*7 総括報告書の概要を提出し、主要評価項目報告書を作成しない場合は不要

17.2 実施計画の作成・変更等

研究開始前の新規審査にあたっては、研究代表医師は、実施計画を認定臨床研究審査委員会に提出し、承認を受ける。承認後、各実施医療機関の管理者の研究実施の承認を得てから jRCT へ登録し、実施計画を厚生労働大臣へ提出する。提出が受理され、jRCT 上で公開された場合、その旨を認定臨床研究審査委員会に通知し、実施医療機関の管理者に報告する。さらに、各研究責任医師に情報提供し研究責任医師は各自の実施医療機関の管理者に報告する。

実施計画の変更を行う際も、同様とするが、認定臨床研究審査委員会及び厚生労働大臣には、変更後の実施計画と一緒に、実施計画事項軽微変更届出書または実施計画事項変更届出書を提出する。

17.3 定期報告

研究に関する情報が jRCT で公表された日を基点として 1 年毎に定期報告を行う。jRCT 公表日から 1 年が経過するごとに2ヵ月以内に、研究代表医師が統一書式5定期報告書及び別紙様式3定期報告書を作成し、認定臨床研究審査委員会に提出する。委員会にて承認された場合、承認から 1 ヵ月以内に別紙様式3を厚生労働大臣に対して提出する。

研究代表医師は定期報告の内容及び委員会での承認・厚生労働大臣への提出に関して、研究責任医師に対して情報提供を行う。また、研究代表医師及び各研究責任医師は、各自の実施医療機関の管理者に対して、前述の定期報告書等を以って報告を行う。

17.4 不適合報告

臨床研究が本研究計画書又は法令等に適合していない状態であることが明らかとなった場合、不適合について知り得た研究分担医師は、研究責任医師に報告する。研究責任医師は速やかに実施医療機関の管理者に報告し、さらに研究代表医師にその旨を通知する。研究代表医師は、全ての研究責任医師に情報提供する。また、研究代表医師及び各研究責任医師は、各自の実施医療機関の管理者に報告する。研究代表医師及び不適合が発生した機関の研究責任医師は、再発防止策等を適切に検討する。

発生した不適合が、特に重大な不適合であると研究代表医師が判断した場合、研究代表医師は速やかに統一書式7重大な不適合報告書を作成し、認定臨床研究審査委員会に提出して意見を聴く。なお、重大な不適合とは、主に以下に示すものとする。

- 研究対象者の人権や安全性に影響を及ぼすもの
- 研究の進捗や結果の信頼性に影響を及ぼすもの
- 未承認の研究の実施
- 同意を得ないで臨床研究を実施した場合

なお、研究責任医師が不適合について然るべき報告先に報告しない可能性が懸念される場合は、研究分担医師が直接報告を行うことができる。

不適合のうち、研究対象者の緊急の危険を回避するため、その他医療上やむを得ない理由により研究計画書に従わなかったものに関しては、重篤な不適合には含まない。そのため、やむを得ず不適合となった場合は認定臨床研究審査委員会への報告は不要とするが、その場合であっても、不適合に関する記録は適切に作成する。

17.5 疾病等報告

疾病等報告は、**9.6.2 疾病等の報告**に従って、研究代表医師が認定臨床研究審査委員会及び厚生労働大臣へ報告する。

研究代表医師は、上記報告を行った旨及び疾病等の内容について、研究責任医師に情報提供を行う。さらに、研究代表医師及び各研究責任医師は、各自の実施医療機関の管理者に報告する。

17.6　研究の中止に関する報告

研究を中止した場合、研究代表医師は中止日より 10 日以内に、統一書式 11 中止通知書を作成し、認定臨床研究審査委員会に通知する。また、様式第四 特定臨床研究中止届書を作成し、厚生労働大臣に届け出る。さらに研究責任医師に情報提供を行い、なお、研究を中止した場合であっても、主要評価項目報告書、総括報告書及びその概要については適切に作成し、それらの作成と研究が終了するまでの間、定期報告及び疾病等報告を実施する。

17.7　研究終了及び報告書の作成に関する報告

研究代表医師は主要評価項目報告書を作成した場合、認定臨床研究審査委員会の意見を聴き、承認後速やかに jRCT に登録するとともに厚生労働大臣に提出し、公表する。また、報告書及び公表された旨について、研究責任医師に情報提供を行う。さらに、研究代表医師及び各研究責任医師は、各自の実施医療機関の管理者に主要評価項目報告書を提出する。

研究代表医師は総括報告書及び別紙様式1総括報告書の概要を作成した場合、同様に認定臨床研究審査委員会の意見を聴き、承認後 1 ヵ月以内に概要を jRCT に登録するとともに、厚生労働大臣に研究計画書、説明同意文書を提出し、公表する。また、研究代表医師は、研究責任医師に報告書及び公表について情報提供を行う。研究代表医師及び各研究責任医師は各自の実施医療機関の管理者に対して総括報告書及びその概要を提出するとともに公表の報告を行う。また、最終的な研究計画書を添付する。

総括報告書の概要について jRCT で公表された場合、研究代表医師は、統一書式 12 終了通知書を作成し、別紙様式1を添付して研究終了に関して認定臨床研究審査委員会に通知する。

17.8　その他

研究代表医師は、認定臨床研究審査委員会から意見を述べられた場合、その内容について研究責任医師に情報提供を行う。さらに、研究代表医師及び各研究責任医師は、各自の実施医療機関の管理者にその内容を報告する。その後、研究代表医師及び各研究責任医師は、述べられた意見に応じて研究の中止・中断・終了を含む適切な措置を検討する。中止の場合には、**10.2 研究の中止**並びに **17.6 研究の中止に関する報告**に従う。

研究に関して、苦情や告発を受けた場合、苦情・告発を受けた者は、速やかに研究責任医師に報告し、研究責任医師は実施医療機関の管理者に報告及び研究代表医師に通知する。研究代表医師は苦情・告発の内容を確認し必要な措置について検討を行うとともに、研究責任医師に情報提供を行う。研究代表医師及び各研究責任医師は、各自の実施医療機関の管理者に報告を行う。

18 研究体制

18.1 研究組織

〇代表機関及び研究代表医師
自治医科大学附属病院 総合周産期母子医療センター
母体・胎児集中治療管理部 教授 大口昭英

〇実施医療機関及び研究責任医師
別紙 1 参照

〇ウレアプラズマ属の培養・PCR 検査
大阪府立母子保健総合医療センター研究所 免疫部門 研究所免疫部門部長 柳原 格

〇調整事務局
自治医科大学附属病院 産婦人科 非常勤講師 高橋佳代

〇調整事務局の支援
国立成育医療研究センター 臨床研究センター 研究推進部門 研究推進部門長 佐古まゆみ

〇モニタリング責任者
国立成育医療研究センター 臨床研究センター データサイエンス部門 モニタリングユニット
ユニット長 佐古まゆみ

〇データマネジメント責任者
国立成育医療研究センター 臨床研究センター データサイエンス部門 データ管理ユニット
ユニット長 清家美和子

〇統計解析責任者
学校法人自治医科大学 情報センター 准教授 三重野牧子

〇統計解析業務
国立成育医療研究センター 臨床研究センター データサイエンス部門 生物統計ユニット
研究員 岩元晋太郎

〇BPD 診断評価委員会
国立成育医療研究センター 新生児科部長 諫山哲哉
北里大学医学部附属新世紀医療開発センター 先端医療領域開発部門 新生児集中治療学教授
中西秀彦
埼玉医科大学 医学部総合医療センター 小児科 准教授 難波文彦

18.2　相談窓口

被験者等からの相談・問い合わせは、実施医療機関ごとの相談窓口(別紙 2)で受け付ける。また、研究全体の相談・問い合わせは、以下の窓口にて受け付ける。

実施医療機関名	自治医科大学附属病院
所在地	〒329-0498　栃木県下野市薬師寺 3311-1
所属・役職	総合周産期母子医療センター母体・胎児集中治療管理部　教授
担当者氏名	大口昭英(不在時は、非常勤講師高橋佳代が担当する)
電話番号	0285-58-7376(産婦人科医局)<br>受付日時: 月曜日　14 時から 16 時まで<br>火曜日　9 時から 17 時まで<br>木曜日　14 時から 16 時まで
メールアドレス	[redacted]

18.3　業務委託

本研究では、国立成育医療研究センター臨床研究センターに、以下の業務を委託する。研究代表医師は、委受託契約書に基づいて委託業務の実施状況に関して定期的に報告を受け、必要に応じて指導を行うことにより監督する。

・研究事務局業務の支援業務
・モニタリング
・データマネジメント、eCRF の構築と運用
・統計解析

19　その他

19.1　略号一覧

略号	正式名称(英語)	正式名称(日本語)
ART	assisted reproductive technique	生殖補助医療
BiPAP	biphasic positive airway pressure	体外式気道陽圧
BPD	bronchopulmonary dysplasia	気管支肺異形成
CPAP	continuous positive airway pressure	持続陽圧呼吸法
CRO	Contract Research Organization	開発業務受託機関
eCRF	electrical Case Report Form	電子症例報告書
DPAP	directional positive airway pressure	呼気吸気変換方式持続陽圧
GIFT	gamete intrafallopian transfer	配偶子卵管内移植法
ICU	intensive care unit	集中治療室

IVF-ET	in vitro fertilization and embryo transfer	体外受精-胚移植
IVH	intraventricular hemorrhage	脳室内出血
MAS	meconium aspiration syndrome	胎便吸引症候群
MedDRA	Medical Dictionary for Regulatory Activities	国際医薬用語集
NCPAP	nasal continuous positive airway pressure	経鼻的持続陽圧
NDPAP	nasal-directional positive airway pressure	呼気吸気変換方式経鼻的持続陽圧
NEC	neonatal necrotizing enterocolitis	壊死性腸炎
NICHD	National Institute of Child Health & Human Development	アメリカ国立子ども人間発達研究所
NICU	neonatal intensive care unit	新生児集中治療室
(s)NIPPV	(synchronized) nasal intermittent positive pressure ventilation	(同期式)経鼻的間欠的陽圧換気
NIV-NAVA	non-invasive ventilation-neurally adjusted ventilatory assist	非侵襲的神経調節補助換気
PVL	periventricular leukomalacia	脳室周囲白質軟化症
pPROM	preterm premature rupture of the membranes	早産期前期破水
RDS	respiratory distress syndrome	新生児呼吸窮迫症候群
SOP	Standard Operating Procedure	標準業務手順書
TTN	transient tachypnea of the newborn	新生児一過性多呼吸

19.2 改訂履歴

版数	作成日	施行日	変更点	変更理由
第 1.0 版	2021 年 6 月 29 日	該当なし	—	研究計画書の作成
第 1.1 版	2021 年 7 月 30 日	該当なし	5. 対象集団<br>6. 介入<br>7. 研究方法及び手順<br>18. 研究体制	左記項目の手順等の見直し<br>研究体制の見直し<br>記載整備
第 2.0 版	2021 年 10 月 13 日	該当なし	5. 対象集団<br>6. 介入<br>7. 研究方法及び手順<br>9. 有害事象及び疾病等<br>11.3 リスクを最小化する方法	対象集団、リスクを最小化する方法の見直し<br>左記項目の手順等の見直し<br>記載整備

第 2.1 版	2021 年 12 月 28 日		2.1 背景<br>4.1　研究ザイン<br>5.1　選択基準<br>6.2　プロトコル治療<br>8. 同意取得方法<br>10. 中止と終了<br>11.1 予測される利益<br>12. 倫理的事項及び要配慮事項<br>14.3　統計解析計画の変更<br>15.3 試料・情報の新たな研究での利用<br>17.3 法令に基づく報告及び情報共有等	認定臨床研究審査委員会からの指摘により修正<br>誤記修正
第 2.2 版	2022 年 1 月 4 日		15.3　　試料・情報の新たな研究での利用	研究終了後の保存期間が 10 年であることを明記したため
第 2.3 版	2022 年 4 月 8 日		1.3 研究スケジュール<br>2.1 背景<br>5.1　選択基準<br>7.5 観察項目及び収集する情報・手順<br>8.1 インフォームド・コンセント<br>9.6.1　　重篤な有害事象の報告<br>12.1　法令・指針の遵守<br>12.8　被験者の経済的負担又は負担軽減費<br>13.4　結果の公表<br>15.1.2　情報の保管方法<br>16.2　データマネジメント<br>17.4　不適合報告	誤記修正<br>補足説明追加
第 2.4 版	2022 年 5 月 30 日		1.3 研究スケジュール<br>7.5 観察項目及び収集する情報・手順<br>7.6 実施期間及び登録期間<br>8.1　インフォームドコンセント	補足説明追加<br>jRCT 登録情報更新
第 2.5 版	2022 年 6 月 27 日		6.2　プロトコル治療	エリスロシン錠の服薬方法の変更
第 2.6 版	2022 年 10 月 25 日		1.1 概要<br>1.3 研究スケジュール<br>3 目的及び評価項目	BPD 診断評価委員会の設置に伴う変更<br>中央測定検体の保存方法、中央測定施

			7.5 観察項目及び収集する情報・手順　表 7.5-2 児のスケジュール<br>7.5.3 児の観察・調査項目及び実施時期<br>7.5.4 児における検査項目及び実施時期<br>7.5.5 中央測定検体の取り扱い<br>16.6　BPD 診断評価委員会<br>18.1 研究組織	設への検体送付方法の変更<br>誤記修正
第 2.7 版	2023 年 2 月 28 日		13.4 結果の公表	著者決定ルールの変更
第 2.8 版	2023 年 6 月 28 日		1. 1 概要<br>5. 1. 1 選択基準<br>5. 1. 2 除外基準<br>7.5 観察項目及び収集する情報・手順　表 7. 5 - 1 被験者（母体、胎児）のスケジュール、表 7.5-2 児のスケジュール<br>7. 5. 1 被験者（母体、胎児）の観察・調査項目及び実施時期<br>7. 5. 3 児の観察・調査項目及び実施時期<br>7. 5. 4 児における検査項目及び実施時期<br>18. 1 研究組織	除外基準の見直し<br>スクリーニング期の許容範囲の見直し<br>誤記修正<br>記載整備

20　引用文献

1. Jobe AH, Bancalari E. Bronchopulmonary dysplasia. Am J Respir Crit Care Med. 2001:163(7):1723-1729.
2. Sriram S, Schreiber MD, Msall ME, et al. Cognitive Development and Quality of Life Associated With BPD in 10-Year-Olds Born Preterm. Pediatrics. 2018:141(6): e20172719.
3. Hanke K, Hartz A, Manz M, et al. Preterm Prelabor Rupture of Membranes and Outcome of Very-Low-Birth-Weight Infants in the German Neonatal Network. Plos One. 2015 Apr 9;10(4):e0122564.
4. Suzuki Y, Horie K, Yada Y, et al. Vaginal Ureaplasma species increase chorioamnionitis in very preterm infants with preterm premature rupture of the membranes at < 28 weeks of gestation. Eur J Clin Meicrobiol Infect Dis. 2018:37(12):2371-2380.

5. Kenyon S, Boulvain M, Neilson JP. Antibiotics for preterm rupture of membranes. Cochrane Database Syst Rev 2013:12:CD001058.
6. 日本産科婦人科学会. 日本産科婦人科学会 産婦人科診療ガイドライン産科編（2020）. 日本産婦人科学会; 2020. 142-146p.
7. American College of Obstetricians and Gynecologists' Committee on Practice Bulletins No.217 (2020)
8. Royal College of Obstetricians and Gynaecologists Green-top Guideline No.73 (2019)
9. Society of Obstetricians and Gynaecologists of Canada Reaffirmed Guidelines (2017)
10. Hutzal CE, Boyle EM, Kenyon SL, et al. Use of antibiotics for the treatment of preterm parturition and prevention of neonatal morbidity: a metaanalysis. J Obstet Gynecol. 2008:199(6):620.e1-8.
11. Tanaka S, Tsumura K, Nakura Y, et al. New antibiotic regimen for preterm premature rupture of membrane reduces the incidence of bronchopulmonary dysplasia. J Obstet Gynaecol Res. 2019:45:967-973.
12. Gomez R, Romero R, Nien JK, et al. Antibiotic administration to patients with preterm premature rupture of membranes does not eradicate intra-amniotic infection. J Matern Fetal Neonatal Med. 2007:20:167-173.
13. Ramsey PS, Vaules MB, Vasdev GM, et al. Maternal and transplacental pharmacokinetics of azithromycin. Am J Obstet Gynecol. 2003:188:714-718.
14. Higgins RD, Jobe Ah, Koso-Thomas M, et al. Bronchopulmonary Dysplasia: Executive Summary of a Workshop. J Pediatr. 2018:197:300-308.
15. Isayama T, Lee SK, Yang J, et al. Revisiting the Definition of Bronchopulmonary Dysplasia: Effect of Changing Panoply of Respiratory Support for Preterm Neonates. JAMA Pediatr. 2017:171:271-279.
16. Jensen EA, Dysart K, Gantz MG, et al. The Diagnosis of Bronchopulmonary Dysplasia in Very Preterm Infants. An Evidence-based Approach. Am J Respir Crit Care Med. 2019:200:751-759.
17. Gagliardi L, Amador C, Puglia M, et al. Area-based study identifies risk factors associated with missed antenatal corticosteroid prophylaxis in women delivering preterm infants. Acta Paediatr. 2017:106：250-255.
18. Walsh MC, Wilson-Costello D, Zadell A, et al. Safety, reliability, and validity of a physiologic definition of bronchopulmonary dysplasia. J Perinatol. 2003;23(6):451-456.
19. Marc I, Piedboeuf B, Lacaze-Masmonteil T, et al. Effect of Maternal Docosahexaenoic Acid Supplementation on Bronchopulmonary Dysplasia-Free Survival in Breastfed Preterm Infants: A Randomized Clinical Trial. JAMA. 2020:324(2):157-167.
20. Kim SH, Chun J, Ko KH, et al. Effects of antenatal azithromycin for Ureaplasma spp. on neonatal outcomes. Pediatr Int. 2018: 61:58-62.
