# Supplementary material for: A phase II, multicenter, nonblinded, randomized controlled trial for evaluating protective effects of ABPC/SBT plus, azithromycin versus erythromycin, in pregnant women with pPROM occurring at <28 weeks of gestation on the development of BPD in neonates: Study protocol
Source: PLoS One. 2024 Jul 9;19(7):e0304705. doi: 10.1371/journal.pone.0304705 (PMC11232965; doi:10.1371/journal.pone.0304705)
Supplement: S2 Appendix — (PDF) [file pone.0304705.s003.pdf]

A phase II, multicenter, nonblinded, randomized controlled trial for evaluating protective effects of ABPC/SBT plus azithromycin versus erythromycin in pregnant women with pPROM occurring at <28 weeks of gestation on the development of BPD in neonates: Study protocol

○ REPRESENTATIVE ORGANIZATION
NAME OF MEDICAL INSTITUTION: JICHI MEDICAL UNIVERSITY HOSPITAL
PRINCIPAL INVESTIGATOR: DEPARTMENT OF MATERNAL AND FETAL INTENSIVE CARE MANAGEMENT, PERINATAL MEDICAL CENTER
AKIHIDE OHKUCHI

Edition number：2.9

Creation Date: November 29, 2023

Table of Contents

1 study summary 5
1.1 Overview 5
1.2 Schematic diagram 8
1.3 study schedule 10
2 Background 14
2.1 Background 14
2.1.1 Preterm premature rupture of fetal membrane (pPROM) occurring at <28 weeks of gestation and bronchopulmonary dysplasia (BPD) 14
2.1.2 Standard of care and problems of antimicrobial therapy for pPROM in Japan and abroad 15
2.1.3 Expected effects of ABPC/SBT + AZM 16
2.2 Significance of the study 18
3 Objective and evaluation Items 18
4 Study design 19
4.1 Study design 19
4.2 Randomization 21
4.3 Reasons of scientific rationality 21
5 Target group 22
5.1 Eligibility criteria 22
5.1.1 Selection criteria 22
5.1.2 Exclusion criteria 22
5.1.3 Reasons for setting 23
5.2 Target number of cases 24
5.2.1 Target number of cases 24
5.2.2 Reasons for setting the number of cases 24
6 Intervention 25
6.1 Drugs used 25
6.1.1 Summary of drugs 25
6.1.2 Predicted adverse effects 28
6.2 Protocol treatment 28
6.2.1 Dosage and administration 28
6.2.2 Rationale for establishing protocol treatment 29
6.2.3 Dose change 30
6.2.4 Interruption or withdrawal 30
6.2.5 Protocol treatment duration 30
6.2.6 Criteria for discontinuation of protocol treatmen 30
6.2.7 Packaging and labeling 31
6.2.8 Preparation and post-processing 31
6.3 Management method 32
6.3.1 Storage and delivery 32
6.3.2 Disposal/Return 32
6.4 Concomitant medications 32
6.4.1 Concomitant medications 32
6.4.2 Concomitant restricted drugs ans concomitant restricted treatments 32

6.4.3 Antimicrobial therapy for exacerbations of infection after completion of protocol treatment 33
6.4.4 Concomitant Prohibited treatment 33
7 study methods and procedures 34
7.1 Subject recruitment 34
7.2 Consent to participate 34
7.3 Screening 34
7.3.1 Practice item 34
7.3.2 Screening dropout 34
7.4 Subject enrollment 34
7.5 Observation items and information/procedures to be collected 36
7.5.1 Items to be observed and investigated in subjects (mother and fetus) and time of implementation 40
7.5.2 Test items and timing in subjects (mother and fetus) 41
7.5.3 Test to be observed and investigated in the children and the time of implementation 44
7.5.4 Test items and timing in the children 48
7.5.5 Handling of centrally measured specimens 49
7.6 Period of implementation and registration 49
7.7 End of enrollment and observation period 50
8 Method of obtaining consent 50
8.1 Informed consent 50
8.2 Informed assent for adult pregnant women over 18 years of age 50
8.3 Withdrawal of consent 51
9 Adverse events and “disease, etc.” 51
9.1 Definition 51
9.1.1 Definition of adverse events 51
9.1.2 Definition of critical adverse events 51
9.1.3 Definition of serious adverse events 51
9.1.4 Definition of “disease, etc.” 52
9.2 Causal relationship with study 52
9.3 Severity 52
9.4 Predictability 52
9.5 Collection and tracking period 52
9.6 Adverse event reports 53
9.6.1 Serious adverse event reports 53
9.6.2 Reporting of “disease, etc.” 53
10 Cancelation and completion 54
10.1 Cancelation of participation in the trial 54
10.2 Cancelation of the entire trial 55
10.3 Completion of the trial 55
11 Anticipated benefits and disadvantages, and methods to minimize risks 56
11.1 Anticipated benefits 56
11.2 Anticipated disadvantages 56
11.3 Methods to minimize risks 56
12 Ethical matters and matters requiring consideration 56

12.1 Compliance with laws and guidelines 56
12.2 Certified Clinical Research Review Board 57
12.3 Handling of personal information, etc. 57
12.3.1 Anonymization 57
12.3.2 Rights of subjects and others with respect to personal information 57
12.4 Compensation for health hazards 58
12.5 Method of treatment if not participating in the study 58
12.6 Provision of medical care after completion of the study 58
12.7 Handling of study results on genetic characteristics, etc. 58
12.8 Subjects' financial burden, and burden reduction expenses 58
13 Research funding, conflicts of interest, and disclosure of information 59
13.1 Sources of funding for this study 59
13.2 Conflicts of interest 59
13.3 Method of information disclosure 59
13.4 Publication of results 60
14 Statistics 60
14.1 Population analyzed 60
14.2 Statistical analysis 61
14.2.1 Subject background 61
14.2.2 Analysis of primary outcome 61
14.2.3 Analysis of secondary outcomes 62
14.2.4 Analysis of safety endpoints 64
14.2.5 Subpopulation analysis 64
14.2.6 Interim analysis plan 65
14.3 Change in statistical analysis plan 65
15 Storage and disposal of samples and information 65
15.1 Storage method/period 65
15.1.1 Storage method and period of samples 65
15.1.2 Storage method and period of information 65
15.2 Disposal method 66
15.2.1 Disposal methods for samples 66
15.2.2 Disposal methods for information 66
15.3 Use of samples and information in new researches 66
16 Quality control and quality assurance 66
16.1 Original data 66
16.2 Data management 67
16.3 Monitoring 67
16.4 Audit 67
16.5 Effectiveness and safety evaluation committee 67
16.6 BPD Diagnosis and Evaluation Committee 67
17 Reporting and information sharing based on laws and regulations, etc. 68
17.1 Scope of reporting and information sharing 68
17.2 Creation and modification, etc. of implementation plans 69
17.3 Periodic Reporting 69
17.4 Nonconformity report 69
17.5 Report of "disease, etc." 70

17.6 Reports of cancelation of the entire trial 70
17.7 Completion of the trial, and reporting of making the final reports 70
17.8 Other 71
18 Study system 71
18.1 Study organization 71
18.2 Consultation service 72
18.3 Outsourcing 73
19 Other 73
19.1 Abbreviation list 73
19.2 Revisoin history 74
20 References 76

1 STUDY SUMMARY

1.1 OVERVIEW

**Title**	**A phase II, multicenter, nonblinded, randomized controlled trial for evaluating protective effects of ABPC/SBT plus azithromycin versus erythromycin in pregnant women with pPROM occurring at <28 weeks of gestation on the development of BPD in neonates**
**Aims of the trial**	The aims of this trial are: (1) to investigate the effectiveness and safety of AZM and EM under the co-administration of ABPC/SBT to prevent BPD in infants who are delivered by pregnant women with pPROM occurring at <28 weeks of gestation, and to calculate the sample size requirements for a full-scale clinical trial powered at 80% to determine a clinically important difference in the occurrence rate of BPD between AZM and EM treatments, (2) to investigate the association between the detection of *Ureaplasma* spp. in the vagina, on the surface of the placenta, in the neonatal throat, and in the neonatal ear cavity and the occurrence of BPD in infants who are delivered by pregnant women with pPROM occurring at <28 weeks of gestation.
**Objective**	Primary objective：to investigate the effectiveness and safety of AZM and EM under the co-administration of ABPC/SBT to prevent BPD in infants who are delivered by pregnant women with pPROM occurring at <28 weeks of gestation, and to calculate the sample size requirements for a full-scale clinical trial powered at 80% to determine a clinically important difference in the occurrence rate of BPD between AZM and EM treatments.
	Secondary objective： to investigate the association between the detection of *Ureaplasma* spp. in the vagina, on the surface of the placenta, in the neonatal throat, and in the neonatal ear cavity and the occurrence of BPD in infants who are delivered by pregnant women with pPROM occurring at <28 weeks of gestation.
**Evaluation items**	Primary outcome：the composite outcome of "incidence rate of either moderate/severe BPD_{36} or IUFD, and infantile death at or less than 36 weeks 0 days PMA".
	Secondary outcomes (Efficacy evaluation items for mother)：<br>days from the onset of pPROM to delivery, gestational weeks at delivery, reasons for pregnancy termination, findings at delivery (mode of delivery, presence/absence of stillbirth, placental weight), incidence rate of IUFD, incidence rate of histological chorioamnionitis, rate of cesarean delivery, incidence rate of placental abruption.<br><br>Secondary outcomes (Efficacy evaluation items for infants):

	incidence rate of BPD_{28}, perinatal death rate (total of IUFD + early neonatal death / total of neonatal live infants), rate of infantile death until just before discharge to home of the infants, birth weight, birth height, birth head circumference, day of hospitalization (from the birth date to discharge to home), day of invasive ventilation, day of non-invasive ventilation, day of oxygen administration, incidence rate of infants with surfactant administration, incidence rate of infants with RDS, incidence rate of infants with pulmonary hypertension in the neonate, incidence rate of infants with IVH, incidence rate of infants with PVL, incidence rate of infants with sepsis, incidence rate of infants with NEC, incidence rate of infants with symptomatic patent ductus arteriosus.<br><br>Secondary outcomes (Safety evaluation iterms for mother and fetus):<br>incidence rates of adverse events or side effects, incidence rates of serious adverse events or serious side effects, incidence rates of critical adverse events (IUFD, sepsis of mother, admission to intensive care unit of mother, multiple organ disorders of mother, artificial ventilation for mother, hysterectomy for mother).<br><br>Secondary outcomes (Safety evaluation items for infants):<br>incidence rates of serious adverse events.<br><br>Secondary outcomes (Other evaluation items in the current trial):<br>detection rate of Ureaplasma spp. (in vagina, on surface of the placenta, in infantile pharynx, and in infantile ear cavity).
**Study design**	We will plan a Phase II multicenter, open-label, randomized, parallel-group trial to explore and compare the efficacy and safety of AZM and EM in combination with ABPC/SBT for the prevention of BPD in infants born to pregnant women with pPROM onset at <28 weeks of gestation. The BPD diagnosis will be judged by a BPD Diagnosis and Evaluation Committee consisting of three medical experts who are not directly involved in this study from facilities that are not participating in this study, and the evaluators will be blinded for the allocation of treatment (PROBE method).
**Subjects**	Inclusion criteria<br>1. Pregnant woman who gave consent to participate in the current trial.<br>2. Pregnant woman whose age at the time of receiving informed consent is equal to or over 18 years old.<br>3. Pregnant woman with a singleton pregnancy who developed pPROM at 22 weeks 0 days - 27 weeks 6 days.<br><br>Exclusion criteria<br>1. Pregnant woman with placenta previa.<br>2. Pregnant woman with trachelectomy; however, pregnant woman with cervical incision should not be excluded, because they are at high risk for the occurrence of pPROM.<br>3. Pregnant woman with hypertensive disorders of pregnancy; however, pregnant woman with chronic hypertension should not be excluded.

	4. Pregnant woman with a fetus who shows either absent end-diastolic velocity or reverse end-diastolic velocity before the onset of pPROM.<br>5. Pregnant woman with any malignant tumors for whom the attending physicians consider the termination of pregnancy at or soon after the onset of pPROM.<br>6. Pregnant woman with pPROM for whom the attending physicians consider the imminent termination of pregnancy at or soon after the onset of pPROM.<br>7. Pregnant woman with pPROM who has had diseases or symptoms causing attending physicians to consider the imminent termination of pregnancy at or soon after the onset of pPROM.<br>8. Pregnant woman with the administration of AZM due to Chlamydia infection within 2 weeks before the onset of pPROM.<br>9. Pregnant woman receiving any kinds of antibiotics other than either penicillin or cephem antibiotics for two weeks before the onset of pPROM.<br>10. Pregnant woman who has been administered antibiotics rather than either ABPC or ABPC/SBT, from the onset of pPROM to the time obtaining consent; However, the following pregnant woman should not be excluded: when the administration of antibiotics rather than either ABPC or ABPC/SBT (excluding any kinds of macrolide antibiotics) were performed one day before the time of obtaining consent or earlier and the administration period of them were within 3 days.<br>11. Pregnant woman with the administration of any kinds of macrolide antibiotics, from the onset of pPROM to the time obtaining consent.<br>12. Pregnant woman with allergy for either penicillin or macrolide antibiotics.<br>13. Pregnant woman complicated with infectious mononucleosis.<br>14. Pregnant woman with a fetus with either chromosomal abnormalities or suspected severe malformation syndrome.<br>15. Pregnant woman with a creatinine level of ≥1.1 mg/dL at the screening test just after obtaining consent.<br>16. Pregnant woman complicated with QT syndrome.<br>17. Pregnant woman with any types of psychiatric disorders for whom the attending physicians think that the patient should not participate in the current trial.<br>18. Pregnant woman for whom the attending physicians think that the patient should not participate in the current trial, because there are some factors that may hamper the achievement of the trial.
**Intervention**	Intervention group (AZM administration group)<br>1. ABPC/SBT (Sulbacillin®) 1.5 g intravenous drip (div.) 4x/day 14 days (from Days 1 to 14).<br>2. AZM (Zithromax®) 500 mg div. 1x/day 4 days (on Days 1, 2, 8, and 9).<br>3. AZM (Zithromax®) 250 mg tablets orally 1x/day 10 days (from Days 3 to 7, and from Days 10 to 14).

	Control group (standard antibiotic administration group, EM administration group)<br>1. ABPC/SBT (Sulbacillin®) 1.5 g div. 4x/day 14 days (from Days 1 to 14).<br>2. EM (Erythrocin®) 500 mg div. 2x/day 4 days (on Days 1, 2, 8, and 9).<br>3. EM (Erythrocin®) 200 mg tablets orally 4x/day 10 days (from Days 3 to 7, and from Days 10 to 14).
**Target number of cases**	Total 100 cases (test group: 50 cases, control group: 50 cases)
**Study Period**	Planned implementation period: jRCT registration date – March 31, 2025<br>Expected registration period: April 1, 2022 – March 31, 2024<br>Planned observation period: April 1, 2022 – August 31, 2024
**Participation Period**	Up to 5 months from obtaining consent to last visit
**Combination Probihibed**	<Intervention group><br>（1）Macrolide preparations other than azithromycin<br><Control group><br>（1）Macrolide preparations other than erythromycin<br>（2）Ergotamine (ergotamine tartrate, dihydroergotamine mesylate) containing preparations [Cleamin®, Digidelgot®, etc.]<br>（3）Pimozide [Orap®]<br>（4）Asunaprevir [Sunbepra®]
**Study system**	○ Principal Investigator and Representative Institute<br>Akihide Ohkuchi, Professor, Department of Maternal and Fetal Intensive Care Unit, Perinatal Medical Center, Jichi Medical University Hospital<br>○ Study Secretariat<br>Kayo Takahashi, Part-time Lecturer, Department of Obstetrics and Gynecology, Jichi Medical University Hospital

1.2 SCHEMATIC DIAGRAM

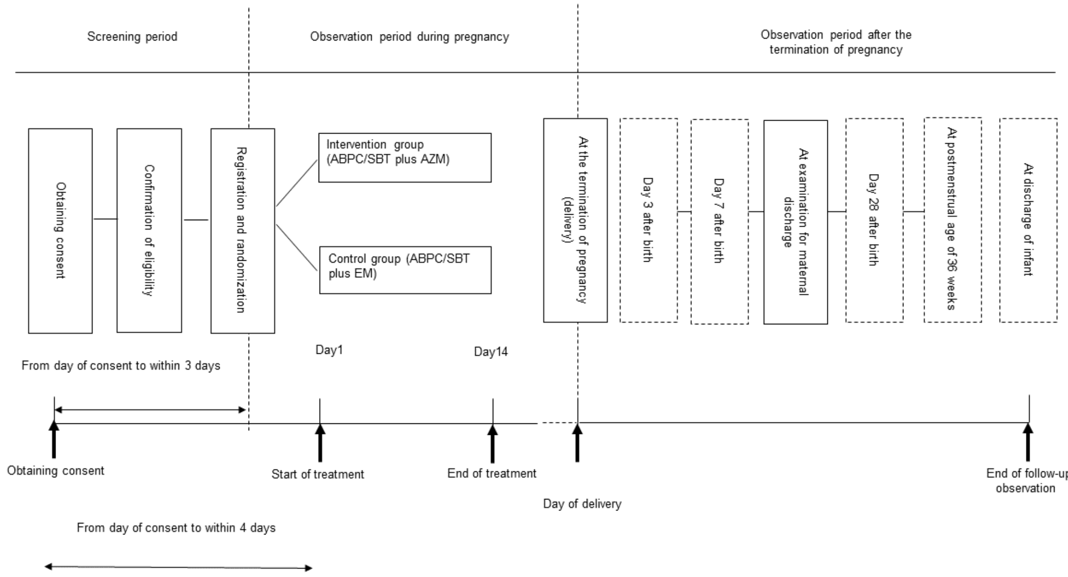

Schedule of enrollment intervention and assessments. CI or SIs must obtain written consent by the screening test. The maximum screening period (from consent acquisition to registration / randomization) is 3 days. CI or SIs must start protocol treatment within 4 days of obtaining consent. Predetermined observations, investigations, and tests must be performed prior to administration of the study drug. If the decision to terminate the pregnancy is made before the protocol treatment is completed, the protocol treatment must be terminated at that point, and the patient shifts to the post-pregnancy observation period after delivery.

1.3 STUDY SCHEDULE

The study will be conducted according to the following table

<Table 1.3-1 Schedule of subjects (mother and fetus)>

Table 1.3-1 Schedule of data collection and monitoring for participant (mother and fetus)

Period	Screening period[a]		Observation period during pregnancy[b]								Observation period after the termination of pregnancy		At the cancelation of the participation in the trial[d]	Follow-up observation after the cancelation of participation in the trial[e]
Working item	Screening period Days -3 to 0	Registration on Day 0	Week 1 Day 1	Week 2 Day 8 ± 2 day2	Week 3 Day 15 ± 2 days	Week 4 Day 22 ± 2 days	Week 5 Day 29 ± 2 days	Week 6 Day 36 ± 2 days	Week 7 Day 43 ± 2 days	Week 8〜[c] Day 50 ± 2 days	At the termination of pregnancy (delivery) ± 1 day	At examination for maternal discharge ± 2 days		
Obtaining consent[f]	X													
Participant: background information · body weight	X[o]													
Registration · randomization		X												
Care in hospital[g]			X	X	X	X	X	X	X	X	X	X		
Protocol treatment[h]			X	X										
Physical findings[i]	X		X	X	X	X	X	X	X	X	X	X	X	
Vital signs (blood pressure · pulse · body temperature)[i]	X		X	X	X	X	X	X	X	X	X	X	X	
Hematological examination[i]	X[p]			X	X	X	X	X	X	X	X	X	X	
Blood biochemical test[i]	X[p]			X	X	X	X	X	X	X	X	X	X	
Urinalysis (Qualitative: protein · sugar)[i]	X[p]			X	X	X	X	X	X	X	X	X	X	
ECG	X[p]													

Culture of vaginal microorganisms (each institute)[i]			X[r]	X[s]	X	X	X	X	X	X	X			
Culture of vaginal *Ureaplasma* spp.[j]			X[r]	X[s]										
PCR for vaginal *Ureaplasma* spp.[j]			X[r]	X[s]										
Culture of *Ureaplasma* spp. on the surface of the placenta[j]											X			
PCR for *Ureaplasma* spp. on the surface of the placenta[j]											X			
Histological examination for placenta											X			
Estimated fetal weight[i]	X[q]			X	X	X	X	X	X	X				
Fetal ultrasonography (Doppler · amniotic fluid volume estimation)[i]	X[q]			X	X	X	X	X	X	X				
Confirmation of drug used concomitantly[k]	X		X	X	X	X	X	X	X	X	X	X	X	X
Confirmation of adverse events[l]			X	X	X	X	X	X	X	X	X	X	X	X
Survey for the reasons of the termination of pregnancy[m]											X			
Findings on delivery[n]											X			

a. The maximum length of screening period is 3 days.

b. The planned interview, physical examination, ultrasonography, and sampling of biological specimens should be performed before the administration of test drugs. If the chief investigator (CI) or sub-investigator (SI) decides to terminate the pregnancy before the completion of protocol treatment, he/she should finish protocol treatment at the time of the decision, and will transfer to the observation period after the termination of pregnancy.

c. After week 8, CI or SI should perform the planned interview, physical examination, ultrasonography, and sampling of biological specimens once a week until the day of the termination of pregnancy.

d. CI or SI should perform the physical examination, sampling of biological specimens, and confirmation of drugs used concomitantly and adverse events within 3 days after the cancelation of participation in the trial.

e. CI or SI should perform follow-up observation after the cancelation of participation in the trial at the one-month postpartum medical examination.

f. CI or SI must obtain consent before starting examination during the screening period.

g. CI or SI should care for patients in hospital at least for the period from the start of protocol treatment to 3 days after delivery.

h. CI or SI must start protocol treatment within 4 days after obtaining consent.
i. CI or SI should examine the estimated fetal weight once a week at or after Week 3, until the decision to terminate the pregnancy. However, the hematological examination and blood biochemical test should be performed only when needed at or after Week 4, until the decision to terminate the pregnancy.
j. CI or SI should not take biological specimens from patients if the patients become complicated with COVID-19 infection. In addition, all preserved specimens should be abandoned.
k. If CI or SI decides to cancel participation in the trial due to the adverse events, CI or SI should continue the investigation of adverse events due to the test drugs or drugs used concomitantly which are the reasons for the cancelation of participation in the trial, from the cancelation to the one-month postpartum medical examination.
l. CI or SI should investigate the occurrence of adverse events in the subject (mother and fetus). As for the mother, CI or SI should investigate from the starting day of protocol treatment to the observation period after the termination of pregnancy (or day of cancelation of participation in the trial). As for the fetus, CI or SI should investigate from the starting day of protocol treatment to the termination of pregnancy (or day of cancelation of participation in the trial). However, if CI or SI decides to cancel participation in the trial due to the appearance of adverse events, CI or SI should continue the investigation of adverse events due to the test drugs or drugs used concomitantly which are the reasons for the cancelation of participation in the trial, from the cancelation to the one-month postpartum medical examination.
m. CI or SI should investigate the decision day of the termination of pregnancy, the time (day, hour, minute) of delivery, the reasons for termination of the pregnancy (both maternal and fetal causes).
n. CI or SI should investigate the delivery mode, presence/absence of stillbirth and the day of stillbirth, placental weight, and presence/absence of placental infarction.
o. CI or SI can use the data for body weight which is measured and recorded appropriately within 14 days before obtaining consent instead of body weight during screening period.
p. CI or SI can use the data for hematological examination, blood biochemical test, urinalysis, and ECG, which are measured and recorded appropriately within 3 days before obtaining consent instead of those during screening period.
q. CI or SI can use the data for estimated fetal weight, and fetal ultrasound (amniotic fluid volume estimation), which are measured and recorded appropriately within 7 days before obtaining consent instead of those during screening period.
r. The sampling of vaginal flora should be performed before the starting of protocol treatment. It is possible to perform it during the screening period.
s. CI or SI should perform the sampling of vaginal flora for the culture of *Ureaplasma* spp. or PCR of *Ureaplasma* spp. on Day 8 ± 2 days. However, if CI or SI finishs the protocol treatment before Day 8, CI or SI should not perform these samplings.

<Table 1.3-2 Schedule of children?

Table 1.3-2 Schedule of data collection and monitoring for participant (infant)

Working item	At the termination of pregnancy (delivery)	Day 3 after birth (± 1 day)	Day 7 after birth (± 3 days)	Day 28 after birth† (± 2 days)	postmenstrual age of 36 weeks# (± 2 days)	At discharge of infant (± 7 days)	At the cancelation of participation in the trial[a]
Umbilical blood gas analysis	X						
Neonatal findings[b]	X						
Apgar scores[c]	X						
Blood gas analysis[d]	X	X [k]					
Hematological examination[d]	X [i]	X [k]					
Blood biochemical test[d]	X [i, j]	X [k]					
Culture of *Ureaplasma* spp. in throat[e]	X						
PCR for *Ureaplasma* spp. in throat[e]	X						
Culture of *Ureaplasma* spp. in ear cavity[e]	X						
PCR for *Ureaplasma* spp. in ear cavity[e]	X						
Chest X-ray	X		X [l]	X	X[l]		

Total admission days	X	X	X	X	X	X	
Presence/absence, kinds, and duration of respiratory management	X	X	X	X	X	X	
Presence/absence of BPD_{36}					X[m]		
Presence/absence of BPD_{28}				X			
Neonatal diseases[f]	X	X	X	X	X	X	
Treatment for infant[g]	X	X	X	X	X	X	
Serious adverse events[h]	X	X	X	X	X	X	X

† Chief investigator (CI) or sub-investigator (SI) should perform data collection and monitoring at the one-month medical checkup, when the infant discharges within 28 days after birth.

# As for infants with birth at gestational ages <32 weeks PMA, the assessment of BPD_{36} should be performed at 36 weeks PMA or discharge to home, whichever comes first. As for infants with birth at gestational ages ≥32 weeks PMA, the assessment of BPD_{36} should be performed at ≥28 and <56 days postnatal age or discharge to home, whichever comes first.

a. CI or SI should investigate serious adverse events of the infant within 3 days after the cancelation of participation in this trial.

b. CI or SI should investigate the neonatal sex, birth weight (g), birth height (cm), head circumference at birth (cm), and presence/absence of small for dates, light for dates, large for dates, and heavy for dates.

c. The Apgar score should be recorded at 1 and 5 minutes after delivery.

d. The sampling of blood for the blood gas test is acceptable from either artery, vein, or capillary vessels.

e. CI or SI should not take biological specimens from the infant if the mother becomes complicated with COVID-19 infection.

f. CI or SI should investigate the presence/absence of intracranial hemorrhage (ICH), periventricular leukomalacia (PVL), respiratory distress syndrome (RDS), meconium aspiration syndrome (MAS), transient tachypnea in newborns (TTN), prolonged pulmonary hypertension (PPH), sepsis, necrotizing enterocolitis (NEC), local intestinal perforation, symptomatic patent ductus arteriosus (PDA), subglottic stenosis, laryngomalacia, .and other upper airway diseases. CI or SI should also investigate the presence/absence of surgery for the infant. If the infant has any surgeries, CI or SI should investigate the surgical procedure, day of surgery, aim of surgery, and execution of intubation for the surgery.

g. CI or SI should investigate oxygen administration, intravenous/intratracheal administration of steroid, nitrogen monoxide inhalation, surfactant administration, caffeine administration, and azithromycin (AZM) treatment.

h. CI or SI should investigate serious adverse events of infants from the termination of pregnancy to discharge of the infant (or day of cancelation of participation in the trial for the mother).

i. The hematological examination and blood biochemical test at the termination of pregnancy for the infant are possible using cord blood.

j. The examination of IgM is performed only at delivery.

k. The blood gas test, hematological examination, and blood biochemical test should not be performed if the infant's birth weight is under 1,000 g.

l. Chest X-ray at 7 and 28 days after birth should be performed when needed.

m. As for an infant delivered at or after 32 weeks of gestation, the evaluation of BPD_{36} should be performed at 56 days after delivery or at discharge to home, whichever comes first.

2 BACKGROUND

2.1 BACKGROUND

2.1.1 PRETERM PREMATURE RUPTURE OF FETAL MEMBRANE (PPROM) OCCURRING AT <28 WEEKS OF GESTATION AND BRONCHOPULMONARY DYSPLASIA (BPD)

Preterm premature rupture of the membranes (pPROM) is defined as PROM occurring at <37 weeks of gestation.

In Japan, pPROM accounted for 28.7% and 28.1% of very preterm births of less than 28 and 32 weeks of gestation, respectively. Therefore, it is estimated that about 700 children are born very preterm each year as a result of pPROM and about 1,700 children are born extremely preterm each year.

There are many kinds of neonatal complications in infants born to women with pPROM: pulmonary hypoplasia due to oligohydramnios, bronchopulmonary dysplasia (BPD), dry lung syndrome, amniotic band syndrome, neonatal asphyxia, persistent pulmonary hypertension in the neonate (PPHN), intraventricular hemorrhage (IVH), acute inflammatory disease due to amniotic infection such as sepsis, pneumonia, and meningitis, fetal inflammatory response syndrome such as chronic lung disease, necrotizing enterocolitis (NEC), periventricular leukomalacia (PVL), and long-term neurodevelopment impairments.

BPD is defined as respiratory distress symptoms that require oxygenation persisting beyond day 28, after neonatal respiratory compromise has abated or beginning soon after the neonatal respiratory compromise[1)]. The severity of BPD is classified by National Institute of Child Health and Human Development (NICHD) as mild, moderate, or severe depending on the infant's oxygen use at postmenstrual age (PMA) of 36 weeks, the oxygen concentration administered, and whether positive pressure ventilation.[1)] BPD is a frequent complication that occurs in approximately 50% of very low birth weight infants. The earlier the week of gestation, the higher the rate of BPD complications. Recent analyses using the Neonatal Research Network database Japan (http://plaza.umin.ac.jp/nrndata/) reported that the incidence of BPD_{36} in surviving discharged children delivered at 22, 23, 24, 25, 26, 27, 28, 29, and 30 weeks of gestation was 76, 72, 67, 58, 50, 41, 32, 24, and 17%, respectively. Because these data included infants not complicated with pPROM, we estimate that the incidence of BPD_{36} in infants with pPROM may be higher.

Some infants with moderate/severe BPD_{36} show continued respiratory failure beyond the neonatal period, and they need long-term oxygen and/or respiratory support. After school age, central nervous system effects are also seen, including decreased brain capacity and lower IQ in cognitive and language development[2)]. Although much research and treatment has been done, the incidence is still high, around 50%, and has a significant impact on the prognosis of very low birth weight infants.

It has been reported that pPROM is an independent risk factor for BPD[3)]. We analyzed 55 pregnant women with singleton pregnancy who had pPROM at <28 weeks of gestation, and delivered between 22 and 31 weeks of gestation, and we investigated the association between pPROM and the subsequent occurrence of short- and long-term prognoses; perinatal death occurred in 13% (intrauterine death: 6%, early neonatal death: 7%), pulmonary hypoplasia in 14%, respiratory distress syndrome (RDS) in 56%, transient tachypnea of newborn (TTN) in 42%, NEC

in 6%, IVH in 10%, PVL in 10%, sepsis in 4%, BPD at 28 days of life (BPD_{28}) in 72%, and BPD_{36} in 60% [4].

Since supportive care such as oxygen therapy is the only treatment for BPD, the prevention of BPD is strongly desired. To this end, it is very important for women with pPROM occurring at <28 weeks of gestation to receive appropriate antibiotics, and to prolong the pregnancy period.

2.1.2 STANDARD OF CARE AND PROBLEMS OF ANTIMICROBIAL THERAPY FOR PPROM IN JAPAN AND ABROAD

According to the Guideline for Obstetrical Practice in Japan 2020[6], "If the pregnancy is less than 26 weeks, the policy should be decided in consultation with a pediatrician, taking into consideration the presence of clinical chorioamnionitis, estimated weight, gestational age, and the facility's ability to handle low birthweight infants (Recommendation Level B)" and "If the pregnancy is 26 weeks to 34 weeks, the patient should wait under antimicrobial In principle, the patient should be kept on antibiotics, but early delivery may be considered depending on the facility's ability to handle the baby (recommendation level C)," but the details of recommended antimicrobial agents are not given. In addition, there are no antimicrobial agents approved in Japan for the indication of pPROM.

The basic management principle in the US, UK, and Canadian society guidelines are generally the same: waiting termination of pregnancy under antimicrobial therapy for prolonged gestation. The recommended antimicrobial agents are the combination of ampicillin (ABPC)/amoxicillin (AMPC) and erythromycin (EM) in the US, EM alone in the UK EM, and either the combination or EM alone in Canada. In a meta-analysis of randomized controlled trials of maternal antimicrobial therapy for pPROM <37 weeks of gestation[5], antimicrobial therapy significantly reduced chorioamnionitis and the number of newborns born within 48 hours and 7 days after pPROM. There was no difference in neonatal mortality, but it reduced neonatal infections, surfactant therapy, and the need for oxygenation.

Although azithromycin (AZM) is not currently the standard of care, the U.S. guideline[7] lists AZM as a candidate for first-line treatment as an alternative when EM is not available.

(Table 2-1) Standard treatment guidelines for pPROM in Japan and abroad (treatments recommended in the guidelines)

Country	Guidelines	Basic Management Principal	Recommended Antimicrobials
Japan	Japan Society of Obstetrics and Gynecology, Guideline for Obstetrical Practice in Japan 2020[6]	If the pregnancy is less than 26 weeks, consult with the pediatrician to determine the policy (B)<br>In principle, the patient should be kept under antimicrobial therapy from 26 to 34 weeks (C).	None stated
America	ACOG Practice Bulletins No. 217 (2020)[7]	Antimicrobial therapy is considered if the pregnancy is less than 24 weeks.<br>Waiting termination of pregnancy under antimicrobial therapy is recommended for	ABPC 2g intravenous drip (div.) + EM 250mg div. every 6 hours x 2 days →AMPC 500 mg oral + EM 333 mg oral

		pregnancies after 24 weeks but before 34 weeks.	every 8 hours x 5 days
United Kingdom	RCOG Green-top Guideline No. 73 (2019)[8)]	After 24 weeks of gestation, antimicrobial therapy is recommended for 10 days or until onset of labor (Grade A).	EM 250 mg every 6 hours x 10 days or until onset of labor
Canada	SOGC Reaffirmed Guidelines (2017)[9)]	Antimicrobial therapy is considered for pregnancies of less than 32 weeks unless labor has started (I-A)<br>After 32 weeks of gestation, antimicrobial therapy is recommended if lung maturity has not yet been achieved (I-A).	ABPC 2g div. + EM 250mg div. every 6 hours x 2 days →AMPC 500 mg oral + EM 333 mg oral every 8 hours x 5 days<br>EM 250 mg orally every 6 hours x 10 days

Although maternal antimicrobial therapy for pPROM has been shown to reduce the need for surfactant therapy and oxygenation in the child, it is not clear whether it reduces BPD development itself.

In a meta-analysis of randomized controlled trials of maternal antimicrobial therapy for pPROM <34 weeks, antimicrobials [ABPC, AMPC, EM] significantly prolonged gestational length, but there was no difference in BPD development (treatment group vs control group: RR 0.90, 95% CI 0.71-1.14)[10)]. Therefore, the standard treatment recommended outside of Japan (ABPC/AMPC + EM) is not expected to be effective in preventing the development of BPD.

2.1.3 EXPECTED EFFECTS OF ABPC/SBT + AZM

In pPROM with onset less than 28 weeks of gestation, a high rate of intrauterine *Ureaplasma* spp. is found. In 22 pregnant women with pPROM occurring at <28 weeks of gestation, positive amniotic fluid cultures have been reported in 91% and positive *Ureaplasma* in 55%[11)]. Macrolide antimicrobials are effective against *Ureaplasma* spp. infections, but EM is reported to be ineffective due to low intrauterine transfer rates.[12)]

Thus, especially for pPROM under 28 weeks of gestation, the probability of intrauterine infection is expected to be high. Therefore, different antimicrobial management (broad-spectrum penicillins or cephems effective against common bacteria and macrolides effective against *Ureaplasma* spp.) will be considered.

Therefore, in this study, instead of the standard treatment recommended outside of Japan (ABPC/AMPC + EM), ABPC/AMPC was replaced by the more broad-spectrum ampicillin sodium/sulbactam sodium (ABPC/SBT), which could be administered for up to 14 days instead of 7 days. The duration of EM administration was also set to 14 days instead of 7 days. We believe that this may have a potential effect on BPD, which could not be expected with the conventional method.

AZM has been reported to have a very high rate of transfer to the placenta[13)].

The incidence of BPD moderate/severe (severity classification published by NICHD in 2001) in infants born to pPROM pregnant women in Japan with onset at <28 weeks of gestation, who were complicated amniotic fluid infection and inflammation, was higher in the anti-*Pseudomonas*

aeruginosa penicillin (PIPC) or cefmetazole (CMZ) + clindamycin (CLDM) group (10/11) than in the ABPC/SBT + AZM group (2/11 cases).[11)] When AZM was treated in pregnant women with amniotic fluid *Ureaplasma* spp. infection, the median and quartiles of amniotic fluid interleukin (IL)-6 (12 cases) were 28.2 ng/mL (10.8-73.7) and 2.4 ng/mL (0.5-23.1), respectively, before and 2 days after administration, and the *Ureaplasma* spp. nucleic acid levels (8 cases) were 2.21×10^8 /mL (3.12×10^7 -4.96×10^8) and 4.29×10^6 /mL (3.47×10^5 -1.48×10^7), respectively, showing a significant decrease in both amniotic fluid IL-6 and *Ureaplasma* spp. levels (Nomiyama *et al*., unpublished data). Nevertheless, only 40% of the patients became negative for *Ureaplasma* spp. within 7 days. However, in two patients in whom AZM was used twice beyond day 7 from the start of administration, one patient became negative for *Ureaplasma* on day 15, and the intrauterine inflammation became milder after day 9. This may be due to the effect of repeated AZM administration. In the other case, *Ureaplasma* did not become negative, but the intrauterine inflammation normalized on day 12. This may be due to the fact that AZM reduced *Ureaplasma* and normalized inflammation, although it did not eradicate *Ureaplasma*. Thus, in some cases, administration of AZM on or after the seventh day after the start of treatment inhibited the growth of *Ureaplasma* spp. in the uterus, and as a result, normalized or mildly reduced intrauterine inflammation, thereby prolonging the duration of pregnancy. In addition, two patients treated with long-term AZM did not develop BPD_{36} . Based on these facts, we believe that the administration of EM or AZM for up to 2 weeks in pPROM with onset of pregnancy <28 weeks of gestation may be meaningful in reducing intrauterine inflammation caused by *Ureaplasma* and thus in preventing BPD_{36}. However, this study[11)] is not a randomized controlled trial with matched patient backgrounds, but rather a before-and-after study, and it is unclear whether AZM is truly preventable in the development of BPD.

Thus, although both ABPC/SBT + EM and ABPC/SBT + AZM are expected to be more effective against BPD than conventional methods, no trials have ever been conducted for BPD using these methods, and therefore no data exist on how good the treatment outcome can be expected. Therefore, we planned a parallel-group study comparing ABPC/SBT + EM and ABPC/SBT + AZM, rather than a single-arm study using ABPC/SBT + AZM, and a phase II study to investigate the safety of ABPC/SBT, EM, and AZM. The study was designed to compare the safety of ABPC/SBT, EM, and AZM. The data on BPD incidence in both groups obtained in this study will provide essential information for calculating the sample size for the planned Phase III trial.

Regarding safety, with regard to EM, no serious (or critical) adverse events have occurred in pPROM cases at Jichi Medical University, except for vascular pain, which has been treated with a protocol similar to that used in this study. In addition, antimicrobials are often administered for pPROM, usually for up to 2 weeks. In addition, there have been no reports of serious (or critical) adverse events of EM to mothers and infants at academic meetings to our knowledge. On the other hand, we believe that the incidence of adverse events and safety of the use of AZM should be investigated in detail because the dose and the number of days of administration of AZM are higher than those used in previous clinical trials. If a causal relationship is determined to be found, we have also established a system to ensure that the clinical research insurance will provide adequate compensation. If a large number of serious adverse events occur during the course of the study, we will consider cancelation of the entire trial.

In conclusion, ABPC/SBT + AZM administration is expected to be more effective than ABPC/AMPC + EM administration recommended outside of Japan in preventing and treating intrauterine infection, and is expected to prevent the onset of BPD by preventing the transition to fetal lung injury and neonatal lung infection and inflammation via amniotic fluid inflammation and infection mainly by *Ureaplasma* spp.

2.2 SIGNIFICANCE OF THE STUDY

In Japan, ABPC/SBT, AZM, and EM are all off-label use against pPROM. EM in combination with ABPC/SBT is a recommended therapy for pPROM in foreign countries, however, it is rarely used in Japan. There have also been few studies of AZM administered in combination with ABPC/SBT.

Therefore, this study will explore and compare the efficacy and safety of AZM and EM in combination with ABPC/SBT as prophylaxis for the development of BPD in infants born to pregnant women with pPROM with onset at <28 weeks of gestation.

If this study shows that AZM administration is expected to be more effective than EM administration in preventing the development of BPD under the ABPC/SBT combination, a Phase III validation study will be conducted.

If a large number of newborns born to pregnant women with pPROM can be escaped from the development of moderate/severe BPD, it is expected that in the short term the number of days of oxygen administration and artificial ventilation will be reduced, and the number of hospital days will decrease and the number of drugs such as steroids will decrease, thus contributing to health economics. In the long term, it is expected to prevent the decline in school-age IQ caused by BPD and improve the intellectual prognosis of very low birth weight infants. Early discharge from the hospital is also expected to shorten the period of separation from mother and child and promote attachment between mother and child. Furthermore, in extremely preterm infants born at <28 weeks of gestation, the higher the amount of *Ureaplasma* bacteria in the placenta, the lower the respiratory function (%FEF50) at 8 years of age (Yanagihara *et al*., unpublished data). If AZM contributes to a decrease in intrauterine *Ureaplasma* spp., it is also expected to improve the infant's respiratory function over the long term.

3 OBJECTIVE AND EVALUATION ITEMS

The aims of this trial are:

1) to investigate the effectiveness and safety of AZM and EM under the co-administration of ABPC/SBT to prevent BPD in infants who are delivered by pregnant women with pPROM occurring at <28 weeks of gestation, and to calculate the sample size requirements for a full-scale clinical trial powered at 80% to determine a clinically important difference in the occurrence rate of BPD between AZM and EM treatments,
2) to investigate the association between the detection of *Ureaplasma* spp. in the vagina, on the surface of the placenta, in the neonatal throat, and in the neonatal ear cavity and the occurrence of BPD in infants who are delivered by pregnant women with pPROM occurring at <28 weeks of gestation.

Therefore, a phase II multicenter, open-label, randomized, parallel-group study will be conducted. The primary outcome will be the proportion of events with events being BPD_{36} of moderate or greater and child death by 36 weeks PMA. The BPD diagnosis will be made in an evaluator-blind fashion (PROBE method) by a BPD Diagnosis and Evaluation Committee consisting of three medical experts who are not directly involved in this study and who will be selected from institutions not participating in this study.

To explore the efficacy and safety of AZM and EM in combination with ABPC/SBT, the following secondary endpoints will be established.

Secondary outcomes (Efficacy evaluation items for mother)

Days from pPROM onset to delivery
Weeks of gestation at delivery
Reasons for termination of pregnancy
Delivery findings (mode of delivery, stillbirth, placental weight)
Intrauterine fetal death rate
Histologic chorioamnionitis rate
Caesarean section ratio
Percentage of placental abruption

Secondary outcomes (Efficacy evaluation items for child)
BPD_{28} occurrence rate
Perinatal mortality (intrauterine fetal death + early neonatal death) rate
Percentage of deaths before discharge
Birth weight, birth height, birth head circumference
Hospitalization days (birth date to discharge date)
Invasive artificial ventilation days
Non-invasive artificial ventilation days
Number of days when oxygen was administered
Percentage of children who required surfactant administration
Neonatal RDS incidence rate
Prolonged pulmonary hypertension incidence rate
Intracranial hemorrhage incidence rate
PVL incidence rate
Percentage of sepsis
NEC incidence rate
Symptomatic ductus arteriosus incidence rate

Secondary outcomes (Safety evaluation iterms for mother and fetus)
Occurrence of adverse events and side effects
Occurrence of serious adverse events and serious side effects
Incidence of critical adverse events (fetal death, sepsis, intensive care unit (ICU) admission, multiple organ failure, artificial ventilation, total hysterectomy)

Secondary outcomes (Safety evaluation iterms for child)
Occurrence of serious adverse events and side effects

Secondary outcomes (Other evaluation items in the current trial):
Detection rate of *Ureaplasma* spp. (in vagina, on surface of the placenta, in infantile pharynx, and in infantile ear cavity).

4 STUDY DESIGN

4.1 STUDY DESIGN

To compare the efficacy and safety of AZM and EM in combination with ABPC/SBT for the prevention of BPD in infants born to pregnant women with pPROM occurring at <28 weeks of

gestation, we will plan a phase II, multicenter, open-label, randomized, parallel-group study. The efficacy of AZM and EM will be evaluated by comparing the rate of events, with events defined as BPD_{36} of moderate or greater and infant death up to 36 weeks PMA. Safety will be evaluated by the occurrence of adverse events and side effects in mother and child.

The study is a clinical trial conducted during the hospitalization period for pregnant women admitted after the onset of pPROM and consists of a screening phase, a treatment observation phase from the date of protocol treatment initiation to termination of pregnancy, and a post-termination observation phase from termination of pregnancy to the date of discharge of the child.

The chief investigator (CI) or sub-nvestigator (SI) will screen and assess eligibility after obtaining consent. The CI or SI will enroll subjects in REDCap prior to the start of protocol treatment. After enrollment, eligible subjects will be assigned to the intervention or control group by stratified allocation. The screening period from obtaining consent to randomization will be a maximum of 3 days.

The CI or SI will initiate protocol treatment within 4 days of obtaining consent. The study drug Zithromax 500 mg for intravenous infusion or Erythrosine 500 mg for intravenous infusion will be administered under inpatient supervision with the start date as Day 1.

The protocol treatment period is 14 days from Day 1 to Day 14. If a decision of termination of pregnancy is made[*1] before completing the 14 days of dosing, the protocol treatment will be discontinuated at that time.

From the end of protocol treatment until “date of birth”[*2], the prescribed observations, investigations, and tests are performed under inpatient supervision.

After the termination of pregnancy, the study will be completed with an examination at the time of termination of pregnancy, an examination at the time of the mother's discharge examination, an examination 28 days after delivery, an examination at 36 weeks 0 days PMA, and an examination at the time of the child's discharge from the hospital. The safety of the subjects (mother and fetus) will be ensured by inpatient or outpatient management after the termination of pregnancy.

In the event of cancelation of participation in the trial, a cancelation examination will be conducted within 3 days of cancelation whenever possible, and a follow-up survey will be conducted after cancelation of participation in the trial, usually at the mother's one-month health examination.

[*1]The date when CI or SI will decide the termination of pregnancy shall be the “date of the pregnancy termination decision”.

[*2]The date when the fetus will be delivered shall be the “date of birth”.

A schematic of the study design is shown in Figure 4.1-1.

Figure 4.1-1 Schematic of study design

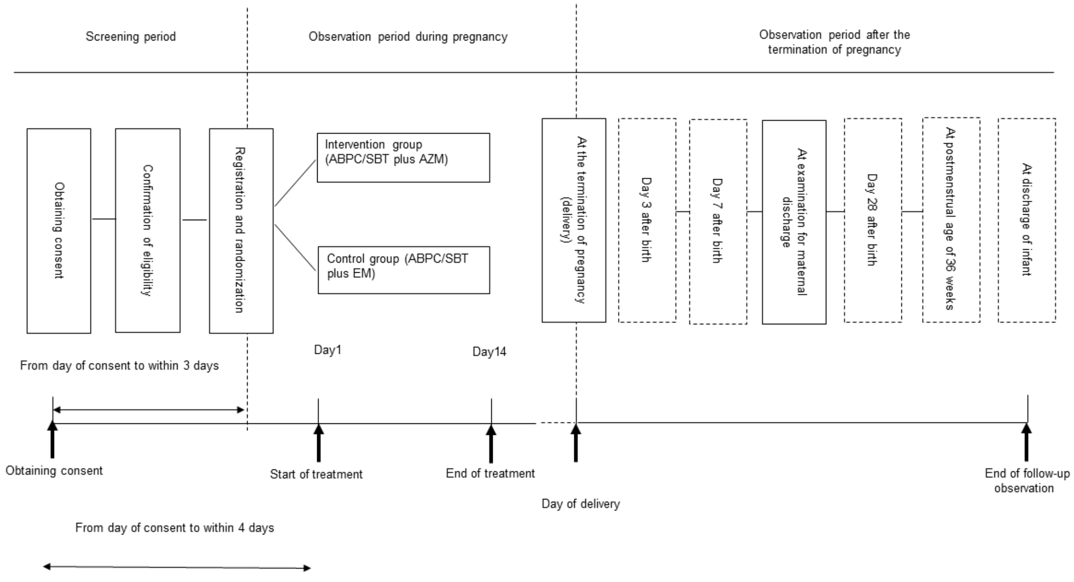

CI or SIs must obtain written consent by the screening test.

The maximum screening period (from consent acquisition to registration/randomization) is 3 days.

CI or SIs must start protocol treatment within 4 days of obtaining consent. Predetermined observations, investigations, and tests must be performed prior to administration of the study drug. If the decision to terminate the pregnancy is made before the protocol treatment is completed, the protocol treatment must be terminated at that point, and the patient shifts to the post-pregnancy observation period after delivery.

4.2 RANDOMIZATION

Allocation will be done by REDCap in the order of registration. REDCap will assign subjects (maternal and fetal) who meet all inclusion criteria to the intervention or control group 1:1 by stratified allocation. Because the incidence of BPD varies with the number of weeks of gestation at the onset of pPROM (22 weeks 0 days to 23 weeks 6 days and 24 weeks 0 days to 27 weeks 6 days) and facility (early caffeine-using facility or early caffeine-unused facility), stratification factors will be used at the time of randomization. The implementing facility will be surveyed prior to participation to determine if the facility is an early caffeine using facility.

4.3 REASONS OF SCIENTIFIC RATIONALITY

In Japan, AZM and EM have not been approved for the treatment of pPROM, and clinical trials have not been conducted. Therefore, we decided to conduct a phase II, multicenter, open-label, randomized, parallel-group, open-label study to explore and compare the efficacy and safety of

AZM and EM as prophylaxis against BPD in infants born to pregnant women with pPROM occurring at <28 weeks of gestation.

In this study, referring to previous studies, the target population was defined as pregnant women with singleton pregnancies who developed pPROM from 22 weeks 0 days to 27 weeks 6 days gestation. The earlier the gestational week of pPROM onset, the BPD incidence rate is higher. Therefore, weeks of gestational age at onset of pPROM will be used as a stratification factor for randomization.

The primary outcome of this study was the incidence of moderate or higher BPD_{36} or infant death by 36 weeks PMA. BPD is a lung tissue disorder that develops due to prematurity, prolonged ventilator management, high concentrations of oxygen, infection, etc. It occurs mainly in preterm infants and requires long-term oxygen administration and respiratory support (e.g., ventilator with tracheal intubation, nasal continuous positive airway pressure [NCPAP], high-flow nasal cannula [HFNC]).

Most BPD definitions proposed in recent years use the presence or absence of oxygen and respiratory support[1,14-17)]. In addition, the current classification of severity using oxygen and respiratory support use at 36 weeks PMA is the classification reported by NICHD in 2001,[1)] which is widely used both inside and outside the country. Therefore, for this study, BPD_{36} occurrence is defined as "presence or absence of oxygen or respiratory support use at 36 weeks PMA (for children born at 32 or more weeks of gestation at birth, presence or absence of oxygen or respiratory support use at days 28 to 56 or discharge home, whichever occurs first)" and BPD severity classification uses "NICHD, 2001"[1)]. If the patient is using between 22% and 30% oxygen at the time of assessment, the supplement oxygen reduction test (SORT)[18-19)] is used to diagnose BPD.

5 TARGET GROUP

5.1 ELIGIBILITY CRITERIA

5.1.1 SELECTION CRITERIA

All of the following criteria must be met in order to participate in this study

1) Pregnant woman who gave consent to participate in the current trial.
2) Pregnant woman whose age at the time of receiving informed consent is equal to or over 18 years old.
3) Pregnant woman with a singleton pregnancy who developed pPROM at 22 weeks 0 days - 27 weeks 6 days.

The diagnosis of pPROM is defined as persistent leakage, pooling, or both on cusco examination.

If the patient only tests positive with a water breakage diagnostic reagent, it is not considered a pPROM.

5.1.2 EXCLUSION CRITERIA

If any of the following criteria apply to subject, the subject must not participate in this study.

1) Pregnant woman with placenta previa.
2) Pregnant woman with trachelectomy; however, pregnant woman with cervical incision should not be excluded, because they are at high risk for the occurrence of pPROM.

3) Pregnant woman with hypertensive disorders of pregnancy; however, pregnant woman with chronic hypertension should not be excluded.
4) Pregnant woman with a fetus who shows either absent end-diastolic velocity or reverse end-diastolic velocity before the onset of pPROM.
5) Pregnant woman with any malignant tumors for whom the attending physicians consider the termination of pregnancy at or soon after the onset of pPROM.
6) Pregnant woman with pPROM for whom the attending physicians consider the imminent termination of pregnancy at or soon after the onset of pPROM.
7) Pregnant woman with pPROM who has had diseases or symptoms causing attending physicians to consider the imminent termination of pregnancy at or soon after the onset of pPROM.
8) Pregnant woman with the administration of AZM due to Chlamydia infection within 2 weeks before the onset of pPROM.
9) Pregnant woman receiving any kinds of antibiotics other than either penicillin or cephem antibiotics for two weeks before the onset of pPROM.
10) Pregnant woman who has been administered antibiotics rather than either ABPC or ABPC/SBT, from the onset of pPROM to the time obtaining consent; However, the following pregnant woman should not be excluded: when the administration of antibiotics rather than either ABPC or ABPC/SBT (excluding any kinds of macrolide antibiotics) were performed one day before the time of obtaining consent or earlier and the administration period of them were within 3 days.
11) Pregnant woman with the administration of any kinds of macrolide antibiotics, from the onset of pPROM to the time obtaining consent.
12) Pregnant woman with allergy for either penicillin or macrolide antibiotics.
13) Pregnant woman complicated with infectious mononucleosis.
14) Pregnant woman with a fetus with either chromosomal abnormalities or suspected severe malformation syndrome.
15) Pregnant woman with a creatinine level of ≥1.1 mg/dL at the screening test just after obtaining consent.
16) Pregnant woman complicated with QT syndrome.
17) Pregnant woman with any types of psychiatric disorders for whom the attending physicians think that the patient should not participate in the current trial.
18) Pregnant woman for whom the attending physicians think that the patient should not participate in the current trial, because there are some factors that may hamper the achievement of the trial.

5.1.3 REASONS FOR SETTING

Criteria for inclusion
1) Set from an ethical point of view.
2) To select the appropriate age for evaluation of the study drug.
3) To select patients diagnosed with eligible diseases.

Criteria for exclusion
1) - 11) Set from the viewpoint of the efficacy of the study drug.
12) - 17) Set from the viewpoint of safety of subjects (mother and fetus).
18) To exclude patients who were not eligible for the study.

5.2 TARGET NUMBER OF CASES

5.2.1 TARGET NUMBER OF CASES

Target number of cases: 50 in the test group and 50 in the control group (total 100 cases)

5.2.2 REASONS FOR SETTING THE NUMBER OF CASES

In this study, since fetal and neonatal deaths were considered to be at higher risk of developing BPD and infant deaths up to 36 weeks PMA were also considered as one of events, infant deaths up to 36 weeks PMA (5%) were also taken into account to establish effect sizes for the two groups.

In a previous foreign study, the incidence of BPD_{36} was 26.4% (29/110, all children survived) when 110 pregnant women who developed pPROM at <28 weeks of gestation were treated with AZM.[20)] In a previous study in Japan, the incidence of BPD_{36} was 18.2% (2/11, all children survived) when 11 pregnant women with pPROM at <28 weeks of gestation were treated with ABPC/SBT + AZM.[11)] In addition, when ABPC/SBT + AZM was administered to 10 pregnant women with pPROM at <28 weeks of gestation from June 2013 to May 2020 at Saga Hospital, the incidence of BPD_{36} was 40.0% (4/10, all children survived) (Nomiyama *et al*, unpublished data). Based on the above, the incidence of moderate or severe BPD_{36} or child death by 36 weeks PMA in the intervention group (AZM group) was assumed to be 18.0% to 40.0%.

On the other hand, the literature search did not retrieve any reports of EM administration to pregnant women with pPROM at <28 weeks' gestation alone. Therefore, we assumed that the incidence rate of moderate or severe BPD_{36} or infant death by 36 weeks PMA in the control group (EM group) was 60.0% based on the general BPD_{36} incidence rate.

The median number of weeks of gestational duration for pregnant women with pPROM is 2 weeks. Based on the annual number of deliveries, the number of births by week, and the percentage of pPROM, we can estimate that 1,200 newborns per year are born at <30 weeks to pregnant women with pPROM complications. In the 20 regional and comprehensive perinatal centers scheduled to participate in this study, the number of cases is estimated to be approximately 200 per year over a 2-year period. Assuming that 50% of the patients meet the eligibility criteria for this study and consent is obtained, 100 patients can be enrolled over a two-year period, and from the viewpoint of feasibility, the target number of cases for this study was set at 100. Under a two-tailed test and a significance level of 5%, the power of detection ranged from 0.516 to 0.995 when the assumed BPD_{36} incidence rate in the intervention group was 18.0% to 40.0%, which is considered to guarantee a power of at least 0.5.

For reference, the power curve for the BPD_{36} incidence rates of 18.0% to 60.0% in the intervention group are shown below. In addition, the incidence rates in previous foreign and domestic studies and the study at Saga Hospital are indicated by red lines.

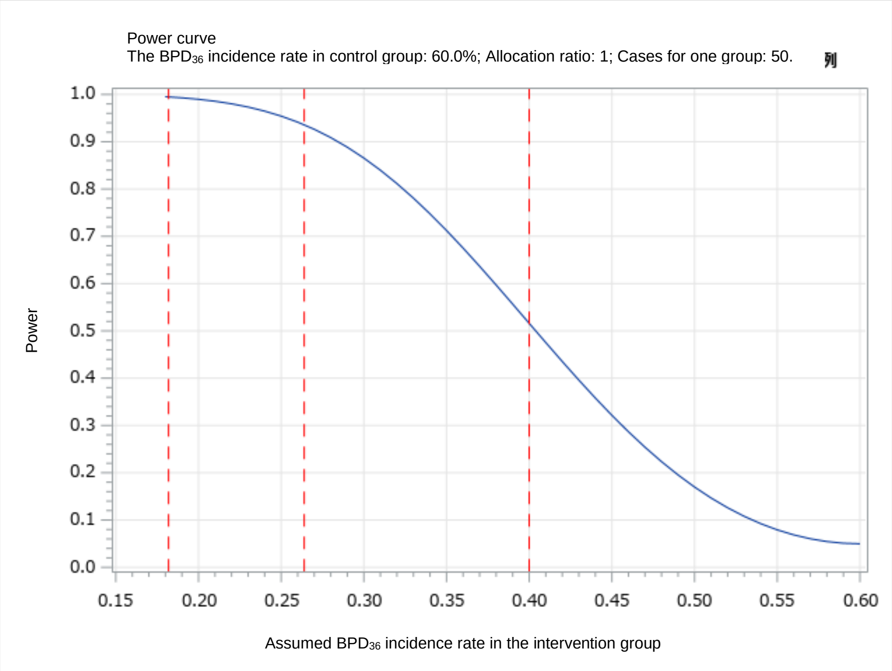

6 INTERVENTION

6.1 DRUGS USED

6.1.1 SUMMARY OF DRUGS

The following test and control drugs (study drugs) will be used in this study.

Test drug

Generic name	Sulbactam Sodium, Ampicillin Sodium for Injection
Product name/manufacturer	Sulbacillin 1.5g for Injection (Meiji Seika Pharma Co., Ltd.)
Dosage form, product description, and content	Each vial contains 0.5 g (potency) of Japanese Pharmacopoeia Sulbactam Sodium and 1 g (potency) of Japanese Pharmacopoeia Ampicillin Sodium, and is an injectable solution to be dissolved during use.<br>White to yellowish-white powder, pH: 8.0-10.0 [1.5 g (potency)/10 mL, aqueous solution].
Therapeutic category	Antibiotic preparations with beta-lactamase inhibitor
Indications: susceptible strains	Staphylococcus spp. (ABPC/SBT-sensitive), Streptococcus pneumoniae, Moraxella (branhamella) Catalysis, Escherichia coli, Proteus, Haemophilus influenzae
Indications: adaptation disease	Pneumonia, lung abscess, cystitis, peritonitis
Dosage and Administration	[Pneumonia, lung abscess, peritonitis] The usual adult dosage of sulbactam sodium ampicillin sodium is 6 g (potency) per day divided

	into two doses for intravenous injection or intravenous drip infusion. In cases of severe infection, the dose may be increased as necessary, but should not exceed 3 g (potency) four times a day (12 g (potency) as the daily dose).<br><br>[For cystitis] Normally, for adults, sulfactam sodium ampicillin sodium is administered in two divided doses of 3 g (potency) per day by intravenous injection or intravenous drip infusion.
Storage conditions	Store at room temperature

Generic name	Azithromycin Hydrate for Injection
Product name/manufacturer	ZITHROMAC Intravenous use (Pfizer Inc.)
Dosage form, product description, and content	Each vial contains 524.1 mg of Japanese Pharmacopoeia azithromycin hydrate (500.0 mg (potency) of azithromycin). White mass or powder (lyophilized), pH 6.2-6.8 (in solution at a concentration of 100 mg/mL dissolved in 4.8 mL of water for injection).
Therapeutic category	15-membered ring macrolide antibiotic preparation
Indications: susceptible strains	Azithromycin-sensitive Staphylococcus spp., Streptococcus spp., Streptococcus pneumoniae, Neisseria gonorrhoeae, Moraxella (branhamella) catalis, Haemophilus influenzae, Legionella pneumophila, Peptostreptococcus, Prevotella, Chlamydia, Mycoplasma spp.
Indications: adaptation disease	Pneumonia, lung abscess, cystitis, peritonitis
Dosage and Administration	For adults, azithromycin is administered at a dose of 500 mg (potency) once daily over 2 hours by intravenous drip infusion.
Storage conditions	Store at room temperature

Generic name	Azithromycin Hydrate Tablets
Product name/manufacturer	ZITHROMAC tablets (Pfizer Inc.)
Dosage form, product description, and content	White film-coated tablets containing 262.0 mg of azithromycin hydrate (250 mg of azithromycin (potency) per tablet)
Therapeutic category	15-membered ring macrolide antibiotic preparation
Indications: susceptible strains	Azithromycin-sensitive Staphylococcus spp., Streptococcus spp., Streptococcus pneumoniae, Neisseria gonorrhoeae, Moraxella (branhamella) catalis, Haemophilus influenzae, Legionella pneumophila, Peptostreptococcus, Prevotella, Chlamydia, Mycoplasma spp.
Indications: adaptation disease	Deep skin infection, lymphatic duct and lymphadenitis, pharyngolaryngitis, tonsillitis (including peritonsillar and peritonsillar abscess), acute bronchitis, pneumonia, lung abscess, secondary infection of chronic respiratory tract lesions, urethritis,

	cervicitis, pelvic inflammatory disease, sinusitis, periodontitis, periapical inflammation, jaw inflammation
Dosage and Administration	<deep skin infections, lymphatic duct and lymphadenitis, pharyngolaryngitis, tonsillitis (including peritonsillar and peritonsillar abscess), acute bronchitis, pneumonia, lung abscess, secondary infection of chronic respiratory tract lesions, sinusitis, periodontitis, periapical inflammation, jaw infection><br>For adults, azithromycin is administered orally at a dose of 500 mg (potency) once daily for a total of 1.5 g (potency) for 3 days.<br><urethritis, cervicitis><br>For adults, azithromycin is administered orally at a dose of 1000 mg (potency).<br><pelvic inflammatory disease><br>For adults, after treatment with azithromycin injection, azithromycin should be administered orally at a dose of 250 mg (potency) once daily.
Storage conditions	preservation at room temperature

Control drug

Generic name	Erythromycin Lactobionate
Product name/manufacturer	Erythrocin for Intravenous Infusion (Mylan EPD LLC)
Dosage form, product description, and content	1 vial contains 500 mg (potency) of Japanese Pharmacopoeia erythromycin lactobionate, a white powder (lyophilized injection). pH 5.0-7.5 [50 mg (potency)/mL solution]
Therapeutic category	Macrolide antibiotic preparations
Indications: susceptible strains	Secondary infections such as trauma, burns, and surgical wounds, pneumonia, diphtheria
Indications: adaptation disease	Staphylococcus spp., Streptococcus spp., Streptococcus pneumoniae, and Diphtheria spp. sensitive to erythromycin
Dosage and Administration	The usual adult dosage of erythromycin is 600 to 1500 mg (potency) per day divided into two to three doses and administered intravenously over at least two hours each time. The dosage may be adjusted according to age and symptoms.
Storage conditions	Store at room temperature

Generic name	Erythromycin Stearate Tablets
Product name/manufacturer	Erythrosine Tablets (Mylan EPD LLC)
Dosage form, product description, and content	Enteric-coated film-coated tablets containing 200 mg of Japanese Pharmacopoeia erythromycin steatate (potency) per tablet.
Therapeutic category	Macrolide antibiotic preparations
Indications: susceptible strains	Staphylococcus spp. (susceptible to EM), Streptococcus spp., Streptococcus pneumoniae, Neisseria gonorrhoeae, Bacillus meningitidis, Diphtheria, Shigella dysenteriae, Bacillus subtilis,

	Bacillus pertussis, Tetanus, Gas gangrene group, Treponema syphiliticus, Chlamydia trachomatis, Mycoplasma spp.
Indications: adaptation disease	Superficial skin infections, deep skin infections, lymphatic duct and lymphadenitis, chronic pyoderma, secondary infections from trauma, burns, and surgical wounds, mastitis, osteomyelitis, pharyngolaryngitis, tonsillitis (including peritonsillitis), acute bronchitis, pneumonia, lung abscess, empyema, secondary infection from chronic respiratory tract lesions, cystitis, pyelonephritis, urethritis, gonococcal infection, Soft chancre, syphilis, venereal (inguinal) lymphogranuloma, infectious enteritis, intrauterine infection, adnexitis, dacryocystitis, malt granuloma, otitis externa, otitis media, sinusitis, pericoronitis, scarlet fever, diphtheria, pertussis, tetanus, gas gangrene, amoebic dysentery
Dosage and Administration	The usual oral dosage for adults is 800 to 1,200 mg (potency) of erythromycin daily in 4 to 6 divided doses.
Storage conditions	Store at room temperature

6.1.2 PREDICTED ADVERSE EFFECTS

Both the test drug and the drug used as a control (study drug) are for off-label use. Refer to the respective package inserts/interview forms for anticipated side effects.

6.2 PROTOCOL TREATMENT

6.2.1 DOSAGE AND ADMINISTRATION

The day that the study drug Zithromax 500 mg or Erythrosine 500 mg for intravenous infusion is administered is defined as the protocol treatment start date (Day 1), and protocol treatment is initiated under inpatient supervision. The full daily dose is administered unless criteria for discontinuation of protocol treatment are met.

From the time consent is obtained until the day before protocol treatment begins, ABPC infusion or ABPC/SBT infusion can be administered.

<Intervention group>

（1）Sulbacillin for intravenous injection 1.5g

Sulbacillin for intravenous infusion 1.5 g once a day, 4 times a day for a total of 14 days (Day 1 to Day 14).

Dose intravenously over 1 hour every 6 hours ± 1 hour.

（2）Zithromax 500 mg for intravenous infusion

Zithromax 500 mg for intravenous infusion is administered intravenously 500 mg once daily for a total of 4 days (Day 1 to Day 2, Day 8 to Day 9).

Dose intravenously every 24 hours ± 2 hours over 2 hours.

（3）Zithromax Tablets 250 mg

Take one 250 mg Zithromax tablet once daily (after breakfast) for a total of 10 days (Day 3 to Day 7 and Day 10 to Day 14).

If a missed dose is noticed within 4 hours of the dose time, take the dose immediately. If more than 4 hours have elapsed, do not take that dose and resume taking the next dose as usual.

<Control group>

(1) Sulbacillin for intravenous injection 1.5g

Sulbacillin for intravenous infusion 1.5 g once a day, 4 times a day for a total of 14 days (Day 1 to Day 14).

Dose intravenously over 1 hour every 6 hours ± 1 hour.

(2) Erythrosine for intravenous infusion 500 mg

Erythrosine for intravenous infusion 500 mg is administered intravenously 500 mg twice daily for a total of 4 days (Day 1 to Day 2, Day 8 to Day 9).

The drug is administered intravenously every 12 hours ± 2 hours for at least 2 hours at a time.

(3) Erythrosine Tablet 200mg

Take one 200 mg erythrocin tablet four times a day for a total of 10 days (Day 3 to Day 7 and Day 10 to Day 14).

If a missed dose is noticed within 2 hours of the dose time, take the dose immediately. If more than two hours have elapsed, do not take the dose that time and resume taking it as usual the next time.

6.2.2 RATIONALE FOR ESTABLISHING PROTOCOL TREATMENT

<Abour ABPC/SBT>

In particular, since pPROM under 28 weeks of gestation are expected to have a high probability of already having an intrauterine infection, different antimicrobial management (broad-spectrum penicillins or cephems effective against common bacteria and macrolides effective against Ureaplasma spp.), which is not usually used for pPROM occurring at or over than 28 weeks of gestation, will be considered.

Therefore, in this study, instead of the standard treatment recommended outside of Japan (ABPC/AMPC + EM), ABPC/AMPC was replaced by the more broad-spectrum ABPC/SBT, which could be administered for up to 14 days instead of 7 days. The duration of EM administration was also set to 14 days instead of 7 days. We believe that this method may have a potential effect on BPD, which could not be expected with the conventional method.

<About EM and AZM>

In this study, we established a dosing regimen (switch therapy) for EM and AZM that consists of 2 days of intravenous infusion plus 5 days of oral administration repeated twice.

The control group was not placebo, but ABPC/SBT + EM group. The current standard treatment is ABPC (intravenous infusion) for 2 days ⇨ AMPC (oral) for 5 days + EM (intravenous infusion) for 2 days ⇨ EM (oral) for 5 days in the US, EM (oral) for 10 days in the UK, and one of the above in Canada, which all include EM. In the present study, because the number of gestational weeks is earlier than the pPROM subjects in the previous RCTs, and in many cases mixed infection by common bacteria already develops in utero, we thought it appropriate to change ABPC⇨AMPC to ABPC/SBT (intravenous infusion) and furthermore, to increase the administration period from 7 to 14 days. For the use of EM in the control group, we chose to use a combination of intravenous and oral EM (switch therapy), in accordance with European and US guidelines. Furthermore, since the gestational age of the patients is earlier than that of the pPROM subjects in the previous RCTs and many of them already develop intrauterine infection with Ureaplasma, we considered it appropriate to increase the administration period from 7 days (one course) to 14 days (two courses).

In the test group, since one objective was to verify the significance of AZM over EM, the ABPC/SBT administration method was not changed, and the EM portion of the study was administered with AZM. AZM (intravenous infusion) for 2 days ⇨ AZM (oral) for 5 days. This treatment could be continued for a maximum of two courses. The reason for this is that in a previous study, when Ureaplasma infection was present in the uterus, 3 days of AZM (intravenous infusion) resulted in only about 40% disappearance of Ureaplasma in the amniotic fluid after 1 week, and in 2 cases where 2 courses of 3-day treatment were conducted, Ureaplasma did not disappear, but intrauterine inflammation was controlled and BPD did not occur.

6.2.3 DOSE CHANGE

No changes in protocol treatment doses are allowed in this study.

6.2.4 INTERRUPTION OR WITHDRAWAL

(1) For intravenous infusion

Administration of intravenous infusion of the test drug or control drug may be interrupted only when an adverse event or other event occurs after the start of intravenous infusion of the test drug or control drug and it is deemed necessary to interrupt the intravenous infusion of the test drug or control drug. If the intravenous infusion is interrupted, the intravenous infusion will be resumed after the patient has recovered from symptoms and the CI or SI determines that the intravenous infusion of the test or control drug can be resumed. If intravenous administration of the test or control drug is interrupted and cannot be resumed on the day of administration, and the full dose cannot be administered, it will be treated as a drug withdrawal. If it cannot be resumed on the following day, protocol treatment should be discontinuated.

If an adverse event or other event occurs after the initial intravenous administration of the test or control drug and prior to the intravenous administration of the test or control drug, and it is determined that a drug withdrawal is necessary, the drug withdrawal is acceptable. Withdrawal is limited to 1 day. If withdrawal of more than 2 days is necessary, protocol treatment should be discontinuated.

(2) For tablets

A drug withdrawal will be permitted only when an adverse event or other event occurs after initiation of the test drug or control drug and a drug withdrawal is judged to be necessary. If a drug is withdrawn, it will be resumed after symptoms have resolved and the CI or SI determines that the study drug or control drug can be resumed.

6.2.5 PROTOCOL TREATMENT DURATION

The protocol treatment period is 14 days for each group; if a decision is made to terminate the pregnancy before completing the 14-day protocol treatment, the protocol treatment is discontinuated at that time.

6.2.6 CRITERIA FOR DISCONTINUATION OF PROTOCOL TREATMEN

If any of the following applies, protocol treatment for the subject (mother, fetus) will be discontinuated to ensure the safety of the subject (mother, fetus). If protocol treatment is discontinuated, consider appropriate antimicrobial therapy for each subject (mother and fetus) and switch to a different antimicrobial therapy.

After discontinuation of protocol treatment, the subject (mother and fetus) should continue to participate in the study unless "10.1 Cancelation of participation in the trial" applies, and the CI or SI should continue observation, investigation, and testing in accordance with the schedule (7.5).

1. When intrauterine infection is strongly suspected due to Lencki's diagnostic criteria or other reasons.
   Diagnostic criteria for clinical chorioamnionitis (CAM) by Lencki, et al.
   1) If maternal temperature is ≥38.0 degrees Celsius and at least one of the following 4 items (A to D) is observed: A. maternal tachycardia ≥100 beats/min, B. uterine tenderness, C. foul-smelling vaginal discharge or amniotic fluid, D. leukocytosis (≥15 000/mm^3)
   2) If all of the above 4 items are observed even if the mother's body temperature is <38.0 degrees Celsius

   However, since there is a possibility that 1) may occur due to pneumonia, pyelonephritis, appendicitis, meningitis, influenza, etc., these should be differentially diagnosed as much as possible during maternal fever.
2. When Pseudomonas aeruginosa or multidrug-resistant bacteria are detected in vaginal culture.
3. When adverse events occur, and it is necessary to withdraw protocol treatment for intravenous drip infusion for 2 days or more.
4. When the delivery is determined.
5. When the subject desires to discontinue protocol treatment.
6. In addition, when the attending physicians determine that it is difficult to continue the protocol treatment.

6.2.7 PACKAGING AND LABELING

Commercially available drugs Sulbacillin 1.5g for Injection (Meiji Seika Pharma Co., Ltd.), ZITHROMAC Intravenous use (Pfizer Inc.), ZITHROMAC tablets (Pfizer Inc.), Erythrocin for Intravenous Infusion (Mylan EPD LLC), and Erythrosine Tablets (Mylan EPD LLC) will be purchased for use in the study. Water for injection, saline solution, and 5% dextrose solution necessary for drug preparation and administration will also be purchased and used for the study. Study drugs should be managed and used separately from drugs that are usually prescribed in the clinic. Details will be specified separately in the study drug administration procedures for this study.

6.2.8 PREPARATION AND POST-PROCESSING

Dissolve 1.5 g of Sulbacillin 1.5g for Injection in 100 mL of rehydration solution and infuse intravenously over 1 hour. Use promptly after dissolution.

Dissolve ZITHROMAC Intravenous use in 4.8 mL of water for injection (concentration 100 mg/mL) and dilute to an injectable concentration of 1.0 mg/mL using an infusion solution such as 5% dextrose injection solution that has been confirmed to be free of compositional changes. When preparing a 100 mg/mL solution, do not use any solution other than water for injection, since there is no preparation data available. Use immediately after dissolution.

To prepare a 5% solution, add 10 mL of water for injection to one vial of erythrosine. The 5% solution is stable in the refrigerator for 2 weeks.

Zithromax Tablets 250 mg and Erythrosine Tablets 200 mg are oral medications in tablet form and should be removed from the PTP sheet immediately before taking. There is no special preparation required in advance.

6.3 MANAGEMENT METHOD

6.3.1 STORAGE AND DELIVERY

Study drugs will be purchased for study purposes and stored appropriately in the pharmacy or pharmacy department of each institution. When delivered, they should be prescribed for study use and provided directly to subjects by the CI or study partner pharmacist or SI. Unused and expired medications due to missed doses will be disposed of as usual in accordance with the hospital's drug disposal procedures. Details will be specified separately in the study drug administration procedures for this study.

6.3.2 DISPOSAL/RETURN

If a study drug is not used during the study period due to damage, contamination, or other defects in the study drug, the number of vials to be disposed of, serial number, expiration date, reason for disposal, and number of vials in stock after disposal should be recorded on the study drug control chart. When discarding, dispose of as medical waste in accordance with the regulations of each medical institution.

At the end of the study drug administration period, unused study drug product should not be returned or otherwise disposed of as medical waste in the same manner as for disposal.

6.4 CONCOMITANT MEDICATIONS

6.4.1 CONCOMITANT MEDICATIONS

The following items will be entered in the electronic case report form (eCRF) when the following drugs are used on the subject (mother or fetus) (maternal) from the date consent is obtained to the end of study participation (or when study participation is cancelated).

- Ritodrine hydrochloride
- Magnesium sulfate
- Betamethasone
- Antimicrobials used during exacerbations of infection after the end of the protocol treatment period

The following items will also be entered in the eCRF when an adverse fetal event occurs from the date of protocol treatment initiation to the time of pregnancy termination (or cancelation of participation in the trial) and when a drug or therapy other than the study drug is used for the adverse event.

Name of drug/therapy
route of administration
Start and end date of treatment
Reason for use (treatment)

Note that no entry is required for saline or other rehydration or dissolution solutions that are not intended for treatment.

6.4.2 CONCOMITANT RESTRICTED DRUGS ANS CONCOMITANT RESTRICTED TREATMENTS

The use of corticosteroids during the period of participation in the study will be permitted only for the following purposes, from the time consent is obtained until the termination of pregnancy.

(1) Use of maternal administration to prevent the onset of RDS via fetal lung maturation when preterm delivery is anticipated.
(2) Topical for skin, bronchus, ear, nose, throat, eye, oral cavity, anus, vagina, etc.
(3) Use of prednisolone for treatment of complications prior to study participation.

The use of antimicrobial agents from the time consent is obtained until the start of protocol treatment will only be allowed for ABPC infusion or ABPC/SBT infusion if deemed necessary by the CI or SI.

6.4.3 ANTIMICROBIAL THERAPY FOR EXACERBATIONS OF INFECTION AFTER COMPLETION OF PROTOCOL TREATMENT

If an exacerbation of infection is observed after completion of protocol treatment and before termination of pregnancy, antimicrobial therapy other than macrolide therapy may be administered, as deemed appropriate by the CI or SI.

For the purposes of this study, an infection exacerbation is defined as any of the following:
(1) WBC count or CRP level is worse than during the protocol treatment period, and the CI or SI determines that antimicrobial therapy is necessary
(2) Vaginal culture showing Pseudomonas aeruginosa or multidrug-resistant bacteria

6.4.4 CONCOMITANT PROHIBITED TREATMENT

In the control group, the following drugs must not be used concomitantly from the time consent is obtained until the end of the protocol treatment period.
(1) Macrolide preparations other than erythromycin
(2) Ergotamine (ergotamine tartrate, dihydroergotamine mesylate) containing preparations [CLEAMIN®, DIGIDELGOT®, etc.]
(3) Pimozide [Orap®].
(4) Asunaprevir [Sunbepra®].

Basis for setting
(1) Set for the same drug as the control drug.
(2) - (4): These were selected because the control drugs bind to CYP3A and form a complex, which may inhibit the metabolism of these drugs.

In the study group, the following drugs may not be used concomitantly from the time consent is obtained until the end of the treatment observation period.
(1) Macrolide preparations other than azithromycin

Basis for setting
(1) Set because of the same drug class as the test drug control.

7 STUDY METHODS AND PROCEDURES

7.1 SUBJECT RECRUITMENT

Pregnant women who are seeing an obstetrician/gynecologist at the implementing institution and who may meet the eligibility criteria based on prior medical record review will be informed about the study by their physician during their outpatient visit.

The relevant obstetrician/gynecologist at each practicing institution will be introduced to the study and asked to cooperate in referring pregnant women with pPROM at 22 weeks 0 days to 27 weeks 6 days gestation before administering antimicrobials whenever possible, and to administer antimicrobials by ABPC infusion or ABPC/SBT infusion.

7.2 CONSENT TO PARTICIPATE

After explaining the study subject in accordance with the method for obtaining consent, the fact that the explanation was given and the fact that consent was obtained should be noted in the medical record. If the subject does not consent on the date of explanation, but confirms his/her willingness to consent at a later date, the date of explanation and the physician's signature should be written on the consent form, which should be provided to the study subject together with the explanation. If the date of explanation and the date of consent are on the same day, record that the consent was given after sufficient time was given for consideration of participation in the study.

Screening may be performed on the same day after consent is obtained. If screening is not conducted on the same day, screening should be conducted within 3 days of the date of consent.

7.3 SCREENING

7.3.1 PRACTICE ITEM

Screening involves the following tests, observations and evaluations from the time of obtaining consent to enrollment.

If the screening results meet the 5.1 eligibility criteria, the subject (mother and fetus) will be enrolled in the study and will begin taking the study drug after the Day 1 (start date of administration) implementation items.

7.3.2 SCREENING DROPOUT

Subjects who do not meet the eligibility criteria as a result of the screening test will be considered as screening dropouts and will not be allowed to participate in the study. Subjects who fail to meet the screening eligibility criteria will not be rescreened.

7.4 SUBJECT ENROLLMENT

The principal investigator or subinvestigator enrolls subjects according to the following procedure:

(1) All subjects who have given consent will be assigned a subject identification code at the site in accordance with the following rules. The same subject identification code will be used from the time consent is obtained until the end of the study.
Method of Assigning Subject Identification Codes
Subject Identification Code: PROM-AZM-YYYY-ZZZ
YYYY: Implementing medical institution number
ZZZZ: Serial number of the subject whose consent will be obtained at the institution
(2) After obtaining consent, a screening test is performed to confirm that all selection criteria are met and none of the exclusion criteria are met.
(3) Log in to REDCap and enter your eligibility.
(4) Subjects who are determined to be eligible based on the information entered into REDCap will be assigned by REDCap to either the test or control group, with the assignment group presented on REDCap.

7.5 OBSERVATION ITEMS AND INFORMATION/PROCEDURES TO BE COLLECTED

Perform the necessary observations and investigations, etc. on each prescribed day according to the schedule (Tables 7.5-1 and 7.5-2). Unless otherwise noted, observations, investigations, and tests on the first day of protocol treatment (Day 1) should be performed from the time of waking to before administration of the study drug. Observations, investigations, and tests to be performed and collected on the specified days are as follows:

Table 7.5-1 Schedule of data collection and monitoring for participant (mother and fetus)

Period	Screening period[a]		Observation period during pregnancy[b]								Observation period after the termination of pregnancy		At the cancelation of the participation in the trial[d]	Follow-up observation after the cancelation of participation in the trial[e]
Working item	Screening period Days -3 to 0	Registration on Day 0	Week 1 Day 1	Week 2 Day 8 ± 2 day2	Week 3 Day 15 ± 2 days	Week 4 Day 22 ± 2 days	Week 5 Day 29 ± 2 days	Week 6 Day 36 ± 2 days	Week 7 Day 43 ± 2 days	Week 8〜[c] Day 50 ± 2 days	At the termination of pregnancy (delivery) ± 1 day	At examination for maternal discharge ± 2 days		
Obtaining consent[f]	X													
Participant: background information · body weight	X[o]													
Registration · randomization		X												
Care in hospital[g]			X	X	X	X	X	X	X	X	X	X		
Protocol treatment[h]			X	X										
Physical findings[i]	X		X	X	X	X	X	X	X	X	X	X	X	
Vital signs (blood pressure · pulse · body temperature)[i]	X		X	X	X	X	X	X	X	X	X	X	X	
Hematological examination[i]	X[p]			X	X	X	X	X	X	X	X	X	X	
Blood biochemical test[i]	X[p]			X	X	X	X	X	X	X	X	X	X	
Urinalysis	X[p]			X	X	X	X	X	X	X	X	X	X	

(Qualitative: protein · sugar)[i]														
ECG	X[p]													
Culture of vaginal microorganisms (each institute)[i]			X[r]	X[s]	X	X	X	X	X	X	X			
Culture of vaginal *Ureaplasma* spp.[j]			X[r]	X[s]										
PCR for vaginal *Ureaplasma* spp.[j]			X[r]	X[s]										
Culture of *Ureaplasma* spp. on the surface of the placenta[j]											X			
PCR for *Ureaplasma* spp. on the surface of the placenta[j]											X			
Histological examination for placenta											X			
Estimated fetal weight[i]	X[q]			X	X	X	X	X	X	X				
Fetal ultrasonography (Doppler · amniotic fluid volume estimation)[i]	X[q]			X	X	X	X	X	X	X				
Confirmation of drug used concomitantly[k]	X		X	X	X	X	X	X	X	X	X	X	X	X
Confirmation of adverse events[l]			X	X	X	X	X	X	X	X	X	X	X	X
Survey for the reasons of the termination of pregnancy[m]											X			
Findings on delivery[n]											X			

a. The maximum length of screening period is 3 days.
b. The planned interview, physical examination, ultrasonography, and sampling of biological specimens should be performed before the administration of test drugs. If the chief investigator (CI) or sub-investigator (SI) decides to terminate the pregnancy before the completion of protocol treatment, he/she should finish protocol treatment at the time of the decision, and will transfer to the observation period after the termination of pregnancy.
c. After week 8, CI or SI should perform the planned interview, physical examination, ultrasonography, and sampling of biological specimens once a week until the day of the termination of pregnancy.
d. CI or SI should perform the physical examination, sampling of biological specimens, and confirmation of drugs used concomitantly and adverse events within 3 days after the cancelation of participation in the trial.

e. CI or SI should perform follow-up observation after the cancelation of participation in the trial at the one-month postpartum medical examination.
f. CI or SI must obtain consent before starting examination during the screening period.
g. CI or SI should care for patients in hospital at least for the period from the start of protocol treatment to 3 days after delivery.
h. CI or SI must start protocol treatment within 4 days after obtaining consent.
i. CI or SI should examine the estimated fetal weight once a week at or after Week 3, until the decision to terminate the pregnancy. However, the hematological examination and blood biochemical test should be performed only when needed at or after Week 4, until the decision to terminate the pregnancy.
j. CI or SI should not take biological specimens from patients if the patients become complicated with COVID-19 infection. In addition, all preserved specimens should be abandoned.
k. If CI or SI decides to cancel participation in the trial due to the adverse events, CI or SI should continue the investigation of adverse events due to the test drugs or drugs used concomitantly which are the reasons for the cancelation of participation in the trial, from the cancelation to the one-month postpartum medical examination.
l. CI or SI should investigate the occurrence of adverse events in the subject (mother and fetus). As for the mother, CI or SI should investigate from the starting day of protocol treatment to the observation period after the termination of pregnancy (or day of cancelation of participation in the trial). As for the fetus, CI or SI should investigate from the starting day of protocol treatment to the termination of pregnancy (or day of cancelation of participation in the trial). However, if CI or SI decides to cancel participation in the trial due to the appearance of adverse events, CI or SI should continue the investigation of adverse events due to the test drugs or drugs used concomitantly which are the reasons for the cancelation of participation in the trial, from the cancelation to the one-month postpartum medical examination.
m. CI or SI should investigate the decision day of the termination of pregnancy, the time (day, hour, minute) of delivery, the reasons for termination of the pregnancy (both maternal and fetal causes).
n. CI or SI should investigate the delivery mode, presence/absence of stillbirth and the day of stillbirth, placental weight, and presence/absence of placental infarction.
o. CI or SI can use the data for body weight which is measured and recorded appropriately within 14 days before obtaining consent instead of body weight during screening period.
p. CI or SI can use the data for hematological examination, blood biochemical test, urinalysis, and ECG, which are measured and recorded appropriately within 3 days before obtaining consent instead of those during screening period.
q. CI or SI can use the data for estimated fetal weight, and fetal ultrasound (amniotic fluid volume estimation), which are measured and recorded appropriately within 7 days before obtaining consent instead of those during screening period.
r. The sampling of vaginal flora should be performed before the starting of protocol treatment. It is possible to perform it during the screening period.
s. CI or SI should perform the sampling of vaginal flora for the culture of *Ureaplasma* spp. or PCR of *Ureaplasma* spp. on Day 8 ± 2 days. However, if CI or SI finishs the protocol treatment before Day 8, CI or SI should not perform these samplings.

Table 7.5-2 Schedule of data collection and monitoring for participant (infant)

Working item	At the termination of pregnancy (delivery)	Day 3 after birth (± 1 day)	Day 7 after birth (± 3 days)	Day 28 after birth† (± 2 days)	postmenstrual age of 36 weeks# (± 2 days)	At discharge of infant (± 7 days)	At the cancelation of participation in the trial[a]
Umbilical blood gas analysis	X						
Neonatal findings[b]	X						
Apgar scores[c]	X						
Blood gas analysis[d]	X	X[k]					
Hematological examination[d]	X[i]	X[k]					
Blood biochemical test[d]	X[i, j]	X[k]					
Culture of *Ureaplasma* spp. in throat[e]	X						
PCR for *Ureaplasma* spp. in throat[e]	X						
Culture of *Ureaplasma* spp. in ear cavity[e]	X						
PCR for *Ureaplasma* spp. in ear cavity[e]	X						

Chest X-ray	X		X[l]	X	X[l]		
Total admission days	X	X	X	X	X	X	
Presence/absence, kinds, and duration of respiratory management	X	X	X	X	X	X	
Presence/absence of BPD_{36}					X[m]		
Presence/absence of BPD_{28}				X			
Neonatal diseases[f]	X	X	X	X	X	X	
Treatment for infant[g]	X	X	X	X	X	X	
Serious adverse events[h]	X	X	X	X	X	X	X

† Chief investigator (CI) or sub-investigator (SI) should perform data collection and monitoring at the one-month medical checkup, when the infant discharges within 28 days after birth.

# As for infants with birth at gestational ages <32 weeks PMA, the assessment of BPD_{36} should be performed at 36 weeks PMA or discharge to home, whichever comes first. As for infants with birth at gestational ages ≥32 weeks PMA, the assessment of BPD_{36} should be performed at ≥28 and <56 days postnatal age or discharge to home, whichever comes first.

a. CI or SI should investigate serious adverse events of the infant within 3 days after the cancelation of participation in this trial.

b. CI or SI should investigate the neonatal sex, birth weight (g), birth height (cm), head circumference at birth (cm), and presence/absence of small for dates, light for dates, large for dates, and heavy for dates.

c. The Apgar score should be recorded at 1 and 5 minutes after delivery.

d. The sampling of blood for the blood gas test is acceptable from either artery, vein, or capillary vessels.

e. CI or SI should not take biological specimens from the infant if the mother becomes complicated with COVID-19 infection.

f. CI or SI should investigate the presence/absence of intracranial hemorrhage (ICH), periventricular leukomalacia (PVL), respiratory distress syndrome (RDS), meconium aspiration syndrome (MAS), transient tachypnea in newborns (TTN), prolonged pulmonary hypertension (PPH), sepsis, necrotizing enterocolitis (NEC), local intestinal perforation, symptomatic patent ductus arteriosus (PDA), subglottic stenosis, laryngomalacia, .and other upper airway diseases. CI or SI should also investigate the presence/absence of surgery for the infant. If the infant has any surgeries, CI or SI should investigate the surgical procedure, day of surgery, aim of surgery, and execution of intubation for the surgery.

g. CI or SI should investigate oxygen administration, intravenous/intratracheal administration of steroid, nitrogen monoxide inhalation, surfactant administration, caffeine administration, and azithromycin (AZM) treatment.

h. CI or SI should investigate serious adverse events of infants from the termination of pregnancy to discharge of the infant (or day of cancelation of participation in the trial for the mother).

i. The hematological examination and blood biochemical test at the termination of pregnancy for the infant are possible using cord blood.

j. The examination of IgM is performed only at delivery.

k. The blood gas test, hematological examination, and blood biochemical test should not be performed if the infant's birth weight is under 1,000 g.

l. Chest X-ray at 7 and 28 days after birth should be performed when needed.

m. As for an infant delivered at or after 32 weeks of gestation, the evaluation of BPD_{36} should be performed at 56 days after delivery or at discharge to home, whichever comes first.

7.5.1 ITEMS TO BE OBSERVED AND INVESTIGATED IN SUBJECTS (MOTHER AND FETUS) AND TIME OF IMPLEMENTATION

(1) Subject Background Information

- Age (years)
- Race (of people)
- Blood type (ABO, Rh)
- Smoking
- Date of obtaining written consent
- Pregnancy history (not including this pregnancy)
- History of childbirth (not including this pregnancy)
- History of infertility treatment
  ART (assisted reproductive technique): (multiple choice) 1. in vitro fertilization and embryo transfer (IVF-ET), 2. gamete intrafallopian transfer (GIFT). 3. Intracytoplasmic sperm injection (ICSI), 4. IVF-ET + ICSI, 5. Embryo transfer of frozen-thawed embryo (F-ET), 6. blastocyst transfer (BT), 7. other (please specify)
- Registration Date Pregnancy Weeks
- Height (cm)
- Pre-pregnancy weight (Kg)
- Screening period weight (Kg)
  However, if weight is measured and properly recorded within 14 days prior to obtaining consent, the data may be used for the screening period.
- Complications (diseases, symptoms, etc. possessed at the date consent is obtained)
- Hypertension, white coat hypertension, type I diabetes, type II diabetes, gestational diabetes, psychiatric disorders, epilepsy, digestive disorders, thyroid disorders, collagen diseases, malignant tumors, neurological disorders, respiratory diseases, other
- Previous history (cervical conization, late miscarriage, preterm birth)
- Allergy
- Date of pPROM occurrence, pPROM Pregnancy Weeks of occurrence
- Basis for pPROM diagnosis (multiple choice): 1. Leak, 2. Pooling

Implementation period: Screening period

(2) Physical examination findings

During the course of the study, the subjects (mothers and fetuses) (mothers) will be evaluated for changes in symptoms and findings, and for the presence or absence of new symptoms and findings.

Time of implementation: screening period, Day 1, Day 8, Week 3-*1, at the end of pregnancy, at discharge visits, and at cancelation of participation in the trial.

*1: From Week 3 onward, this should be performed once a week until the point at which a decision is made to terminate the pregnancy.

(3) Status of study drug administration

Enter the following items into the eCRF

- Date and number of times the study drug is administered

- Daily dose of study drug
- Whether there has been an interruption, and if so, the reason for the interruption
- Whether or not medication has been withdrawn, and if so, the reason for such withdrawal

Time of implementation: Treatment observation period

(4) Survey on termination of pregnancy

The date of decision of the termination of pregnancy, the reason for the decision of the termination of pregnancy (maternal and fetal requirements), the date of the termination of pregnancy, and the time the fetus was delivered are investigated and the results are entered into the eCRF.

Time of implementation: At the end of pregnancy

(5) Delivery findings

The mode of delivery (spontaneous delivery, induced delivery, cesarean section, vacuum delivery, or breech extraction) and, in the case of cesarean section, the presence or absence of maternal indication and its description, presence or absence of fetal indication and its description, stillbirth and the date of confirmed death, placental weight, and placental infarction are investigated and the results are entered in the eCRF.

Time of implementation: At the end of pregnancy

(6) Check concomitant medications

Check concomitant medications and enter the results into the eCRF. Uterine contraction suppressants (type also asked), antihypertensive drugs, insulin, psychotropic drugs, antiepileptic drugs, salazopyrine, thyroid drugs, thiamazole/propylthiouracil, antitumor drugs, inhaled steroids, bronchodilators, low-dose aspirin and heparin self-injection are only investigated during screening phase and the results are entered into the eCRF.

Time of implementation: screening period, treatment observation period, observation period after termination of pregnancy, at cancelation of participation in the trial, and follow-up after cancelation of participation in the trial.

(7) Confirmation of adverse events

Adverse events should be identified and the results are entered into the eCRF.

Time of implementation: Treatment observation period, observation period after termination of pregnancy, at cancelation of participation in the trial, and follow-up after cancelation of participation in the trial.

7.5.2 TEST ITEMS AND TIMING IN SUBJECTS (MOTHER AND FETUS)

(1) Vital signs

Vital signs are measured once a day, and the measurement time and results are entered into the eCRF.

Test items: body temperature, pulse rate, systolic and diastolic blood pressure

Time of implementation: screening period, Day 1[*1], Day 8[*1], Week 3-[*2], termination of pregnancy, examination at discharge, and cancelation of participation in the trial.

*1: As a rule, the day of study drug administration should be measured before administration, and if after administration, within 2 hours after the start of administration.

*2: Week 3 and thereafter, once a week until the point at which a decision is made to terminate the pregnancy.

(2) hematological examination

Hematological tests are performed and the results are entered into the eCRF.

Test items: white blood cells (WBC), red blood cells (RBC), hemoglobin (Hb) concentration, hematocrit (Ht) value, platelet (PLT), white blood cell fraction

Time of implementation: screening period*1, Day 8*2, Week 3-*2*3, termination of pregnancy, examination at discharge, and cancelation of participation in the trial.

*1: Data may be used for the screening period if it was performed within 3 days prior to obtaining consent and properly documented.

*2: As a rule, the day of study drug administration should be measured before administration, or if after administration, within 2 hours after the start of administration using the upper arm without intravenous infusion.

*3: Once a week during Week 3, and as needed after Week 4 until the time when the pregnancy is determined to be terminated.

(3) Blood biochemistry test

Hematological tests are performed and the results are entered into the eCRF.

Tests: total protein (TP), albumin (ALB), total bilirubin (T-Bil), aspartate aminotransferase (AST), alanine transaminase (ALT), lactate dehydrogenase (LDH), blood urea nitrogen (BUN), creatinine (Cr), uric acid (UA), sodium (Na), potassium (K), chloride (Cl), calcium (Ca), magnesium (Mg), amylase (Amy), creatine kinase (CK), c-reactive protein (CRP).

Time of implementation: screening period*1, Day 8*2, Week 3-*2*3, termination of pregnancy, examination at discharge, and cancelation of participation in the trial.

*1: Data may be used for the screening period if it was performed within 3 days prior to obtaining consent and properly documented.

*2: As a rule, the day of study drug administration should be measured before administration, or if after administration, within 2 hours after the start of administration using the upper arm without intravenous infusion.

*3: Once a week during Week 3, and as needed after Week 4 until the time when the pregnancy is determined to be terminated.

(4) Urinalysis

Perform a urine qualitative test and enter the results into the eCRF.

Test items: urinary protein, urinary sugar

Time of implementation: screening period*1, Day 8*2, Week 3-*2*3, termination of pregnancy, examination at discharge, and cancelation of participation in the trial.

*1: Data may be used for the screening period if it was performed within 3 days prior to obtaining consent and properly documented.

*2: As a rule, the day of study drug administration should be measured before administration, and if after administration, within 2 hours after the start of administration.

*3: From Week 3 onward, the procedure should be performed once a week until the point at which a decision is made to terminate the pregnancy.

(5) ECG

An electrocardiogram is performed and the results are entered into the eCRF.

Implementation period: Screening period*1

*1: Data may be used for the screening period if it was performed within 3 days prior to obtaining consent and properly documented.

(6) Vaginal Bacterial Culture

Vaginal bacterial culture tests are performed and the results are entered into the eCRF.

Time of implementation: Day 1 (can be in screening phase), Day 8, Week 3 -*1, at the end of pregnancy

*1: After Week 3, perform once a week until the time when the pregnancy is determined to be terminated. Anaerobic culture is not required.

(7) Vaginal ureaplasma culture (central measurement)

If a subject (mother) is found to have novel coronavirus infection, the specimen should not be collected, and the specimen that has been collected should be discarded.

Time of implementation: Day 1 (can be screening period), Day 8 (±2 days)

(8) Ureaplasma vaginalis PCR (central measurement)

If a subject (mother) is found to have novel coronavirus infection, the specimen should not be collected, and the specimen that has been collected should be discarded.

Time of implementation: Day 1 (can be screening period), Day 8 (±2 days)

(9) Placental ureaplasma culture (centrally measured)

Perform when possible. If the subject (mother) is found to have novel coronavirus infection, do not collect the specimen and discard the specimen already collected.

Time of implementation: At the end of pregnancy (at the time of delivery)

(10) Placental Ureaplasma PCR (central measurement)

Perform when possible. If the subject (mother) is found to have novel coronavirus infection, do not collect the specimen and discard the specimen already collected.

Time of implementation: At the end of pregnancy (at the time of delivery)

(11) Amniotic fluid ureaplasma culture and amniotic fluid ureaplasma PCR (central measurement)

As an option, facilities that perform amniocentesis may submit Ureaplasma culture and PCR of amniotic fluid only three times. If the subject (mother) is found to have novel coronavirus infection, the specimen will not be collected and the specimen already collected will be discarded.

Time of implementation: Up to 3 times at the discretion of the facility

(12) Placental Histopathology

Presence of histological chorioamnionitis [Blanc classification (chorioamnionitis, corditis)]

Time of implementation: From the time of pregnancy termination (delivery) to the time of discharge examination.

(13) Estimated fetal weight

Fetal weight is estimated by ultrasonography and the results are entered into the eCRF.

Period of implementation: Screening period,*1 Day 8 (±2 days), Week 3-.*2

*1: Data may be used for the screening period if it was done within 7 days prior to obtaining consent and properly documented.

*2: Week 3 and thereafter, once a week until the point at which a decision is made to terminate the pregnancy.

(14) Fetal ultrasound (amniotic fluid volume, Doppler)

Fetal ultrasound is performed and the results are entered into the eCRF.

Amniotic fluid volume: amniotic fluid pocket and amniotic fluid index are measured by ultrasonography.

Doppler: check for fore-lying of umbilical cord .

Period of implementation: Screening period, Day 8 (±2 days), Week 3-.*1

*1: From Week 3 onwards, this should be done once a week until the point at which a decision is made to terminate the pregnancy.

7.5.3 TEST TO BE OBSERVED AND INVESTIGATED IN THE CHILDREN AND THE TIME OF IMPLEMENTATION

(1) Neonatal findings

The Apgar score (1 and 5 minutes after birth), sex of the newborn, birth weight (g), birth height (cm), birth head circumference (cm), and neonatal growth level are entered into the eCRF.

Neonatal development is assessed as light-for-dates, small-for-dates, appropriate-for-dates, large-for-dates, or heavy-for-dates.

Time of implementation: At the end of pregnancy (at birth)

(2) Length of hospitalization of the child

The duration of the child's hospitalization (total number of days from the date of birth to the date of discharge or transfer) should be investigated and the results are entered into the eCRF. If the child's hospitalization continues at the 36 weeks and 0 days PMA (or at the follow-up after discontinuation), the child will be surveyed until discharge, if possible.

Time of implementation: at the end of pregnancy (at birth), 36 weeks 0 days PMA*1, at the time of discharge of the child

*1: For children born at 32 or more weeks of gestation, the child is evaluated at day 56 or at discharge from the hospital, whichever occurs first.

(3) Respiratory management of the child

Investigate whether the child is on respiratory management and, if so, the duration and purpose of use by respiratory management method (invasive*1, non-invasive*2, other), and enter the information in the eCRF. If the child remains hospitalized at the 36 weeks and 0 days PMA (or at the time of cancelation of participation in the trial), the child will be surveyed until discharge, if possible.

Time of implementation: at termination of pregnancy (at birth), day 3, day 7, day 28*3, 36 weeks 0 days PMA*4, at discharge of the child

*1: Ventilatory management by tracheal intubation

*2: (Synchronized) nasal intermittent positive pressure ventilation ((s)NIPPV), nasal continuous positive airway pressure (NCPAP), nasal continuous positive airway pressure (NDPAP) with expiratory inspiration conversion, noninvasive neurally assisted ventilation

(NIV-NAVA), high flow nasal cannula (HFNC), positive deep breathing airway pressure (SiPAP), external Positive Airway Pressure (BiPAP)
*3: For children discharged by day 28, evaluation is performed at the 1-month check-up.
*4: For children born at 32 or more weeks of gestation, evaluate at day 56 or at discharge, whichever comes first.

（4）Administering oxygen to the child

If oxygen is administered to the infant and if oxygen is administered, the duration, oxygen concentration, oxygen flow rate, and inhaled oxygen concentration (if FiO2 , oxygen mask, mouth oxygen blow down, or hood) by oxygen administration method will be surveyed and entered into the eCRF. If the neonate remaines hospitalized at the 36 weeks 0 days PMA (or at the time of cancelation of participation in the trial), the neonate will be surveyed until discharge, if possible.

Time of implementation: at termination of pregnancy (at birth), day 3, day 7, day 28[*1], 36 weeks 0 days[*2], at discharge of the child
*1: For children discharged by day 28, evaluation is performed at the 1-month check-up.
*2: For children born at 32 or more weeks of gestation, evaluate at day 56 or at discharge from the hospital, whichever comes first.

（5）BPD_{28} presence/absence

The presence or absence of BPD_{28} onset is defined as "the presence or absence of oxygen or respiratory support use at day age 28.".[1)]

Time of implementation: Day 28[*1] (±2 days)
*1: For children discharged by day 28, evaluation is performed at the 1-month check-up.

(6) BPD_{36} presence/absence

The presence or absence of BPD_{36} is defined as "the presence or absence of oxygen or respiratory support use at a 36 weeks PMA (for children born at 32 or more weeks of gestation, the presence or absence of oxygen or respiratory support use at day 56 or discharge, whichever occurs first)" and the severity of the condition is assessed using Table 7.5 -3 BPD severity classification is used.[1)]

<Table 7.5-3 BPD Severity Classification[1)]>

weeks of pregnancy at birth	Less than 32 weeks	More than 32 weeks
Evaluation Period	36 weeks PMA or discharge, whichever comes first	Day 56 or discharge, whichever comes first
At least 28 days on at least 22% oxygen		
Mild BPD	No oxygen use at 36 weeks 0 days PMA or discharge, whichever comes first	No oxygen use until day 56 or discharge from hospital, whichever comes first
Moderate BPD	Less than 30% oxygen use at 36 weeks 0 days PMA or discharge, whichever comes first	Less than 30% oxygen use at day 56 or discharge, whichever comes first

Severe BPD	Requires oxygen or positive pressure ventilation (artificial ventilation or NCPAP) of at least 30% at 36 weeks 0 days PMA or discharge, whichever comes first	Requires oxygen or positive pressure ventilation (artificial ventilation or NCPAP) of at least 30% at day 56 or discharge, whichever comes first

At the time of evaluation, the respiratory status should be assessed and an supplement oxygen reduction test (SORT)[*1] should be performed to determine BPD severity in children who have any (⑧ through ⑪) of the following ① through ⑭. Upper respiratory tract disease is excluded.

① Non-intubated positive pressure ventilation with artificial ventilation NIPPV and NIV-NAVA by tracheal intubation
② More than 30% oxygen administered by mask.
③ Positive pressure ventilation (*) in non-intubated use and receiving at least 30% oxygen
④ Nasal cannula using more than 100% oxygen 251 mL/min
⑤ Discharged on oxygen only (FiO2 0.30 or higher)
⑥ Discharged on oxygen only (FiO2 less than 0.30)
⑦ He/she was discharged from the hospital with a ventilator.
⑧ Oxygen less than 29% and positive pressure ventilation under nonintubated conditions (including high flow, biPAP, and CPAP)
⑨ Oxygen use below FiO2 0.30 in hood or nasal cannula
⑩ Nasal cannula with 100% oxygen at 250 mL/min or less
⑪ Failure to maintain SpO_2 91% or greater on indoor air at 36 weeks 0 days PMA even with oxygen or respiratory assistance on 14 consecutive days prior to 36 weeks 0 days PMA
⑫ Oxygen administration period is less than 28 days
⑬ SpO_2 of 91% or greater for at least 14 days without any respiratory support or oxygenation
⑭ Discharged home without respiratory support or oxygen.

Time of implementation: 36 weeks 0 days (±2 days) PMA, except for children born at 32 or more weeks of gestation, at day 56 or at discharge from the hospital, whichever occurs first. If the patient is unable to perform the SORT due to surgery or other reasons, the SORT should be performed when it is determined that the effects of the surgery or other reason are no longer present.

*1 SORT method[18,19)]

During SORT, CI or SI must observe the infant directly while continuously monitoring with a respiratory heart rate monitor and an SpO_2 monitor. SORT should be started 1 hour after daytime nutrition, whenever possible. SORT consists of the following 3 evaluation periods (1. to 3.):

1. Baseline evaluation period (15 minutes)
2. Oxygen/pressure reduction period
3. Stability observation period

1. Baseline evaluation period (15 minutes)

CI or SI must start SORT after confirming that the infant is stable (SpO_2 90% or more). SpO_2 and cardiopulmonary events should be recorded for 15 minutes. If the infant is unstable (SpO_2 less than 90%), CI or SI should discontinue the test, and should re-evaluate the test within 12 to 24 hours from the start of evaluation. If the infant is unstable at the time of re-evaluation, CI or SI should judge the test as “failure”.

2. Oxygen/pressure reduction period

If the infant is stable for 15 min during the baseline evaluation period (if SpO_2 is maintained at 90% or higher), CI or SI should gradually reduce oxygen, pressure, or flow as follows:

- Pressure for CPAP/directional positive airway pressure (DPAP)/biPAP

The pressure should be gradually reduced to 5 mmHg (about 4 cmH_2O) by 1 mmHg (about 1 cmH_2O) every 5 min. Then, CI or SI should lower the oxygen concentration to room air and stop respiratory support.

- Oxygen

The oxygen dose should be reduced by up to 100 mL/min every 5 min until an infant with nasal oxygen breathes room air. If an infant is oxygenated with a hood or mask, FiO2 should be reduced by up to 0.10.

- High-flow nasal cannula (HFNC)

CI or SI should reduce the flow rate by 1 L/min every 5 min to 3 L/min, and then discontinue HFNC.

3. Stability observation period

Infants with successful withdrawal of oxygen and respiratory support should be observed for 30 min using an SpO_2 monitor and a respiratory heart rate monitor.

CI or SI should record occurrences of cardiopulmonary events and major cardiopulmonary events during the three evaluation periods using the SORT record forms.

- cardiopulmonary events
  - i. Sustained SpO_2 <90% (<5 min)
  - ii. Bradycardia (pulse <80 beats/min) (≥10 sec)
  - iii. Apnea (breathing cessation ≥15 sec)
- Major cardiorespiratory events
  - i. ≥3 consecutive apneas or bradycardia in a 5-min period or during the test period
  - ii. SpO_2 <80% for ≥15 sec
  - iii. SpO_2 <70% requiring stimulation to recover

CI or SI should judge SORT as a success if the infant does not show any major cardiorespiratory events, maintaining SpO_2 at 90% or higher in room air during the stability observation period. On the contrary, CI or SI should judge SORT as a failure in the following two conditions (i or ii):

- i. If major cardiopulmonary events occur during the oxygen/pressure reduction and stability observation period
- ii. If one of the cardiopulmonary events occurs during the oxygen/pressure reduction and stability observation periods, and the attending physicians decide that SORT should not be continued.

（7）Complications in children

Investigate for IVH, PVL, RDS, fetal aspiration syndrome, neonatal transient hypercapnia, prolonged pulmonary hypertension, sepsis, NEC, localized bowel perforation, symptomatic patent ductus arteriosus (PDA), subglottic stenosis, laryngomalacia, upper respiratory tract disease (other).

For IVH, severity (3rd and 4th degree) and diagnostic methods will also be investigated.

For PVL, the site (unilateral or bilateral) and diagnostic methods will also be investigated.

The diagnostic criteria for each complication are defined separately.

Time of implementation: at the end of pregnancy (at birth, day 3, day 7, day 28*1, 36 weeks 0 days PMA*2, at discharge of the child)
*1: For children discharged by day 28, evaluation is performed at the 1-month check-up.
*2: For children born at 32 or more weeks of gestation, evaluate at day 56 or at discharge from the hospital, whichever comes first.

(8) Treatment details for children

Intravenous (duration of administration, daily dose) and inhaled (whether used or not), inhaled nitric oxide (whether used or not), surfactant (whether used or not), caffeine (duration of administration, daily dose), and azithromycin administration (whether used or not) steroids for neonates will be surveyed and entered into the eCRF. In addition, the name of the procedure, date of surgery, purpose of surgery, and whether the child was intubated for the purpose of surgery will be surveyed and entered into the eCRF.

Time of implementation: at the end of pregnancy (at birth, day 3, day 7, day 28*1, 36 weeks 0 days PMA*2, at discharge of the child)
*1: For children discharged by day 28, evaluation is performed at the 1-month check-up.
*2: For children born at 32 or more weeks of gestation, evaluate at day 56 or at discharge from the hospital, whichever comes first.

7.5.4 TEST ITEMS AND TIMING IN THE CHILDREN

(1) Umbilical cord artery blood gas

Umbilical artery blood gas (pH) is measured.
Time of implementation: At the end of pregnancy (at birth)

(2) blood gas test

Blood gas (pH) measurement. arterial, venous, or capillary blood can be measured.
Time of implementation: at the end of pregnancy (at birth), day 3*1
*1: If birth weight is less than 1000 g, no measurement is required.

(3) Hematological examination

Hematological tests shall be able to measure arterial, venous, or capillary blood.
Test items: WBC, RBC, Hb concentration, Ht value, PLT
Time of implementation: at the end of pregnancy (at birth)*1, day 3*2
*1: At the end of pregnancy (at birth), cord blood may be used for measurement.
*2: If birth weight is less than 1000 g, no measurement is required.

(4) Blood Biochemical Tests

Blood biochemical tests shall be able to measure arterial blood, venous blood, or capillary blood.
Tests: blood glucose, T-Bil, AST, ALT LDH, CK, Na K, Cl, CRP, IgM*1
Time of implementation: at the end of pregnancy (at birth)*2, day 3*3
*1: IgM is measured only at the end of pregnancy (at birth).
*2: At the time of termination of pregnancy, measurement with cord blood is also acceptable.
*3: If birth weight is less than 1000 g, no measurement is required.

(5) Pharyngeal and auricular ureaplasma cultures (central measurement)

If the subject (mother) is found to have novel coronavirus infection, the specimen will not be collected.

Date of implementation: Termination of pregnancy (at birth)

(6) Pharyngeal and auricular Ureaplasma PCR (central measurement)

If the subject (mother) is found to have novel coronavirus infection, the specimen will not be collected.

Time of implementation: At the end of pregnancy (at birth)

(7) chest X-ray

Chest X-rays are evaluated for diffuse opacities, diffuse foamy shadows, and irregular cord-like emphysema-like shadows, which are reported on the eCRF.

Time of implementation: at termination of pregnancy (at birth), day 7[*1], day 28[*2], 36 weeks 0 days PMA[*1,*3]

*1: When deemed necessary.

*2: For children discharged by day 28, evaluation is performed at the 1-month check-up.

*3: For children born at 32 or more weeks of gestation, evaluate at day 56 or at discharge from the hospital, whichever occurs first.

7.5.5 HANDLING OF CENTRALLY MEASURED SPECIMENS

Samples of PCR PCR (vaginal ureaplasma PCR, placental ureaplasma PCR, ureaplasma PCR of the pharynx and ear cavity of the infant, and optional amniotic fluid ureaplasma PCR) which will be measured in central laboratory unit are stored in a refrigerator, and samples of culture (vaginal ureaplasma culture, placental ureaplasma culture, ureaplasma culture of the pharynx and ear cavity of the infant, and optional amniotic fluid ureaplasma culture) which will be also measured in central laboratory unit are stored overnight in a refrigerator at (1) 37 degrees Celsius if possible, (2) room temperature (15-25 degrees Celsius) if no culture device is available, or (3) overnight in a refrigerator and stored at -70 to -80 degrees Celsius on the following day. If stored in a refrigerator, they can be stored for several days. Freezing at -70 to 80 degrees Celsius is possible for several months, and the samples should be kept frozen until the day of delivery service for the samples.

The centrally measured specimens should be sent together at room temperature within two months after the child's specimen collection to the "Department of Developmental Medicine, Research Institute, Osaka Women's and Children's Hospital" by delivery service in Category B. As an option, facilities that perform amniocentesis may submit specimens for Ureaplasma culture and PCR of amniotic fluid only three times. However, specimens from subjects (mothers) and their children who have been found to have new-type coronavirus infection will be discarded and not sent to the "Department of Developmental Medicine, Research Institute, Osaka Women's and Children's Hospital". For details, refer to the Central Assay Specimen Handling Procedures.

At the "Department of Developmental Medicine, Research Institute, Osaka Women's and Children's Hospital", cultures of Ureaplasma spp. and Mycoplasma spp. are conducted, and simultaneous culture and PCR of the bacteria are performed to detect the presence of Ureaplasma urealyticum, Ureaplasma parvum, and Mycoplasma hominis.

7.6 PERIOD OF IMPLEMENTATION AND REGISTRATION

Planned implementation period: jRCT registration date - March 31, 2025

Expected registration period: April 1, 2022 - March 31, 2024
Planned observation period: April 1, 2022 - August 31, 2024
jRCT registration date: February 22, 2022

7.7 END OF ENROLLMENT AND OBSERVATION PERIOD

The registration period will end when the target number of patients enrolled in the study is reached. However, subjects who have already given their consent as study candidates at the time the target number of cases is reached may be enrolled even after the target number of cases is reached. In principle, subjects who have not given their consent at the time the target number of patients is reached can not be enrolled.

The observation period will end when the observation of the last enrolled subject's child is completed.

8 METHOD OF OBTAINING CONSENT

8.1 INFORMED CONSENT

Because we recruit women at or over 18 years old, we should obtain written informed consent solely from patients.

CI, SIs, or CRCs should contact patients, explain the contents of study explanatory document, and obtain written informed consent from patients. CI, SI, or CRCs should explain the content of this trial in words that are easy for the patient to understand, using the explanatory document prepared in accordance with the Clinical Research Act and approved by the Jichi Medical University Central Clinical Research Ethics Committee. CI, SI, or CRCs should give the subjects plenty of time to answer questions and consider their participation in this trial. It also emphasizes that consent is based on the free will of the subject, and that even if they do not agree, they should not be treated disadvantageously. When consent to participate in the study is obtained, the signature, explanation date, and consent date of CI (or SI, CRCs) and subject who made the explanation should be stated on two sets of consent forms (for patients, for institutes). The explanatory document and a consent form for patients should be issued, and a consent form for institutes should be kept at the implementing medical institution. Procedures related to the trial must be carried out after obtaining the consent of the patient for any procedure.

During the trial, if new information is obtained that may affect the will of the study subject, which was not expected at the time of obtaining consent, PI should promptly revise the consent explanatory document, and CI or SI should explain it to the study patient, confirm the intention to participate in the study again, and obtain consent. In addition, in the event of a change in the content of the study, etc., the same should be obtained.

8.2 INFORMED ASSENT FOR ADULT PREGNANT WOMEN OVER 18 YEARS OF AGE

Because we recruit women at or over 18 years old, we do not need to perform assent in this study.

8.3 WITHDRAWAL OF CONSENT

Even after agreeing to participate in the trial, if the patient wishes, consent can be withdrawn at any time until just before fixing the data of the analyzed population. When withdrawing consent, CI or SI consults with the patient, confirms the reason for the withdrawal of consent, and explains the observation necessary after the consent withdrawal. Thereafter, the consent withdrawal procedure should involve the consent withdrawal document as much as possible. After withdrawal of consent, participation in the study should be discontinued. In order to ensure the safety of the patient after discontinuing participation in the trial, we should endeavor to conduct later observation, such as confirming the physical condition change of the subject, to the extent possible.

9 ADVERSE EVENTS AND “DISEASE, ETC.”

Details shall be described in the "Procedures for handling cases of “disease, etc.".

9.1 DEFINITION

9.1.1 DEFINITION OF ADVERSE EVENTS

Adverse events in this study are defined as clinically problematic events among all unfavorable symptoms, signs, illnesses, and laboratory abnormalities that occur in subjects (mother, fetus) from the start of protocol treatment to the termination of pregnancy (or when study participation has discontinued). It does not matter if it has a causal relationship with protocol treatment. In infants, they are defined as clinically problematic events among all unfavorable symptoms, signs, illnesses, or abnormal laboratory abnormalities that occur between postpartum and the end of observation. However, IVH, PVL, RDS, meconium aspiration syndrome (MAS), TTN, PPHN, sepsis, NEC, which are defined as neonatal complications in this trial, localized intestinal perforation, and symptomatic PDA are not reported as adverse events in infants; but they are reported as serious adverse events if they fall under the definition of serious adverse events.

9.1.2 DEFINITION OF CRITICAL ADVERSE EVENTS

Critical adverse events in this trial are IUFD, sepsis, ICU admission, multiple organ failure, artificial ventilation, and total hysterectomy in subjects (mother, fetus).

9.1.3 DEFINITION OF SERIOUS ADVERSE EVENTS

If CI or SIs determine that an adverse event meets the following five criteria (1. to 5.), it is considered a serious adverse event:

1. Death or risk of death
2. Those that require hospitalization at a medical institution or extension of the hospitalization period for treatment
3. Disability or risk of disability
4. Serious according to 1-3
5. Congenital diseases or abnormalities in later generations

Hospitalization scheduled before participation in the trial, hospitalization for laboratory purposes, or extension of the hospitalization period is not considered a serious adverse event. If IUFD or child death occurs, it is determined as 1.

9.1.4 DEFINITION OF “DISEASE, ETC.”

Among adverse events, in addition to diseases, disorders, deaths, or infectious diseases, abnormal laboratory test values and various symptom that are suspected to be caused by the implementation of a specific clinical trial are defined as "disease, etc." In addition, that caused by the implementation of clinical study is considered to have a causal relationship with the medicines used in the trial or a causal relationship with the procedure. The causal relationship should be determined by the CI, SI, or PI based on 9.2 Causal Relationship with Study.

9.2 CAUSAL RELATIONSHIP WITH STUDY

CI or SI should determine the causal relationship between the trial and all adverse events that occurred in subjects (mother, fetus) and serious adverse events that occurred in infants. Judgment should be made not only due to the time relationship with the start of intervention treatment, but also due to complications, concomitant medications, study procedures, accidents, and other external factors. The causal relationship should be judged and recorded according to the following two criteria (A and B):

A “Causal or undeniable”: judgment is made according to the following a-c, whether known or unknown to occur during the clinical trial or interventional treatment:

a: Reasonable or possible due to study or interventional treatment.
b: There is a temporal relationship with the study.
c: No other cause is shown, and a causal relationship with study cannot be denied.

B “No causal relationship”: judgment is based on the following d-f:

d: It is not rational to have resulted from study or interventional treatment.
e: No time relationship is shown.
f: Other causes are shown.

9.3 SEVERITY

CI or SI determines the severity of adverse events that occurred in subjects (mother, fetus) in the following three stages (A to C):

A “mild”: It is transient, does not impair daily life, and does not require treatment.
B “moderate”: It causes some problems in daily life and discomfort, and requires treatment.
C “severe”: It markedly impairs daily life, and treatment such as hospitalization is required.

The CI or SI determines the severity of the adverse event in the child using the Neonatal INC version 1.0 Japanese translation.

9.4 PREDICTABILITY

The predictability of adverse events should be determined based on the package insert and interview form. If the nature, severity, or frequency of adverse events does not match, they are considered unknown adverse events.

9.5 COLLECTION AND TRACKING PERIOD

CI or SI should collect and record adverse events starting from the treatment start date. Clinical findings present on the date of consent are considered complications and not adverse events. However, exacerbations of complications are recorded as adverse events. As a rule, “disease etc.” and serious adverse events that are determined to have a causal relationship with the trial should be followed up to recovery or improvement as much as possible. Follow-up should be terminated when CI determines that further recovery is difficult or unfollowable due to death, disability, sequelae, etc. Non-serious adverse events that are not causally related to the trial are not specifically followed up in the trial, regardless of the outcome.

Regardless of the severity of adverse events, any adverse events that occur should be collected and recorded until the end of each subject's participation in the trial.

9.6 ADVERSE EVENT REPORTS

For all adverse events occurring in subjects (mother, fetus), the event name (diagnosis name), onset date, severity, causality, predictability, outcome, and outcome judgment date are investigated, and are entered in eCRF. For infants, only for serious adverse events, the event name (diagnosis name), onset date, severity, seriousness, causality, predictability, outcome (recovery, remission, unrecovered, sequelae, death, unknown), and outcome judgment date are investigated, and are entered in eCRF. Of all adverse events occurring in subjects (mother, fetus) and children, “disease, etc.” should be reported based on 9.6.2 Reporting of Diseases, etc., and serious adverse events should be reported based on 9.6.1 Reporting of Serious Adverse Events.

9.6.1 SERIOUS ADVERSE EVENT REPORTS

If serious adverse events occur in subjects (mother, fetus) or infants, SI who has obtained the information should promptly report it to CI. CI should promptly grasp the seriousness of adverse events and response status, and should consider necessary responses (and treatments). CI should prepare a contact form according to “Pharmaceutical Disease Report (Unified Form 8)” for serious adverse events regardless of whether there is a causal relationship, and should report it to PI and the study secretariat by e-mail. PI and the study secretariat should convey information to CIs of other medical institutions. If a causal relationship cannot be denied, in addition to the procedures in this section, a report will be made based on 9.6.2 Reporting of “Disease, etc.” In addition, CI should adopt necessary measures such as reporting to the director of the implementing medical institution in accordance with the rules and procedure manual of the implementing medical institution.

PI e-mail: okuchi@jichi.ac.jp

Study Secretariat e-mail: pprom.azm.jimu@gmail.com

9.6.2 REPORTING OF “DISEASE, ETC.”

If a serious “disease, etc.” (a serious adverse event for which a causal relationship cannot be ruled out) occurs, the SI who has knowledge of the event will report it to the CI at his/her institution. Upon receiving the report, the CI will report it to the administrator of the medical institution at that institution and notify the PI and the study office. Thereafter, depending on the predictive nature of the “disease, etc.”, PI must report to the Jichi Medical University Central Clinical Research Ethics Committee and the Minister of Health, Labour and Welfare by the reporting deadline.

Submission deadline for unapproved/off-label use

<table>
<tr><th></th><th>Type of adverse events</th><th>Causal relationship</th><th>Predictive nature</th><th>Deadline for Central Clinical Research Ethics Committee</th><th>Deadline for Minister of Health, Labour and Welfare</th></tr>
<tr><td rowspan="3">1</td><td rowspan="3">1 Death or risk of death</td><td rowspan="2">Present</td><td>Possible</td><td>7 days</td><td>7 days</td></tr>
<tr><td>Not possible</td><td>15 days</td><td rowspan="2">-</td></tr>
<tr><td>Absent</td><td>-</td><td>-</td></tr>
<tr><td rowspan="3">2</td><td rowspan="3">2 Hospitalization or extension of the hospitalization period for treatment
3 Disability or risk of disability
4 Death or serious according to 1-3
5 Congenital diseases or abnormalities in later generations</td><td rowspan="2">Present</td><td>Possible</td><td>15 days</td><td>15 days</td></tr>
<tr><td>Not possible</td><td rowspan="2">-</td><td rowspan="2">-</td></tr>
<tr><td>Absent</td><td>-</td></tr>
</table>

When reporting to the Jichi Medical University Central Clinical Research Ethics Committee, Unified Form 8 should be used and prepared by PI with the cooperation of CI or SI from other institutions. In addition, each time PI prepares Unified Form 8, the report should be sent to CI of all medical institutions to share information, and measures such as alerting should be taken as necessary. Furthermore, each CI, including PI, reports the information on the outbreak of "disease, etc." to the director of each implementing medical institute, and takes preventive measures and countermeasures against the outbreak of "disease, etc.", with the cooperation of the director of the implementing medical institution as necessary.

To report to the Minister of Health, Labour and Welfare, in principle, use the Disease Reporting System (https://mh.cr-adr.com/adr/v1/) to prepare Appendix Form 2-1 and send it to the Pharmaceuticals and Medical Devices Agency ([redacted]).

Regarding all "disease, etc." that have occurred in the trial, the status of outbreaks of "disease, etc." should be reported to the Jichi Medical University Central Clinical Research Ethics Committee and directors of the implementing medical institutions in the annual report.

10 CANCELATION AND COMPLETION

10.1 CANCELATION OF PARTICIPATION IN THE TRIAL

If the subject fails to comply with all study schedules, including efficacy and safety assessments, as well as discontinues protocol treatment for the following reasons (1. to 3.), participation in this trial should be canceled. This situation is called "cancelation of participation in the trial".

If the protocol treatment is canceled before it has actually started, no observations should be performed, and appropriate treatment should be given so that the subject is not disadvantaged in

normal practice.

1. When the subject withdraws consent to participate in the study.
2. When "disease, etc." that may lead to death or death occurs.
3. In addition, when the investigator determines that the risk of participating in the trial outweighs the benefits.

Basis for setting
1: Set from an ethical point of view.
2 – 3: Established from the viewpoint of subject safety.

10.2 CANCELATION OF THE ENTIRE TRIAL

If the following situations (1. to 6.) occur and the investigator (CI or PI), Jichi Medical University Central Clinical Research Ethics Committee, or director of the implementing medical institution decides that the entire trial should be discontinued, the entire trial may be canceled. This situation is called "cancelation of the entire trial".

1. When an unpredictable serious "disease etc.". occurs and there is concern about disadvantage to the subject (mother, fetus, infant).
2. When a critical violation/non-compliance with the law and related laws or study plan is found.
3. When a fact that impairs or may impair ethical validity or scientific rationality is obtained.
4. When a critical risk to the subject is identified.
5. When an opinion is given by the Jichi Medical University Central Clinical Research Ethics Committee.
6. When the Minister of Health, Labour and Welfare requests cancelation or makes a recommendation.

In the event of cancelation in the entire trial, the PI will report to the CIs of all the implementing medical institutes, Certified Clinical Research Review Board, and to the administrators of the implementing medical institutions. In addition, the PI will contact the subjects and inform them of the change in the study schedule. In the event of cancelation of the entire trial, the subject will promptly discontinue the protocol treatment, and the safety of the subject (mother and fetus) will be ensured in accordance with the discontinuation criteria in 6.2.6 Criteria for discontinuation of protocol treatment.

10.3 COMPLETION OF THE TRIAL

The day when all the following six items (1. to 6.) are completed and the completion of this trial is announced in the implementation plan is "completion of the trial".

1. End of enrollment of subjects in the trial and end of observation period.
2. Preparation of general report and its summary.
3. Listening to the opinions of Certified Review Board.
4. Submission to the director of the implementing medical institution.
5. Submission to the Minister of Health, Labour and Welfare and publication of the outline of the general report.
6. Reporting to the director of the implementing medical institution.

11 ANTICIPATED BENEFITS AND DISADVANTAGES, AND METHODS TO MINIMIZE RISKS

11.1 ANTICIPATED BENEFITS

Participation in this study may prevent the development of BPD in children who receive treatment with EM or AZM under the ABPC)/SBT combination. This treatment uses ABPC/SBT instead of ABPC, which is expected to treat a wider range of bacteria, and the administration period has been increased from 7 to 14 days, which may cure bacterial infections that could not be cured in 7 days. Therefore, we believe that both the control and test groups can be expected to have an effect on BPD that could not be expected with conventional methods. This study may lead to the establishment of antimicrobial therapy for preterm water withdrawal during preterm labor in Japan.

11.2 ANTICIPATED DISADVANTAGES

Because subjects will be randomly assigned to either the study or control group, they will not be able to choose their preferred treatment. Regardless of which group they are assigned to, they may experience side effects (e.g., diarrhea) from the antimicrobial agents administered. Vascular pain may occur while receiving Erythrocin for Intravenous Infusion or ZITHROMAC Intravenous use.

11.3 METHODS TO MINIMIZE RISKS

Regardless of which group a patient is assigned to in this study, if "disease, etc." attributable to those drugs occur, the patient will be individually considered for treatment, including withdrawal of medication or cancelation of participation in the trial. Venous blood sampling, electrocardiography, and radiographic imaging will be performed using the same techniques as those used in routine medical care, so there will be no increase in risk as a result of participation in the study. The volume of blood drawn from subjects in this study is 10 mL/treatment, which is a medically safe volume. The volume of blood drawn from the children in this study is also 1.0-1.5 mL/treatment, which is a medically acceptable volume. If the birth weight is less than 1000 g, blood tests will not be required.

12 ETHICAL MATTERS AND MATTERS REQUIRING CONSIDERATION

12.1 COMPLIANCE WITH LAWS AND GUIDELINES

This study falls under the category of specifed clinical trial under the Clinical Research Act (Act No. 16 of 2017) because the study drug falls under off-label use. Therefore, this study will be conducted in compliance with the Clinical Research Act and in accordance with ethical principles based on the Declaration of Helsinki.

The materials required by law, including the study protocol and implementation plan, will be reviewed and approved by a Certified Clinical Research Review Board. Thereafter, the CI at each site will obtain permission from the administrator of the respective site, and the PI will complete

registration in jRCT before commencing the study. If there are any changes to these materials, etc., the same procedure will be followed before the changes are put into effect.

Each CI of this study will be required to register with CREDITS (https://www.uhcta.com/uth/member/) and take the "Ethics and Code of Conduct Course" and the "Conducting Clinical Research Course" from the e-learning program before the study begins. However, if the CI has already completed the "Required Courses for Principal Investigators" designated by the Ethics Committee of each institution within one year, the CI will be deemed competent to conduct the study by submitting a record of such course.

12.2 CERTIFIED CLINICAL RESEARCH REVIEW BOARD

This study will be subject to the review and opinion services of the following Certified Clinical Research Review Board. Except in unavoidable cases, the committee to receive review and opinion services will not be changed throughout the entire study period in principle.

Name: Jichi Medical University Central Clinical Research Ethics Committee
Certification Number: CRB320006
Address: 3311-1 Yakushiji, Shimono-shi, Tochigi, 329-0498 Japan
Contact (Tel.): 0285-58-7637
E-mail address: [redacted]

12.3 HANDLING OF PERSONAL INFORMATION, ETC.

12.3.1 ANONYMIZATION

Data containing personal information of subjects (mothers and fetuses) and children to be collected in this study will be transcribed to the electronic case report form without including personal information. The subject identification code (anonymization number) will be a combination of alphanumeric characters that do not have regularity with the medical record ID, and will be a common study code and an institution code with a three-digit alphanumeric case number. In addition, an anonymization correspondence table for identifying individual subjects will be created and stored.

The anonymization correspondence table will be held only by the institution that obtained the subject's consent.

In this study, the Jichi Medical University Central Clinical Research Ethics Committee and the person in charge of monitoring this study may directly inspect and review patients' medical records (e.g., medical charts), but this will be done with caution to avoid information leakage.

12.3.2 RIGHTS OF SUBJECTS AND OTHERS WITH RESPECT TO PERSONAL INFORMATION

Each CI will disclose personal information, notify the purpose of use, correct, add, delete, or suspend on personal information and personal identification codes (anonymization numbers) held in study at the request of subjects. However, if there is a justifiable reason for not responding to the request, such as when there is a risk of harm to the rights of the medical institution, the proper conduct of clinical study, or the rights of the subject him/herself, the reason will be explained to the subject, etc.

12.4 COMPENSATION FOR HEALTH HAZARDS

If the subject (mother) suffers health problems, appropriate treatment will be provided within the scope of insurance coverage. The subject shall bear the co-payment of medical expenses. Since this study is an off-label use of an already-approved drug, the subject will be covered by clinical research insurance to compensate for any health damage. In the event of a health hazard for which a causal relationship with this study cannot be denied, compensation will be provided according to the extent of the health hazard and the terms of the clinical research insurance policy. However, if the subject is negligent, the subject will not be covered. If the physician is at fault, compensation will be provided through the physician liability insurance. The clinical research insurance policy shall be concluded promptly after the initial approval is obtained.

12.5 METHOD OF TREATMENT IF NOT PARTICIPATING IN THE STUDY

If not participating in the study, antimicrobial therapy (penicillin or cephem antibiotics or combination erythromycin) will be administered at each practicing institution. The best treatment appropriate for the subject will be selected even if the subject does not participate in the study.

12.6 PROVISION OF MEDICAL CARE AFTER COMPLETION OF THE STUDY

After termination of pregnancy or completion of protocol treatment, the best treatment for intrauterine infection will be provided within the scope of insurance coverage and in accordance with the subject's wishes.

12.7 HANDLING OF STUDY RESULTS ON GENETIC CHARACTERISTICS, ETC.

Not applicable because this study does not involve any testing or analysis that would yield genetic characteristics of the subjects.

12.8 SUBJECTS’ FINANCIAL BURDEN, AND BURDEN REDUCTION EXPENSES

The study drugs used in this study will be paid for by the research fund of this study, so there will be no financial burden on the subjects. All other costs will be paid by the subject's health insurance and the subject's co-payment. In addition, there is no difference in treatment between the subject and pregnant women with pPROM who do not participate in the study in terms of expenses that are not covered by insurance, such as meals and bed differentials, which are considered to be expensive.

Since the number of hospital visits and examinations is expected to increase as a result of participation in this study, the financial burden on subjects may also be greater than that of regular medical care. To reduce the burden on subjects, a 10,000 yen burden reduction fee will be paid only once during the study period. The burden reduction fee will be explained in advance in the consent document, and the fee will be transferred to the account designated by the subject after participation in the study. Whenever possible, subjects are requested to fill out information on a form indicating

the account number, etc., before leaving the hospital, place the form in a self-addressed envelope, and send it to the Coordinating Office. The Jichi Medical University Accounting Section will bear the transfer fee. The account number and other information will not be used for any purpose other than the payment of burden reduction expenses.

13 RESEARCH FUNDING, CONFLICTS OF INTEREST, AND DISCLOSURE OF INFORMATION

13.1 SOURCES OF FUNDING FOR THIS STUDY

This study is funded by the Japan Agency for Medical Research and Development, Research and Development Fund (Project for Baby and Infant in Research of healTH and Development to Adolescent and Young adult - BIRTHDAY) "Development of Prevention Method for BPD by Azithromycin Administration for Pregnant Women with pPROM at <28 Weeks of Gestation" (PI: Akihide Ohkuchi) and the research fund distributed to the PI from Jichi Medical University.

13.2 CONFLICTS OF INTEREST

Conflicts of interest regarding the study as a whole and conflicts of interest of individual PI, CI, and SI shall be reported prior to the commencement of the study, and the fact shall be confirmed by the respective institutions to which the PI and SIs belong. Conflicts of interest related to the study as a whole and conflicts of interest of individuals shall be reported prior to the commencement of the study, and the fact shall be confirmed at each affiliated institution. If the medical institution has a committee that confirms and advises on the status of conflicts of interest, the fact shall be confirmed by the committee. If no committee has been established at the institution, the conflict of interest should be reported to the head of the study institution or the administrator of the implementing medical institution, and the fact should be confirmed by that person.

A conflict of interest management plan shall be formulated for each implementing medical institution with the results of fact-finding on the status of conflicts of interest, and conflicts of interest shall be managed and disclosed after obtaining approval from the Jichi Medical University Central Clinical Research Ethics Committee. The status of conflicts of interest shall be disclosed in the same manner when the study results are published. The status of conflicts of interest shall also be disclosed to study subjects.

Appropriate re-declaration and approval will be obtained after the commencement of study. For example, when there is a change in the status of conflicts of interest, when adding or changing the CI or SIs, when making periodic reports, or at other appropriate times, the status of conflicts of interest and conflict of interest management plan, etc. should be reviewed, re-declared, and again approved by the Jichi Medical University Central Clinical Research Ethics Committee.

13.3 METHOD OF INFORMATION DISCLOSURE

The study summary and results of this study will be registered in the publication system for clinical trial plan and study summary (jRCT: https://jrct.niph.go.jp/) maintained by the Ministry of Health, Labour and Welfare, and will be made public in jRCT by submitting the clinical trial plan to the Minister of Health, Labour and Welfare. If there are any changes in the content, the jRCT

should be revised, and the changes should be notified to the Minister of Health, Labour and Welfare after obtaining the approval of the Jichi Medical University Central Clinical Research Ethics Committee. In addition, the information should be updated at least every year in accordance with the law.

13.4 PUBLICATION OF RESULTS

The results of the study will be made public through paper submission and conference presentations. In addition, an outline of the summary report and the results regarding the primary outcome will be notified to the Minister of Health, Labour and Welfare and published in jRCT. The outline of the summary report will be submitted to the Minister of Health, Labour and Welfare around March 2025, within one year after the end of the observation period when all collection periods are completed. However, the publication in the jRCT will be made within 2025 after the publication of the article. The first author of a manuscript submitted to a journal should be the CI or SI from the institution with the highest number of registered cases at the end of the study enrollment. The second author should be the CI or SI from the institution with the next highest number of registered cases. Up to two obstetricians and two neonatologists from each institution will be considered as authors. The order of authors after the third author shall be the order of the institution with the largest number of enrolled patients. Mayumi Sako, and Tohru Kobayashi, who contributed to the protocol development for this study, will be the authors. Akihide Ohkuchi, the PI, and Makoto Nomiyama will be co-corresponding authors. For facilities that did not enroll any cases during the enrollment period, their names will be listed in the Acknowledgement.

For the main article on the detection of Ureaplasma spp. and Mycoplasma spp. the PI, Akihide Ohkuchi, and the CI, Itaru Yanagihara, will be the corresponding authors. The CI at each institution shall be the authors (the order shall be alphabetical by last name). Akihide Ohkuchi shall be the last author. In addition, any researcher who has been involved in this study at the Department of Developmental Medicine, Research Institute, Osaka Women's and Children's Hospital may be an author. The first author shall be determined by Itaru Yanagihara.

PI who participated in this study may perform secondary analyses using the data obtained. However, approval from the PI is required to perform secondary analyses. The paper should include the PI and CIs (at least one or more) who participated in this study. CI at each site other than the authors and Mayumi Sako, Tohru Kobayashi, who contributed to the protocol development, should be listed in the acknowledgments. Investigators involved in the secondary analysis may be considered authors even if they were not involved in this study. The investigator who submitted the secondary analysis will be the corresponding author and will determine the first author.

14 STATISTICS

14.1 POPULATION ANALYZED

The analysis target population is as follows: The analysis population for the primary outcome will be the "Full Analysis Set".

1. Full Analysis Set (FAS)

Subject population (mother, fetus/infant) excluding those who fall under the following from all randomized subjects

- Subjects who have never received protocol treatment
- Subjects who received protocol treatment at least once, but data were not obtained after the treatment
- Subjects who do not meet the eligibility criteria

2. Per Protocol Set (PPS)

A group of subjects (mother, fetus/infant) excluding subjects showing deviations associated with protocol treatment and related to prohibited drugs from FAS.

3. Safety analysis target population

All subject populations (mother, fetus/infant) who received at least one protocol treatment.

14.2 STATISTICAL ANALYSIS

14.2.1 SUBJECT BACKGROUND

Background information/primary outcome/secondary outcomes	FAS/PPS	Statistics
Background information		
Age, ethnicity, blood type (ABO, Rh), smoking habit, day of consent, gestational weeks and days at entry into this trial, gravidity, parity, therapy for sterility, body weight (pre-pregnancy, on giving consent), basic complications, any past obstetrical histories, presence/absence of allergy (food, drugs), onset date (gestational weeks and days) of pPROM, findings of pPROM (presence/absence of leak alone, pooling alone, or both leak and pooling)	Mainly FAS, followed by PPS.	Summary statistics (mean, standard deviation, minimum value, median, maximum value)* for continuous variables, and the numbers, frequencies, and 95% CI for discrete variables.

Subject background information will be summarized in the FAS, PPS, and safety analysis populations. For discrete data, frequencies and proportions are calculated for each group; for continuous data, summary statistics (mean, standard deviation, minimum, median, maximum) are calculated for each group.

14.2.2 ANALYSIS OF PRIMARY OUTCOME

Background information/primary outcome/secondary outcomes	FAS/PPS	Statistics
Primary outcome		Intervention group vs. control group
Incidence rate of either moderate/severe BPD_{36} or IUFD at or less than 36 weeks 0 days	Mainly FAS, followed by PPS.	(1) The difference in crude rate, 95% CI. (2) adjusted risk difference and 95% CI by the Mantel-Haenszel method, adjusted for gestational weeks at onset of pPROM (22 weeks 0 days to 23 weeks 6 days, 24 weeks 0 days to 27 weeks 6 days) and institute (institute where early caffeine will be used, institute where early caffeine will not be used)**.

In analyses using FAS, all subjects with missing primary outcome will be treated as having "moderate or greater BPD_{36} or occurrence of death in child up to 36 weeks PMA" for any reason. In analyses using the PPS, subjects with missing primary endpoints will be excluded from the analysis.

A two-tailed test will be conducted with the null hypothesis that "the proportion of moderate or higher BPD_{36} or child deaths up to 36 weeks PMA is equal between groups" and the alternative hypothesis that "the proportion of moderate or higher BPD_{36} or child deaths up to 36 weeks PMA is different between groups." The p-value will be calculated by testing the difference in proportions. The significance level is set at 0.05.

Calculate the percentage of the primary outcome and its 95% confidence interval for each of the intervention and control groups. In addition, the difference in proportions between groups and their 95% confidence intervals will be calculated. This will be the main analysis. The Mantel-Haenszel adjusted risk difference and its 95% confidence interval will also be calculated, adjusted for week of pregnancy (22 weeks 0 days to 23 weeks 6 days, 24 weeks 0 days to 27 weeks 6 days) and facility (early caffeine-using facility, early caffeine-unused facility) as stratification factors.

14.2.3 ANALYSIS OF SECONDARY OUTCOMES

The analysis will be performed primarily in FAS, with a secondary analysis in PPS.

Background information/primary outcome/secondary outcomes	FAS/PPS	Statistics
Secondary outcomes		Intervention group vs. control group
A. Efficacy evaluation items for mother		
Days from the onset of pPROM to delivery	Mainly FAS, followed by PPS.	(1) Cox proportional hazards model: hazard ratio and 95% CI. (2) Kaplan-Meier curve, (3) by stratified Cox proportional hazards model stratified by stratified factors**.
Gestational weeks at delivery		(1) Summary statistics**, (2) box-plots, (3) (1) and (2) according to stratified factors**.
Reasons of pregnancy termination		(1) list 0f reasons for termination of pregnancy, (2) the numbers and frequencies of reasons for pregnancy according to therapy groups, (3) (2) according to stratified factors*.
Findings at delivery (mode of delivery, presence/absence of stillbirth, placental weight)		A: mode of delivery<br>(1) list of mode of delivery (spontaneous vaginal delivery, induced vaginal delivery, cesarean section, vacuum delivery, breech delivery), (2) the numbers and frequencies of the list according to therapy groups, (3) (2) according to stratified factors**.<br>B: presence/absence of stillbirth<br>(1) the numbers, frequencies, and 95% CI, (2) the difference in frequency and 95% CI, (3) adjusted risk difference and 95% CI by the Mantel-Haenszel method, adjusted for stratified factors**.<br>C: placental weight<br>(1) Summary statistics*, (2) Box-plots, (3) (1) and (2) adjusted for stratified factors**.
Incidence rate of IUFD		(1) the numbers, frequencies, and 95% CI, (2) the difference in frequency and 95% CI, (3) adjusted risk difference and 95% CI by the Mantel-Haenszel method, adjusted for stratified factors**.
Incidence rate of histological chorioamnionitis		(1) the numbers, frequencies, and 95% CI, (2) the difference in frequency and 95% CI, (3) adjusted risk difference and 95% CI by the Mantel-Haenszel method, adjusted for stratified factors**.
Rate of cesarean delivery		(1) the numbers, frequencies, and 95% CI, (2) the difference in frequency and 95% CI, (3) adjusted risk difference and 95% CI by the Mantel-Haenszel method, adjusted for stratified factors**.
Incidence rate of placental abruption		(1) the numbers, frequencies, and 95% CI, (2) the difference in frequency and 95% CI, (3) adjusted risk difference and

		95% CI by the Mantel-Haenszel method, adjusted for stratified factors**.
B. Efficacy evaluation items for infant		
Incidence rate of BPD_{28}	Mainly FAS, followed by PPS.	(1) the numbers, frequencies, and 95% CI, (2) the difference in frequency and 95% CI, (3) adjusted risk difference and 95% CI by the Mantel-Haenszel method, adjusted for stratified factors**.
Perinatal death rate (total of IUFD + early neonatal death / total of neonatal live infants)		(1) the numbers, frequencies, and 95% CI, (2) the difference in frequency and 95% CI, (3) adjusted risk difference and 95% CI by the Mantel-Haenszel method, adjusted for stratified factors**.
Rate of infantile deaths until just before discharge of the infants		(1) the numbers, frequencies, and 95% CI, (2) the difference in frequency and 95% CI, (3) adjusted risk difference and 95% CI by the Mantel-Haenszel method, adjusted for stratified factors**.
Birth weight		(1) Summary statistics*, (2) box-plots, and (3) (1) and (2) according to stratified factors**.
Birth height		(1) Summary statistics)*, (2) box-plots, and (3) (1) and (2) according to stratified factors**.
Birth head circumference		(1) Summary statistics*, (2) box-plots, and (3) (1) and (2) according to stratified factors**.
Day of hospitalization (from the birth date to discharge)		(1) Summary statistics*, (2) box-plots, and (3) (1) and (2) according to stratified factors**.
Day of invasive ventilation		(1) Summary statistics*, (2) box-plots, and (3) (1) and (2) according to stratified factors**.
Day of non-invasive ventilation		(1) Summary statistics*, (2) box-plots, and (3) (1) and (2) according to stratified factors**.
Day of oxygen administration		(1) Summary statistics*, (2) box-plots, and (3) (1) and (2) according to stratified factors**.
Incidence rate of infants with surfactant administration		(1) the numbers, frequencies, and 95% CI, (2) the difference in frequency and 95% CI, (3) adjusted risk difference and 95% CI by the Mantel-Haenszel method, adjusted for stratified factors**.
Incidence rate of infants with RDS		(1) the numbers, frequencies, and 95% CI, (2) the difference in frequency and 95% CI, (3) adjusted risk difference and 95% CI by the Mantel-Haenszel method, adjusted for stratified factors**.
Incidence rate of infants with PPHN		(1) the numbers, frequencies, and 95% CI, (2) the difference in frequency and 95% CI, (3) adjusted risk difference and 95% CI by the Mantel-Haenszel method, adjusted for stratified factors**.
Incidence rate of infants with IVH		(1) the numbers, frequencies, and 95% CI, (2) the difference in frequency and 95% CI, (3) adjusted risk difference and 95% CI by the Mantel-Haenszel method, adjusted for stratified factors**.
Incidence rate of infants with PVL		(1) the numbers, frequencies, and 95% CI, (2) the difference in frequency and 95% CI, (3) adjusted risk difference and 95% CI by the Mantel-Haenszel method, adjusted for stratified factors**.
Incidence rate of infants with sepsis		(1) the numbers, frequencies, and 95% CI, (2) the difference in frequency and 95% CI, (3) adjusted risk difference and 95% CI by the Mantel-Haenszel method, adjusted for stratified factors**.
Incidence rate of infants with NEC		(1) the numbers, frequencies, and 95% CI, (2) the difference in frequency and 95% CI, (3) adjusted risk difference and 95% CI by the Mantel-Haenszel method, adjusted for stratified factors**.
Incidence rate of infants with symptomatic PDA		(1) the numbers, frequencies, and 95% CI, (2) the difference in frequency and 95% CI, (3) adjusted risk difference and

		95% CI by the Mantel-Haenszel method, adjusted for stratified factors**.

14.2.4 ANALYSIS OF SAFETY ENDPOINTS

Analysis will be performed on the safety analysis population. Adverse events and side effects will be coded using MedDRA, and the data will be tabulated by System Organ Class (SOC) and Preferred Term (PT).

Background information/primary outcome/secondary outcomes	FAS/PPS	Statistics
Secondary outcomes		Intervention group vs. control group
C. Safety evaluation items for mother and fetus		
Incidence rates of adverse events or side effects	Safety analysis target population	(1) We will perform coding of events using Medical Dictionary for Regulatory Activities (MedDRA), and count the numbers according to System Organ Class (SOC) and Preferred Term (PT). (2) The numbers, frequencies, and 95% CI.
Incidence rates of serious adverse events or serious side effects		(1) We will perform coding of events using MedDRA, and count the numbers according to SOC and PT. (2) The numbers, frequencies, and 95% CI.
Incidence rate of critical adverse events (IUFD, sepsis of mother, admission to ICU of mother, multiple organ disorders of mother, artificial ventilation for mother, hysterectomy for mother		(1) We will perform coding of events using MedDRA, and count the numbers according to SOC and PT. (2) The numbers, frequencies, and 95% CI.
D. Safety evaluation items for infants		
Incidence rates of serious adverse events	Safety analysis target population	(1) We will perform coding of events using MedDRA, and count the numbers according to SOC and PT. (2) The numbers, frequencies, and 95% CI.
E. Other evaluation items in the current trial		
Vital signs (body temperature, pulse, blood pressure)	Mainly FAS, followed by PPS.	(1) Summary statistics*, at each observation point, (2) box-plots
Hematological examination, blood biochemical tests		(1) Summary statistics*, at each observation point, (2) box-plots
Urine protein or urine sugar		(1) Summary statistics*, at each observation point, (2) box-plots
Detection rate of *Ureaplasma* spp. (in vagina, on surface of the placenta, in infantile pharynx, and in infantile ear cavity		(1) the numbers, frequencies, and 95% CI, for *Ureaplasma* spp. in the vagina, on the surface of the placenta, in the infantile pharynx, and in the infantile ear cavity, respectively, (2) the difference in frequency and 95% CI, (3) adjusted risk difference and 95% CI by the Mantel-Haenszel method, adjusted for stratified factors**.

*, Summary statistics: mean, standard deviation, minimum value, median, maximum value.
**, Stratified factors: gestational weeks of onset of pPROM (22 weeks 0 days to 23 weeks 6 days, 24 weeks 0 days to 27 weeks 6 days) and institute (institute where early caffeine will be used, institute where early caffeine will not be used).
Abbreviations: FAS, full analysis set; PPS, per protocol set; BPD_{36}, bronchopulmonary dysplasia (BPD) at week 36 as modified, CI, confidence interval; pPROM, preterm premature rupture of the membranes; IUFD, intrauterine fetal death; BPD_{28}, BPD at 28 days of life; RDS, respiratory distress syndrome; PPHN, persistent pulmonary hypertension in the neonate; IVH, intraventricular hemorrhage; PVL, periventricular leukomalacia; NEC, necrotizing enterocolitis; and PDA, patent ductus arteriosus.

14.2.5 SUBPOPULATION ANALYSIS

Conduct exploratory analysis, such as subpopulation analysis, if necessary.

14.2.6 INTERIM ANALYSIS PLAN

No intermediate analysis is performed.

14.3 CHANGE IN STATISTICAL ANALYSIS PLAN

When changing the statistical analysis plan, the PI and CIs will carefully review the reasons for the change, the impact of the change on the results and the overall study, and the ethical and scientific relevance of the impact. If, as a result of the review, it is determined that the change is appropriate, the study protocol will be revised to clearly state and outline all major changes and modifications related to the primary outcome and its analysis. The method, content, and results of the review regarding the appropriateness of such changes will be documented separately.

The revised study protocol will be reviewed by the Jichi Medical University Central Clinical Research Ethics Committee and its opinion will be heard before any changes are made. Details regarding the changes will also be explained in the summary report.

15 STORAGE AND DISPOSAL OF SAMPLES AND INFORMATION

15.1 STORAGE METHOD/PERIOD

15.1.1 STORAGE METHOD AND PERIOD OF SAMPLES

Samples collected for central measurement in this study will be submitted to the Department of Developmental Medicine, Research Institute, Osaka Women's and Children's Hospital with a subject identification code (anonymization number). The specimens will be frozen and stored in a freezer for pathogen storage in a lockable laboratory of the Immunology Division of the Research Institute. The storage period will be until the end of the study period. If the subject withdraws consent, the specimens will be discarded at that time.

15.1.2 STORAGE METHOD AND PERIOD OF INFORMATION

During the study period, paper information other than medical records will be kept by the person in charge of storage in a lockable locker at each facility. Electronic information will be stored in a locked locker at the Maternal and Fetal Neonatal Intensive Care Unit, Jichi Medical University Hospital. The information on electronic media should be stored in a lockable cabinet in the laboratory.

After the completion of the study, the above paper and electronic data and the following materials will be stored at the Research Office of the Maternal and Fetal Neonatal Intensive Care Unit, Jichi Medical University Hospital. The storage period will be 10 years after the completion of the trial. In facilities other than Jichi Medical University participating in the study, the storage period will be five years after the completion of the trial.

- Study protocol, clinical trial plan, package inserts for study drugs, source documents, etc.
- Explanatory and consent documents for study subjects
- The report on the main evaluation items, the summary report, and the outline of the summary report

- The notification of review results, etc. received from the Jichi Medical University Central Clinical Research Ethics Committee
- Copies of reports to the Minister of Health, Labour and Welfare other than the clinical trial plan
- Monitoring documents
- Contract for the conduct of specified clinical research
- Records related to the management of pharmaceuticals, etc.
- Other important documents related to the study, as designated by the PI

15.2 DISPOSAL METHOD

15.2.1 DISPOSAL METHODS FOR SAMPLES

Samples collected from subjects other than for the central measurement will be discarded immediately after the necessary tests are completed at each facility. Specimens collected for central measurements in this study will be frozen at the central measurement facility with a subject identification code (anonymization number) in a freezer for pathogen storage in a lockable laboratory of the Immunology Division of the Research Institute, and will be disposed of after the study period.

15.2.2 DISPOSAL METHODS FOR INFORMATION

At the time of disposal, appropriate measures such as anonymization should be taken so that specific individuals can not be identified.

15.3 USE OF SAMPLES AND INFORMATION IN NEW RESEARCHES

After the completion of the study, the data collected in this study will be stored at Jichi Medical University for 10 years. If the existing data to be stored are to be used for a new study, the study plan for the new study will be submitted to the Ethics Review Committee for approval before use. In such cases, an information disclosure document will be prepared through an opt-out procedure, and the opportunity for subjects to refuse to participate in the new research will be guaranteed. When providing existing data to researchers at other institutions, the data will be provided within the scope of informed consent, and measures will be taken to ensure that anonymization correspondence tables are not provided and individuals can not be identified.

16 QUALITY CONTROL AND QUALITY ASSURANCE

16.1 ORIGINAL DATA

The source documents in this study are as follows:

Maternal and neonatal medical records, consent forms, anonymization correspondence tables, histopathology results, eCRF records, culture and PCR results related to samples collected in the vaginal, placental, and neonatal pharyngeal and ear cavities at the Research Institute in

Osaka Women's and Children's Hospital, consultation records with the central office and PI, etc., serious adverse event report forms, study drug management tables.

All institutions and each CI should make all clinical research-related records, including source documents, available for direct inspection during monitoring and investigations by Jichi Medical University Central Clinical Research Ethics Committee and regulatory authorities in connection with this specified clinical trial.

16.2 DATA MANAGEMENT

In this study, the National Center for Child Health and Development (NCCHD) Clinical Research Center will conduct data management and data collection using the eCRF. The data is then checked and requested to be corrected on the eCRF as a query. After the data is fixed at the data center, the fixed data will be provided to the person responsible for the analysis. Details will be specified in the data management plan.

16.3 MONITORING

In this study, monitoring will be conducted by the Clinical Research Center of the NCCHD, and will be carried out by monitoring personnel designated by the PI. Details are specified in the monitoring procedures. The name of the person in charge of monitoring will be designated in a separate nomination form.

16.4 AUDIT

This study will not be audited.

16.5 EFFECTIVENESS AND SAFETY EVALUATION COMMITTEE

This study will not establish an efficacy and safety evaluation committee.

16.6 BPD DIAGNOSIS AND EVALUATION COMMITTEE

In order to reduce the diagnostic bias as much as possible and guarantee the reliability of the BPD diagnosis in this study, we decided to blind the assignment of study drugs to third parties who make the final diagnosis of primary outcome. This study design is called as Prospective Randomized Open Blinded End-point study design (PROBE). We set up “BPD Diagnosis and Evaluation Committee”. Diagnosis of BPD and evaluation of severity of BPD is judged by 3 medical experts working in tertiary NICU who are not participating in this study, who do not know to which group the subject is assigned. The BPD Diagnosis and Evaluation Committee will meet approximately once every 6 months. Details are specified separately in the BPD Diagnosis and Evaluation Committee Procedures.

17 REPORTING AND INFORMATION SHARING BASED ON LAWS AND REGULATIONS, ETC.

17.1 SCOPE OF REPORTING AND INFORMATION SHARING

Various reports, notifications, information sharing, etc. should be conducted as follows

	Request for review, report and notification to the Committee	Reporting and submission to the Minister of Health, Labor and Welfare	Provide information to the CIs	Reporting and submission to the administrator*1 of the implementing medical institution
Clinical trial plan (New)	○	jRCT registration ○	○*3	Application for permission
Completion of submission of clinical trial plan to the Minister of Health, Labor and Welfare (completion of public announcement)	○		○	○
Clinical trial plan (Modified)	○	jRCTcorrection ○*2	○*3	Application for permission
9.6.2 reporting for “disease, etc.”	○	△*5	○	○
Non conformity	○*6		○	○
Periodic report	○	○	○*3	○
Primary outcome report*7	○	○	○	○
Completed publication of the primary outcome report			○	○
Summary report	○		○	○
Outline of summary report	○	○	○	○
Study protocol and explanatory consent document (at the completion of the trial)		○	○	○
Cancelation of the entire trial	○	○	○	
Details of the Committee’s opinion			○	○
Complaints and accusations regarding the conduct of study*4			○	○

*1 Reporting and submission to the administrator of the implementing medical institution is conducted by the PI and each CI at their respective institutions.
*2 Submit a revised clinical trial plan and a notification form (Notification Form of Changes to Clinical Trial Plan or Notification Form of Minor Changes to Clinical Trial Plan).
*3 Share information on the results of the review by the Committee.
*4 Make a report for major nonconformities when a complaint or accusation confirms any major nonconformities.
*5 Only diseases within the scope specified in "9.5.3 reporting of “disease, etc." are reported to the Minister of Health, Labour and Welfare.
*6 If a particularly serious nonconformity occurs, it must be promptly reported to the Jichi Medical University Central Clinical Research Ethics Committee for its opinion.
*7 Not required if a outline of summary report is submitted and a primary outcome report is not prepared.

17.2 CREATION AND MODIFICATION, ETC. OF IMPLEMENTATION PLANS

For a new review prior to the start of the study, the PI submits the clinical trial plan to the Jichi Medical University Central Clinical Research Ethics Committee for approval. After approval, the clinical trial plan is registered in jRCT and submitted to the Minister of Health, Labour and Welfare after obtaining the approval of the administrator of each institution for conducting the study. When the submission is accepted and published on jRCT, the Jichi Medical University Central Clinical Research Ethics Committee will be notified and the administrator of the implementing medical institution will be informed. In addition, the information will be provided to each CI, who will report it to the administrator of the medical institution where the study is conducted.

The same should apply when making changes to the clinical trial plan. Notification Form of Minor Changes in Clinical Trial Plan or Notification Form of Changes in Clinical Trial Plan should be submitted to the Jichi Medical University Central Clinical Research Ethics Committee and the Minister of Health, Labour and Welfare together with the changed clinical trial plan.

17.3 PERIODIC REPORTING

Periodic report will be made every year starting from the date on which study information was published in jRCT. The PI prepares a Uniform Form 5 Periodic Report and Appendix Form 3 Periodic Report within two months after each one-year anniversary of the jRCT publication date, and submits them to the Jichi Medical University Central Clinical Research Ethics Committee. If approved by the Committee, the Appendix Form 3 should be submitted to the Minister of Health, Labour and Welfare within one month of the approval.

The PI will provide information to the CIs regarding the content of the periodic report and their approval by the Committee and submission to the Minister of Health, Labour and Welfare. The PI and each CI will also report to the administrators of their respective medical institutions using the aforementioned Periodic Report Form, etc.

17.4 NONCONFORMITY REPORT

If it becomes clear that the clinical trial is not in conformity with this study protocol or with laws and regulations, etc., the SI who has knowledge of the nonconformity will report it to his/her CI. The CI will promptly report to the site administrator and notify the PI of the nonconformity. The PI will provide information to all CIs. The PI and each CI will also report to their respective site administrators. The PI and the CI of the institution where the nonconformity occurred will consider appropriate measures to prevent recurrence.

If the PI determines that the nonconformity that has occurred is a particularly serious nonconformity, the PI will promptly prepare a Uniform Form 7 Serious Nonconformity Report and submit it to the Jichi Medical University Central Clinical Research Ethics Committee for its opinion. Major nonconformities are mainly defined as follows.

- Any impact on study subjects' human rights or safety.
- Any impact on the progress of the study or on the reliability of the results.
- Conducting unapproved study
- When clinical trial is conducted without obtaining consent

If there is concern that the CI may not report the nonconformity to the appropriate reporting party, the SI may report the nonconformity directly.

Among nonconformities, those that are not in accordance with the study protocol in order to avoid immediate danger to study subjects or for other medically unavoidable reasons are not included in serious nonconformities. Therefore, in the event of an unavoidable nonconformity, a report to Jichi Medical University Central Clinical Research Ethics Committee is not required, however, even in such a case, an appropriate record of the nonconformity should be made.

17.5 REPORT OF "DISEASE, ETC."

The PI will report to the Jichi Medical University Central Clinical Research Ethics Committee and the Minister of Health, Labour and Welfare in accordance with Section 9.6.2, Report of "diseases, etc.".

The PI will provide information to the CIs regarding the above report and the nature of the "disease, etc.". In addition, the PI and each CI should report to the administrators of their respective medical institutions.

17.6 REPORTS OF CANCELATION OF THE ENTIRE TRIAL

If the entire trial is cancelated, the PI should prepare a Uniform Form 11 Notification of Cancelation of the entire trial and notify the Jichi Medical University Central Clinical Research Ethics Committee within 10 days of the date of the cancelation of the entire trial. In addition, a Form 4 Notification of Cancelation of Specified Clinical Research will be prepared and submitted to the Minister of Health, Labour and Welfare. Furthermore, information should be provided to the CIs. Even if the entire trial is cancelated, the primary outcome report, summary report, and its outline should be appropriately prepared, and periodic reports and reports of "disease, etc." should be conducted until the trial is completed.

17.7 COMPLETION OF THE TRIAL, AND REPORTING OF MAKING THE FINAL REPORTS

When the PI prepares the primary outcome report, he/she should obtain the opinion of the Jichi Medical University Central Clinical Research Ethics Committee, register it in the jRCT promptly after its approval, and submit it to the Minister of Health, Labour and Welfare for publication. The report and the fact that it has been made public should be provided to the CIs. In addition, the PI and each CI should submit the primary outcome report to the administrators of their respective medical institutions.

When the PI prepares the Summary Report and Appendix Form 1 Outline of Summary Report, he/she will likewise obtain the opinion of the Jichi Medical University Central Clinical Research Ethics Committee, register the summary in jRCT within one month after approval, and submit the study protocol and explanatory consent document to the Minister of Health, Labor and Welfare for publication. In addition, the PI should provide information on the report and publication to the CIs. The PI and each CI will submit a summary report and an outline of the summary report to the administrators of the respective medical institutions, and report on the publication of the summary report. The final study protocol is also attached.

When the outline of the summary report is published in jRCT, the PI should prepare a Uniform Form 12 Notification of Completion of the Trial and notify the Jichi Medical University Central

Clinical Research Ethics Committee regarding the completion of the trial, attaching Appendix Form 1.

17.8 OTHER

When the PI receives an opinion from the Jichi Medical University Central Clinical Research Ethics Committee, the PI will provide information on the contents of the opinion to the CIs. In addition, the PI and each CI will report the contents to the administrator of their respective medical institution. The PI and each CI will then consider appropriate measures, including cancelation, suspension, or completion of the trial, according to the opinions expressed. In the case of cancelation of the entire trial, the report will be in accordance with 10.2 Cancelation of the Entire Trial and 17.6 Report on Cancelation of the Entire Trial.

If a complaint or accusation is received concerning study, the person receiving the complaint or accusation should promptly report it to the CI, then, the CI should report it to the Administrator of the medical institution where the study is conducted and notify the PI. The PI will confirm the contents of the complaint/accusation, consider necessary measures, and provide information to the CIs. The PI and each CI will report to the administrator of their respective medical institution.

18 STUDY SYSTEM

18.1 STUDY ORGANIZATION

○Representative institute and PI
Maternal and Fetal Neonatal Intensive Care Unit, Jichi Medical University Hospital
Professor Akihide Ohkuchi

○The medical institution where the study is being conducted and the CI
See Appendix 1.

○Culture and PCR testing for Ureaplasma spp.
Department of Developmental Medicine, Research Institute, Osaka Women's and Children's Hospital
Director Itaru Yanagihara

○Coordination Secretariat
Department of Obstetrics and Gynecology
Part-time Lecturer Kayo Takahashi

○Support for the Coordinating Secretariat
Department of Clinical Research Promotion, Clinical Research Center, National Center for Child Health and Development
Director Mayumi Sako

○Monitoring Manager

Monitoring Unit, Department of Data Science, Clinical Research Center, National Center for Child Health and Development
Unit Director Mayumi Sako

○Data Management Manager
Data Management Unit, Department of Data Science, Clinical Research Center, National Center for Child Health and Development
Unit Director Miwako Seike

○Statistical Analysis Manager
Center for Information, Jichi Medical University
Associate Professor Makiko Mieno

○Statistical analysis
Biostatistics Unit, Division of Data Science, Clinical Research Center, National Center for Child Health and Development
Researcher Shintaro Iwamoto

○BPD Diagnosis and Evaluation Committee
Division of Neonatology, Center for Maternal-Fetal Neonatal and Reproductive Medicine, National Center for Child Health and Development
Director Tetsuya Isayama

Research and Development Center for New Medical Frontiers, Department of Advanced Medicine, Division of Neonatal Intensive Care Medicine, Kitasato University School of Medicine
Professor Hidehiko Nakanishi

Department of Pediatrics, Saitama Medical Center, Saitama Medical University
Professor Fumihiko Namba

18.2 CONSULTATION SERVICE

Consultations and inquiries from subjects, etc. will be accepted at the consultation desk of each medical institution (Attachment 2). In addition, consultations and inquiries for the study as a whole will be accepted at the following contact points.

Name of medical institution	Jichi Medical University Hospital
Location	3311-1 Yakushiji, Shimono-shi, Tochigi, 329-0498 Japan
Affiliation / Position	Professor, Maternal and Fetal Neonatal Intensive Care Unit, Jichi Medical University Hospital
Name of person in charge	Akihide Ohkuchi (In his absence, part-time Lecturer Kayo Takahashi will be in charge.)
Phone number	0285-58-7376 (Department of Obstetrics and Gynecology)<br>Reception date and time:<br>Mondays from 2:00 p.m. to 4:00 p.m.<br>Tuesdays from 9:00 a.m. to 5:00 p.m.

	Thursdays from 2:00 p.m. to 4:00 p.m.
Email Address	[redacted]

18.3 OUTSOURCING

In this study, the following tasks will be entrusted to the NCCHD Clinical Research Center. The PI will supervise by receiving periodic reports on the implementation of the entrusted work in accordance with the entrustment agreement and by providing guidance as necessary.

- Support for study secretariat operations
- Monitoring
- Data management, eCRF construction and operation
- Statistical Analysis

19 OTHER

19.1 ABBREVIATION LIST

Abbreviation	Name (English)
ART	assisted reproductive technique
BiPAP	biphasic positive airway pressure
BPD	bronchopulmonary dysplasia
CPAP	continuous positive airway pressure
CRO	Contract Research Organization
eCRF	electrical Case Report Form
DPAP	directional positive airway pressure
GIFT	gamete intrafallopian transfer
ICU	intensive care unit
IVF-ET	in vitro fertilization and embryo transfer
IVH	intraventricular hemorrhage
MAS	meconium aspiration syndrome
MedDRA	Medical Dictionary for Regulatory Activities
NCPAP	nasal continuous positive airway pressure
NDPAP	nasal-directional positive airway pressure
NEC	neonatal necrotizing enterocolitis
NICHD	National Institute of Child Health & Human Development
NICU	neonatal intensive care unit
(s)NIPPV	(synchronized) nasal intermittent positive pressure ventilation
NIV-NAVA	non-invasive ventilation-neurally adjusted ventilatory assist
PVL	periventricular leukomalacia
pPROM	preterm premature rupture of the membranes
RDS	respiratory distress syndrome
SOP	Standard Operating Procedure

TTN	transient tachypnea of the newborn

19.2 REVISOIN HISTORY

Version number	Creation-day	Effective date	Changes	Reason for change
Version 1.0	June 29, 2021	na	-	Study plan preparation
Version 1.1	July 30, 2021	na	5. Target group<br>6. Intervention<br>7. Study Methods and procedures<br>18. Study system	Review of procedures, etc. for items listed on the left<br>Review of study structure<br>Maintenance of information
Version 2.0	October 13, 2021	na	5. Target group<br>6. Intervention<br>7. Study Methods and procedures<br>9. Adverse events and "disease, etc."<br>11.3 Methods to minimize risks	Review of target population and methods to minimize risk<br>Review of procedures, etc. for items listed on the left<br>Maintenance of information
Version 2.1	December 28, 2021		2.1 Background<br>4.1 Study design<br>5.1 Eligibility Criteria<br>6.2 Protocol treatment<br>8. Method of obtaining consent<br>10. Cancelation and Completion<br>11.1 Anticipated benefits<br>12. Ethical matters and matters requiring consideration<br>14.3 Change in statistical analysis plan<br>15.3 Use of Samples and information in new research<br>17. Reporting and information sharing based on laws and regulations, etc.	Revised due to suggestions from the Certified Clinical Research Review Committee.<br>Error correction
Version 2.2	January 4, 2022		15. 3 Use of samples and information in new research	For stating that the retention period after the completion of the trial is 10 years.

Version 2.3	April 8, 2022		1.3 Study Schedule<br>2.1 Background<br>5.1 Eligibility Criteria<br>7.5 Observation items and information/procedures to be collected<br>8.1 informed consent<br>9.6.1 Serious adverse event reports<br>12.1 Compliance with laws and guidelines<br>12.8 Subjects' financial burden, and burden reduction expenses<br>13.4 Publication of results<br>15.1.2 Storage method and period of information<br>16.2 Data Management<br>17.4 Nonconformity Report	Error correction<br>Supplemental Explanation added
Version 2.4	May 30, 2022		1.3 Study Schedule<br>7.5 Observation items and information/procedures to be collected<br>7.6 Period of implementation and registration<br>8.1 Informed consent	Supplemental explanation added<br>jRCT registration<br>Information update
Version 2.5	June 27, 2022		6.2 Protocol treatment	Change in Erythrosine tablet dosing regimen
Version 2.6	October 25, 2022		1.1 Overview<br>1.3 Study schedule<br>3 Objective and evaluation items<br>7.5 Observation items and information/procedures to be collected<br>Table 7.5-2 Schedule of data collection and monitoring for participant (infant)<br>7.5.3 Test to be observed and investigated in the children and the time of implementation<br>7.5.4Test items and timing in the children<br>7.5.5 Handling of centrally measured specimens<br>16.6 BPD Diagnosis and Evaluation Committee	Changes due to the establishment of the BPD Diagnosis and Evaluation Committee<br>Change in the method of storing centrally measured specimens and sending specimens to the central measurement facility<br>Error correction

			18.1 Study system	
Version 2.7	February 28, 2023		13.4 Publication of results	Changes to author determination rules
Version 2.8	June 28, 2023		1.1 Overview<br>5.1.1 Selection Criteria<br>5.1.2 Exclusion Criteria<br>7.5 Observation items and information/procedures to be collected<br>Table 7.5-1 Schedule of data collection and monitoring for participant (mother and fetus)<br>Table 7.5-2 Schedule of data collection and monitoring for participant (infant).<br>7.5.1 Items to be observed and investigated in subjects (mother and fetus) and time of implementation<br>7.5.3 Test to be observed and investigated in the children and the time of implementation<br>7.5.4 Test items and timing in the children<br>18.1 Study Organization	Review of exclusion criteria<br>Review of screening period tolerances<br>Error correction<br>Maintenance of information

20 REFERENCES

1. Jobe AH, Bancalari E. Bronchopulmonary dysplasia. Am J Respir Crit Care Med. 2001:163(7):1723-1729.
2. Sriram S, Schreiber MD, Msall ME, et al. Cognitive Development and Quality of Life Associated With BPD in 10-Year-Olds Born Preterm. Pediatrics. 2018:141(6): e20172719.
3. Hanke K, Hartz A, Manz M, et al. Preterm Prelabor Rupture of Membranes and Outcome of Very-Low-Birth-Weight Infants in the German Neonatal Network. Plos One. 2015 Apr 9;10(4):e0122564.

4. Suzuki Y, Horie K, Yada Y, et al. Vaginal Ureaplasma species increase chorioamnionitis in very preterm infants with preterm premature rupture of the membranes at < 28 weeks of gestation. Eur J Clin Meicrobiol Infect Dis. 2018:37(12):2371-2380.
5. Kenyon S, Boulvain M, Neilson JP. Antibiotics for preterm rupture of membranes. Cochrane Database Syst Rev 2013:12:CD001058.
6. How to treat premature rupture of fetal membrane. In Guideline for Obstetrical Practice in Japan 2020; Japan Society of Obstetrics and Gynecology, and Japan Association of Obstetricians and Gynecologists, Eds., Japan Society of Obstetrics and Gynecology, Tokyo, 2020. 142-146p.
7. American College of Obstetricians and Gynecologists' Committee on Practice Bulletins No.217 (2020)
8. Royal College of Obstetricians and Gynaecologists Green-top Guideline No.73 (2019)
9. Society of Obstetricians and Gynaecologists of Canada Reaffirmed Guidelines (2017)
10. Hutzal CE, Boyle EM, Kenyon SL, et al. Use of antibiotics for the treatment of preterm parturition and prevention of neonatal morbidity: a metaanalysis. J Obstet Gynecol. 2008:199(6):620.e1-8.
11. Tanaka S, Tsumura K, Nakura Y, et al. New antibiotic regimen for preterm premature rupture of membrane reduces the incidence of bronchopulmonary dysplasia. J Obstet Gynaecol Res. 2019:45:967-973.
12. Gomez R, Romero R, Nien JK, et al. Antibiotic administration to patients with preterm premature rupture of membranes does not eradicate intra-amniotic infection. J Matern Fetal Neonatal Med. 2007:20:167-173.
13. Ramsey PS, Vaules MB, Vasdev GM, et al. Maternal and transplacental pharmacokinetics of azithromycin. Am J Obstet Gynecol. 2003:188:714-718.
14. Higgins RD, Jobe Ah, Koso-Thomas M, et al. Bronchopulmonary Dysplasia: Executive Summary of a Workshop. J Pediatr. 2018:197:300-308.
15. Isayama T, Lee SK, Yang J, et al. Revisiting the Definition of Bronchopulmonary Dysplasia: Effect of Changing Panoply of Respiratory Support for Preterm Neonates. JAMA Pediatr. 2017:171:271-279.
16. Jensen EA, Dysart K, Gantz MG, et al. The Diagnosis of Bronchopulmonary Dysplasia in Very Preterm Infants. An Evidence-based Approach. Am J Respir Crit Care Med. 2019:200:751-759.
17. Gagliardi L, Amador C, Puglia M, et al. Area-based study identifies risk factors associated with missed antenatal corticosteroid prophylaxis in women delivering preterm infants. Acta Paediatr. 2017:106：250-255.
18. Walsh MC, Wilson-Costello D, Zadell A, et al. Safety, reliability, and validity of a physiologic definition of bronchopulmonary dysplasia. J Perinatol. 2003;23(6):451-456.
19. Marc I, Piedboeuf B, Lacaze-Masmonteil T, et al. Effect of Maternal Docosahexaenoic Acid Supplementation on Bronchopulmonary Dysplasia-Free Survival in Breastfed Preterm Infants: A Randomized Clinical Trial. JAMA. 2020:324(2):157-167.
20. Kim SH, Chun J, Ko KH, et al. Effects of antenatal azithromycin for Ureaplasma spp. on neonatal outcomes. Pediatr Int. 2018: 61:58-62.
