# Supplementary material for: A phase II, multicenter, nonblinded, randomized controlled trial for evaluating protective effects of ABPC/SBT plus, azithromycin versus erythromycin, in pregnant women with pPROM occurring at <28 weeks of gestation on the development of BPD in neonates: Study protocol
Source: PLoS One. 2024 Jul 9;19(7):e0304705. doi: 10.1371/journal.pone.0304705 (PMC11232965; doi:10.1371/journal.pone.0304705)
Supplement: S3 Appendix — (DOCX) [file pone.0304705.s004.docx]

**S3 Appendix**

**Supporting information for the protocol**

**Materials and Methods**

**Recruitment**

The attending physicians should introduce this trial briefly to a candidate woman with pPROM who may fulfill the eligibility assessment, which should be checked by chart review.

CI sends a pamphlet for this trial to potential clinics/hospitals treating women with pPROM occurring at <28 weeks of gestation, and requests referral of such women before starting antibiotics, or if antibiotics are used before referral, either ABPC or ABPC/SBT intravenous administration should be selected.

**Consent to participate**

**Consent and assent**

Because we recruit women at or over 18 years old, we should obtain written informed consent solely from patients. We do not need to perform assent in this study. CI, SIs, or CRCs should contact patients, explain the contents of research explanatory document, and obtain written informed consent from patients. When we receive consent for this trial, we should also obtain consent for the usage of data and materials in future research.

CI, SI, or CRCs should explain the content of this trial in words that are easy for the patient to understand, using the explanatory document prepared in accordance with the Clinical Research Act and approved by the Jichi Medical University Central Clinical Research Ethics Committee. CI, SI, or CRCs should give the subjects plenty of time to answer questions and consider their participation in this trial. It also emphasizes that consent is based on the free will of the subject, and that even if they do not agree, they should not be treated disadvantageously. When consent to participate in the study is obtained, the signature, explanation date, and consent date of CI (or SI, CRCs) and subject who made the explanation should be stated on two sets of consent forms (for patients, for institutes). The explanatory document and a consent form for patients should be issued, and a consent form for institutes should be kept at the implementing medical institution. Procedures related to the trial must be carried out after obtaining the consent of the patient for any procedure.

During the trial, if new information is obtained that may affect the will of the research subject, which was not expected at the time of obtaining consent, PI should promptly revise the consent explanatory document, and CI or SI should explain it to the research patient, confirm the intention to participate in the research again, and obtain consent. In addition, in the event of a change in the content of the research, etc., the same should be obtained.

CI and SI must record the act of explanation and the final answer (acceptance/refusal) on the chart. If the patient does not answer just after the explanation, CI or SI should record his/her name and the date of explanation, and should ask the patient intention afterward. If CI or SI obtains consent at a time different from the explanation time, he/she should record the final answer on the chart later.

The screening test can be performed on the same day as the day of obtaining consent. Alternatively, the screening test must be performed within 3 days after obtaining consent.

**Consent withdrawal**

Even after agreeing to participate in the trial, if the patient wishes, consent can be withdrawn at any time until just before fixing the data of the analyzed population. When withdrawing consent, CI or SI consults with the patient, confirms the reason for the withdrawal of consent, and explains the observation necessary after the consent withdrawal. Thereafter, the consent withdrawal procedure should involve the consent withdrawal document as much as possible. After withdrawal of consent, participation in the research should be discontinued. In order to ensure the safety of the patient after discontinuing participation in the trial, we should endeavor to conduct later observation, such as confirming the physical condition change of the subject, to the extent possible.

**Presence/absence of BPD_36_**

The presence/absence of BPD_36_ is defined as "the presence/absence of oxygen or respiratory support at 36 weeks PMS (if the infant is born at ≥32 weeks PMA, either at ≥28 and <56 days postnatal age, or on discharge to home, whichever comes first)," and the severity rating uses the BPD severity classification [1]. Treatment with oxygen ≥22% for at least 28 days is a necessary condition for the diagnosis of BPD [1]. As for infants with birth at gestational ages <32 weeks PMA, the assessment of BPD_36_ should be performed at 36 weeks PMA or discharge to home, whichever comes first; the severity of BPD is defined as follows: mild BPD is defined when an infant breathes room air at 36 weeks PMA or discharge, whichever comes first; moderate BPD is defined when an infant needs <30% oxygen at 36 weeks PMA or discharge, whichever comes first; and severe BPD is defined when an infant needs ≥30% oxygen and/or positive pressure (positive pressure ventilation [PPV] or nasal continuous positive airway pressure [NCPAP]) at 36 weeks PMA or discharge, whichever comes first [1]. As for infants with birth at gestational ages ≥32 weeks PMA, the assessment of BPD_36_ should be performed at ≥28 and <56 days postnatal age or discharge to home, whichever comes first; the severity of BPD is defined as follows: mild BPD is defined when an infant breathes room air by 56 days postnatal age or discharge, whichever comes first; moderate BPD is defined when an infant needs <30% oxygen at 56 days postnatal age or discharge, whichever comes first; and severe BPD is defined when an infant needs ≥30% oxygen and/or positive pressure (PPV or NCPAP) at 56 days postnatal age or discharge, whichever comes first [1].

**The procedure of SORT**

At the time of evaluation of BPD_36_, a supplement oxygen reduction test (SORT) should be performed to determine the severity of BPD in infants who fulfill any of the following criteria (1. to 4.) [2,3], excluding those with upper respiratory tract diseases:

1. When an infant uses positive pressure ventilation (including high flow, biphasic positive airway pressure [biPAP], continuous positive airway pressure [CPAP]) with oxygen of 29% or less and under non-intubation.
2. When an infant uses oxygen below the fraction of inspiratory oxygen (FiO2) 0.30 in the hood or nasal cannula.
3. When an infant uses 100% oxygen through a nasal cannula at 250 mL/min or less.
4. When an infant uses 22% or more of oxygen for at least 28 days or more, in addition, it is not possible to maintain saturation of percutaneous oxygen (SpO_2_) of 91% or more in room air at 36 weeks 0 days PMA, although the infant has used oxygen or respiratory support for 14 consecutive days before 36 weeks 0 days PMA.

SORT should be performed at 36 weeks PMA for infants born at <32 weeks of gestation; however, it should be performed at 56 days postnatal age or discharge to home, whichever comes first, for infants born at gestational ages ≥32 weeks PMA. If SORT is not able to be performed due to surgery, etc., it should be performed when CI or SI judges that the effects of surgery, etc., have disappeared.

We discussed the procedure of SORT, and constructed the procedure of modified SORT to adapt the available equipment in NICUs in Japan, while referring to the previously published method [2,3].

During SORT, CI or SI must observe the infant directly while continuously monitoring with a respiratory heart rate monitor and an SpO_2_ monitor. SORT should be started 1 hour after daytime nutrition, whenever possible. SORT consists of the following 3 evaluation periods (1. to 3.):

1. Baseline evaluation period (15 minutes)
2. Oxygen/pressure reduction period
3. Stability observation period

1. Baseline evaluation period (15 minutes)

CI or SI must start SORT after confirming that the infant is stable (SpO_2_ 90% or more). SpO_2_ and cardiopulmonary events should be recorded for 15 minutes. If the infant is unstable (SpO_2_ less than 90%), CI or SI should discontinue the test, and should re-evaluate the test within 12 to 24 hours from the start of evaluation. If the infant is unstable at the time of re-evaluation, CI or SI should judge the test as “failure”.

2. Oxygen/pressure reduction period

If the infant is stable for 15 min during the baseline evaluation period (if SpO_2_ is maintained at 90% or higher), CI or SI should gradually reduce oxygen, pressure, or flow as follows:

- Pressure for CPAP/directional positive airway pressure (DPAP)/biPAP

The pressure should be gradually reduced to 5 mmHg (about 4 cmH_2_O) by 1 mmHg (about 1 cmH_2_O) every 5 min. Then, CI or SI should lower the oxygen concentration to room air and stop respiratory support.

- Oxygen

The oxygen dose should be reduced by up to 100 mL/min every 5 min until an infant with nasal oxygen breathes room air. If an infant is oxygenated with a hood or mask, FiO2 should be reduced by up to 0.10.

- High-flow nasal cannula (HFNC)

CI or SI should reduce the flow rate by 1 L/min every 5 min to 3 L/min, and then discontinue HFNC.

3. Stability observation period

Infants with successful withdrawal of oxygen and respiratory support should be observed for 30 min using an SpO_2_ monitor and a respiratory heart rate monitor.

CI or SI should record occurrences of cardiopulmonary events and major cardiopulmonary events during the three evaluation periods using the SORT record forms (**S1 Figure, S2 Figure, and S3 Figure**)

- cardiopulmonary events

1. Sustained SpO_2_ <90% (<5 min)
2. Bradycardia (pulse <80 beats/min) (≥10 sec)
3. Apnea (breathing cessation ≥15 sec)

- Major cardiorespiratory events

1. ≥3 consecutive apneas or bradycardia in a 5-min period or during the test period
2. SpO_2_ <80% for ≥15 sec
3. SpO_2_ <70% requiring stimulation to recover

CI or SI should judge SORT as a success if the infant does not show any major cardiorespiratory events, maintaining SpO_2_ at 90% or higher in room air during the stability observation period. On the contrary, CI or SI should judge SORT as a failure in the following two conditions (i or ii):

1. If major cardiopulmonary events occur during the oxygen/pressure reduction and stability observation period
2. If one of the cardiopulmonary events occurs during the oxygen/pressure reduction and stability observation periods, and the attending physicians decide that SORT should not be continued.

**Definition of “discontinuation of protocol treatment”, “cancelation of participation in the trial”, and “cancelation of the entire trial”**

**Criteria for** **discontinuation of protocol treatment**

If any of the following applies (1. to 6.), the protocol treatment for the subject (mother, fetus) should be discontinued to ensure the safety of the subject (mother, fetus). This situation is called “discontinuation of protocol treatment”. If protocol treatment is discontinued, appropriate antimicrobial treatment should be considered and switched for each subject (mother, fetus).

Even after the protocol treatment is discontinued, the subject (mother, fetus) should continue to participate in the research unless it falls under "Cancelation of participation in the trial”, and CI or SI should continue to observe, investigate, and inspect based on the schedule (**Table 1, Table 2**).

1. When intrauterine infection is strongly suspected due to Lencki's diagnostic criteria [4] or other reasons.

Diagnostic criteria for clinical chorioamnionitis (CAM) by Lencki, et al. [4]

1. If maternal temperature is ≥38.0°C and at least one of the following 4 items (A to D) is observed: A. maternal tachycardia ≥100 beats/min, B. uterine tenderness, C. foul-smelling vaginal discharge or amniotic fluid, D. leukocytosis (≥15 000/mm^3^)
2. If all of the above 4 items are observed even if the mother's body temperature is < 38.0°C

However, since there is a possibility that 1) may occur due to pneumonia, pyelonephritis, appendicitis, meningitis, influenza, etc., these should be differentially diagnosed as much as possible during maternal fever.

1. When *Pseudomonas aeruginosa* or multidrug-resistant bacteria are detected in vaginal culture.
2. When adverse events occur, and it is necessary to withdraw protocol treatment for intravenous drip infusion for 2 days or more.
3. When the delivery is determined.
4. When the subject desires to discontinue protocol treatment.
5. In addition, when the attending physicians determine that it is difficult to continue the protocol treatment.

**Criteria for cancelation of participation in the trial**

If the subject fails to comply with all study schedules, including efficacy and safety assessments, as well as discontinues protocol treatment for the following reasons (1. to 3.), participation in this trial should be canceled. This situation is called “cancelation of participation in the trial”.

If the protocol treatment is canceled before it has actually started, no observations should be performed, and appropriate treatment should be given so that the subject is not disadvantaged in normal practice.

- - - 1. When the subject withdraws consent to participate in the study.
      2. When “disease, etc.” that may lead to death or death occurs.
      3. In addition, when the investigator determines that the risk of participating in the trial outweighs the benefits.

**Criteria for cancelation of the entire trial**

If the following situations (1. to 6.) occur and the investigator, Jichi Medical University Central Clinical Research Ethics Committee, or director of the implementing medical institution decides that the entire trial should be discontinued, the entire trial may be canceled. This situation is called “cancelation of the entire trial”.

1. When an unpredictable serious “disease etc.”. occurs and there is concern about disadvantage to the subject (mother, fetus, infant).
2. When a critical violation/non-compliance with the law and related laws or research plan is found.
3. When a fact that impairs or may impair ethical validity or scientific rationality is obtained.
4. When a critical risk to the subject is identified.
5. When an opinion is given by the Jichi Medical University Central Clinical Research Ethics Committee.
6. When the Minister of Health, Labour and Welfare requests cancelation or makes a recommendation.

**Declaration of completion of the trial**

The day when all the following six items (1. to 6.) are completed and the completion of this trial is announced in the implementation plan is “completion of the trial”.

- 1. End of enrollment of subjects in the trial and end of observation period.
  2. Preparation of general report and its summary.
  3. Listening to the opinions of Certified Review Board.
  4. Submission to the director of the implementing medical institution.
  5. Submission to the Minister of Health, Labour and Welfare and publication of the outline of the general report.
  6. Reporting to the director of the implementing medical institution.

**Data monitoring**

In this trial, a monitoring staff member in National Center for Child Health and Development has monitored the patients. The monitoring are carried out by a monitoring staff member appointed by PI. Details are specified in the monitoring procedure manual for this trial. The name of the person in charge of monitoring is stated separately in the nomination form for this trial.

**Serious adverse events reporting and monitoring**

Adverse events in this study are defined as clinically problematic events among all unfavorable symptoms, signs, illnesses, and laboratory abnormalities that occur in subjects (mother, fetus) from the start of protocol treatment to the termination of pregnancy (or when study participation has discontinued). It does not matter if it has a causal relationship with protocol treatment. In infants, they are defined as clinically problematic events among all unfavorable symptoms, signs, illnesses, or abnormal laboratory abnormalities ​​that occur between postpartum and the end of observation. However, IVH, PVL, RDS, meconium aspiration syndrome (MAS), TTN, pulmonary hypertension in the neonate (PPHN), sepsis, NEC, which are defined as neonatal complications in this trial, localized intestinal perforation, and symptomatic PDA are not reported as adverse events in infants; but they are reported as serious adverse events if they fall under the definition of serious adverse events.

Critical adverse events in this trial are IUFD, sepsis, intensive care unit (ICU) admission, multiple organ failure, artificial ventilation, and total hysterectomy in subjects (mother, fetus).

If CI or SIs determine that an adverse event meets the following five criteria (1. to 5.), it is considered a serious adverse event:

1. Death or risk of death
2. Those that require hospitalization at a medical institution or extension of the hospitalization period for treatment
3. Disability or risk of disability
4. Serious according to 1-3
5. Congenital diseases or abnormalities in later generations

Hospitalization scheduled before participation in the trial, hospitalization for laboratory purposes, or extension of the hospitalization period is not considered a serious adverse event. If IUFD or child death occurs, it is determined as 1.

Among adverse events, in addition to diseases, disorders, deaths, or infectious diseases, abnormal laboratory test values and various symptom that are suspected to be caused by the implementation of a specific clinical trial are defined as "disease, etc." In addition, that caused by the implementation of clinical research is considered to have a causal relationship with the medicines used in the trial or a causal relationship with the procedure.

CI, SI, or PI should determine the causal relationship between the trial and all adverse events that occurred in subjects (mother, fetus) and serious adverse events that occurred in infants. Judgment should be made not only due to the time relationship with the start of intervention treatment, but also due to complications, concomitant medications, research procedures, accidents, and other external factors. The causal relationship should be judged and recorded according to the following two criteria (A and B):

A “Causal or undeniable”: judgment is made according to the following a-c, whether known or unknown to occur during the clinical trial or interventional treatment:

a: Reasonable or possible due to research or interventional treatment.

b: There is a temporal relationship with the research.

c: No other cause is shown, and a causal relationship with research cannot be denied.

B “No causal relationship”: judgment is based on the following d-f:

d: It is not rational to have resulted from research or interventional treatment.

e: No time relationship is shown.

f: Other causes are shown.

CI or SI determines the severity of adverse events that occurred in subjects (mother, fetus) in the following three stages (A to C):

A “mild”: It is transient, does not impair daily life, and does not require treatment.

B “moderate”: It causes some problems in daily life and discomfort, and requires treatment.

C “severe”: It markedly impairs daily life, and treatment such as hospitalization is required.

The predictability of adverse events should be determined based on the package insert and interview form. If the nature, severity, or frequency of adverse events does not match, they are considered unknown adverse events.

CI or SI should collect and record adverse events starting from the treatment start date. Clinical findings present on the date of consent are considered complications and not adverse events. However, exacerbations of complications are recorded as adverse events. As a rule, “disease etc.” and serious adverse events that are determined to have a causal relationship with the trial should be followed up to recovery or improvement as much as possible. Follow-up should be terminated when CI determines that further recovery is difficult or unfollowable due to death, disability, sequelae, etc. Non-serious adverse events that are not causally related to the trial are not specifically followed up in the trial, regardless of the outcome.

Regardless of the severity of adverse events, any adverse events that occur should be collected and recorded until the end of each subject's participation in the trial.

For all adverse events occurring in subjects (mother, fetus), the event name (diagnosis name), onset date, severity, causality, predictability, outcome, and outcome judgment date are investigated, and are entered in eCRF. For infants, only for serious adverse events, the event name (diagnosis name), onset date, severity, seriousness, causality, predictability, outcome (recovery, remission, unrecovered, sequelae, death, unknown), and outcome judgment date are investigated, and are entered in eCRF.

If serious adverse events occur in subjects (mother, fetus) or infants, SI who has obtained the information should promptly report it to CI. CI should promptly grasp the seriousness of adverse events and response status, and should consider necessary responses (and treatments). CI should prepare a contact form according to “Pharmaceutical Disease Report (Unified Form 8)” for serious adverse events regardless of whether there is a causal relationship, and should report it to PI and the research secretariat by e-mail. PI and the research secretariat should convey information to CIs of other medical institutions. In addition, CI should adopt necessary measures such as reporting to the director of the implementing medical institution in accordance with the rules and procedure manual of the implementing medical institution.

If a serious “disease, etc.” (a serious adverse event for which a causal relationship cannot be ruled out) occurs, depending on the predictability of the “disease, etc.”, PI must report to the Jichi Medical University Central Clinical Research Ethics Committee and the Minister of Health, Labour and Welfare by the reporting deadline.

When reporting to the Jichi Medical University Central Clinical Research Ethics Committee, Unified Form 8 should be used and prepared by PI with the cooperation of CI or SI from other institutions. In addition, each time PI prepares Unified Form 8, the report should be sent to CI of all medical institutions to share information, and measures such as alerting should be taken as necessary. Furthermore, each CI, including PI, reports the information on the outbreak of “disease, etc.” to the director of each implementing medical institute, and takes preventive measures and countermeasures against the outbreak of “disease, etc.”, with the cooperation of the director of the implementing medical institution as necessary.

Regarding all “disease, etc.” that have occurred in the trial, the status of outbreaks of “disease, etc.” should be reported to the Jichi Medical University Central Clinical Research Ethics Committee and directors of the implementing medical institutions in the annual report.

**Auditing**

In this study, we do not perform auditing, because this trial is a specified clinical trial, and not a physician-led clinical trial.

**References**

1. Jobe AH, Bancalari E. Bronchopulmonary dysplasia. Am J Respir Crit Care Med. 2001;163:1723−1729.
2. Walsh MC, Wilson-Costello D, Zadell A, Newman N, Fanaroff A. Safety, reliability, and validity of a physiologic definition of bronchopulmonary dysplasia. J Perinatol. 2003;23:451−456.
3. Marc I, Piedboeuf B, Lacaze-Masmonteil T, Fraser W, Mâsse B, Mohamed I, et al. Effect of Maternal Docosahexaenoic Acid Supplementation on Bronchopulmonary Dysplasia-Free Survival in Breastfed Preterm Infants: A Randomized Clinical Trial. JAMA. 2020;324:157−167.
4. Lencki SG, Maciulla MB, Eglinton GS. Maternal and umbilical cord serum interleukin levels in preterm labor with clinical chorioamnionitis. Am J Obstet Gynecol. 1994;170:1345-1351.
