# Supplementary figures and images for: A phase II, multicenter, nonblinded, randomized controlled trial for evaluating protective effects of ABPC/SBT plus, azithromycin versus erythromycin, in pregnant women with pPROM occurring at <28 weeks of gestation on the development of BPD in neonates: Study protocol
Source: PLoS One. 2024 Jul 9;19(7):e0304705. doi: 10.1371/journal.pone.0304705 (PMC11232965; doi:10.1371/journal.pone.0304705)

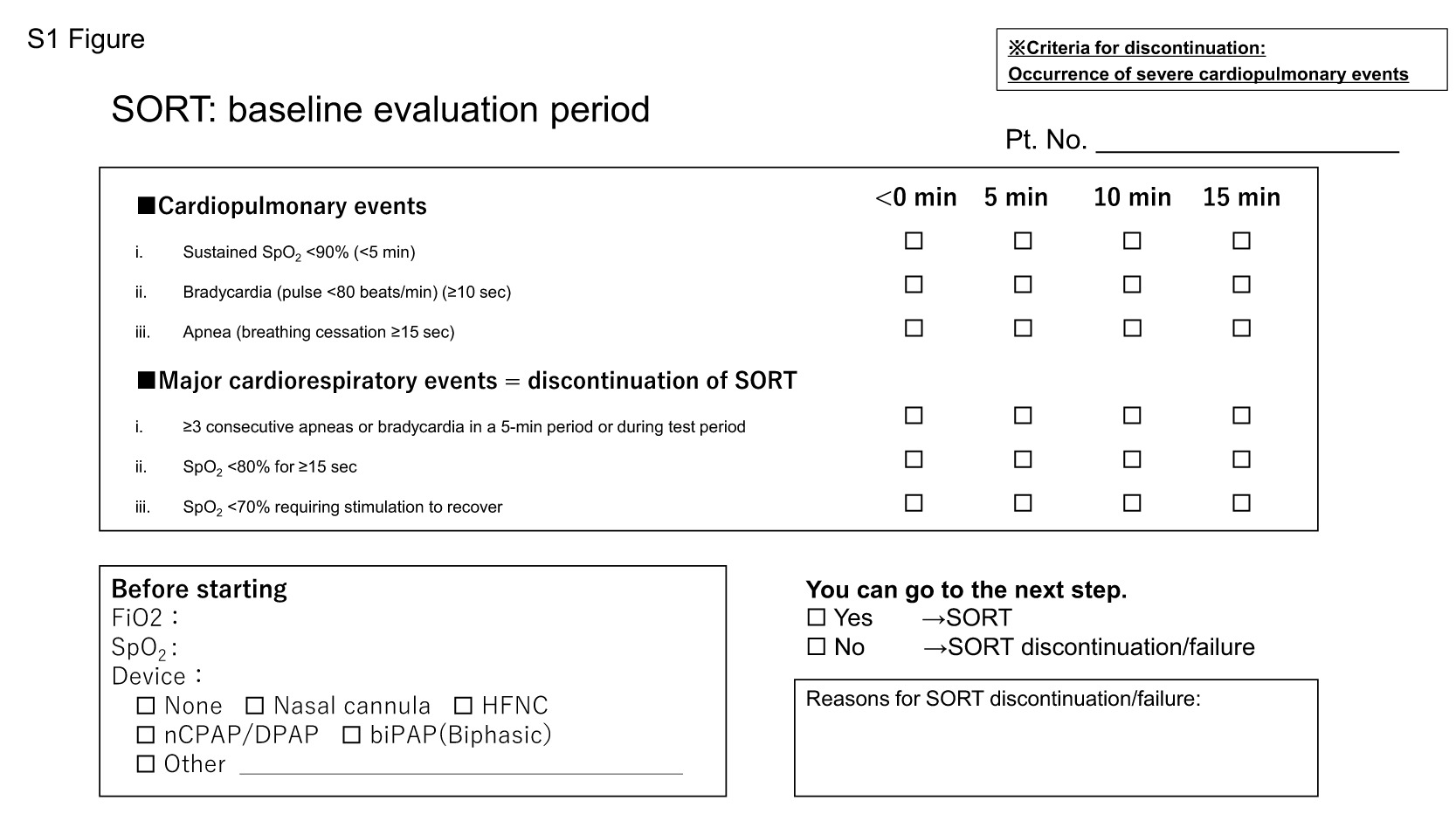

Supplement: S1 Fig — (TIF) [file pone.0304705.s005.tif]

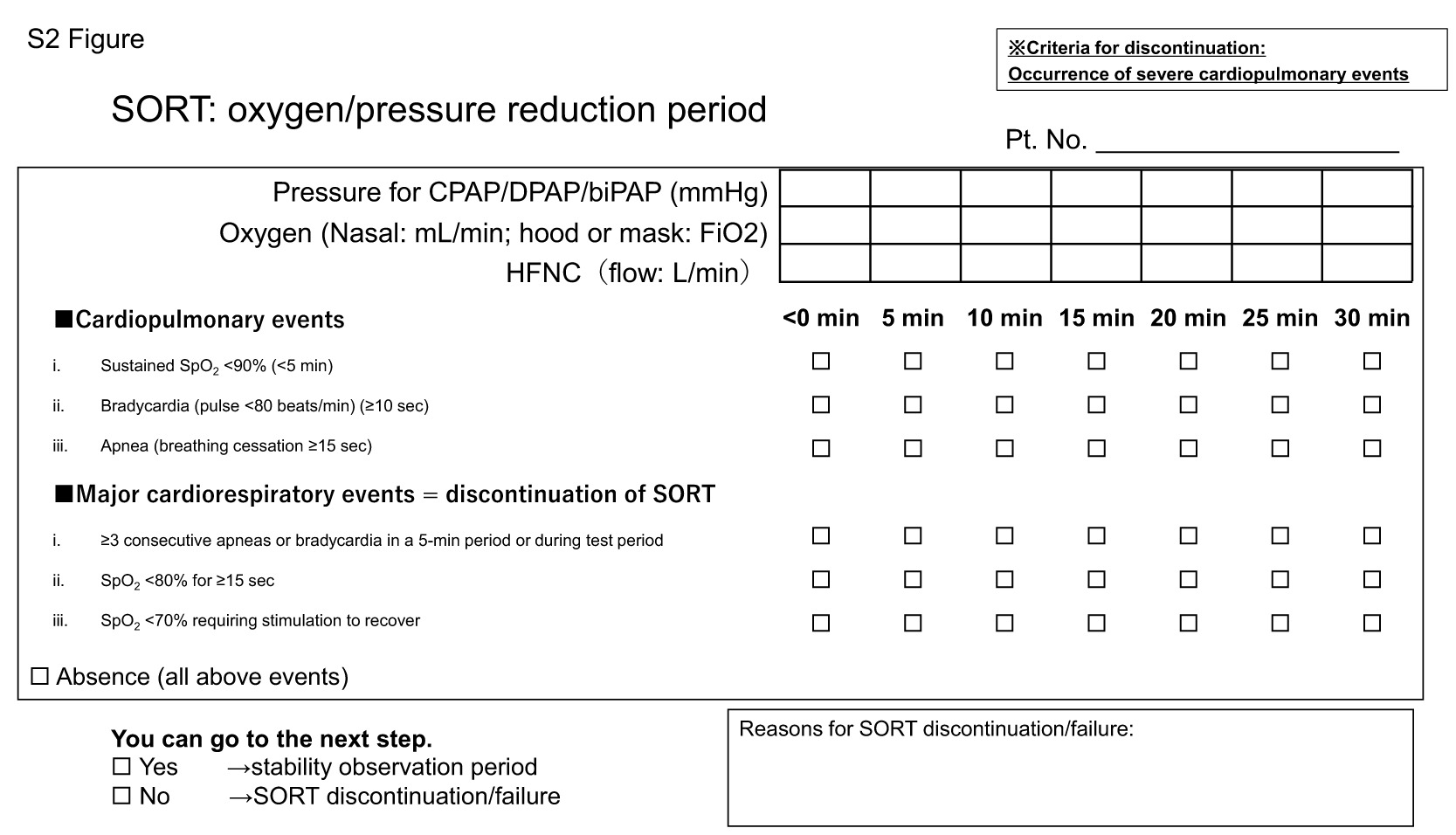

Supplement: S2 Fig — (TIF) [file pone.0304705.s006.tif]

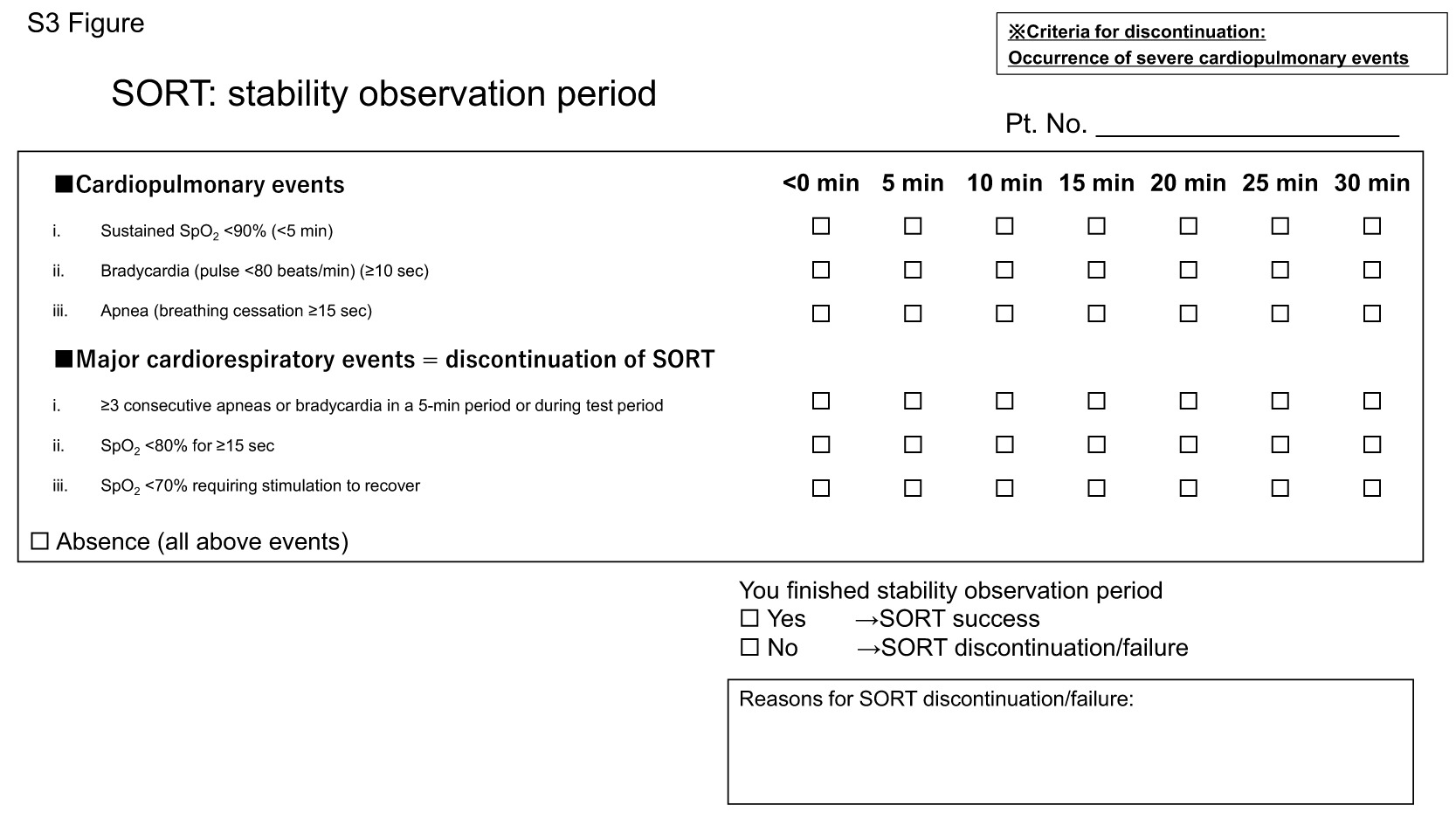

Supplement: S3 Fig — (TIF) [file pone.0304705.s007.tif]
